# Highly Aggressive and Metastatic MDA-MB-231 and Mel Z Cancer Cells Have Common Sets of Down- and Upregulated Genes During Formation of the Vasculogenic Mimicry Phenotype

**DOI:** 10.3390/ijms27114952

**Published:** 2026-05-29

**Authors:** Nickolai A. Tchurikov, Elena S. Klushevskaya, Viktoriya N. Lukicheva, Antonina N. Kretova, Vladimir R. Chechetkin, Galina I. Kravatskaya, Amalia A. Vartanian, Ildar R. Alembekov, Yuri V. Kravatsky

**Affiliations:** 1 Department of Epigenetic Mechanisms of Gene Expression Regulation, Engelhardt Institute of Molecular Biology Russian Academy of Sciences, Moscow 119334, Russia; 2 Department of Experimental Diagnosis and Therapy of Tumors, N.N. Blokhin National Medical Research Center of Oncology of the Ministry of Health of Russia, Moscow 115478, Russia

**Keywords:** RNA-seq, vasculogenic mimicry, Mel Z, MDA-MB-231, development

## Abstract

Vasculogenic mimicry (VM) refers to the capacity of cancer cells from aggressive tumors to form a set of sinuses and channels that mimic normal blood vessels and lack endothelial cells. The rapid growth of a tumor leads to a deficiency in normal vessels, followed by poor oxygen and nutrient supply to tumor cells and VM induction. Understanding the mechanisms behind the development of the VM phenotype is important for the development of new anti-cancer therapies. Previous reports indicate that, during VM formation by melanoma Mel Z cells, about 2000 developmental genes undergo dramatic changes in expression. To identify genes more tightly linked to VM development, we compared the transcriptomes of Mel Z and MDA-MB-231 cells (triple-negative breast cancer cells), which also form VM. Most of the genes that change expression differ substantially between these two cell types. However, we identified 51 up- and 98 downregulated genes common to both cell lines. The non-overlapping groups of these genes are involved in regulating cell adhesion and proliferation. The group of common upregulated genes includes nine genes controlling blood vessel development and tube morphogenesis. Two genes in this group (*BAK1* and *SERPINE1*) rapidly form numerous contacts with nucleoli during VM phenotype formation. We observed that knockdown of the *SERPINE1* gene prevents the development of VM in Mel Z cells. Our data indicate that the formation of VM by aggressive cancer cells might be controlled by a special set of genes.

## 1. Introduction

Identifying the key genes involved in cancer genesis is important for the development of novel therapy approaches. This problem is still hampered by limited knowledge of basic gene expression regulation mechanisms; however, over the last decade, our data on different epigenetic mechanisms, including the roles of short and long noncoding RNAs and dynamic 3D chromosomal structures, have considerably expanded [1,2,3,4,5,6,7].

Vasculogenic mimicry was discovered to be the inherited capacity of aggressive tumor cells to overcome the lack of normal vessels—which leads to hypoxia and nutrient deficit—and to induce the formation of vascular channels and sinuses, formed by the tumor cells themselves, which facilitate tumor perfusion without the formation of normal vessels covered by endothelial cells [8,9,10]. Previous studies have shown that melanoma Mel Z and MDA-MB-231 (MB) cells grown in vitro in 3D on Matrigel or on collagen matrix, respectively, form the VM phenotype [11,12]. After Mel Z cells are transferred from plastic to Matrigel, they demonstrate changes in the expression of about 3000 genes [13]. The cells were obtained from the N.N. Blokhin National Medical Research Center of Oncology at the Ministry of Health, Russia. Upregulated genes mainly correspond to those controlling ribosome function, while downregulated genes mostly include numerous developmental genes. The latter suggests that developmental repression leads to the formation of a less differentiated stemness phenotype in cells during the establishment of VM and melanoma cells with aggressive behavior [11,13]. The detected upregulation of ribosomal genes could indicate that the development of stemness phenotypes requires the activation of biogenesis and ribosome activity in fast-growing tumor cells. However, these data do not elucidate which key genes are characteristic of the development of the vascular channels and sinuses essential for the formation of the VM phenotype.

We aimed to compare the data previously obtained on melanoma Mel Z cells with those of another cell line that also forms VM: MB cells. The goal was to identify common genes whose expression changes during VM phenotype development and which, therefore, may be involved in the formation of vascular channels and sinuses in both cell lines, and which could then be considered candidate genes associated with the formation of a VM-like phenotype. MB cells were originally isolated from a pleural effusion from a metastatic triple-negative breast cancer patient at MD Anderson Cancer Center in 1973 [14]. Mel Z melanoma cells were obtained at the Research Institute of Experimental Diagnostics and Therapy of Tumors of the Blokhin National Medical Research Center for Oncology of the Ministry of Health of Russia from metastasis to the lymph node of a patient with disseminated melanoma in 2003 [11]. We found that most up- and downregulated genes differed between these cell lines during VM phenotype formation. Nevertheless, we also detected common genes, which could be involved in the development of the VM phenotype.

## 2. Results

### 2.1. Transferring MB Cells from a Plastic Surface to a 3D Matrix Dramatically Changed the Expression of About 2700 Genes

**Figure 1:**
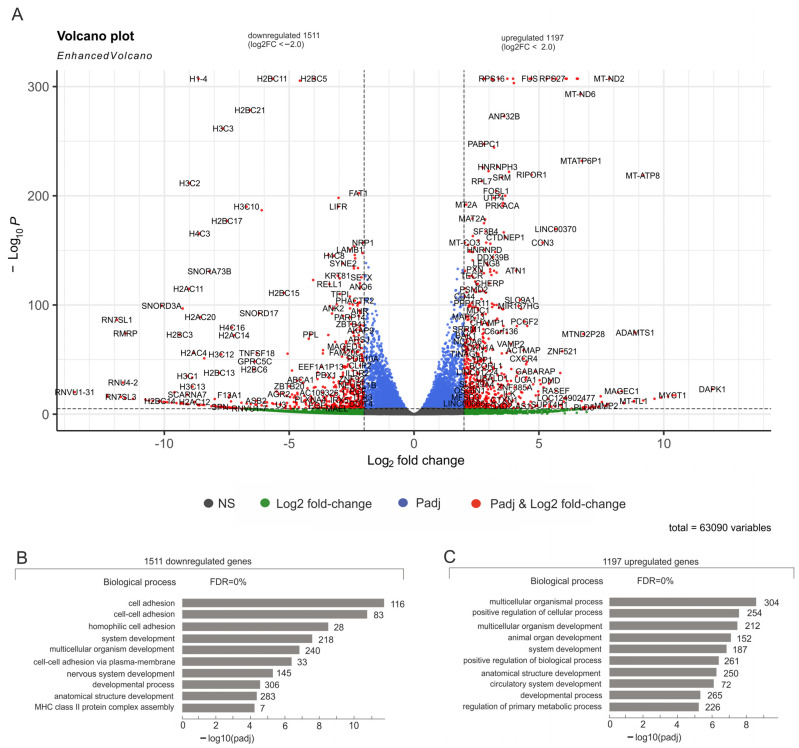
The effects of the growth of MB cells on a 3D matrix. (**A**) Differential expression of genes in MB cells cultured either on plastic or a 3D matrix. The volcano plot shows statistically significant log_2_ fold changes in expression, determined with DESeq2 [18], from RNA-seq experiments. *p*-values were corrected using the built-in Benjamini–Hochberg method for multiple testing in DESeq2. (**B**) Top 10 Gene Ontology (GO) biological process associations of 1511 downregulated genes using Profiler (https://biit.cs.ut.ee/gprofiler/gost, accessed on 11 December 2025). Values to the right of the bars show the number of genes associated with a process. The complete list of corresponding genes, including lincRNA genes, is shown in Table S2. (**C**) Top 10 Gene Ontology (GO) biological process associations of 1197 upregulated genes. Values to the right of the bars show the number of genes associated with a process. The complete list of corresponding genes is shown in Table S3.

Previously, RNA-seq data were described for MB cells cultivated either on plastic [15] or on a *3D matrix* [12]. Similarly to the corresponding data obtained for Mel Z cells [13], we observed dramatic changes in gene expression following transfer. Figure 1A and Table S1 show that under |log2FC| ≥ 2.0, there are 1511 downregulated genes and 1197 upregulated genes.

The top ten GO items for downregulated genes mainly include genes involved in cell adhesion and development (Figure 1B). The complete data on GO analysis are shown in Table S2, and the complete list of 63,090 transcribing genes and their expression values on plastic and 3D matrix are shown in Table S1. The GO analysis of these genes was performed using g:Profiler software (https://biit.cs.ut.ee/gprofiler/gost, accessed on 11 December 2025). Using the “Highlight driver terms in GO” option [16], we observed that the drivers for the downregulated genes are “system development” (*p*-value = 3.6 × 10^−7^) and “cell adhesion” (*p*-value = 3.7 × 10^−11^) (Figure S1). The products of the corresponding genes are highly associated either with nuclei “nucleosomes” (*p*-value = 4.5 × 10^−8^) or “cell periphery” (*p*-value = 1.4 × 10^−13^). The downregulated genes in MB cells include 24 ZNF genes, 6 integrin genes (*ITGB4*, *ITGB6*, *ITGB8*, *ITGBL1*, *ITGA1*, and *ITGA10*), and 107 lincRNA genes (Table S1). In addition, 23 downregulated histone genes were indicated at the “structural constituent of chromatin” item (Table S2, Figure 1A), as well as 22 *PCDH* genes specifying the membrane protein found at cell–cell boundaries and involved in neural cell adhesion.

Based on previous Mel Z data, we expected that the fast-growing MB cells transferred to a 3D matrix would also possess upregulated genes connected with ribosome function [13]. However, we observed that the top 10 biological processes in which upregulated genes in MB cells are involved differ. The entire list of upregulated genes in MB cells is shown in Table S1, and the complete data on the GO analysis of upregulated genes are shown in Table S3. The top ten GO items for upregulated genes include genes controlling multicellular organismal development, the positive regulation of cellular processes and development, and genes involved in circular system development and vasculature development (Figure 1C). Among the upregulated genes, there are also genes involved in blood vessel development, blood vessel morphogenesis, tube development, cell migration, and tube morphogenesis (Table S3).

Analysis of the driver terms for upregulated genes revealed that they include the “multicellular organismal process” (*p*-value = 2.8 × 10^−8^), “positive regulation cellular process” (*p*-value = 2.3 × 10^−7^), and “cell junction” (*p*-value = 3.9 × 10^−6^), and the products of upregulated genes are mainly located in the “nucleoplasm” (*p*-value = 2.9 × 10^−3^) (Figure S2). Upregulated genes in MB cells include 16 ZNF genes and 83 lincRNA genes (Table S1). The volcano plot in Figure 1A shows that, among the upregulated genes, numerous mitochondrially encoded genes involved in electron transfer are closely related to the activation of signaling pathways during various life processes [17].

### 2.2. There Are 98 Common Downregulated Genes in MB and Mel Z Cells Cultivated on a 3D Matrix

**Figure 2:**
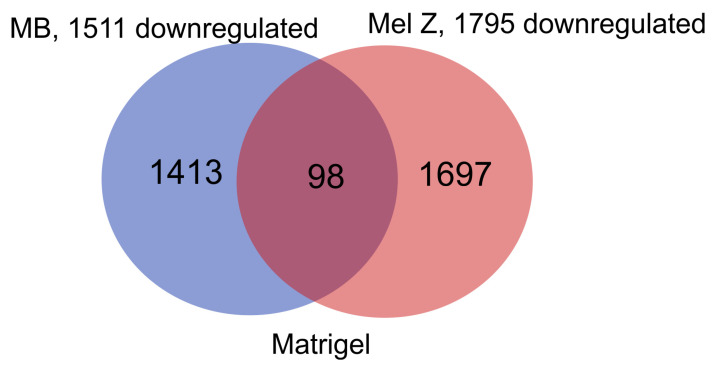
A Venn diagram showing intersections between downregulated genes detected in MB and Mel Z cells grown on a 3D matrix. The complete list of the overlapping genes is shown in Table S4. The intersection is significantly higher than what would be expected by chance (*p* = 7.56 × 10^−33^).

Next, we used the data on downregulated genes detected in MB cells grown on a 3D matrix (Figure 1A, Table S1) to identify common downregulated genes previously described in Mel Z cells also forming VM [13]. As expected, we observed that only a small proportion of downregulated genes in the two cell lines were shared (Figure 2). The MB line originates from mammary epithelial cells [19], whereas melanoma Mel Z cells originate from melanocytes; the pigment-producing cells in skin, hair, and eyes; and embryonic neural crest cells [20]. The data clearly indicate that cancer cells of different origins exhibit markedly different gene expression patterns. It follows that the development of the VM phenotype requires the downregulation of distinct sets of genes, at least in these two cell lines. Nevertheless, our data indicate that there are also common genes that should be repressed during VM formation.

Table 1 shows that there are 98 common downregulated genes in these two cell lines. The driver terms for this set of genes include “homophilic cell adhesion via plasma membrane adhesion molecules” (*p*-value = 2.8 × 10^−3^), “regulation of cell population proliferation” (*p*-value = 3.4 × 10^−3^), “nervous system development” (*p*-value = 1.4 × 10^−2^), and “developmental process” (*p*-value = 4.6 × 10^−2^) (Figure S3). It follows that repression of these genes is required for the development of the VM phenotype and for the formation of less differentiated cancer cells, at least in MB and Mel Z cells. Table 1 shows the Gene Ontology (GO) biological process associations of these 98 genes, including “cell population proliferation” and “system development”.

The search for KEGG associations of these 98 genes revealed their involvement in the P53 signaling pathway. These genes also regulate DNA damage, DNA integrity checkpoints, and microtubule cytoskeletal structures (Figure S4). It follows that the downregulation of this set of genes would lead to disturbances in tumor repression, the prevention of abnormal cell proliferation, and the maintenance of genomic stability.

### 2.3. There Are 51 Common Upregulated Genes in MB and Mel Z Cells Cultivated on 3D Matrix

**Figure 3:**
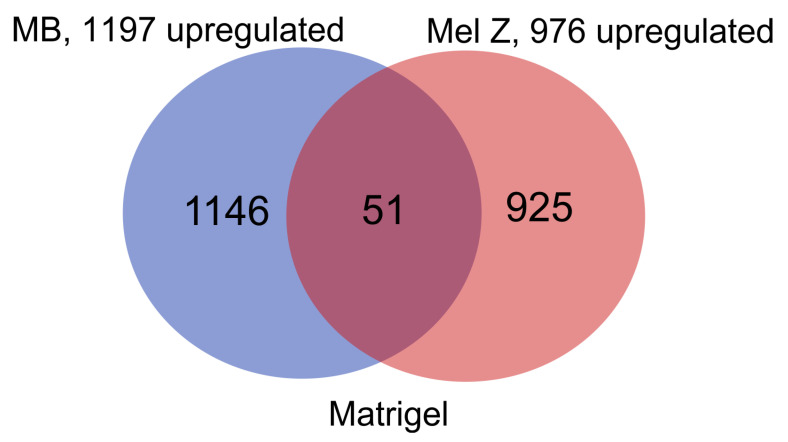
Venn diagram showing intersections between upregulated genes detected in MB and Mel Z cells forming the VM phenotype. The complete list of the overlapping genes is shown in Table S5. The intersection is significantly higher than what would be expected by chance (*p* = 9.94 × 10^−11^).

Similarly, the search for common upregulated genes in MB and Mel Z cells revealed only 51 genes (Figure 3, Table S5). This group of 51 genes is associated with epithelial cell–cell adhesion, cell population proliferation, and blood vessel development (Table 2). The products of these genes are located in the nucleoplasm (Table 2, Figure S5). The driver terms for this set of genes include the “regulation of cell population proliferation” (*p*-value = 1.5 × 10^−2^), “blood vessel development” (*p*-value = 4.3 × 10^−2^), and “nucleoplasm” (*p*-value = 2.8 × 10^−3^) (Figure S5).

These genes are associated with the HIF-1 signaling and transcriptional misregulation pathways (Figure S6). The Hypoxia-Inducible Factor 1 (HIF-1) signaling pathway is involved in hypoxia adaptation, which evolved VM through the activation of genes for angiogenesis and proliferation [21]. The transcriptional misregulation pathway refers to the misregulation of the dynamic interplay between transcription factors, cofactors, epigenetic modifiers, noncoding RNAs, and chromatin architecture [22]. The misregulation of transcriptional networks is common for different diseases, including cancer and cardiovascular diseases [23].

Taken together with the data on the 98 downregulated genes, it follows that the development of VM in MB and Mel Z cells leads to the downregulation of specific sets of genes controlling cell adhesion, proliferation, system development, and developmental processes. This is coupled with the upregulation of another set of genes involved in cell adhesion and proliferation. At the same time, a small group of upregulated genes controls blood vessel development (Table 1 and Table 2).

### 2.4. Nine Common Upregulated Genes Controlling Blood Vessel Development Are Also Involved in Tube Morphogenesis and Development in MB and Mel Z Cells

Normal blood vessel development requires progenitor cell differentiation into endothelial cells under the control of Vascular Endothelial Growth Factor (VEGF) and other factors [24,25,26]. The VM phenotype formed by MB and Mel Z cells on a 3D matrix forms quickly (15–24 h) by the cancer cells themselves [12]. The only common upregulated genes related to the development of vascular-like structures are the nine genes that control blood vessel development, as indicated in Table 2. To understand the biological processes closely related to this group of genes, we carried out a more detailed search of the GO database. Table 3 shows the top ten functions for these genes. The functions of this small set of genes include vasculature and circulatory system development, as well as tube morphogenesis and development (the complete list is shown in Table S6).

This small group of genes is involved in the HIF-1 pathway (Figure S7), which facilitates cell plasticity and tube formation [26,27]. HIF-1 has been reported to promote vasculogenic mimicry formation [26]; our data agree with this observation. It has been previously described that the ability of cancer cells to form tubes on a 3D matrix could be used as a standard in vitro assay [26,28].

### 2.5. Analysis of Expression of Common 98 Downregulated Genes in MB and Mel Z Cells

**Figure 4:**
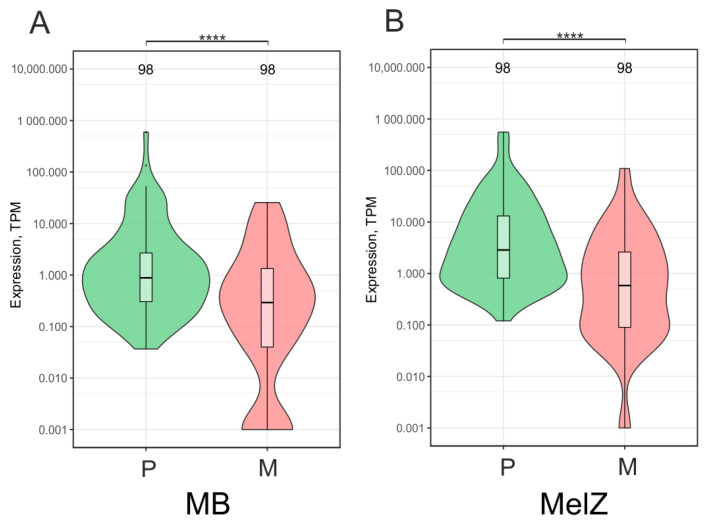
Violin plots showing the expression levels of 98 common downregulated genes in MB (**A**) and Mel Z cells (**B**) cultivated either on plastic (P) or on a 3D matrix (M). TPM—Transcripts Per Million. **** *p*-values < 10^−6^.

We observed that the downregulated genes detected in both cell types primarily regulate cell adhesion and systemic development (Figure 1B and Figure S2). It follows that, when cultivated on a 3D matrix, cells of both types should be less differentiated and more aggressive. The violin plots in Figure 4 show the levels of downregulation for these gene groups in MB and Mel Z cells. On a 3D matrix, MB cells exhibit more prominent downregulation of this group of 98 genes, including a fraction of practically silent genes (Figure 4A). At the same time, in Mel Z cells, the group of practically silent genes at the very bottom of the violin is several times smaller (Figure 4B). Given that these 98 genes are involved in system development, we suppose that the Mel Z cells exhibit stronger RNA expression of the same gene sets.

### 2.6. Analysis of Expression of Common 51 Upregulated Genes in MB and Mel Z Cells

**Figure 5:**
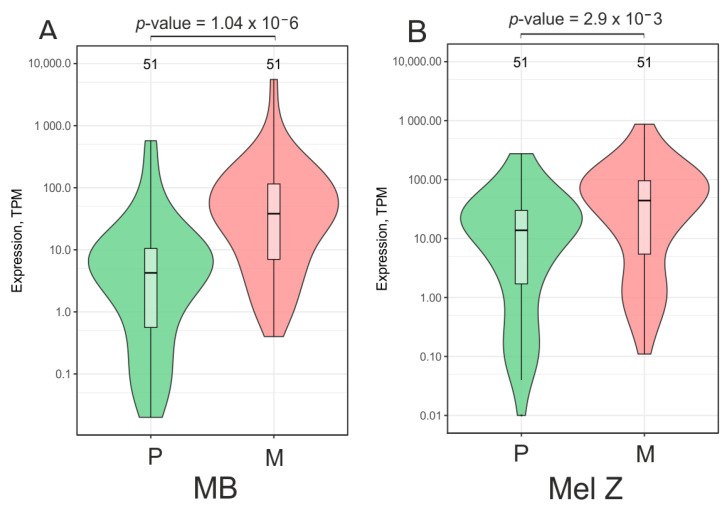
Violin plots showing the expression levels of 51 common upregulated genes in MB (**A**) and Mel Z cells (**B**) cultivated either on plastic (P) or on a 3D matrix (M). TPM—Transcripts Per Million.

Fifty-one genes were upregulated in both cell types when they were cultivated on a 3D matrix. These genes are involved in epithelial cell–cell adhesion, cell population proliferation, and blood vessel development (Table 2). Figure 5 shows that, originally, on plastic, these genes were more active in Mel Z cells than in MB cells. The transfer of MB and Mel Z cells onto a 3D matrix essentially activated this group of genes in both cell types.

### 2.7. BAK1 and SERPINE1 Genes Controlling Blood Vessel Development Quickly Form Frequent Contacts with Nucleoli and Are Activated When Mel Z Cells Are Cultivated on Matrigel

Previous reports indicate that many developmental genes shape frequent inter-chromosomal contacts with nucleoli in human cells of different origins [29,30]. These data were obtained using 4C-rDNA experiments with a ligation-mediated approach [29,30,31,32,33]. It was demonstrated that, after growth on Matrigel and the formation of the VM phenotype, dramatic changes occur in Mel Z cells in rDNA contacts with genomic regions that predominantly encode developmental genes. It was concluded that the inter-chromosomal interactions between developmental genes and rDNA clusters are dynamic, and that nucleoli play an important role in the development of vasculogenic mimicry and stemness phenotypes in aggressive tumor genes [31,32]. Nucleoli were reported to induce either the repression or activation of the expression of different sets of contacting genes [32]. Researchers have inferred that nucleoli induce the formation of numerous phase-separated condensates containing either transcriptional repressors or activators around them [33,34].

**Figure 6:**
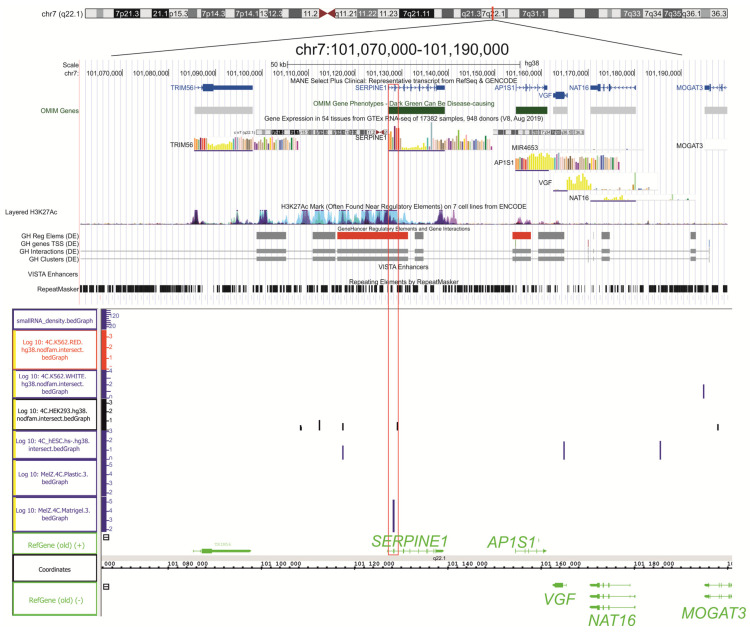
The target of inter-chromosomal contacts of nucleoli genes inside a region of chr7 at the *SERPINE1* gene. Gene expression in 54 tissues, the distribution of layered H3K27ac marks, GeneHancer regulatory elements, and gene interactions are shown in the UCSC Browser (hg38). The 4C-DNA data for K562, HEK293T, and hESM01 cells are also shown [29,31,32,33,35]. The red frame shows the frequent contacts of nucleoli in these genes that were observed only in Mel Z cells cultivated on Matrigel.

We observed VM formation in the two cancer cell lines and nine common upregulated genes (Table 3). Thus, we suggest that these developmental genes, which could be involved in alterations in 3D chromosomal structures and in considerable changes in expression, could be regulated by modulations in their interactions with nucleoli. Data on 4C-rDNA experiments with Mel Z cells cultivated on plastic or Matrigel were previously described [32]. Here, we detected two genes (*BAK1* and *SERPINE1*) that quickly formed frequent inter-chromosomal contacts with rDNA clusters in Mel Z cells cultivated on Matrigel (Figure 6 and Figure S8). No rDNA contacts were detected in the cells cultivated on plastic (Figure 6). These genes are part of the group of nine genes that control blood vessel development and tube morphogenesis (Table 3) and are strongly activated in Mel Z cells upon cultivation on Matrigel [32]. Table S1 shows that these genes are also strongly activated in MB cells after being transferred onto a 3D matrix.

Figure 6 shows the region of numerous inter-chromosomal contacts with nucleoli inside chr7, where the *SERPINE1* gene resides. The gene is expressed in different human vessels (aorta, coronary artery, and tibial artery). The contact region is decorated by the H3K27ac mark and GeneHancer elements. The same is true for *BAK1* gene expression patterns (Figure S8). We conclude that, during VM formation, these genes are substantially activated in both cell lines and form frequent contacts with rDNA clusters, at least in Mel Z cells.

### 2.8. Knockdown of the SERPINE1 Gene Prevents the Formation of the VM Phenotype in Mel Z Cells Growing on Matrigel

To check whether knockdown of any of these nine genes controlling blood vessel development could affect the formation of the VM phenotype, we knocked down the *SERPINE1* gene in Mel Z cells.

**Figure 7:**
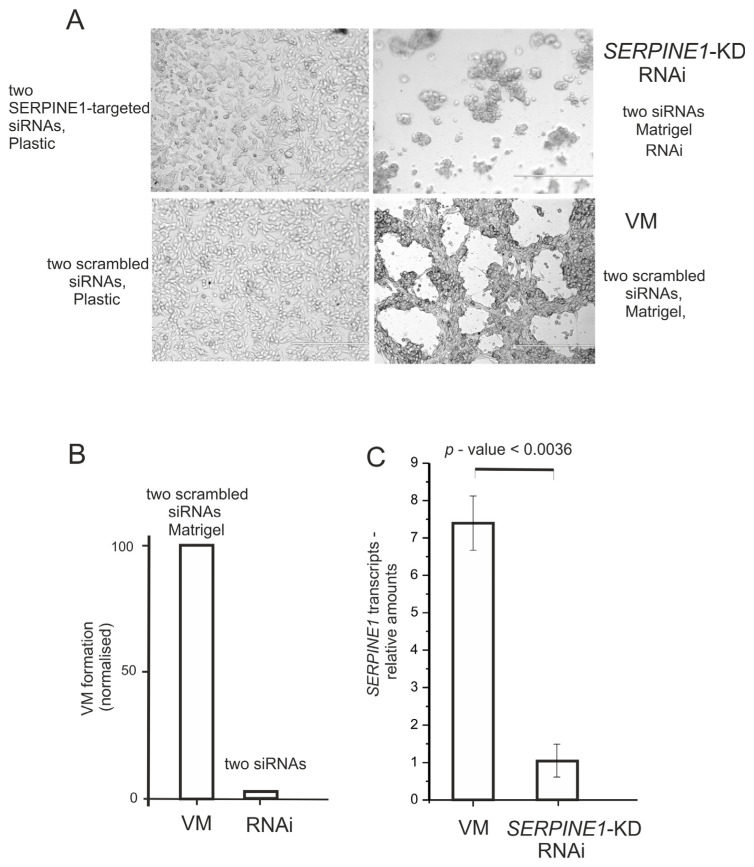
Knockdown of the *SERPINE1* gene prevents the formation of the VM phenotype in Mel Z cells. (**A**) Transfection of Mel Z cells using either two targeting siRNAs or two scrambled siRNAs as a negative control (scale bar = 400 μm). (**B**) Number of branches per well formed after transfection with two *SERPINE1*-specific siRNAs or two scrambled siRNAs, n = 3. (**C**). Targeted RNAi leads to about a seven-times reduction in *SERPINE1* transcripts in transfected cells and to the loss of the capacity of Mel Z cells to form VM.

We found that these cells lost the capacity to form sinuses when seeded on Matrigel (Figure 7). Practically no branches formed between the aggregates of Mel Z cells. At the same time, many sinuses joined by cell branches formed on Matrigel when Mel Z cells were transfected with scrambled siRNAs (control). As expected, transfection with two scrambled siRNAs did not interfere with the formation of sinuses (Figure 7B). The data strongly suggest that the *SERPINE1* gene is involved in the development of the VM phenotype. The results are clearly in agreement with our assumption that the two cell lines containing commonly upregulated genes that are involved in blood vessel development and tube morphogenesis (Table 3) could also be involved in VM formation. Currently, we are testing the effects of the knockdown of some other common genes and plan to conduct a detailed analysis of the subsequent changes in transcriptomes. The results will be published separately.

## 3. Discussion

### 3.1. The Level of Differentiation of Cancer Cells Growing on a 3D Matrix Determines the Changes in Gene Expression

Normal vasculogenesis refers to the development of new vessels from primordial endothelial stem cells. It is known that endothelial cell development requires the activity of endothelial cell-associated genes, including *VEGF*, *SOX7*, *SOX17*, and *SOX18* [36,37]. As expected, these genes were not upregulated in the MB and Mel Z cells forming VM. Nevertheless, these cancer cells can quickly acquire endothelial-like properties and form vessel-like channels or tubes. To identify the genes involved in this transformation in cancer cells, we searched for genes whose expression changed rapidly in MB and Mel Z cells as they grew on a 3D matrix. The idea was to identify the common down- and upregulated genes that could be involved in VM formation.

Previously, we found that about 3000 genes in Mel Z melanoma cells quickly change their expression after being transferred onto Matrigel [13]. Here, we found that an extracellular matrix, collagen, which mimics the cellular microenvironment and enables 3D growth, induces markedly different gene expression patterns across cell types. For example, in Mel Z cells, the most upregulated genes were those involved in ribosome biogenesis, whereas genes involved in development and cell differentiation were downregulated [13]. As a result, the Mel Z cells could easily acquire a less differentiated and more stem-like phenotype.

Here, we show that MB cells behave differently; they upregulated numerous developmental genes while downregulating another set of developmental genes (Figure 1). Although the easily observed reactions of different types of aggressive cancer cells growing on a 3D matrix are very similar—with the formation of incubation sinuses and tubes within a few hours—the underlying molecular mechanisms are quite different. As strong evidence, we consider the fact that the response of cancer cells to a 3D matrix mainly depends on the nature of the cell types and their previous state of differentiation. Based on these data, we assume that the development of primitive sinuses and tubes is controlled by a relatively small set of genes. What we observed in both these cell types is the downregulation of developmental genes. The sets of these genes differ substantially between the two cell types, likely reflecting a previous state of differentiation. We suppose that they finally reach a less differentiated state and share common upregulated genes that control the formation of sinuses and tubes from cancer cells grown on a 3D matrix. We propose that the reduced level of differentiation during cell growth on a 3D matrix enables them to form sinuses and tubes.

The data strongly indicate that the growth of cancer cells of different origins on a 3D matrix induces the activation of a mainly different set of genes. We suppose this is due to differences in the established states of differentiation among these cell types before being transferred onto a 3D matrix. To develop the VM phenotype within a 3D matrix, the cells down- and upregulate distinct sets of genes. It follows that it is common for only a small portion of genes to change expression.

In this study, we observed that knockdown of the *SEPINE1* gene in Mel Z cells prevents the formation of VM (Figure 7). These findings confirm our assumption that even a small group of common genes (even just one gene) could be critical for the development of a set of sinuses and channels. Currently, we are testing the role of other genes (Table 3) also involved in “blood vessel development” using in vitro VM formation tests (the data will be published separately).

Our knowledge is lacking regarding the molecular mechanisms behind the VM phenomenon. Serpine2 and Slpi are among the first validated drivers of this process [38]. These proteins drive the formation of tubules in vitro and extravascular networks in vivo [38]. As inhibitors of different serine proteases, *SERPINE* genes are indirectly involved in different processes, including the degradation of blood clots and the regulation of cell adhesion, cell spreading, and cell migration. The last three processes are directly involved in the formation of VM [38]. They act as regulators of cell migration, independent of their role as protease inhibitors, which is why the KD of *SERPINE* genes prevents the formation of the VM phenotype.

### 3.2. Possible Role of ID1, ID2, and ID3 Genes in Dedifferentiation of Mel Z Cells During Formation of VM

Previously, during the analysis of gene expression changes upon cultivation on Matrigel, we mentioned the strong activation of three Inhibitors of Differentiation or Inhibitors of DNA Binding (ID) genes (ID1, ID, and ID3) in Mel Z cells [13]. These genes can inhibit numerous transcription factors from the HLH family, important regulators of cellular growth and differentiation [39,40,41]. ID proteins lack the basic DNA-binding domain and can dimerize HLH family transcription factors [41]. The resulting ID-HLH heterodimers cannot bind DNA because ID proteins lack the basic DNA-binding domain [42], which is why ID proteins act as dominant-negative antagonists of HLH proteins [43]. Most HLH transcription factors activate developmental genes, while ID genes are considered strong inhibitors of cell differentiation [43].

Interestingly, at the same time, we unexpectedly observed that the opposite event occurs when differentiating K562 cells—the strong concerted downregulation of these three genes [31]. Thus, when discussing the MB cell data, we assume that another set of genes is responsible for their dedifferentiation on Matrigel—by either activating or repressing particular developmental genes. Good candidates for this role are the numerous repressed or activated developmental genes indicated in Tables S2 and S3.

### 3.3. Epigenetic Mechanisms That Are Behind the Downregulation and Upregulation of Large Groups of Genes During the Formation of the VM Phenotype

We observed that most of the genes whose expression changes rapidly during VM formation in Mel Z and MB cells differ. In Mel Z cells, developmental genes are downregulated, and genes involved in ribosomal biogenesis are upregulated [13]. Here, we show that MB cells contain large groups of down- and upregulated genes corresponding to the same GO term (Figure 1). For example, about 200–300 non-overlapping groups of genes that are down- or upregulated are related to “system development”, developmental process”, and “anatomical structure development”.

The changes in observed gene expression were induced by cultivating these two types of cancer cells on a 3D matrix, i.e., these changes emerged epigenetically. Previously, we observed that changes in the expression of large groups of genes in Mel Z cells are tightly linked to numerous lincRNAs, miRNAs, and active and repressive histone marks [13]. In Mel Z cells, both down- and upregulated genes were simultaneously regulated by hundreds of different transcription factors.

Here, we observed that downregulated genes in MB cells were associated with dramatic changes in chromosomal structure, and the major GO driver terms for downregulated genes included “structural constituent of chromatin” and “nucleosome” (Figure S1). A so-called epichromatin that loops at the nuclear periphery has also been described to involve perinucleolar heterochromatin [44,45]. It is likely that some of the downregulated genes in MB cells are transferred to epichromatin regions.

To determine whether genes whose expression changes in MB cells grown on a 3D matrix are co-regulated by hundreds of different transcription factors, we searched for common down- and upregulated genes in these two cell lines. We found 580 transcription factors that simultaneously regulate the 98 common downregulated genes and 795 transcription factors that simultaneously regulate the 51 common upregulated genes (Tables S7 and S8). For example, the *SERPINE1* gene is simultaneously regulated by 422 transcription factors, while *HMOX* is simultaneously regulated by 446 factors. Mechanistically, we propose that numerous micro-drops contain different sets of transcription factors that either repress or activate genes within chromosomal loops that merge into these micro-drops [13].

It has been reported that epigenetic changes in the phenotype of non-cancer cells after cultivation on a 3D matrix could be reversible. Vascular smooth muscle cells cultured on Matrigel could be completely reversed through at least five passages after replating them on uncoated plastic dishes [46], while fetal human type II cells are only partially reversible after this procedure [47]. Currently, we are studying whether Mel Z cells could be reversed to their initial phenotype and gene expression patterns after several passages.

Here, we observed that at least two out of nine common genes controlling blood vessel development and tube morphogenesis (*SERPINE1* and *BAK1*) shape frequent inter-chromosomal contacts with nucleoli after transferring Mel Z cells onto Matrigel (Figure 7 and Figures S8). Different lines of evidence indicate that nucleoli are involved in the dynamic regulation of developmental genes [7,29,30,31,32,33,34,48,49,50]. A large proportion of rDNA-contacting genes in Mel Z cells growing on Matrigel have been associated with downregulation [13]. Here, we observed that these contacts activate *SERPINE1* and *BAK1* genes. Currently, we are studying the nature of histone marks at these contacts for Mel Z cells growing on Matrigel.

## 4. Materials and Methods

### 4.1. Mel Z Cell Culture

The cells were obtained from the N.N. Blokhin National Medical Research Center at the Oncology Department of the Ministry of Health, Russia [13], and were initially propagated on a plastic surface in an RPMI-1640 medium supplemented with 10% fetal calf serum, 2 mM glutamine, and 0.1% gentamicin sulfate at 37 °C in a humidified atmosphere containing 5% CO_2_. At 70–75% confluency, the cells were divided into two equal samples and seeded either on plastic or Matrigel. After cultivation for 15 h, the medium above the cells was removed, and then the cells were liberated by incubation for 20 min at 37 °C in 5 mL of Versene solution in Dulbecco’s phosphate-buffered saline containing 0.05% trypsin. The cells were then used for 4C experiments and RNA isolation. Petri dishes coated with Matrigel (BD Bioscience, Bedford, MA, USA) were prepared as follows: the Matrigel (8.7 mg/mL) was thawed at 4 °C, and 6 mL of Matrigel was quickly added to each 10 cm dish and allowed to solidify for 30 min at room temperature; then, it was placed in a humidified 5% CO_2_ incubator for 1 h at 37 °C. The RNA-seq and 4C-rDNA analysis process for cells grown on plastic and Matrigel was previously described in [13,32].

### 4.2. MDA-MB-231 RNA-Seq Data

The raw RNA-seq datasets of human breast cancer MDA-MB-231 cells were retrieved from the NCBI Gene Expression Omnibus (GEO) repository. The accession numbers were as follows: cells cultured on plastic (GSM1944521, GSM1944522, and GSM1944523) and cells cultured on a 3D collagen matrix (GSM2700358, GSM2700359, and GSM2700360). Cell cultivation and RNA-seq procedures are described in each relevant GEO accession. The number of raw SE reads per replicate for MDA-MB-231 cells grown on plastic were as follows: 21,603,980; 28,531,869; and 29,758,765. The number of raw PE reads per replicate for MDA-MB-231 cells grown on a 3D matrix were as follows: 9,999,972, 10,023,414, and 12,121,191.

### 4.3. RNA-Seq Data Processing

All RNA-seq datasets were processed using a uniform analysis pipeline. To ensure consistency between the reference genome sequence and gene annotation, the same genome build was used for alignment and annotation (Homo sapiens hg38/GRCh38, Ensembl release 112; available online: https://ftp.ensembl.org/pub/release-112/, accessed on 14 February 2026).

Raw sequencing reads were preprocessed using Trimmomatic v0.39 [51]. Reads shorter than 20 bp were discarded, and low-quality bases (Q < 18) were removed from both ends of the reads. Read quality was further controlled using a sliding window approach (SLIDINGWINDOW: 4:22). The following parameters were applied: LEADING: 18; TRAILING: 18; SLIDINGWINDOW: 4:22; and MINLEN: 20. For SE libraries, Illumina adapter sequences were removed using ILLUMINACLIP:TruSeq3-SE.fa:2:30:10. For PE libraries, adapter trimming was performed using ILLUMINACLIP:TruSeq3-PE-2.fa:2:30:10:2.

Trimmed reads from each replicate were quantified against the *H. sapiens* Ensembl release 112 reference genome and annotation using RSEM v1.3.1 [52]. The following parameters were applied: --star -p 16 --calc-ci --ci-memory 60720. Gene- and transcript-level expression estimates obtained for biological replicates were averaged using an in-house R 4.2.2 script.

To ensure consistency with RSEM-derived alignments, trimmed reads were independently aligned to the same reference genome using STAR aligner v2.7.11b [53] with parameters consistent with those implemented internally by RSEM (--outSAMunmapped Within --outFilterType BySJout --outSAMattributes NH HI AS NM MD --outFilterMultimapNmax 20 --outFilterMismatchNmax 999 --outFilterMismatchNoverLmax 0.04 --alignIntronMin 20 --alignIntronMax 1000000 --alignMatesGapMax 1000000 --alignSJoverhangMin 8 --alignSJDBoverhangMin 1 --sjdbScore 1 --quantMode TranscriptomeSAM). Aligned reads generated with STAR were assigned to genomic features using featureCounts v2.0.6 [54]. The recommended parameters were applied as follows: -t exon -g gene_id for SE libraries, and -p --countReadPairs -t exon -g gene_id for PE libraries.

Reproducibility between biological replicates was assessed using deepTools v.3.5.5 [55]. Pearson’s (*r*) and Spearman’s (*ρ*) correlation coefficients indicated a high degree of consistency for Mel Z cells cultured on plastic (*r* = 0.92, *ρ* = 0.79) and on Matrigel substrate (*r* = 0.92, *ρ* = 0.79), supporting the reliability of the corresponding RNA-seq datasets.

In contrast, the consistency analysis of RNA-seq data obtained for MDA-MB-231 cells cultured on the 3D matrix substrate, performed using deepTools [55] and pcaExplorer v.2.24.0 [56], revealed that one of the three replicates (GSM2700358/SRR5817998) displayed insufficient concordance with the other two replicates. Therefore, this dataset was excluded from subsequent analyses.

The following conclusions can be drawn from the consistency analysis of the MDA-MB-231 datasets: Pearson’s (r) and Spearman’s (*ρ*) correlation coefficients indicate a strong concordance between three datasets representing RNASeq expression data obtained from MDA-MB-231 cells cultured on plastic (*r* = 0.86, 0.73, 0.65; *ρ* = 0.84 for all three comparisons) and a high degree of concordance (*r* = 0.92 and *ρ* = 0.92) for the cells grown on the 3D matrix substrate. Scatterplots illustrating concordance between biological replicates for all datasets employed in the manuscript are available in Figure S9.

Differential gene expression analysis was performed using the DESeq2 v1.38.3 [18] R library. Differential expression tables were generated for the comparisons of Mel Z plastic versus Matrigel and MDA-MB-231 plastic versus 3D matrix. DESeq2 was also employed to generate scatterplots of quantified datasets for consistency assessment between biological replicates (Figure S9). Gene identifiers were converted to ISO gene names using an in-house R script to facilitate subsequent genetic and Gene Ontology analyses. Figure 1A was generated using an R script based on the EnhancedVolcano v1.16.0 [56,57] library.

Intersections between gene lists obtained from the Mel Z plastic versus Matrigel and MDA-MB-231 plastic versus 3D matrix comparisons were identified. This analysis revealed 98 commonly downregulated genes (Figure 2; Table 1) and 51 commonly upregulated genes (Figure 3; Table 2). Violin plots for these gene sets (Figure 3 and Figure 4) were generated from TMM-normalized expression data using R scripts based on dplyr v.1.1.4 [58] and ggplot2 v.3.5.0 [59] R libraries.

Statistical differences between subsets of 4C-rDNA-associated genes were assessed using the nonparametric Mann–Whitney U test for independent samples. Resulting *p*-values were adjusted for multiple comparisons using the Benjamini–Hochberg FDR correction [60] implemented in the pairwise.wilcox.test() standard function in R. The analysis confirmed that, in each pairwise comparison, the subsets were drawn from different distributions, indicating statistical independence. The statistical significance of gene list intersections was assessed using the hypergeometric test implemented through the dhyper() function in R.

### 4.4. SERPINE1 Knockdown Using siRNAs

In this experiment, *SERPINE1*-targeting siRNAs rArUrUrArGrArUrUrArCrArUrUrCrArUrUrUrCrArC and rArArUrUrGrUrArUrGrGrUrCrArArUrUrUrCrCrArU were used, plus as well as nontargeting scrambled siRNAs (negative controls).

siRNAs were selected using siDirect software, version 2.1 (https://sidirect2.rnai.jp/, accessed on 17 April 2026). Transfections were performed using Lipofectamine 3000 (Invitrogen by Thermo Fisher Scientific, Waltham, MA, USA) according to the manufacturer’s instructions, with knockdown maximized at 10 nM. Then, equal amounts of transfected cells (320 × 10^3^ cells/well) were seeded either on plastic or Matrigel and incubated for 8 h on average. They were then photographed using an EVOS XL Core Imaging System. Viability of Lipofectamine 3000-transfected cells remained above 80–90% compared to untransfected controls.

For RT-PCR experiments, RNA samples were isolated by TRIzol (Invitrogen, Carlsbad, CA, USA) from about 320 × 10^3^ transfected Mel Z cells. The DNA-free RNA samples were prepared using a TURBO DNA-free Ambion Kit. Primer extensions and amplifications were performed using a High-Capacity Reverse Transcription Kit (Applied Biosystems, Waltham, MA, USA) and Taq-Polymerase (SibEnsyme, Novosibirsk, Russia). Oligonucleotide 5′ TTTTTCAGTGGAGAACATGG 3′ was used for the primer extension, and oligonucleotides 5′ GGGCTGCATGACCTACCAGG 3′ and 5′ TTGTGCCCTACCCTCTGGCT 3′ were used as forward and reverse primers, respectively.

## Figures and Tables

**Table 1:** GO associations with biological processes of 98 downregulated genes common to MB and Mel Z cells forming the VM phenotype.

GO.ID	Description	padj	Genes
GO:0007156	homophilic cell adhesion via plasma membrane adhesion molecules	0.00275	*PCDHGA5*, *PCDHGA4*, *PCDHGB7*, *PCDHGA11*, *PCDHGB3*, *PCDHGA2*, *PCDHGA6*
GO:0042127	regulation of cell population proliferation	0.003406	*KIF14*, *MEGF10*, *ESR1*, *VASH2*, *SPRY1*, *PTPN6*, *PTPRJ*, *DLC1*, *ST6GAL1*, *ERBB4*, *ALDH1A2*, *PDE9A*, *ST8SIA1*, *CLMN*, *ASPM*, *LAMB1*, *NR5A2*, *AR*, *P3H3*
GO:0008283	cell population proliferation	0.01202	*KIF14*, *MEGF10*, *ESR1*, *GNG2*, *VASH2*, *SPRY1*, *PTPN6*, *PTPRJ*, *DLC1*, *ST6GAL1*, *ERBB4*, *ALDH1A2*, *PDE9A*, *ST8SIA1*, *CLMN*, *ASPM*, *LAMB1*, *NR5A2*, *AR*, *P3H3*
GO:0007399	nervous system development	0.014038	*KIF14*, *PCDHGA5*, *PCDHGA4*, *VASH2*, *PCDHGB7*, *PCDHGA11*, *PTPRJ*, *DLC1*, *PCDHGB3*, *ETV1*, *ERBB4*, *SYNE2*, *ALDH1A2*, *CLMN*, *SLITRK6*, *PCDHGA2*, *ASPM*, *DDIT4*, *LAMB1*, *SEMA3D*, *PRICKLE1*, *PCDHGA6*, *ARHGEF10*
GO:0048731	system development	0.03740	*KIF14*, *SLC2A10*, *PCDHGA5*, *ESR1*, *PCDHGA4*, *VASH2*, *PCDHGB7*, *SPRY1*, *PTPN6*, *PCDHGA11*, *PTPRJ*, *DLC1*, *PCDHGB3*, *ETV1*, *ERBB4*, *SYNE2*, *ALDH1A2*, *MAP2K6*, *CLMN*, *SLITRK6*, *PCDHGA2*, *ASPM*, *DDIT4*, *LAMB1*, *SEMA3D*, *PRICKLE1*, *PCDHGA6*, *AR*, *ARHGEF10*
GO:0032502	developmental process	0.045702	*KIF14*, *MEGF10*, *SLC2A10*, *PCDHGA5*, *ESR1*, *PCDHGA4*, *NNMT*, *VASH2*, *PCDHGB7*, *SPRY1*, *PTPN6*, *PCDHGA11*, *PTPRJ*, *DLC1*, *PCDHGB3*, *JDP2*, *ETV1*, *ERBB4*, *SYNE2*, *FAM20A*, *ALDH1A2*, *MAP2K6*, *ADAMTS20*, *CLMN*, *SLITRK6*, *PCDHGA2*, *ASPM*, *DDIT4*, *LAMB1*, *EXPH5*, *NR5A2*, *RBM47*, *SEMA3D*, *SLFN5*, *PRICKLE1*, *HIP1*, *PCDHGA6*, *AR*, *ARHGEF10*

**Table 2:** GO associations with biological processes (BP) and cellular components (CC) of 51 upregulated genes common for MB and Mel Z cells forming the VM phenotype.

GO.ID	Description	padj	Genes
**BP**			
GO:0090136	epithelial cell–cell adhesion	0.01124	*SERPINB8*, *PLEKHA7*, *CCN3*
GO:0042127	regulation of cell population proliferation	0.01523	*PDCL3*, *BAK1*, *FAM98B*, *NGFR*, *HMOX1*, *TAX1BP3*, *CDKN1A*, *ADM*, *S1PR3*, *SPHK1*, *ATF5*, *SF1*, *CORO1A*, *CCN3*
GO:0008283	cell population proliferation	0.02494	*BYSL*, *PDCL3*, *BAK1*, *FAM98B*, *NGFR*, *HMOX1*, *TAX1BP3*, *CDKN1A*, *ADM*, *S1PR3*, *SPHK1*, *ATF5*, *SF1*, *CORO1A*, *CCN3*
GO:0008285	negative regulation of cell population proliferation	0.03215	*BAK1*, *NGFR*, *HMOX1*, *TAX1BP3*, *CDKN1A*, *ADM*, *ATF5*, *SF1*, *CCN3*
GO:0001568	blood vessel development	0.04276	*PDCL3*, *BAK1*, *NGFR*, *HMOX1*, *ADM*, *SPHK1*, *PDE2A*, *SERPINE1*, *CCN3*
**CC**			
GO:0005654	nucleoplasm	0.00153	*BYSL*, *PDCL3*, *FAM98B*, *NGFR*, *PSMD2*, *HMOX1*, *NUDC*, *POLR2A*, *CDKN1A*, *TTC4*, *PRCC*, *MRNIP*, *PRPF19*, *LSM10*, *SPHK1*, *CCDC86*, *PLEKHA7*, *ATF5*, *NXF1*, *GAR1*, *SF1*, *GEMIN7*, *SF3B4*
GO:0031981	nuclear lumen	0.03967	*BYSL*, *PDCL3*, *FAM98B*, *NGFR*, *PSMD2*, *HMOX1*, *TAX1BP3*, *NUDC*, *POLR2A*, *CDKN1A*, *TTC4*, *PRCC*, *MRNIP*, *PRPF19*, *LSM10*, *SPHK1*, *CCDC86*, *PLEKHA7*, *ATF5*, *NXF1*, *GAR1*, *SF1*, *GEMIN7*, *SF3B4*

**Table 3:** GO associations with biological processes of nine highly upregulated genes common to MB and Mel Z cells, forming the VM phenotype.

GO.ID	Description	padj	Genes
GO:0001568	blood vessel development	7.95882699585203 × 10^−11^	*PDCL3*, *BAK1*, *NGFR*, *HMOX1*, *ADM*, *SPHK1*, *PDE2A*, *SERPINE1*, *CCN3*
GO:0001944	vasculature development	1.1446992774816197 × 10^−10^	*PDCL3*, *BAK1*, *NGFR*, *HMOX1*, *ADM*, *SPHK1*, *PDE2A*, *SERPINE1*, *CCN3*
GO:0072359	circulatory system development	4.542051208666478 × 10^−9^	*PDCL3*, *BAK1*, *NGFR*, *HMOX1*, *ADM*, *SPHK1*, *PDE2A*, *SERPINE1*, *CCN3*
GO:0048514	blood vessel morphogenesis	6.453829580008994 × 10^−9^	*PDCL3*, *BAK1*, *NGFR*, *HMOX1*, *ADM*, *SPHK1*, *SERPINE1*, *CCN3*
GO:0035239	tube morphogenesis	8.714904047216382 × 10^−8^	*PDCL3*, *BAK1*, *NGFR*, *HMOX1*, *ADM*, *SPHK1*, *SERPINE1*, *CCN3*
GO:0001525	angiogenesis	2.969092680768523 × 10^−7^	*PDCL3*, *NGFR*, *HMOX1*, *ADM*, *SPHK1*, *SERPINE1*, *CCN3*
GO:0035295	tube development	5.262592688842451 × 10^−7^	*PDCL3*, *BAK1*, *NGFR*, *HMOX1*, *ADM*, *SPHK1*, *SERPINE1*, *CCN3*
GO:0045766	positive regulation of angiogenesis	0.000003940661311141707	*PDCL3*, *HMOX1*, *ADM*, *SPHK1*, *SERPINE1*
GO:1904018	positive regulation of vasculature development	0.0000043124816928016285	*PDCL3*, *HMOX1*, *ADM*, *SPHK1*, *SERPINE1*
GO:0045765	regulation of angiogenesis	0.0000679284344718263	*PDCL3*, *HMOX1*, *ADM*, *SPHK1*, *SERPINE1*

## Data Availability

4C-rDNA data for Mel Z cells were deposited in the Gene Expression Omnibus (GEO) repository under accession number GSE295545 and RNA-Seq data for Mel Z cells were deposited in the GEO repository under accession numbers GSE221876 and GSE221872.

## Supplementary Materials

The following supporting information can be downloaded at https://www.mdpi.com/article/10.3390/ijms27114952/s1.
