# Supplementary material for: Highly Aggressive and Metastatic MDA-MB-231 and Mel Z Cancer Cells Have Common Sets of Down- and Upregulated Genes During Formation of the Vasculogenic Mimicry Phenotype
Source: Int J Mol Sci. 2026 May 29;27(11):4952. doi: 10.3390/ijms27114952 (PMC13256147; doi:10.3390/ijms27114952)
Supplement: Supplementary file 1 [file ijms-27-04952-s001.zip › Supplemental Information-May-2026.pdf]

-**Supplemental Information for:**

Highly aggressive and metastatic MDA-MB-231 and Mel Z cancer cells have common sets of downregulated and upregulated genes during formation of vasculogenic mimicry phenotype

Nickolai A. Tchurikov, [1*] Elena S. Klushevskaya [1], Viktoriya N. Lukicheva [1], Antonina N. Kretova [1], Vladimir R. Chechetkin [1], Galina I. Kravatskaya [1], Amalia A. Vartanian [2], Ildar R. Alembekov [1], and Yuri V. Kravatsky [1]

[1] Department of Epigenetic Mechanisms of Gene Expression Regulation, Engelhardt Institute of Molecular Biology Russian Academy of Sciences, Moscow, 119334, Russia
[2] Department of Experimental Diagnosis and Therapy of Tumors, N.N. Blokhin National Medical Research Center of Oncology of the Ministry of Health of Russia, Moscow 115478, Russia
*Correspondence: tchurikov@eimb.ru

This PDF file includes:

Figures S1-9

Tables S1–S8

Figures S1-9

ID	Source	Term ID	Term Name	p_{adj} (query_1)
1	GO:MF	GO:0030527	structural constituent of chromatin	4.561×10^{-11}
2	GO:MF	GO:0005102	signaling receptor binding	2.456×10^{-7}
3	GO:MF	GO:0005509	calcium ion binding	1.014×10^{-5}
4	GO:MF	GO:0046982	protein heterodimerization activity	1.251×10^{-3}
5	GO:MF	GO:0023026	MHC class II protein complex binding	8.127×10^{-3}
6	GO:MF	GO:0030627	pre-mRNA 5'-splice site binding	1.089×10^{-2}
7	GO:MF	GO:0031492	nucleosomal DNA binding	1.244×10^{-2}
8	GO:BP	GO:0007155	cell adhesion	3.724×10^{-11}
9	GO:BP	GO:0048731	system development	3.596×10^{-7}
10	GO:BP	GO:0002399	MHC class II protein complex assembly	2.382×10^{-4}
11	GO:BP	GO:0042129	regulation of T cell proliferation	3.813×10^{-4}
12	GO:BP	GO:0023052	signaling	8.388×10^{-4}
13	GO:BP	GO:0016477	cell migration	1.376×10^{-3}
14	GO:BP	GO:0141124	intracellular signaling cassette	4.675×10^{-3}
15	GO:BP	GO:0006334	nucleosome assembly	1.189×10^{-2}
16	GO:BP	GO:0051050	positive regulation of transport	1.198×10^{-2}
17	GO:BP	GO:0002684	positive regulation of immune system process	2.543×10^{-2}
18	GO:BP	GO:0006959	humoral immune response	2.722×10^{-2}
19	GO:BP	GO:0045937	positive regulation of phosphate metabolic process	3.734×10^{-2}
20	GO:CC	GO:0071944	cell periphery	1.444×10^{-13}
21	GO:CC	GO:0000786	nucleosome	4.520×10^{-8}
22	GO:CC	GO:0042613	MHC class II protein complex	3.864×10^{-4}
23	GO:CC	GO:0005685	U1 snRNP	2.656×10^{-3}

Figure S1. Gene Ontology driver terms for 1511 downregulated genes. The search was performed using Gene Ontology driver terms (https://biit.cs.ut.ee/gprofiler). Related to Figure 1B.

ID	Source	Term ID	Term Name	p_{adj} (query_1)
1	GO:MF	GO:0005515	protein binding	2.377×10^{-8}
2	GO:MF	GO:0003729	mRNA binding	4.981×10^{-4}
3	GO:MF	GO:0009055	electron transfer activity	3.139×10^{-3}
4	GO:MF	GO:0015453	oxidoreduction-driven active transmembrane trans...	5.738×10^{-3}
5	GO:MF	GO:0015078	proton transmembrane transporter activity	8.212×10^{-3}
6	GO:MF	GO:0022853	active monoatomic ion transmembrane transporte...	2.104×10^{-2}
7	GO:BP	GO:0032501	multicellular organismal process	2.781×10^{-8}
8	GO:BP	GO:0048522	positive regulation of cellular process	2.322×10^{-7}
9	GO:BP	GO:0050896	response to stimulus	2.920×10^{-4}
10	GO:BP	GO:0051384	response to glucocorticoid	3.821×10^{-4}
11	GO:BP	GO:1902600	proton transmembrane transport	7.422×10^{-4}
12	GO:BP	GO:0042542	response to hydrogen peroxide	3.145×10^{-3}
13	GO:BP	GO:0009410	response to xenobiotic stimulus	3.927×10^{-3}
14	GO:BP	GO:0016477	cell migration	1.057×10^{-2}
15	GO:BP	GO:0045471	response to ethanol	2.216×10^{-2}
16	GO:BP	GO:0006810	transport	3.433×10^{-2}
17	GO:BP	GO:0019646	aerobic electron transport chain	4.009×10^{-2}
18	GO:CC	GO:0030054	cell junction	3.945×10^{-6}
19	GO:CC	GO:0005654	nucleoplasm	2.898×10^{-5}
20	GO:CC	GO:1902495	transmembrane transporter complex	1.787×10^{-4}
21	GO:CC	GO:0098803	respiratory chain complex	5.129×10^{-3}
22	GO:CC	GO:0099572	postsynaptic specialization	5.205×10^{-3}
23	GO:CC	GO:0005737	cytoplasm	6.607×10^{-3}
24	GO:CC	GO:0031252	cell leading edge	1.118×10^{-2}
25	GO:CC	GO:0070382	exocytic vesicle	1.353×10^{-2}
26	GO:CC	GO:0005615	extracellular space	4.093×10^{-2}

Figure S2. Gene Ontology driver terms for 1197 upregulated genes detected in MB cells cultivated on 3D matrix. The search was performed using Gene Ontology driver terms (https://biit.cs.ut.ee/gprofiler). Related to Figure 1C.

ID	Source	Term ID	Term Name	p_{adj} (query_1)
1	GO:MF	GO:0036094	small molecule binding	9.705×10^{-3}
2	GO:BP	GO:0007156	homophilic cell adhesion via plasma membrane ad...	2.752×10^{-3}
3	GO:BP	GO:0042127	regulation of cell population proliferation	3.403×10^{-3}
4	GO:BP	GO:0007399	nervous system development	1.404×10^{-2}
5	GO:BP	GO:0032502	developmental process	4.570×10^{-2}

Figure S3. Gene Ontology driver terms for 98 common downregulated genes detected in MB and Mel Z cells cultivated on 3D matrix. The search was performed using Gene Ontology driver terms (https://biit.cs.ut.ee/gprofiler). Related to Figure 2.

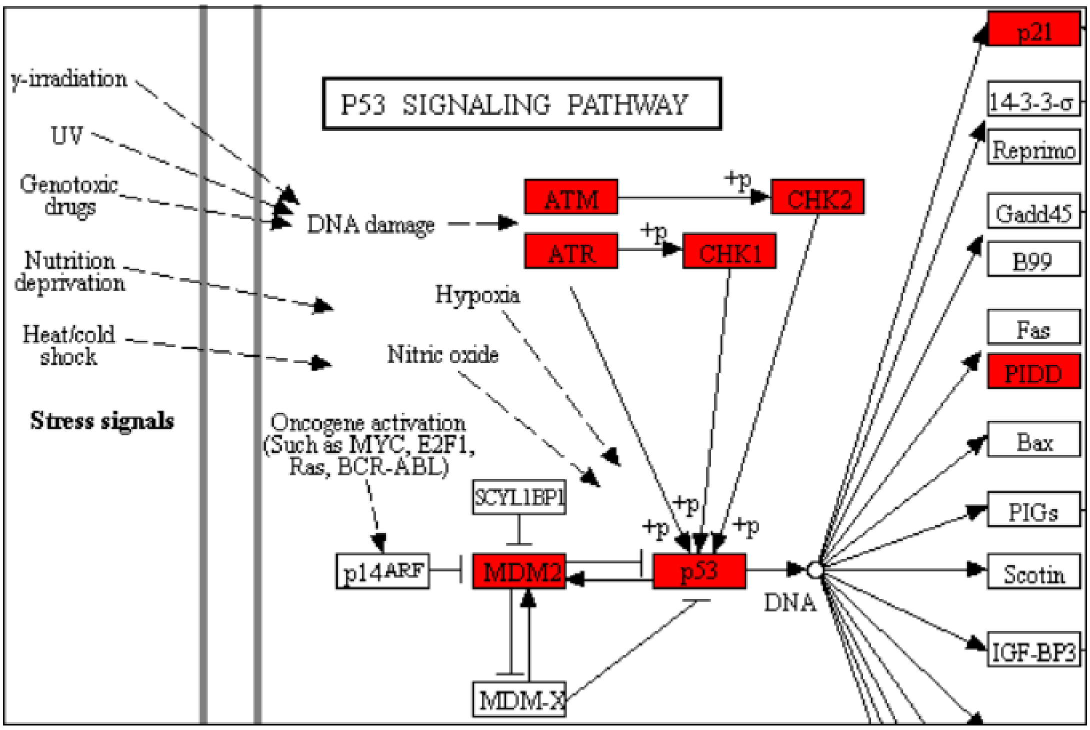

GO Enrichment analysis, plus a lot more!

Just paste your gene list to get enriched GO terms and othe pathways for over 14,000 species. based on annotation from Ensembl and STRING-db. Produce KEGG pathway diagrams with your genes highlighted, hierarchical clustering trees and networks summarizing overlapping terms/pathways, protein-protein interaction networks, gene characterristics plots, and enriched promoter motifs.

Enrichment FDR	Genes in list	Total genes	Functional Category
6.5E-220	86	101	DNA damage checkpoint
5.3E-215	86	108	DNA integrity checkpoint
3.2E-188	86	169	Cell cycle checkpoint
1.1E-131	87	659	Cellular response to DNA damage stimulus
1.2E-113	87	1039	Cell cycle process
3.6E-102	87	1395	Cell cycle
1.1E-100	45	57	Mitotic DNA integrity checkpoint
5.0E-99	87	1517	Cellular response to stress
1.9E-98	43	51	Mitotic DNA damage checkpoint

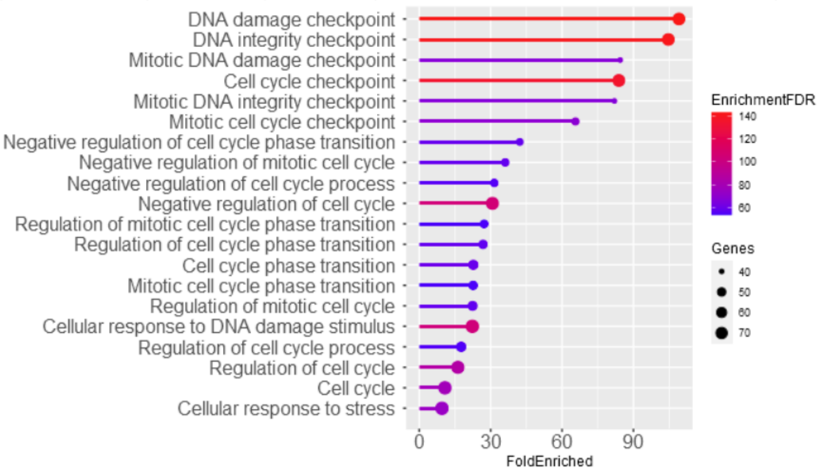

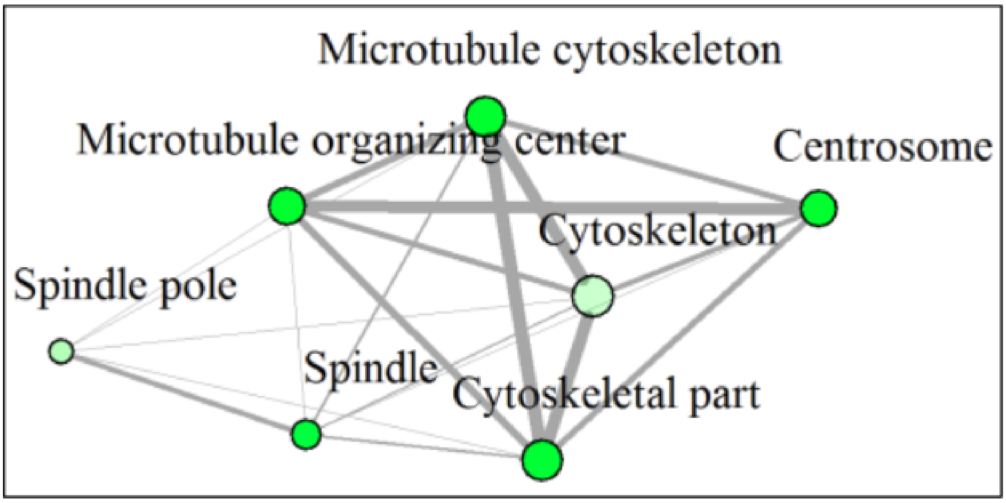

Figure S4. KEGG pathways and enrichment analysis associated with 98 downregulated genes common for MB and Mel Z cells grown on 3D matrix. The search was performed using GO search in **ShinyGO 0.80** (https://bioinformatics.sdstate.edu/go80/).

ID	Source	Term ID	Term Name	p_{adj} (query_1)
1	GO:MF	GO:0005515	protein binding	1.145×10^{-2}
2	GO:BP	GO:0090136	epithelial cell-cell adhesion	1.125×10^{-2}
3	GO:BP	GO:0042127	regulation of cell population proliferation	1.524×10^{-2}
4	GO:BP	GO:0001568	blood vessel development	4.277×10^{-2}
5	GO:CC	GO:0005654	nucleoplasm	1.534×10^{-3}

Figure S5. Gene Ontology driver terms for 51 upregulated genes. The search was performed using Gene Ontology driver terms (https://biit.cs.ut.ee/gprofiler). Related to Figure 3.

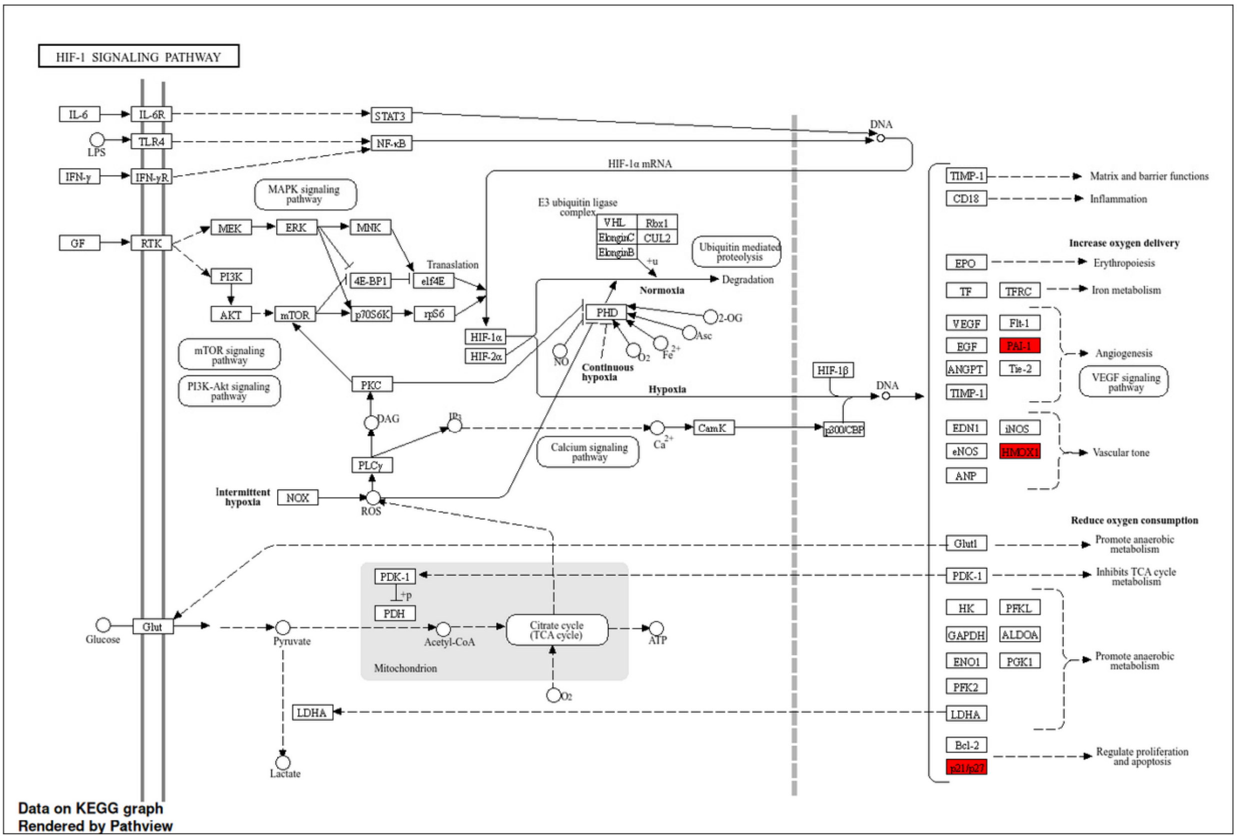

Your genes are highlighted in red. Downloading pathway diagram from KEGG can take 3 minutes.

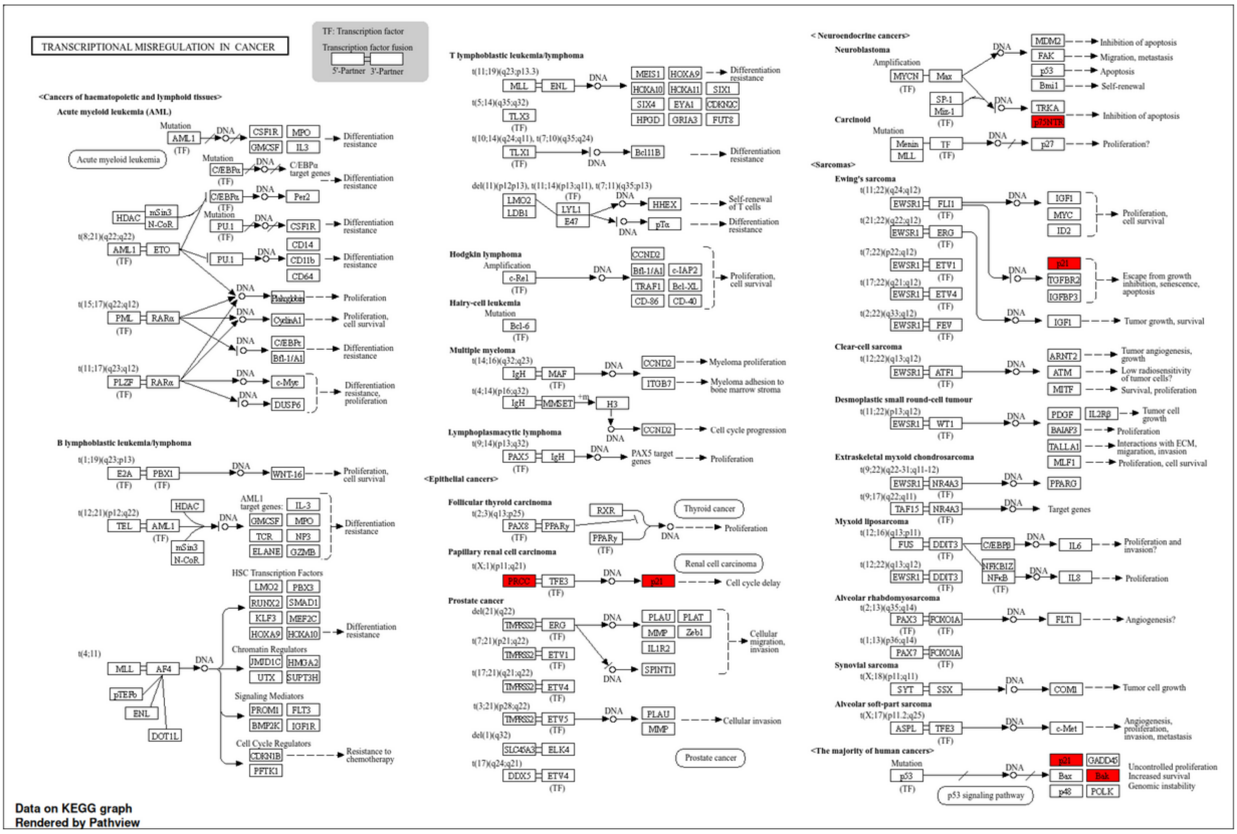

Your genes are highlighted in red. Downloading pathway diagram from KEGG can take 3 minutes.

Figure S6. KEGG pathways associated with 51 upregulated genes. The search was performed using GO search in **ShinyGO 0.80** (https://bioinformatics.sdstate.edu/go80/).

A

ID	Source	Term ID	Term Name	p_{adj} (query_1)
1	GO:BP	GO:0001568	blood vessel development	7.959×10^{-11}
2	GO:BP	GO:0006954	inflammatory response	3.918×10^{-4}
3	GO:BP	GO:1901700	response to oxygen-containing compound	6.803×10^{-4}
4	GO:BP	GO:0042475	odontogenesis of dentin-containing tooth	8.177×10^{-3}
5	GO:BP	GO:0009605	response to external stimulus	8.476×10^{-3}
6	GO:BP	GO:0042542	response to hydrogen peroxide	8.980×10^{-3}
7	GO:BP	GO:0043116	negative regulation of vascular permeability	3.083×10^{-2}
8	GO:BP	GO:0035556	intracellular signal transduction	3.391×10^{-2}
9	GO:CC	GO:0005741	mitochondrial outer membrane	1.047×10^{-2}

B

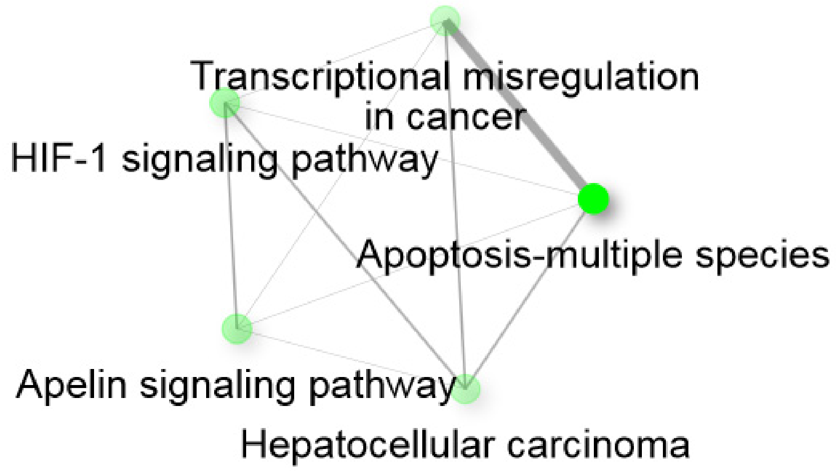

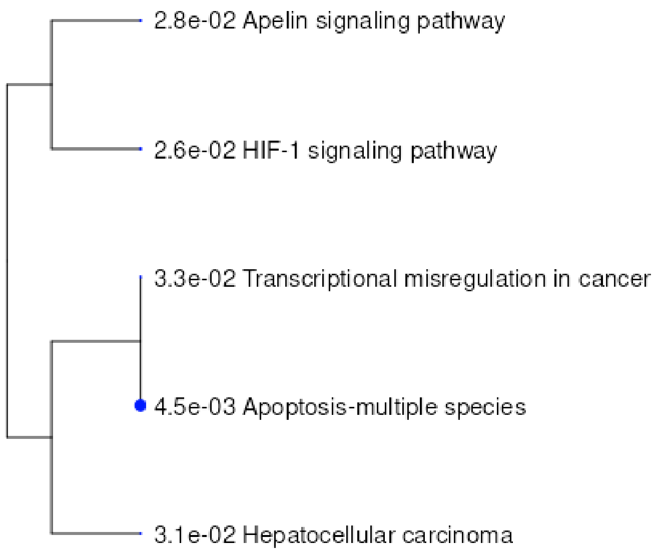

Figure S7. A-Gene Ontology driver terms for commom 9 upregulated genes controlling blood vessel development (*PDCL3,BAK1,NGFR,HMOX1,ADM,SPHK1,PDE2A,SERPINE1,CCN3*) in MB and Mel Z cells growing on 3D matrix**.** The search was performed using Gene Ontology driver terms (https://biit.cs.ut.ee/gprofiler).

B-KEGG pathways associated with the commom 9 upregulated genes controlling blood vessel development (*PDCL3,BAK1,NGFR,HMOX1,ADM,SPHK1,PDE2A,SERPINE1,CCN3*) in MB and Mel Z cells. The search was performed using GO search in **ShinyGO 0.80** (https://bioinformatics.sdstate.edu/go80/).

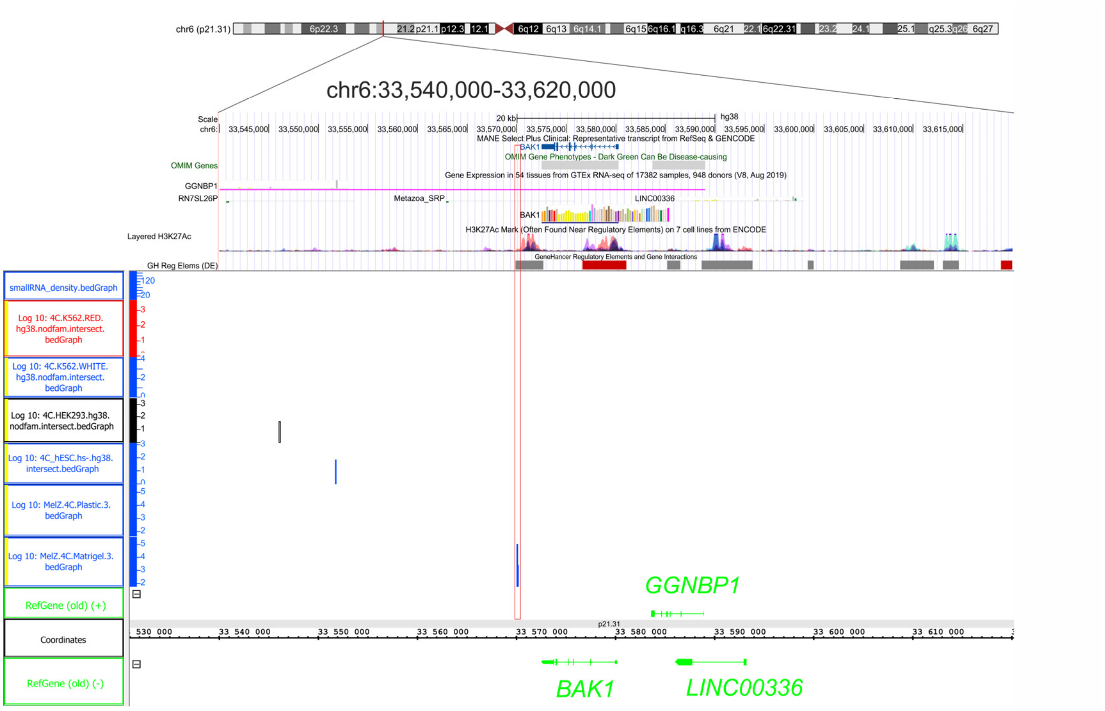

Figure S8. The target of inter-chromosomal contacts of nucleoli gene inside a region of chr6 at *BAK1* gene. The gene expression in 54 tissues, distribution of layered H3K27ac marks, GeneHancer regulatory elements and gene interactions are shown as in the UCSC Browser (hg38), as well the 4C-DNA data for K562, HEK293T, and for hESM01 cells are shown [29,31-33,35]. In the red frame the frequent contacts of nucleoli in this genes that were observe only in Mel Z cells cultivated on Matrigel are shown.

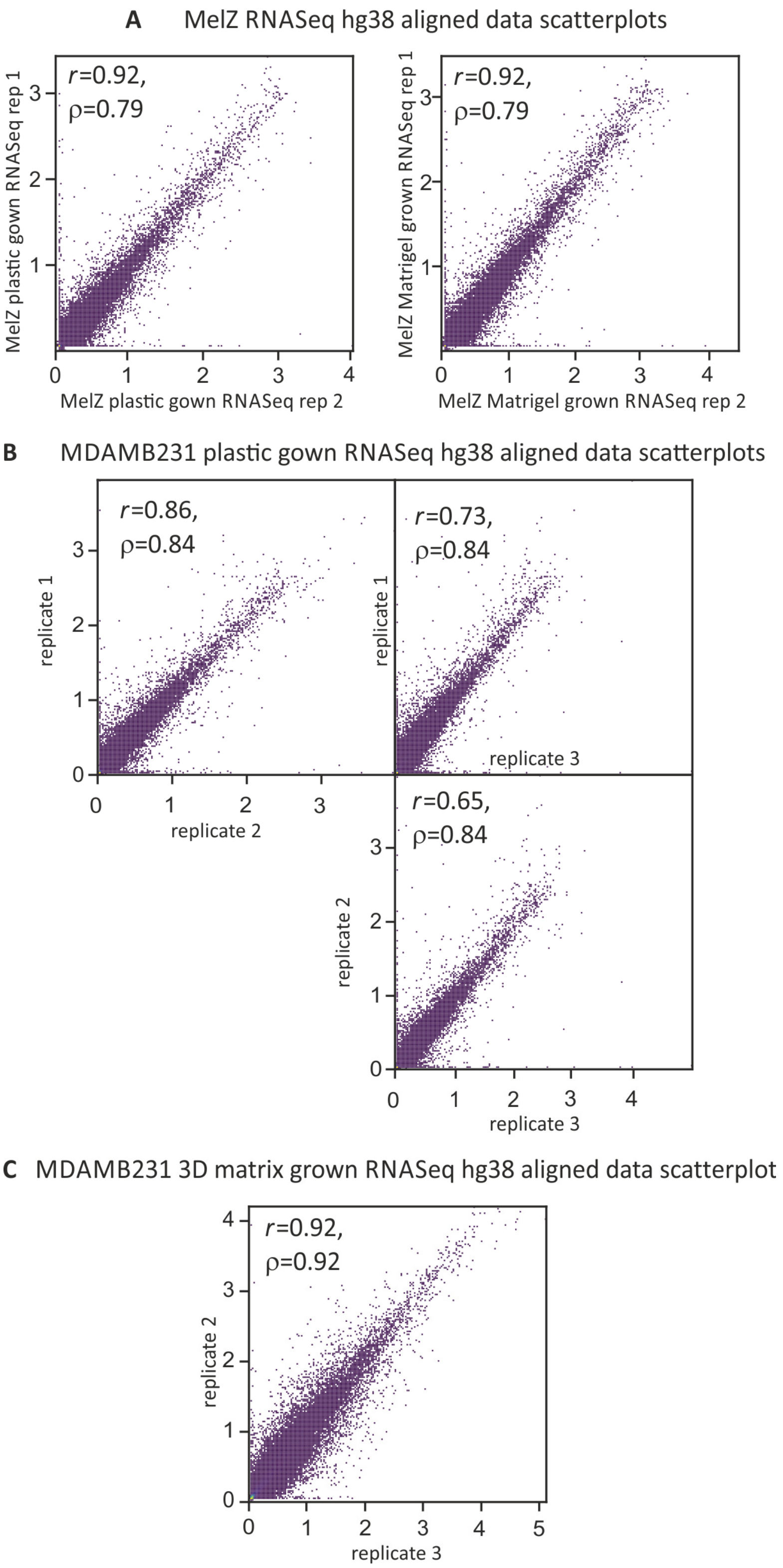

Figure S9. The correlation analysis is shown for RNA-seq data between the replicates. A – Mel Z cells grown on Plastic or Matrigel. B – MDA-MB-231 cells grown on plastic. C - MDA-MB-231 cells matrix grown.

Tables S1–S8

Table S1. List of downregulated and upregulated genes genes in MB cells grown on 3D matrix. The Excel file is attached separately.

Table S2. GO associations with molecular functions (MF), biological processes (BP), and cellular components (CC) of 1511 downregulated genes in MB cells grown on 3D matrix. Related to Figure 1B.

GO.ID	Description	padj	Genes
MF			
GO:0030527	structural constituent of chromatin	4.5613999942450925e-11	*H2AC21,H2BC14,H2AC15,H2BC3,H4C2,H2BC1,H3C13,H2AC12,H1-4,H2AC20,H2BC13,H2BC17,H2BC18,H2AC14,H2AC1,H2BC21,H2BC6,H2BC11,H1-6,H2BC15,H1-5,H2BC5,H1-2,H2AC6*
GO:0005102	signaling receptor binding	2.4558367962790114e-7	*CCL24,TNFSF18,BMP5,VCAM1,NDP,JAK3,CACNG4,AGR2,LRRTM2,OGN,MACC1,CXCL10,FGF13,NTF3,LGR6,ABCA1,ZP3,GNAZ,C1QL1,SNED1,FGF14,KIF5C,MUC4,CCL5,AR,ANGPT2,MEGF10,LEFTY1,ERBB4,IL1B,ADRA2C,DLL1,CADM1,ECM2,EGF,CSF3,ITGB6,TIAM1,SEMA6B,LTB,PIK3AP1,PTPRC,ITGBL1,P2RY1,GRK3,C5,ADAM28,RTP4,KLB,PLXNB1,GDF5,NOTCH2NLB,ANO1,LIFR,ENPP1,CRTAM,ITGA1,LCP1,SHH,CXCL11,MAP7,TNFSF4,DLG3,TNFSF10,PDGFB,SEMA6D,CPE,ITGA10,INHBB,PTCH2,SVEP1,SEMA3E,HIP1,PIK3CG,IL6,ITGB8,FZD1,HLA-DRA,CXCL8,PTPRJ,ATRNL1,ADRB1,DIAPH2,C3,PDGFA,PYCARD,PGF,HLA-G,ITGB4,BTC,HLA-DRB1,GMFG,SEMA3D,SPRED1,BEX2,IL1A,AKAP9,KCNJ8,LANCL1,NOTCH2NLA,MDM2*
GO:0005509	calcium ion binding	0.000010139359392841558	*PCDHGA8,TESC,DCHS1,PCDHGA11,PCDHGB7,PCDHGB5,TENM2,PADI3,MYL1,S100P,SNED1,FER1L5,PCDHGA2,PCDHGB4,NOX5,DLL1,EGF,PCDHGA12,PCDHGA10,PLCB4,PCDHGA4,PRSS2,PCDHB12,LRP1B,DNAH7,PCDH7,PCDHGB6,UNC13A,NOTCH2NLB,PCDHGB3,ENPP1,PCDHB11,C1R,LCP1,SHH,PCDHGA9,SPOCK3,FAM20C,SVEP1,PCDHB10,PCDHGB1,GJB2,CAPS2,ITPR2,GCA,FLG,PCDHGA1,PCDHGA5,PCDHGA7,ADGRV1,PCDHGA6,FAT1,C1S,CELSR1,PCDHB8,NOTCH2NLA,SYT2*
GO:0060089	molecular transducer activity	0.000198640757114442742	*SPN,PTPRN2,CCL24,KLRC2,GPRC5C,TNFSF18,BMP5,ADGRF1,NDP,COLEC12,TAS2R30,OGN,MACC1,PROKR1,CXCL10,FGF13,F2RL3,NTF3,KLRC3,GRIN2B,LGR6,LRRC19,ABCA1,ZP3,NTSR1,TNFRSF18,TAS2R13,FGF14,CCL5,AR,ANGPT2,PLXNA4,LEFTY1,TNFRSF9,ERBB4,TRHR,IL1B,ADRA2C,DLL1,EGF,CSF3,GPR176,TNFRSF8,ESR1,NPR3,PDE3A,SEMA6B,LTB,CCR1,PTPRC,PECAM1,IL20RA,P2RY1,C5,PLXNB1,GDF5,GPR162,GPR158,NPR1,LIFR,HLA-DPA1,GPC6,HLA-DOA,GPR85,SHH,GRIK4,CXCL11,RORB,AIM2,ABCC9,TNFSF4,NR5A2,TNFSF10,EPHB6,PDGFB,SEMA6D,P2RY6,INHBB,PTCH2,SEMA3E,CMKLR1,LEPR,IL6,TAS2R20,SORCS2,SORL1,FZD1,HLA-DRA,PTPN6,TNFRSF10C,LRP5,CXCL8,ADRB1,DMBT1,GPR55,FZD3,C3,PDGFA,ADGRV1,PGF,BTC,PAQR8,IL7R,HLA-DRB1,GPR153,GMFG,SEMA3D,CELSR1,AHR,ADGRF5,IL1A,LANCL1,TLR3,NRP1,IFIH1*
GO:0038023	signaling receptor activity	0.000198640757114442742	*SPN,PTPRN2,CCL24,KLRC2,GPRC5C,TNFSF18,BMP5,ADGRF1,NDP,COLEC12,TAS2R30,OGN,MACC1,PROKR1,CXCL10,FGF13,F2RL3,NTF3,KLRC3,GRIN2B,LGR6,LRRC19,ABCA1,ZP3,NTSR1,TNFRSF18,TAS2R13,FGF14,CCL5,AR,ANGPT2,PLXNA4,LEFTY1,TNFRSF9,ERBB4,TRHR,IL1B,ADRA2C,DLL1,EGF,CSF3,GPR176,TNFRSF8,ESR1,NPR3,PDE3A,SEMA6B,LTB,CCR1,PTPRC,PECAM1,IL20RA,P2RY1,C5,PLX*

			NB1,GDF5,GPR162,GPR158,NPR1,LIFR,HLA-DPA1,GPC6,HLA-DOA,GPR85,SHH,GRIK4,CXCL11,RORB,AIM2,ABCC9,TNFSF4,NR5A2,TNFSF10,EPHB6,PDGFB,SEMA6D,P2RY6,INHBB,PTCH2,SEMA3E,CMKLR1,LEPR,IL6,TAS2R20,SORCS2,SORL1,FZD1,HLA-DRA,PTPN6,TNFRSF10C,LRP5,CXCL8,ADRB1,DMBT1,GPR55,FZD3,C3,PDGFA,ADGRV1,PGF,BTC,PAQR8,IL7R,HLA-DRB1,GPR153,GMFG,SEMA3D,CELSR1,AHR,ADGRF5,IL1A,LANCL1,TLR3,NRP1,IFIH1
GO:0046982	protein heterodimerization activity	0.001250968935226416	*H2AC21,H2BC14,H2AC15,H2BC3,H4C2,H2BC1,H3C13,H2AC12,H2AC20,H2BC13,H2BC17,H2BC18,H2AC14,H2AC1,H2BC21,H2BC6,ABCG1,H2BC11,H2BC15,TENM2,H2BC5,ABCD2,ADRA2C,P2RY1,PDGFB,HIP1,GCA,JDP2,ADRB1,H2AC6,PDGFA,AHR*
GO:0023026	MHC class II protein complex binding	0.008127127842704883	*HLA-DPB1,HLA-DPA1,HLA-DOA,HLA-DRB5,HLA-DRA,HLA-DRB1,HLA-DMB*
GO:0023023	MHC protein complex binding	0.008797148877277046	*KLRC2,HLA-DPB1,HLA-DPA1,HLA-DOA,HLA-DRB5,HLA-DRA,HLA-DRB1,HLA-DMB*
GO:0030627	pre-mRNA 5'-splice site binding	0.010893843848586831	*RNVU1-31,RNVU1-28,RNU4ATAC,RNU1-1,RNVU1-7,RNVU1-34,RNVU1-8,RNVU1-29,RNVU1-3,RNU1-2,RNVU1-2,RNVU1-27,RNU6ATAC,RNU1-67P,ENSG00000287979,RNVU1-15,RNVU1-4,ENSG00000287190,RNVU1-30*
GO:0048018	receptor ligand activity	0.011750024183675706	*CCL24,TNFSF18,BMP5,NDP,OGN,MACC1,CXCL10,FGF13,NTF3,ZP3,FGF14,CCL5,ANGPT2,LEFTY1,IL1B,DLL1,EGF,CSF3,SEMA6B,LTB,C5,GDF5,SHH,CXCL11,TNFSF4,TNFSF10,PDGFB,SEMA6D,INHBB,SEMA3E,IL6,CXCL8,C3,PDGFA,PGF,BTC,GMFG,SEMA3D,IL1A*
GO:0031492	nucleosomal DNA binding	0.012436528082274217	*H2AC21,H2AC15,H2AC12,H1-4,H2AC20,H2AC14,H2AC1,H1-6,H1-5,H1-2,H2AC6*
GO:0030546	signaling receptor activator activity	0.015679236374619526	*CCL24,TNFSF18,BMP5,NDP,OGN,MACC1,CXCL10,FGF13,NTF3,ZP3,FGF14,CCL5,ANGPT2,LEFTY1,IL1B,DLL1,EGF,CSF3,SEMA6B,LTB,C5,GDF5,SHH,CXCL11,TNFSF4,TNFSF10,PDGFB,SEMA6D,INHBB,SEMA3E,IL6,CXCL8,C3,PDGFA,PGF,BTC,GMFG,SEMA3D,IL1A*
GO:0008083	growth factor activity	0.03681931103478555	*BMP5,OGN,MACC1,FGF13,NTF3,FGF14,LEFTY1,EGF,CSF3,GDF5,PDGFB,INHBB,IL6,PDGFA,PGF,BTC,GMFG*
		BP	
GO:0007155	cell adhesion	3.724203542280578e-11	*SPN,PCDHGA8,TNFSF18,BMP5,VCAM1,JAK3,BHLHA15,GLDN,TESC,DCHS1,PCDHGA11,AGR2,PCDHGB7,PCDHGB5,TENM2,F2RL3,VAV1,ZP3,TNFRSF18,SNED1,SOX4,MUC4,CCL5,LGALS12,ANGPT2,MEGF10,PLXNA4,PCDHGA2,SSPN,PCDHGB4,IL1B,DLL1,CADM1,ECM2,PCDHGA12,PCDHGA10,CLDN11,PCDHGA4,COL6A2,PRSS2,PCDHB12,HLA-DPB1,ITGB6,MPZL2,OMD,TIAM1,CCR1,PTPRC,ITGBL1,PDE5A,PECAM1,PCDH7,PCDHGB6,PLXNB1,JCAD,PCDHGB3,RADIL,HLA-DPA1,PCDHB11,DLC1,CRTAM,ITGA1,SOX2,HLA-DOA,C1QTNF1,SHH,TNFSF4,RGCC,DLG3,EPHB6,PDGFB,PRKG1,PCDHGA9,CNTNAP3B,HLA-DRB5,ITGA10,SVEP1,PCDHB10,SEMA3E,LAMB1,CARD11,PIK3CG,PCDHGB1,IL6,ITGB8,HLA-DRA,PTPN6,LRP5,CXCL8,PREX1,PCDHGA1,PTPRJ,*

			PCDHGA5,ILDR2,PYCARD,FUT2,PCDHGA7,ADGRV1,HLA-G,ITGB4,IL7R,HLA-DRB1,PCDHGA6,IGSF9B,FAT1,CELSR1,IL1A,PCDHB8,RAP2B,ST6GAL1,CLDN23,PRICKLE1,NRP1,HLA-DMB,KIF14,COL18A1
GO:0098609	cell-cell adhesion	8.365470406036056e-10	*SPN,PCDHGA8,BMP5,VCAM1,JAK3,BHLHA15,GLDN,DCHS1,PCDHGA11,PCDHGB7,PCDHGB5,TENM2,F2RL3,VAV1,ZP3,SOX4,CCL5,LGALS12,MEGF10,PCDHGA2,PCDHGB4,IL1B,CADM1,PCDHGA12,PCDHGA10,CLDN11,PCDHGA4,PCDHB12,HLA-DPB1,ITGB6,MPZL2,PTPRC,ITGBL1,PDE5A,PECAM1,PCDH7,PCDHGB6,PCDHGB3,HLA-DPA1,PCDHB11,CRTAM,ITGA1,SOX2,HLA-DOA,C1QTNF1,SHH,TNFSF4,RGCC,DLG3,EPHB6,PRKG1,PCDHGA9,HLA-DRB5,ITGA10,PCDHB10,LAMB1,CARD11,PIK3CG,PCDHGB1,IL6,ITGB8,HLA-DRA,PTPN6,LRP5,PCDHGA1,PCDHGA5,ILDR2,PYCARD,PCDHGA7,ADGRV1,HLA-G,ITGB4,IL7R,HLA-DRB1,PCDHGA6,FAT1,CELSR1,IL1A,PCDHB8,RAP2B,CLDN23,PRICKLE1,HLA-DMB*
GO:0007156	homophilic cell adhesion via plasma membrane adhesion molecules	3.5070789328713256e-8	*PCDHGA8,DCHS1,PCDHGA11,PCDHGB7,PCDHGB5,PCDHGA2,PCDHGB4,CADM1,PCDHGA12,PCDHGA10,PCDHGA4,PCDHB12,MPZL2,PECAM1,PCDH7,PCDHGB6,PCDHGB3,PCDHB11,PCDHGA9,PCDHB10,PCDHGB1,PCDHGA1,PCDHGA5,PCDHGA7,PCDHGA6,FAT1,CELSR1,PCDHB8*
GO:0048731	system development	3.595845939185191e-7	*LZTS1,PCDHGA8,CCL24,ASB2,TP63,BMP5,ADGRF1,HSD3B1,VCAM1,DHRS3,NDP,BHLHA15,GLDN,SCRG1,DCHS1,PCDHGA11,AGR2,LRRTM2,OGN,PCDHGB7,MEIS3,PCDHGB5,RP1L1,TENM2,CXCL10,FGF13,BCL11B,SLITRK6,NTF3,ASB4,GRIN2B,LGR6,ADAMTSL2,ZP3,NCMAP,ATP9A,C1QL1,CYP19A1,DMC1,FGFBP1,FGF14,KIF5C,GSX2,SOX4,ALDH1A2,AR,ANGPT2,PLXNA4,PCDHGA2,LEFTY1,COLQ,PCDHGB4,ABCD2,ERBB4,LRRN3,IL1B,ADRA2C,NOX5,DLL1,CADM1,EGF,PCDHGA12,MMP17,ST8SIA4,PCDHGA10,CLDN11,PLN,XIRP2,USH2A,PCDHGA4,SLC2A10,EYA1,GPM6A,SNAP25,PCDHB12,GCNT4,ESR1,PBX1,ITGB6,NPR3,TIAM1,MYLK,ULK2,SEMA6B,LTB,PARD6B,PTPRC,SPEF2,ANK2,PECAM1,LRRN1,LRRK2,PCDHGB6,P2RY1,SLC2A12,PLXNB1,GDF5,JCAD,UNC13A,NOTCH2NLB,ANO1,PCDHGB3,GPRIN3,GPR158,NPR1,PCDHB11,IRX5,DLC1,ITGA1,SOX2,SHH,RORB,SYNE2,AIM2,ABCC9,SPRY1,TNFSF4,INSIG1,RGCC,TNFSF10,STMN3,PDGFB,MAGED1,PITX2,PRKG1,FOXP2,SEMA6D,PCDHGA9,CPE,FAM20C,NEK3,MAP2K6,INHBB,IRX2,VASH2,SVEP1,PCDHB10,SEMA3E,CMKLR1,LEPR,ETV1,LAMB1,PIK3CG,CLMN,KREMEN1,PCDHGB1,GPRIN2,IL6,ITGB8,LFNG,SORL1,FZD1,RASSF2,PTPN6,LRP5,EVL,UST,CXCL8,PREX1,PPP1R9A,PCDHGA1,PTPRJ,IRX1,LRIG1,ARHGEF10,TMEM135,RO60,PCDHGA5,FZD3,C3,PDGFA,ATRX,PCDHGA7,ADGRV1,PGF,HLA-G,ITGB4,IL7R,PCDHGA6,MYO16,SH3TC2,IGSF9B,ZNF430,FAT1,HLTF,APBA2,SEMA3D,HOXA13,CELSR1,IRF2BPL,SPRED1,AHR,UGCG,ADGRF5,DIXDC1,IL1A,PCDHB8,MMP16,DDIT4,CRPPA,KCNJ8,SETX,ASPM,BICC1,SLC40A1,TLR3,ISM1,TTBK2,NOTCH2NLA,PRICKLE1,NRP1,APOLD1,SYT2,MDM2,MSX2,KIF14,IMMP2L,COL18A1*
GO:0007	multicellul	0.00000757	*H4C2,LZTS1,PCDHGA8,CCL24,ASB2,TP63,BMP5,A*

275	ar organism development	5050784462615	*DGRF1,HSD3B1,VCAM1,DHRS3,NDP,JAK3,BHLHA15,GLDN,ELF3,SCRG1,TESC,DCHS1,PCDHGA11,AGR2,LRRTM2,OGN,PCDHGB7,MEIS3,PCDHGB5,RP1L1,TENM2,CXCL10,FGF13,BCL11B,SLITRK6,NTF3,HCLS1,ASB4,GRIN2B,LGR6,ADAMTSL2,ZP3,NCMAP,ATP9A,C1QL1,CYP19A1,PPP4R4,DMC1,FGFBP1,FGF14,KIF5C,GSX2,SOX4,ALDH1A2,AR,ANGPT2,PLXNA4,PCDHGA2,LEFTY1,COLQ,PCDHGB4,ABCD2,ERBB4,LRRN3,IL1B,ADRA2C,NOX5,DLL1,CADM1,EGF,PCDHGA12,MMP17,ST8SIA4,ZBED6,PCDHGA10,CLDN11,PLN,CSF3,XIRP2,USH2A,PCDHGA4,SLC2A10,EYA1,GPM6A,SNAP25,PCDHB12,GCNT4,ESR1,PBX1,ITGB6,NPR3,TIAM1,MYLK,ULK2,SEMA6B,LTB,CCR1,PARD6B,PTPRC,SPEF2,ANK2,PECAM1,LRRN1,LRRK2,PCDHGB6,P2RY1,SLC2A12,PLXNB1,GDF5,JCAD,UNC13A,NOTCH2NLB,ANO1,PCDHGB3,GPRIN3,GPR158,NPR1,ENPP1,PCDHB11,IRX5,DLC1,CRTAM,ITGA1,SOX2,HLA-DOA,SHH,RORB,PLAAT4,SYNE2,AIM2,ABCC9,SPRY1,TNFSF4,INSIG1,RGCC,NR5A2,TNFSF10,STMN3,PDGFB,MAGED1,PITX2,PRKG1,FOXP2,SEMA6D,PCDHGA9,CPE,FAM20C,NEK3,MAP2K6,INHBB,PTCH2,IRX2,VASH2,SVEP1,PCDHB10,SEMA3E,CMKLR1,LEPR,ETV1,LAMB1,CARD11,PIK3CG,CLMN,KREMEN1,PCDHGB1,GJB2,GPRIN2,IL6,ITGB8,LFNG,SORL1,FZD1,RASSF2,HLA-DRA,PTPN6,LRP5,EVL,UST,CXCL8,PREX1,PPP1R9A,PCDHGA1,PTPRJ,IRX1,LRIG1,ARHGEF10,TMEM135,RO60,GPR55,PCDHGA5,FZD3,C3,PDGFA,ATRX,PCDHGA7,ADGRV1,PGF,HLA-G,ITGB4,IL7R,HLA-DRB1,PCDHGA6,MYO16,SH3TC2,IGSF9B,ZNF430,FAT1,HLTF,APBA2,SEMA3D,HOXA13,CELSR1,IRF2BPL,SPRED1,AHR,UGCG,ADGRF5,DIXDC1,IL1A,ADAMTS3,ANO6,PCDHB8,MMP16,DDIT4,CRPPA,KCNJ8,SETX,ASPM,BICC1,SLC40A1,TLR3,ISM1,TTBK2,NOTCH2NLA,PRICKLE1,NRP1,APOLD1,SYT2,MDM2,MSX2,KIF14,IMMP2L,COL18A1*
GO:0098742	cell-cell adhesion via plasma-membrane adhesion molecules	0.000008522043566285456	*PCDHGA8,VCAM1,DCHS1,PCDHGA11,PCDHGB7,PCDHGB5,TENM2,PCDHGA2,PCDHGB4,CADM1,PCDHGA12,PCDHGA10,CLDN11,PCDHGA4,PCDHB12,MPZL2,PECAM1,PCDH7,PCDHGB6,PCDHGB3,PCDHB11,CRTAM,PCDHGA9,PCDHB10,PCDHGB1,PCDHGA1,PCDHGA5,PCDHGA7,PCDHGA6,FAT1,CELSR1,PCDHB8,CLDN23*
GO:0007399	nervous system development	0.0000453875235210239 1	*LZTS1,PCDHGA8,TP63,BMP5,ADGRF1,HSD3B1,VCAM1,NDP,BHLHA15,GLDN,SCRG1,DCHS1,PCDHGA11,LRRTM2,PCDHGB7,MEIS3,PCDHGB5,RP1L1,TENM2,FGF13,BCL11B,SLITRK6,NTF3,GRIN2B,LGR6,NCMAP,ATP9A,C1QL1,FGF14,KIF5C,GSX2,SOX4,ALDH1A2,PLXNA4,PCDHGA2,COLQ,PCDHGB4,ABCD2,ERBB4,LRRN3,IL1B,ADRA2C,DLL1,EGF,PCDHGA12,ST8SIA4,PCDHGA10,CLDN11,USH2A,PCDHGA4,EYA1,GPM6A,SNAP25,PCDHB12,PBX1,TIAM1,ULK2,SEMA6B,PARD6B,SPEF2,ANK2,LRRN1,LRRK2,PCDHGB6,P2RY1,PLXNB1,GDF5,UNC13A,NOTCH2NLB,ANO1,PCDHGB3,GPRIN3,GPR158,PCDHB11,IRX5,DLC1,ITGA1,SOX2,SHH,RORB,SYNE2,AIM2,STMN3,PITX2,PRKG1,FOXP2,SEMA6D,PCDHGA9,NEK3,INHBB,IRX2,VASH2,PCDHB10,SEMA3E,LEPR,ETV1,LAMB1,CLMN,KREMEN1,PCDHGB1,GPRIN2,IL6,SORL1,FZD1,LRP5,EVL,UST,PREX1,PPP1R9A,PCDHGA1,PTPRJ,IRX1,LRIG1,ARHGEF10,PCDHGA5,FZD3,C3,ATRX,PCDHGA7,ADGRV1,ITGB4,PCDHGA6,MYO16,SH3TC2,IGSF9B,ZNF430,HLTF,APBA2,SEMA3D,CELSR1,I*

			RF2BPL,UGCG,DIXDC1,PCDHB8,DDIT4,CRPPA,KCNJ8,SETX,ASPM,TTBK2,NOTCH2NLA,PRICKLE1,NRP1,SYT2,MDM2,KIF14,IMMP2L
GO:0032502	developmental process	0.0001333432696850878 6	*H4C2,H2BC1,SPN,LZTS1,EDARADD,SERPINA5,PCDHGA8,CCL24,ASB2,KRT83,TP63,BMP5,ADGRF1,HSD3B1,VCAM1,DHRS3,NDP,JAK3,ABCG1,BHLHA15,GLDN,H1-6,SLC2A14,ELF3,SCRG1,TESC,DCHS1,PCDHGA11,AGR2,LRRTM2,OGN,PCDHGB7,MEIS3,PCDHGB5,RP1L1,TENM2,EPPK1,CXCL10,FGF13,ADAMTS20,BCL11B,STEAP4,SLITRK6,NTF3,HCLS1,NLRP14,ASB4,GRIN2B,VAV1,LGR6,SPANXB1,ABCA1,ADAMTSL2,ZP3,NCMAP,ATP9A,C1QL1,CYP19A1,CST6,PPP4R4,DMC1,FGFBP1,FGF14,H1-5,KIF5C,GSX2,WDR38,SOX4,ALDH1A2,AR,ANGPT2,MEGF10,PLXNA4,PCDHGA2,PPL,BTBD18,LEFTY1,COLQ,TNFRSF9,TEX15,PCDHGB4,NKX1-2,PRDM16,ABCD2,ERBB4,LRRN3,IL1B,ADRA2C,NOX5,DLL1,CADM1,EGF,PCDHGA12,MMP17,ST8SIA4,ZBED6,PCDHGA10,CLDN11,PLN,CSF3,XIRP2,USH2A,PCDHGA4,SLC2A10,EYA1,SLCO4C1,GPM6A,SNAP25,PCDHB12,GCNT4,ESR1,ALDOC,PBX1,FSIP2,ITGB6,NPR3,MPZL2,TIAM1,MYLK,TMT1A,PDE3A,ULK2,SEMA6B,TMEM232,LTB,CCR1,PARD6B,PTPRC,SPEF2,ANK2,PDE5A,PECAM1,EXPH5,FAM20A,LRRN1,LRRK2,PCDHGB6,P2RY1,SLC2A12,ADAM28,PLXNB1,GDF5,JCAD,MAEL,UNC13A,NOTCH2NLB,ANO1,PCDHGB3,GPRIN3,GPR158,KRT81,NPR1,RADIL,ENPP1,PLCG2,PCDHB11,IRX5,DLC1,KRT86,CRTAM,ITGA1,SOX2,HLA-DOA,LCP1,SHH,RORB,PLAAT4,SYNE2,AIM2,ABCC9,SPRY1,TNFSF4,INSIG1,MAP3K5,RGCC,NR5A2,DLG3,TNFSF10,STMN3,PDGFB,MAGED1,PITX2,PRKG1,FOXP2,SEMA6D,PCDHGA9,CPE,FAM20C,NEK3,MAP2K6,SLFN5,INHBB,PTCH2,TGM5,IRX2,VASH2,SVEP1,PCDHB10,SEMA3E,CMKLR1,HIP1,LEPR,ETV1,LAMB1,CARD11,PIK3CG,CLMN,KREMEN1,RBM47,PCDHGB1,GJB2,GPRIN2,IL6,ITGB8,LFNG,SORL1,FZD1,RASSF2,HLA-DRA,PTPN6,LRP5,EVL,ALDH6A1,UST,CXCL8,PREX1,PPP1R9A,FLG,PCDHGA1,PTPRJ,IRX1,LRIG1,ARHGEF10,TMEM135,LIN7C,JDP2,ADRB1,DMBT1,RO60,GPR55,DIAPH2,PCDHGA5,FZD3,C3,IRF2BP2,PDGFA,CEBPD,ILDR2,ATRX,PCDHGA7,ADGRV1,PGF,HLA-G,ITGB4,BTC,PAQR8,IL7R,HLA-DRB1,PCDHGA6,GLIPR2,MYO16,SH3TC2,IGSF9B,ZNF430,FAT1,NNMT,COCH,HLTF,SVIL,APBA2,SEMA3D,HOXA13,CELSR1,IRF2BPL,SPRED1,AHR,UGCG,ADGRF5,DIXDC1,IL1A,ADAMTS3,ANO6,PCDHB8,CDKL2,MMP16,DDIT4,CRPPA,KCNJ8,SETX,ASPM,BICC1,SLC40A1,TLR3,ISM1,TTBK2,NOTCH2NLA,PRICKLE1,NRP1,APOLD1,VSIG1,SYT2,MDM2,MSX2,KIF14,IMMP2L,COL18A1*
GO:0048856	anatomical structure development	0.0002377714506515531	*H4C2,H2BC1,SPN,LZTS1,PCDHGA8,CCL24,ASB2,KRT83,TP63,BMP5,ADGRF1,HSD3B1,VCAM1,DHRS3,NDP,JAK3,BHLHA15,GLDN,ELF3,SCRG1,TESC,DCHS1,PCDHGA11,AGR2,LRRTM2,OGN,PCDHGB7,MEIS3,PCDHGB5,RP1L1,TENM2,EPPK1,CXCL10,FGF13,BCL11B,SLITRK6,NTF3,HCLS1,ASB4,GRIN2B,VAV1,LGR6,SPANXB1,ADAMTSL2,ZP3,NCMAP,ATP9A,C1QL1,CYP19A1,CST6,PPP4R4,DMC1,FGFBP1,FGF14,H1-5,KIF5C,GSX2,WDR38,SOX4,ALDH1A2,AR,ANGPT2,MEGF10,PLXNA4,PCDHGA2,PPL,LEFTY1,COLQ,TN*

			FRSF9,PCDHGB4,ABCD2,ERBB4,LRRN3,IL1B,ADRA2C,NOX5,DLL1,CADM1,EGF,PCDHGA12,MMP17,ST8SIA4,ZBED6,PCDHGA10,CLDN11,PLN,CSF3,XIRP2,USH2A,PCDHGA4,SLC2A10,EYA1,GPM6A,SNAP25,PCDHB12,GCNT4,ESR1,ALDOC,PBX1,FSIP2,ITGB6,NPR3,MPZL2,TIAM1,MYLK,TMT1A,PDE3A,ULK2,SEMA6B,LTB,CCR1,PARD6B,PTPRC,SPEF2,ANK2,PDE5A,PECAM1,EXPH5,FAM20A,LRRN1,LRRK2,PCDHGB6,P2RY1,SLC2A12,PLXNB1,GDF5,JCAD,MAEL,UNC13A,NOTCH2NLB,ANO1,PCDHGB3,GPRIN3,GPR158,KRT81,NPR1,RADIL,ENPP1,PLCG2,PCDHB11,IRX5,DLC1,KRT86,CRTAM,ITGA1,SOX2,HLA-DOA,LCP1,SHH,RORB,PLAAT4,SYNE2,AIM2,ABCC9,SPRY1,TNFSF4,INSIG1,MAP3K5,RGCC,NR5A2,DLG3,TNFSF10,STMN3,PDGFB,MAGED1,PITX2,PRKG1,FOXP2,SEMA6D,PCDHGA9,CPE,FAM20C,NEK3,MAP2K6,INHBB,PTCH2,TGM5,IRX2,VASH2,SVEP1,PCDHB10,SEMA3E,CMKLR1,LEPR,ETV1,LAMB1,CARD11,PIK3CG,CLMN,KREMEN1,RBM47,PCDHGB1,GJB2,GPRIN2,IL6,ITGB8,LFNG,SORL1,FZD1,RASSF2,HLA-DRA,PTPN6,LRP5,EVL,UST,CXCL8,PREX1,PPP1R9A,FLG,PCDHGA1,PTPRJ,IRX1,LRIG1,ARHGEF10,TMEM135,LIN7C,DMBT1,RO60,GPR55,DIAPH2,PCDHGA5,FZD3,C3,IRF2BP2,PDGFA,CEBPD,ILDR2,ATRX,PCDHGA7,ADGRV1,PGF,HLA-G,ITGB4,PAQR8,IL7R,HLA-DRB1,PCDHGA6,GLIPR2,MYO16,SH3TC2,IGSF9B,ZNF430,FAT1,NNMT,COCH,HLTF,SVIL,APBA2,SEMA3D,HOXA13,CELSR1,IRF2BPL,SPRED1,AHR,UGCG,ADGRF5,DIXDC1,IL1A,ADAMTS3,ANO6,PCDHB8,MMP16,DDIT4,CRPPA,KCNJ8,SETX,ASPM,BICC1,SLC40A1,TLR3,ISM1,TTBK2,NOTCH2NLA,PRICKLE1,NRP1,APOLD1,VSIG1,SYT2,MDM2,MSX2,KIF14,IMMP2L,COL18A1
GO:0002503	peptide antigen assembly with MHC class II protein complex	0.00023816246491053578	*HLA-DPB1,HLA-DPA1,HLA-DOA,HLA-DRB5,HLA-DRA,HLA-DRB1,HLA-DMB*
GO:0002399	MHC class II protein complex assembly	0.00023816246491053578	*HLA-DPB1,HLA-DPA1,HLA-DOA,HLA-DRB5,HLA-DRA,HLA-DRB1,HLA-DMB*
GO:0042129	regulation of T cell proliferation	0.000381302496439571 1	*SPN,TNFSF18,VCAM1,ZP3,CCL5,LGALS12,TNFRSF9,IL1B,HLA-DPB1,PTPRC,PDE5A,HLA-DPA1,CRTAM,SHH,TNFSF4,CARD11,IL6,PTPN6,PYCARD,HLA-G,HLA-DRB1,IL1A,HLA-DMB*
GO:0042098	T cell proliferation	0.000637444600860429 2	*SPN,TNFSF18,VCAM1,ZP3,CCL5,LGALS12,TNFRSF9,IL1B,HLA-DPB1,PTPRC,PDE5A,HLA-DPA1,CRTAM,SHH,TNFSF4,EPHB6,CARD11,PIK3CG,IL6,PTPN6,PYCARD,HLA-G,HLA-DRB1,IL1A,HLA-DMB*
GO:0023052	signaling	0.000838843533882500 2	*GNG2,SPN,LZTS1,PTPRN2,EDARADD,CCL24,LAIR2,KLRC2,GPRC5C,ASB2,TNFSF18,TP63,BMP5,RASSF9,ADGRF1,DHRS3,NDP,JAK3,BHLHA15,CACNG4,GLDN,H2BC11,COLEC12,TAS2R30,DCHS1,MCF2L,AGR2,LRRTM2,OGN,MEIS3,MACC1,RP1L1,SLC30A10,RAPGEF4,PROKR1,TENM2,PDK4,CXCL10,FGF13,SHISA3,INSYN2A,ADAMTS20,RAB39A,F2RL3,NTF3,HCLS1,ARHGAP28,KLRC3,ASB4,GRIN2B,VAV1,LGR6*

			,LRRC19,ABCA1,ADAMTSL2,ZP3,GNAZ,NTSR1,CYP19A1,TNFRSF18,TAS2R13,FGFBP1,FGF14,DCDC2B,GSX2,SOX4,ALDH1A2,CCL5,AR,LGALS12,ANGPT2,FER1L5,RASSF6,PLXNA4,LEFTY1,COLQ,TRPM8,DDX60,PRDM16,ERBB4,TRHR,SUMO4,IL1B,ADRA2C,CPLX1,ATP2A3,DLL1,CADM1,EGF,ZBED6,PLN,PLCB4,CSF3,SLC2A10,EYA1,COL6A2,GPR176,PPP1R1C,SNAP25,HLA-DPB1,TNFRSF8,ESR1,STXBP5L,DUSP9,ITGB6,NPR3,TRIL,TIAM1,PDE3A,ULK2,RELL1,SEMA6B,SLC19A1,ASB14,LTB,CCR1,PIK3AP1,PTPRC,ANK2,ITGBL1,PDE5A,PECAM1,ALPK1,IL20RA,ARHGAP30,LRRK2,P2RY1,GRK3,C5,KLB,PLXNB1,GDF5,JCAD,MAEL,UNC13A,NOTCH2NLB,GPR162,ANO1,GPR158,NPR1,LIFR,SLC4A8,RADIL,ENPP1,PDK3,PLCG2,PCDHB11,DLC1,RAB40A,ITGA1,SOX2,GPC6,GPR85,LCP1,C1QTNF1,TRIM59,SHH,GRIK4,CXCL11,RORB,QRICH1,AIM2,ABCC9,SPRY1,TNFSF4,INSIG1,MAP3K5,NR5A2,DLG3,TNFSF10,EPHB6,STMN3,PDGFB,MAGED1,PRKG1,SEMA6D,CPE,FAM20C,P2RY6,ITGA10,MAP2K6,INHBB,PTCH2,SRGAP3,SVEP1,OASL,PCDHB10,SEMA3E,CMKLR1,BEST1,HIP1,LEPR,LAMB1,PARP14,KCTD12,CARD11,PIK3CG,GNG7,KREMEN1,CADPS2,RBM47,GJB2,IL6,DAPK2,ITGB8,TAS2R20,SORCS2,CTSS,LFNG,SORL1,ITPR2,FZD1,RASSF2,PTPN6,TNFRSF10C,SLC1A7,LRP5,EVL,CXCL8,PREX1,PPP1R9A,PTPRJ,RASSF8,ATRNL1,ARHGEF10,LIN7C,ADRB1,RO60,GPR55,RPS6KA2,CYTH4,FZD3,COL4A5,TRBV13,CGNL1,C3,PDGFA,CEBPD,ILDR2,PYCARD,NEURL1B,ATRX,ADGRV1,PGF,HLA-G,ITGB4,BTC,PAQR8,RBMS3,IL7R,HLA-DRB1,GPR153,GLIPR2,CHN2,MYO16,SH3TC2,KLHL24,FAT1,GMFG,APBA2,SEMA3D,HOXA13,CELSR1,SPRED1,MDFIC,AHR,UGCG,BEX2,LNPEP,ADGRF5,DIXDC1,IL1A,ADAMTS3,AKAP9,CXXC4,MVB12B,ANO6,RAP2B,PDE9A,CDKL2,DDIT4,KCNJ8,MX1,LANCL1,SNX13,ASPM,BICC1,TLR3,TTBK2,NOTCH2NLA,PDE10A,PRICKLE1,NRP1,IFIH1,SYT2,MDM2,APOL3,RGS5,MSX2,KIF14
GO:0051239	regulation of multicellular organismal process	0.0011660223967104562	H4C2,SPN,CCL24,KLRC2,TNFSF18,TP63,BMP5,VCAM1,DHRS3,JAK3,TESC,LRRTM2,ZBTB20,EPPK1,PDK4,CXCL10,FGF13,SLITRK6,HCLS1,KLRC3,GRIN2B,VAV1,LRRC19,ABCA1,ZP3,NTSR1,NCMAP,CYP19A1,GSX2,SOX4,CCL5,AR,LGALS12,ANGPT2,PLXNA4,TNFRSF9,TRPM8,PRDM16,ABCD2,ERBB4,LRRN3,IL1B,ADRA2C,NOX5,ATP2A3,DLL1,CADM1,EGF,LIPG,PLN,CSF3,SLC2A10,HLA-DPB1,TNFRSF8,ESR1,PBX1,NPR3,TRIL,TIAM1,PDE3A,ULK2,LTB,CCR1,PTPRC,XAF1,ANK2,PDE5A,IL20RA,LRRN1,LRRK2,P2RY1,C5,PLXNB1,GDF5,JCAD,MAEL,NPR1,ENPP1,PLCG2,HLA-DPA1,TFPI,CRTAM,HLA-DOA,C1QTNF1,SHH,PLAAT4,AIM2,ABCC9,SPRY1,TNFSF4,RGCC,EPHB6,PDGFB,MAGED1,PRKG1,SEMA6D,HLA-DRB5,FAM20C,MAP2K6,INHBB,PTCH2,VASH2,SVEP1,TBXAS1,SEMA3E,CMKLR1,LEPR,CARD11,PIK3CG,KREMEN1,RBM47,IL6,ITGB8,LFNG,SORL1,RASSF2,HLA-DRA,PTPN6,EVL,CXCL8,PTPRJ,ADRB1,GPR55,FZD3,C3,PDGFA,ILDR2,PYCARD,ADGRV1,PGF,HLA-G,IL7R,HLA-DRB1,GLIPR2,HLTF,SPRED1,AHR,APOM,LNPEP,ADGRF5,IL1A,AKAP9,ANO6,PDE9A,KCNJ8,ASPM,TLR

			3,ISM1,PRICKLE1,NRP1,APOLD1,IFIH1,MDM2,HLA-DMB,MSX2,KIF14
GO:0032501	multicellular organismal process	0.001175545560307589 5	H4C2,SPN,LZTS1,F13A1,PCDHGA8,CCL24,KLRC2,ASB2,TNFSF18,KRT83,TP63,BMP5,ADGRF1,HSD3B1,VCAM1,DHRS3,NDP,JAK3,ABCG1,BHLHA15,CACNG4,GLDN,TAS2R30,ELF3,SCRG1,TESC,DCHS1,PCDHGA11,AGR2,LRRTM2,OGN,SCN3A,PCDHGB7,MEIS3,PCDHGB5,RP1L1,SERPINA1,TENM2,ZBTB20,EPPK1,PDK4,CXCL10,FGF13,INSYN2A,BCL11B,F2RL3,SLITRK6,NTF3,HCLS1,KLRC3,ASB4,GRIN2B,VAV1,LGR6,LRRC19,ABCA1,ADAMTSL2,ZP3,NTSR1,MYL1,NCMAP,ATP9A,C1QL1,CYP19A1,PPP4R4,DMC1,TAS2R13,FGFBP1,FGF14,KIF5C,GSX2,CKMT2,SOX4,MUC4,ALDH1A2,CCL5,AR,LGALS12,ANGPT2,TMPRSS3,MEGF10,PLXNA4,PCDHGA2,PPL,SSPN,LEFTY1,COLQ,TNFRSF9,TRPM8,PCDHGB4,PRDM16,CCND2,ABCD2,ERBB4,LRRN3,IL1B,ADRA2C,NOX5,ATP2A3,DLL1,CADM1,EGF,PCDHGA12,MMP17,ST8SIA4,ZBED6,LIPG,PCDHGA10,CLDN11,PLN,CSF3,XIRP2,USH2A,PCDHGA4,SLC2A10,EYA1,GPR176,GPM6A,SNAP25,PRSS2,PCDHB12,GCNT4,HLA-DPB1,CRACD,TNFRSF8,ESR1,PBX1,ITGB6,NPR3,TRIL,TIAM1,MYLK,TMT1A,PDE3A,ULK2,SEMA6B,SLC19A1,LTB,CCR1,PARD6B,PTPRC,ABCC6,XAF1,SPEF2,ANK2,PDE5A,PECAM1,IL20RA,LRRN1,LRRK2,PCDHGB6,P2RY1,SLC2A12,C5,RTP4,PLXNB1,GDF5,JCAD,MAEL,UNC13A,NOTCH2NLB,ANO1,PCDHGB3,GPRIN3,GPR158,KRT81,NPR1,SLC4A8,ENPP1,PLCG2,HLA-DPA1,PCDHB11,IRX5,DLC1,TFPI,KRT86,CRTAM,ITGA1,SOX2,HLA-DOA,LCP1,C1QTNF1,SHH,RORB,PLAAT4,SYNE2,AIM2,ABCC9,SPRY1,TNFSF4,INSIG1,RGCC,NR5A2,TNFSF10,EPHB6,STMN3,PDGFB,MAGED1,PITX2,PRKG1,FOXP2,SEMA6D,PCDHGA9,CPE,HLA-DRB5,FAM20C,NEK3,MAP2K6,INHBB,PTCH2,IRX2,VASH2,SVEP1,TBXAS1,PCDHB10,FLVCR2,SEMA3E,CMKLR1,BEST1,CLIC5,LEPR,ETV1,LAMB1,CARD11,PIK3CG,TMEM150C,CLMN,GNG7,KREMEN1,RBM47,PCDHGB1,GJB2,GPRIN2,IL6,ITGB8,TAS2R20,LFNG,SORL1,ALOX5AP,FZD1,RASSF2,HLA-DRA,PTPN6,SLC1A7,LRP5,EVL,UST,CXCL8,PREX1,PPP1R9A,PCDHGA1,PTPRJ,IRX1,LRIG1,ARHGEF10,TMEM135,ADRB1,RO60,GPR55,PCDHGA5,FZD3,C3,IRF2BP2,PDGFA,CEBPD,ILDR2,PYCARD,ATRX,PCDHGA7,ADGRV1,PGF,HLA-G,ITGB4,BTC,IL7R,HLA-DRB1,PCDHGA6,GLIPR2,MYO16,RCSD1,SH3TC2,IGSF9B,ZNF430,FAT1,COCH,HLTF,APBA2,SEMA3D,HOXA13,CELSR1,IRF2BPL,SPRED1,CALHM3,AHR,UGCG,APOM,LNPEP,ADGRF5,DIXDC1,IL1A,ADAMTS3,AKAP9,ANO6,PCDHB8,RAP2B,PDE9A,MMP16,DDIT4,CRPPA,KCNJ8,SETX,ASPM,BICC1,SLC40A1,TLR3,ISM1,TTBK2,NOTCH2NLA,PRICKLE1,NRP1,APOLD1,VSIG1,IFIH1,SYT2,MDM2,HLA-DMB,MSX2,KIF14,IMMP2L,COL18A1
GO:0007154	cell communication	0.001222245551238671 5	GNG2,SPN,LZTS1,PTPRN2,EDARADD,CCL24,LAIR2,KLRC2,GPRC5C,ASB2,TNFSF18,TP63,BMP5,RASSF9,ADGRF1,DHRS3,NDP,JAK3,BHLHA15,CACNG4,GLDN,H2BC11,COLEC12,TAS2R30,DCHS1,MCF2L,AGR2,LRRTM2,OGN,MEIS3,MACC1,RP1L1,SLC30A10,RAPGEF4,PROKR1,TENM2,PDK4,CXCL10,FGF13,SHISA3,INSYN2A,ADAMTS20,RAB39A,F2RL3,NTF3,HCLS1,ARHGAP28,KLRC3,ASB4,GRIN2B,VAV1,LGR6

			,LRRC19,ABCA1,ADAMTSL2,ZP3,GNAZ,NTSR1,CYP19A1,TNFRSF18,TAS2R13,FGFBP1,FGF14,DCDC2B,GSX2,SOX4,ALDH1A2,CCL5,AR,LGALS12,ANGPT2,FER1L5,RASSF6,PLXNA4,LEFTY1,COLQ,TRPM8,DDX60,PRDM16,ERBB4,TRHR,SUMO4,IL1B,ADRA2C,CPLX1,ATP2A3,DLL1,CADM1,EGF,ZBED6,PLN,PLCB4,CSF3,SLC2A10,EYA1,COL6A2,GPR176,PPP1R1C,SNAP25,HLA-DPB1,TNFRSF8,ESR1,STXBP5L,DUSP9,ITGB6,NPR3,TRIL,TIAM1,PDE3A,ULK2,RELL1,SEMA6B,SLC19A1,ASB14,LTB,CCR1,PIK3AP1,PTPRC,ANK2,ITGBL1,PDE5A,PECAM1,ALPK1,IL20RA,ARHGAP30,LRRK2,P2RY1,GRK3,C5,KLB,PLXNB1,GDF5,JCAD,MAEL,UNC13A,NOTCH2NLB,GPR162,ANO1,GPR158,NPR1,LIFR,SLC4A8,RADIL,ENPP1,PDK3,PLCG2,PCDHB11,DLC1,RAB40A,ITGA1,SOX2,GPC6,GPR85,LCP1,C1QTNF1,TRIM59,SHH,GRIK4,CXCL11,RORB,QRICH1,AIM2,ABCC9,SPRY1,TNFSF4,INSIG1,MAP3K5,NR5A2,DLG3,TNFSF10,EPHB6,STMN3,PDGFB,MAGED1,PRKG1,SEMA6D,CPE,FAM20C,P2RY6,ITGA10,MAP2K6,INHBB,PTCH2,SRGAP3,SVEP1,OASL,PCDHB10,SEMA3E,CMKLR1,BEST1,HIP1,LEPR,LAMB1,PARP14,KCTD12,CARD11,PIK3CG,GNG7,KREMEN1,CADPS2,RBM47,GJB2,IL6,DAPK2,ITGB8,TAS2R20,SORCS2,CTSS,LFNG,SORL1,ITPR2,FZD1,RASSF2,PTPN6,TNFRSF10C,SLC1A7,LRP5,EVL,CXCL8,PREX1,PPP1R9A,PTPRJ,RASSF8,ATRNL1,ARHGEF10,LIN7C,ADRB1,RO60,GPR55,RPS6KA2,CYTH4,FZD3,COL4A5,TRBV13,CGNL1,C3,PDGFA,CEBPD,ILDR2,PYCARD,NEURL1B,ATRX,ADGRV1,PGF,HLA-G,ITGB4,BTC,PAQR8,RBMS3,IL7R,HLA-DRB1,GPR153,GLIPR2,CHN2,MYO16,SH3TC2,KLHL24,FAT1,GMFG,APBA2,SEMA3D,HOXA13,CELSR1,SPRED1,MDFIC,AHR,UGCG,BEX2,LNPEP,ADGRF5,DIXDC1,IL1A,ADAMTS3,AKAP9,CXXC4,MVB12B,ANO6,RAP2B,PDE9A,CDKL2,DDIT4,KCNJ8,MX1,LANCL1,SNX13,ASPM,BICC1,TLR3,TTBK2,NOTCH2NLA,PDE10A,PRICKLE1,NRP1,IFIH1,SYT2,MDM2,APOL3,RGS5,MSX2,KIF14
GO:0007166	cell surface receptor signaling pathway	0.001264981540785460 6	SPN,CCL24,KLRC2,TNFSF18,TP63,BMP5,ADGRF1,NDP,JAK3,H2BC11,AGR2,SLC30A10,PDK4,CXCL10,SHISA3,INSYN2A,NTF3,HCLS1,KLRC3,GRIN2B,VAV1,LGR6,ABCA1,ADAMTSL2,NTSR1,TNFRSF18,FGFBP1,GSX2,SOX4,CCL5,AR,ANGPT2,PLXNA4,LEFTY1,PRDM16,ERBB4,IL1B,DLL1,CADM1,EGF,PLN,CSF3,SLC2A10,EYA1,HLA-DPB1,ITGB6,TRIL,TIAM1,SEMA6B,LTB,CCR1,PIK3AP1,PTPRC,ITGBL1,PECAM1,IL20RA,LRRK2,P2RY1,C5,KLB,PLXNB1,GDF5,JCAD,NOTCH2NLB,NPR1,LIFR,ENPP1,PLCG2,ITGA1,SOX2,SHH,GRIK4,CXCL11,AIM2,SPRY1,TNFSF10,EPHB6,PDGFB,SEMA6D,CPE,FAM20C,P2RY6,ITGA10,INHBB,PTCH2,SVEP1,OASL,SEMA3E,CMKLR1,HIP1,LEPR,LAMB1,PARP14,CARD11,GNG7,KREMEN1,RBM47,IL6,ITGB8,LFNG,SORL1,FZD1,PTPN6,TNFRSF10C,SLC1A7,LRP5,EVL,CXCL8,PTPRJ,RO60,FZD3,COL4A5,TRBV13,C3,PDGFA,PYCARD,NEURL1B,ADGRV1,PGF,HLA-G,ITGB4,BTC,RBMS3,IL7R,HLA-DRB1,SH3TC2,SEMA3D,HOXA13,CELSR1,SPRED1,MDFIC,UGCG,ADGRF5,DIXDC1,IL1A,ADAMTS3,CXXC4,MVB12B,ANO6,DDIT4,MX1,ASPM,BICC1,TLR3,TTBK2,NOTCH2NLA,PRICKLE1,NRP1,IFIH1,MSX2
GO:0042127	regulation of cell	0.001326169558384112	SPN,CCL24,TNFSF18,TP63,BMP5,VCAM1,TESC,OGN,MEIS3,EPPK1,CXCL10,BCL11B,NTF3,HCLS1,ZP

	population proliferation	3	*3,FGFBP1,SOX4,ALDH1A2,CCL5,AR,LGALS12,MEGF10,TNFRSF9,CCND2,ERBB4,IL1B,DLL1,EGF,CSF3,EYA1,FABP6,HLA-DPB1,TNFRSF8,ESR1,PBX1,NPR3,TIAM1,ST8SIA1,EGLN3,PTPRC,PDE5A,LRRK2,KLB,PLXNB1,GDF5,JCAD,NPR1,LIFR,HLA-DPA1,DLC1,P3H3,CRTAM,ITGA1,SOX2,SHH,CXCL11,PLAAT4,SPRY1,TNFSF4,MAP3K5,RGCC,NR5A2,DLG3,PDGFB,MAGED1,PITX2,PRKG1,P2RY6,VASH2,LAMB1,CARD11,CLMN,IL6,PTPN6,LRP5,CXCL8,PTPRJ,RPS6KA2,FZD3,H2AC6,PDGFA,PYCARD,FUT2,PGF,HLA-G,BTC,IL7R,HLA-DRB1,MYO16,AHR,IL1A,ST6GAL1,PDE9A,ASPM,NRP1,MDM2,HLA-DMB,MSX2,KIF14,COL18A1*
GO:0016477	cell migration	0.001375818103665739 4	*H2BC1,SPN,CCL24,ASB2,TNFSF18,BMP5,VCAM1,DCHS1,EPPK1,CXCL10,FGF13,BCL11B,NTF3,ZG16B,VAV1,LGR6,ZP3,S100P,CYP19A1,TNFRSF18,FGFBP1,GSX2,CCL5,ANGPT2,MEGF10,PLXNA4,ERBB4,IL1B,EGF,GPM6A,TIAM1,MYLK,SEMA6B,CCR1,PARD6B,PTPRC,PECAM1,LRRK2,P2RY1,C5,PLXNB1,JCAD,RADIL,PLCG2,DLC1,CRTAM,ITGA1,GPC6,LCP1,SHH,CXCL11,SYNE2,RGCC,PDGFB,PITX2,PRKG1,SEMA6D,P2RY6,SRGAP3,SEMA3E,CMKLR1,LAMB1,PIK3CG,IL6,DAPK2,SORL1,LRP5,EVL,CXCL8,PREX1,PTPRJ,FZD3,PDGFA,PYCARD,PGF,ITGB4,GLIPR2,FAT1,SEMA3D,CELSR1,SPRED1,DIXDC1,IL1A,ANO6,RAP2B,DDIT4,ASPM,TTBK2,PRICKLE1,NRP1,MDM2,MSX2,KIF14*
GO:0007267	cell-cell signaling	0.001407495320589828 5	*LZTS1,PTPRN2,CCL24,TNFSF18,TP63,BHLHA15,CACNG4,LRRTM2,RAPGEF4,TENM2,CXCL10,FGF13,INSYN2A,NTF3,GRIN2B,ABCA1,GNAZ,NTSR1,CYP19A1,FGFBP1,FGF14,SOX4,CCL5,AR,FER1L5,COLQ,IL1B,ADRA2C,CPLX1,DLL1,ZBED6,PLCB4,GPR176,SNAP25,STXBP5L,TIAM1,LTB,CCR1,ANK2,LRRK2,P2RY1,GDF5,UNC13A,ANO1,GPR158,SLC4A8,PCDHB11,C1QTNF1,SHH,GRIK4,CXCL11,DLG3,TNFSF10,PDGFB,CPE,MAP2K6,INHBB,PCDHB10,BEST1,HIP1,CADPS2,GJB2,IL6,SORCS2,FZD1,SLC1A7,LRP5,PPP1R9A,LIN7C,RPS6KA2,PDGFA,ILDR2,PGF,HLA-DRB1,FAT1,APBA2,LNPEP,IL1A,AKAP9,PDE9A,KCNJ8,NRP1,SYT2,MDM2*
GO:0030155	regulation of cell adhesion	0.001633893156588735 4	*SPN,TNFSF18,VCAM1,JAK3,TESC,AGR2,VAV1,ZP3,TNFRSF18,SOX4,CCL5,LGALS12,ANGPT2,MEGF10,PLXNA4,IL1B,DLL1,ECM2,PRSS2,HLA-DPB1,PTPRC,PDE5A,PLXNB1,HLA-DPA1,DLC1,CRTAM,SOX2,HLA-DOA,C1QTNF1,SHH,TNFSF4,RGCC,EPHB6,PDGFB,PRKG1,HLA-DRB5,SEMA3E,LAMB1,CARD11,PIK3CG,IL6,HLA-DRA,PTPN6,CXCL8,PREX1,PTPRJ,ILDR2,PYCARD,FUT2,HLA-G,IL7R,HLA-DRB1,IL1A,ST6GAL1,NRP1,HLA-DMB,KIF14*
GO:0048584	positive regulation of response to stimulus	0.002045565694840543	*CCL24,KLRC2,TP63,BMP5,NDP,COLEC12,MCF2L,AGR2,MEIS3,SLC30A10,CXCL10,ADAMTS20,F2RL3,NTF3,HCLS1,KLRC3,GRIN2B,VAV1,LGR6,LRRC19,ZP3,FGFBP1,GSX2,SOX4,CCL5,AR,MGMT,DDX60,ERBB4,IL1B,ADRA2C,DLL1,CADM1,EGF,CSF3,SLC2A10,EYA1,HLA-DPB1,ESR1,TRIL,TIAM1,MYLK,RELL1,SLC19A1,LTB,CCR1,PIK3AP1,PTPRC,ALPK1,IL20RA,LRRK2,P2RY1,C5,KLB,PLXNB1,GDF5,JCAD,NOTCH2NLB,NPR1,PLCG2,HLA-DPA1,CRTAM,C1R,ITGA1,SOX2,HLA-*

			DOA,C1QTNF1,SHH,AIM2,TNFSF4,MAP3K5,RGCC,TNFSF10,PDGFB,MAGED1,HLA-DRB5,P2RY6,MAP2K6,INHBB,SVEP1,OASL,SEMA3E,CMKLR1,HIP1,LAMB1,PARP14,CARD11,PIK3CG,RBM47,IL6,DAPK2,CTSS,LFNG,SORL1,CFD,ALOX5AP,RASSF2,HLA-DRA,PTPN6,CXCL8,PTPRJ,ADRB1,GPR55,C3,PDGFA,PYCARD,ADGRV1,PGF,HLA-G,IL7R,HLA-DRB1,GLIPR2,C1S,SPRED1,DIXDC1,IL1A,ADAMTS3,ANO6,KCNJ8,ASPM,C2,TLR3,NOTCH2NLA,NRP1,IFIH1,HLA-DMB,APOL3,MSX2
GO:0008283	cell population proliferation	0.002155257233889859 6	*GNG2,SPN,CCL24,TNFSF18,TP63,BMP5,VCAM1,NDP,SCRG1,TESC,DCHS1,OGN,MEIS3,EPPK1,CXCL10,FGF13,BCL11B,NTF3,HCLS1,LGR6,ZP3,FGFBP1,SOX4,ALDH1A2,CCL5,AR,LGALS12,MEGF10,TNFRSF9,CCND2,ERBB4,IL1B,NOX5,DLL1,EGF,CSF3,EYA1,FABP6,HLA-DPB1,TNFRSF8,ESR1,PBX1,NPR3,TIAM1,ST8SIA1,EGLN3,PTPRC,PDE5A,LRRK2,KLB,PLXNB1,GDF5,JCAD,NPR1,LIFR,HLA-DPA1,DLC1,P3H3,CRTAM,ITGA1,SOX2,SHH,CXCL11,PLAAT4,ABCC9,SPRY1,TNFSF4,MAP3K5,RGCC,NR5A2,DLG3,EPHB6,PDGFB,MAGED1,PITX2,PRKG1,P2RY6,VASH2,LEPR,LAMB1,CARD11,PIK3CG,CLMN,IL6,PTPN6,LRP5,CXCL8,PTPRJ,RPS6KA2,FZD3,H2AC6,PDGFA,PYCARD,FUT2,PGF,HLA-G,BTC,IL7R,HLA-DRB1,MYO16,AHR,DIXDC1,IL1A,ST6GAL1,PDE9A,MMP16,KCNJ8,ASPM,NRP1,MDM2,HLA-DMB,MSX2,KIF14,COL18A1*
GO:0002495	antigen processing and presentation of peptide antigen via MHC class II	0.002871064312980784 5	*HLA-DPB1,HLA-DPA1,HLA-DOA,HLA-DRB5,CTSS,HLA-DRA,PYCARD,HLA-DRB1,HLA-DMB*
GO:0002501	peptide antigen assembly with MHC protein complex	0.003766572191224931 4	*HLA-DPB1,HLA-DPA1,HLA-DOA,HLA-DRB5,HLA-DRA,HLA-DRB1,HLA-DMB*
GO:0002396	MHC protein complex assembly	0.003766572191224931 4	*HLA-DPB1,HLA-DPA1,HLA-DOA,HLA-DRB5,HLA-DRA,HLA-DRB1,HLA-DMB*
GO:0007165	signal transduction	0.004146256725920777	*GNG2,SPN,EDARADD,CCL24,LAIR2,KLRC2,GPRC5C,ASB2,TNFSF18,TP63,BMP5,RASSF9,ADGRF1,DHRS3,NDP,JAK3,BHLHA15,CACNG4,GLDN,H2BC11,COLEC12,TAS2R30,DCHS1,MCF2L,AGR2,OGN,MEIS3,MACC1,RP1L1,SLC30A10,RAPGEF4,PROKR1,TENM2,PDK4,CXCL10,FGF13,SHISA3,INSYN2A,ADAMTS20,RAB39A,F2RL3,NTF3,HCLS1,ARHGAP28,KLRC3,ASB4,GRIN2B,VAV1,LGR6,LRRC19,ABCA1,ADAMTSL2,ZP3,GNAZ,NTSR1,TNFRSF18,TAS2R13,FGFBP1,FGF14,DCDC2B,GSX2,SOX4,ALDH1A2,CCL5,AR,LGALS12,ANGPT2,RASSF6,PLXNA4,LEFTY1,TRPM8,DDX60,PRDM16,ERBB4,TRHR,SUMO4,IL1B,ADRA2C,ATP2A3,DLL1,CADM1,EGF,PLN,PLCB4,CSF3,SLC2A10,EYA1,COL6A2,GPR176,PPP1R1C,HLA-DPB1,TNFRSF8,ESR1,DUSP9,ITGB6,NPR3,TRIL,T*

			IAM1,PDE3A,ULK2,RELL1,SEMA6B,SLC19A1,ASB14,LTB,CCR1,PIK3AP1,PTPRC,ANK2,ITGBL1,PDE5A,PECAM1,ALPK1,IL20RA,ARHGAP30,LRRK2,P2RY1,GRK3,C5,KLB,PLXNB1,GDF5,JCAD,MAEL,NOTCH2NLB,GPR162,ANO1,GPR158,NPR1,LIFR,RADIL,ENPP1,PDK3,PLCG2,DLC1,RAB40A,ITGA1,SOX2,GPC6,GPR85,LCP1,C1QTNF1,TRIM59,SHH,GRIK4,CXCL11,RORB,QRICH1,AIM2,ABCC9,SPRY1,TNFSF4,INSIG1,MAP3K5,NR5A2,TNFSF10,EPHB6,STMN3,PDGFB,MAGED1,PRKG1,SEMA6D,CPE,FAM20C,P2RY6,ITGA10,MAP2K6,INHBB,PTCH2,SRGAP3,SVEP1,OASL,SEMA3E,CMKLR1,HIP1,LEPR,LAMB1,PARP14,KCTD12,CARD11,PIK3CG,GNG7,KREMEN1,RBM47,IL6,DAPK2,ITGB8,TAS2R20,SORCS2,CTSS,LFNG,SORL1,ITPR2,FZD1,RASSF2,PTPN6,TNFRSF10C,SLC1A7,LRP5,EVL,CXCL8,PREX1,PPP1R9A,PTPRJ,RASSF8,ATRNL1,ARHGEF10,ADRB1,RO60,GPR55,RPS6KA2,CYTH4,FZD3,COL4A5,TRBV13,CGNL1,C3,PDGFA,CEBPD,PYCARD,NEURL1B,ATRX,ADGRV1,PGF,HLA-G,ITGB4,BTC,PAQR8,RBMS3,IL7R,HLA-DRB1,GPR153,GLIPR2,CHN2,MYO16,SH3TC2,KLHL24,GMFG,SEMA3D,HOXA13,CELSR1,SPRED1,MDFIC,AHR,UGCG,BEX2,ADGRF5,DIXDC1,IL1A,ADAMTS3,AKAP9,CXXC4,MVB12B,ANO6,RAP2B,PDE9A,CDKL2,DDIT4,KCNJ8,MX1,LANCL1,SNX13,ASPM,BICC1,TLR3,TTBK2,NOTCH2NLA,PDE10A,PRICKLE1,NRP1,IFIH1,MDM2,APOL3,RGS5,MSX2,KIF14
GO:0030334	regulation of cell migration	0.004302118487226495	*SPN,CCL24,TNFSF18,BMP5,EPPK1,CXCL10,NTF3,LGR6,ZP3,CYP19A1,TNFRSF18,FGFBP1,GSX2,CCL5,ANGPT2,PLXNA4,ERBB4,IL1B,EGF,TIAM1,MYLK,SEMA6B,CCR1,PARD6B,PTPRC,PECAM1,C5,PLXNB1,JCAD,PLCG2,DLC1,SHH,SYNE2,RGCC,PDGFB,PRKG1,SEMA6D,P2RY6,SRGAP3,SEMA3E,CMKLR1,LAMB1,PIK3CG,IL6,DAPK2,SORL1,EVL,CXCL8,PREX1,PTPRJ,PDGFA,PYCARD,PGF,GLIPR2,FAT1,SEMA3D,SPRED1,IL1A,ANO6,RAP2B,TTBK2,NRP1,MDM2,KIF14*
GO:0141124	intracellular signaling cassette	0.004675492536088890	*BMP5,NDP,BHLHA15,MCF2L,AGR2,MEIS3,SLC30A10,RAPGEF4,FGF13,RAB39A,F2RL3,NTF3,HCLS1,ARHGAP28,GRIN2B,VAV1,LRRC19,ABCA1,FGF14,CCL5,AR,TRPM8,ERBB4,SUMO4,IL1B,ADRA2C,EGF,PLN,CSF3,COL6A2,ESR1,DUSP9,TIAM1,PDE3A,RELL1,LTB,CCR1,PIK3AP1,PTPRC,ANK2,PDE5A,ALPK1,ARHGAP30,LRRK2,P2RY1,KLB,PLXNB1,GDF5,JCAD,NPR1,PLCG2,DLC1,ITGA1,SOX2,C1QTNF1,TRIM59,QRICH1,AIM2,ABCC9,SPRY1,MAP3K5,NR5A2,TNFSF10,STMN3,PDGFB,PRKG1,P2RY6,MAP2K6,SRGAP3,SEMA3E,CMKLR1,HIP1,CARD11,PIK3CG,IL6,ITPR2,RASSF2,PTPN6,CXCL8,PREX1,PPP1R9A,PTPRJ,ARHGEF10,ADRB1,GPR55,CYTH4,CGNL1,PDGFA,CEBPD,PYCARD,HLA-G,HLA-DRB1,GLIPR2,CHN2,MYO16,CELSR1,SPRED1,MDFIC,AHR,IL1A,RAP2B,PDE9A,KCNJ8,TLR3,PDE10A,NRP1,APOL3,KIF14*
GO:0032944	regulation of mononuclear cell proliferation	0.005763148900993934	*SPN,TNFSF18,VCAM1,ZP3,CCL5,LGALS12,TNFRSF9,IL1B,HLA-DPB1,PTPRC,PDE5A,HLA-DPA1,CRTAM,SHH,TNFSF4,CARD11,IL6,PTPN6,PYCARD,HLA-G,HLA-DRB1,AHR,IL1A,ST6GAL1,HLA-DMB*
GO:0030335	positive regulation of cell	0.006821679457039099	*SPN,CCL24,TNFSF18,CXCL10,NTF3,LGR6,ZP3,TNFRSF18,FGFBP1,CCL5,IL1B,EGF,TIAM1,MYLK,SEMA6B,CCR1,PTPRC,PECAM1,JCAD,PLCG2,SYNE2,P*

	migration		*DGFB,SEMA6D,P2RY6,SEMA3E,CMKLR1,LAMB1,PIK3CG,IL6,DAPK2,CXCL8,PREX1,PTPRJ,PDGFA,PYCARD,PGF,GLIPR2,FAT1,SEMA3D,IL1A,ANO6,NRP1,MDM2*
GO:0072359	circulatory system development	0.00795889390716741	*CCL24,ASB2,BMP5,VCAM1,DHRS3,NDP,DCHS1,CXCL10,ASB4,FGFBP1,SOX4,ALDH1A2,ANGPT2,PLXNA4,LEFTY1,ERBB4,IL1B,NOX5,DLL1,EGF,PLN,XIRP2,SLC2A10,EYA1,NPR3,MYLK,ANK2,PECAM1,SLC2A12,JCAD,NPR1,DLC1,SHH,ABCC9,SPRY1,RGCC,PDGFB,PITX2,PRKG1,CPE,VASH2,SVEP1,SEMA3E,LEPR,PIK3CG,IL6,ITGB8,FZD1,PTPN6,LRP5,CXCL8,PTPRJ,C3,PDGFA,PGF,HLA-G,HOXA13,SPRED1,AHR,ADGRF5,IL1A,KCNJ8,BICC1,TLR3,ISM1,PRICKLE1,NRP1,APOLD1,MDM2,MSX2,IMMP2L,COL18A1*
GO:0051240	positive regulation of multicellular organismal process	0.008291289907299155	*SPN,CCL24,TP63,VCAM1,TESC,LRRTM2,ZBTB20,SLITRK6,HCLS1,VAV1,ABCA1,ZP3,NTSR1,NCMAP,CYP19A1,GSX2,SOX4,CCL5,ANGPT2,PLXNA4,TRPM8,PRDM16,ERBB4,LRRN3,IL1B,NOX5,DLL1,CADM1,EGF,LIPG,HLA-DPB1,TNFRSF8,TIAM1,LTB,CCR1,PTPRC,PDE5A,LRRN1,LRRK2,P2RY1,C5,PLXNB1,GDF5,JCAD,NPR1,PLCG2,HLA-DPA1,CRTAM,HLA-DOA,C1QTNF1,SHH,PLAAT4,AIM2,SPRY1,TNFSF4,RGCC,EPHB6,PDGFB,MAGED1,HLA-DRB5,FAM20C,INHBB,VASH2,SVEP1,TBXAS1,CMKLR1,LEPR,CARD11,PIK3CG,RBM47,IL6,ITGB8,SORL1,HLA-DRA,PTPN6,CXCL8,PTPRJ,ADRB1,FZD3,C3,PYCARD,ADGRV1,PGF,HLA-G,IL7R,HLA-DRB1,GLIPR2,IL1A,ANO6,PDE9A,ASPM,TLR3,NRP1,IFIH1,HLA-DMB,MSX2*
GO:0042102	positive regulation of T cell proliferation	0.008381739347103689	*SPN,VCAM1,ZP3,CCL5,IL1B,HLA-DPB1,PTPRC,HLA-DPA1,SHH,TNFSF4,CARD11,IL6,PYCARD,IL1A,HLA-DMB*
GO:0002504	antigen processing and presentation of peptide or polysaccharide antigen via MHC class II	0.008459601438288297	*HLA-DPB1,HLA-DPA1,HLA-DOA,HLA-DRB5,CTSS,HLA-DRA,PYCARD,HLA-DRB1,HLA-DMB*
GO:0019886	antigen processing and presentation of exogenous peptide antigen via MHC class II	0.009843784910162518	*HLA-DPB1,HLA-DPA1,HLA-DOA,HLA-DRB5,CTSS,HLA-DRA,HLA-DRB1,HLA-DMB*
GO:0048583	regulation of response to stimulus	0.010522218547113705	*SPN,CCL24,LAIR2,KLRC2,TP63,BMP5,DHRS3,NDP,JAK3,CACNG4,H2BC11,COLEC12,MCF2L,AGR2,MEIS3,SLC30A10,EPPK1,CXCL10,SHISA3,ADAMTS20,F2RL3,NTF3,HCLS1,ARHGAP28,KLRC3,NLRP14,GRIN2B,VAV1,LGR6,LRRC19,ABCA1,ADAMTSL2,ZP3*

			,NTSR1,CYP19A1,FGFBP1,GSX2,SOX4,CCL5,AR,ANGPT2,PLXNA4,APOBEC3G,TEX15,MGMT,DDX60,PRDM16,ERBB4,SUMO4,IL1B,ADRA2C,DLL1,CADM1,EGF,CSF3,SLC2A10,EYA1,HLA-DPB1,ESR1,DUSP9,TRIL,TIAM1,MYLK,PDE3A,RELL1,SLC19A1,LTB,CCR1,PIK3AP1,PTPRC,PDE5A,ALPK1,IL20RA,ARHGAP30,LRRK2,P2RY1,GRK3,C5,KLB,PLXNB1,GDF5,JCAD,NOTCH2NLB,GPR158,NPR1,ENPP1,PLCG2,HLA-DPA1,DLC1,TFPI,CRTAM,C1R,ITGA1,SOX2,GPC6,HLA-DOA,C1QTNF1,TRIM59,SHH,AIM2,SPRY1,TNFSF4,INSIG1,MAP3K5,RGCC,TNFSF10,STMN3,PDGFB,MAGED1,PRKG1,HLA-DRB5,FAM20C,P2RY6,MAP2K6,INHBB,PTCH2,SRGAP3,SVEP1,OASL,SEMA3E,CMKLR1,HIP1,LAMB1,PARP14,KCTD12,CARD11,PIK3CG,GNG7,KREMEN1,RBM47,IL6,DAPK2,CTSS,LFNG,SORL1,CFD,ALOX5AP,FZD1,RASSF2,HLA-DRA,PTPN6,CXCL8,PREX1,PTPRJ,ARHGEF10,ADRB1,GPR55,CYTH4,CGNL1,C3,PDGFA,PYCARD,ADGRV1,PGF,HLA-G,RBMS3,IL7R,HLA-DRB1,GLIPR2,CHN2,SH3TC2,KLHL24,C1S,HOXA13,SPRED1,MDFIC,AHR,UGCG,DIXDC1,IL1A,ADAMTS3,MVB12B,ANO6,HELB,ST6GAL1,PDE9A,DDIT4,KCNJ8,SNX13,ASPM,C2,BICC1,TLR3,NOTCH2NLA,PDE10A,PRICKLE1,NRP1,IFIH1,MDM2,HLA-DMB,APOL3,RGS5,MSX2,KIF14
GO:0006334	nucleosome assembly	0.011892319587260254	H2BC14,H2BC3,H4C2,H2BC1,H3C13,H1-4,H2BC13,H2BC17,H2BC21,H2BC6,H2BC11,H1-6,H2BC15,H1-5,H1-2,ATRX
GO:0051050	positive regulation of transport	0.011976865047683057	KLRC2,ABCG1,TESC,RAPGEF4,CXCL10,FGF13,F2RL3,NTF3,HCLS1,ABCA1,ZP3,NTSR1,CYP19A1,SOX4,ABCB4,CCL5,IL1B,DLL1,EGF,LIPG,SNAP25,MYLK,CCR1,PTPRC,ANK2,EXPH5,LRRK2,P2RY1,ANO1,GPR158,NPR1,SLC4A8,PLCG2,C1QTNF1,SHH,CXCL11,RGCC,PDGFB,P2RY6,MAP2K6,INHBB,ABCA13,HIP1,CADPS2,CTSS,SORL1,PTPRJ,DENND5B,C3,PYCARD,TBC1D5,HLA-DRB1,IL1A,AKAP9,ANO6,C2,MDM2
GO:0050670	regulation of lymphocyte proliferation	0.012801748617689032	SPN,TNFSF18,VCAM1,ZP3,CCL5,LGALS12,TNFRSF9,IL1B,HLA-DPB1,PTPRC,PDE5A,HLA-DPA1,CRTAM,SHH,TNFSF4,CARD11,IL6,PTPN6,PYCARD,HLA-G,HLA-DRB1,AHR,IL1A,HLA-DMB
GO:0050870	positive regulation of T cell activation	0.013654470208218441	SPN,VCAM1,VAV1,ZP3,SOX4,CCL5,IL1B,HLA-DPB1,PTPRC,HLA-DPA1,HLA-DOA,SHH,TNFSF4,EPHB6,HLA-DRB5,CARD11,IL6,HLA-DRA,PTPN6,PYCARD,HLA-G,IL7R,HLA-DRB1,IL1A,HLA-DMB
GO:0002478	antigen processing and presentation of exogenous peptide antigen	0.013725394065688101	HLA-DPB1,HLA-DPA1,HLA-DOA,HLA-DRB5,CTSS,HLA-DRA,HLA-DRB1,LNPEP,HLA-DMB
GO:2000145	regulation of cell motility	0.016764245577051144	SPN,CCL24,TNFSF18,BMP5,EPPK1,CXCL10,NTF3,LGR6,ZP3,CYP19A1,TNFRSF18,FGFBP1,GSX2,CCL5,ANGPT2,PLXNA4,ERBB4,IL1B,EGF,TIAM1,MYLK,SEMA6B,CCR1,PARD6B,PTPRC,PECAM1,C5,PLXNB1,JCAD,PLCG2,DLC1,SHH,SYNE2,RGCC,PDGFB,PR

			KG1,SEMA6D,SPOCK3,P2RY6,SRGAP3,SEMA3E,CMKLR1,LAMB1,PIK3CG,IL6,DAPK2,SORL1,EVL,CXCL8,PREX1,PTPRJ,PDGFA,PYCARD,PGF,GLIPR2,FAT1,SEMA3D,SPRED1,IL1A,ANO6,RAP2B,TTBK2,NRP1,MDM2,KIF14
GO:0035239	tube morphogenesis	0.017887151819113788	*CCL24,ASB2,TP63,BMP5,NDP,DCHS1,AGR2,CXCL10,ASB4,FGFBP1,SOX4,AR,ANGPT2,IL1B,NOX5,DLL1,EGF,EYA1,ESR1,PBX1,NPR3,MYLK,JCAD,NPR1,DLC1,SHH,SPRY1,RGCC,MAGED1,IRX2,VASH2,SEMA3E,LEPR,PIK3CG,IL6,ITGB8,PTPN6,LRP5,CXCL8,PTPRJ,IRX1,FZD3,C3,PDGFA,PGF,HLA-G,HOXA13,CELSR1,SPRED1,ADGRF5,IL1A,TLR3,ISM1,PRICKLE1,NRP1,APOLD1,MSX2,COL18A1*
GO:0010646	regulation of cell communication	0.018143415191037418	*LZTS1,TP63,BMP5,DHRS3,NDP,JAK3,CACNG4,H2BC11,MCF2L,AGR2,LRRTM2,MEIS3,SLC30A10,RAPGEF4,SHISA3,ADAMTS20,F2RL3,NTF3,HCLS1,ARHGAP28,GRIN2B,VAV1,LGR6,LRRC19,ABCA1,ADAMTSL2,GNAZ,NTSR1,CYP19A1,FGFBP1,GSX2,SOX4,CCL5,AR,FER1L5,DDX60,PRDM16,ERBB4,SUMO4,IL1B,ADRA2C,CPLX1,DLL1,CADM1,EGF,ZBED6,PLCB4,CSF3,SLC2A10,EYA1,SNAP25,ESR1,STXBP5L,DUSP9,TIAM1,PDE3A,RELL1,SLC19A1,LTB,CCR1,PIK3AP1,PTPRC,ANK2,PDE5A,ALPK1,IL20RA,ARHGAP30,LRRK2,P2RY1,GRK3,KLB,PLXNB1,GDF5,JCAD,UNC13A,NOTCH2NLB,ANO1,GPR158,NPR1,SLC4A8,ENPP1,PLCG2,DLC1,ITGA1,SOX2,GPC6,C1QTNF1,TRIM59,SHH,GRIK4,AIM2,SPRY1,INSIG1,MAP3K5,TNFSF10,STMN3,PDGFB,MAGED1,FAM20C,P2RY6,MAP2K6,INHBB,PTCH2,SRGAP3,OASL,SEMA3E,CMKLR1,BEST1,HIP1,LAMB1,PARP14,KCTD12,CARD11,PIK3CG,GNG7,KREMEN1,RBM47,IL6,DAPK2,SORCS2,CTSS,LFNG,SORL1,FZD1,RASSF2,PTPN6,LRP5,CXCL8,PREX1,PPP1R9A,PTPRJ,ARHGEF10,ADRB1,GPR55,CYTH4,CGNL1,C3,PDGFA,PYCARD,ADGRV1,HLA-G,RBMS3,IL7R,HLA-DRB1,GLIPR2,CHN2,SH3TC2,KLHL24,APBA2,HOXA13,SPRED1,MDFIC,UGCG,DIXDC1,IL1A,ADAMTS3,MVB12B,PDE9A,DDIT4,SNX13,ASPM,BICC1,TLR3,NOTCH2NLA,PDE10A,PRICKLE1,NRP1,MDM2,APOL3,RGS5,MSX2,KIF14*
GO:0040012	regulation of locomotion	0.018382713328468147	*SPN,CCL24,TNFSF18,BMP5,EPPK1,CXCL10,NTF3,LGR6,ZP3,CYP19A1,TNFRSF18,FGFBP1,GSX2,CCL5,ANGPT2,PLXNA4,ERBB4,IL1B,EGF,TIAM1,MYLK,SEMA6B,CCR1,PARD6B,PTPRC,PECAM1,LRRK2,C5,PLXNB1,JCAD,PLCG2,DLC1,SHH,SYNE2,RGCC,PDGFB,PRKG1,SEMA6D,SPOCK3,P2RY6,SRGAP3,SEMA3E,CMKLR1,LAMB1,PIK3CG,IL6,DAPK2,SORL1,EVL,CXCL8,PREX1,PTPRJ,PDGFA,PYCARD,PGF,GLIPR2,FAT1,SEMA3D,SPRED1,IL1A,ANO6,RAP2B,ST6GAL1,TTBK2,NRP1,MDM2,KIF14*
GO:0032103	positive regulation of response to external stimulus	0.020253450550170464	*CCL24,KLRC2,COLEC12,CXCL10,NTF3,KLRC3,VAV1,LRRC19,ZP3,CCL5,DDX60,IL1B,CADM1,ESR1,TRIL,TIAM1,SLC19A1,CCR1,PIK3AP1,ALPK1,LRRK2,PLCG2,CRTAM,AIM2,TNFSF4,PDGFB,MAP2K6,OASL,CMKLR1,PIK3CG,RBM47,IL6,DAPK2,CTSS,ALOX5AP,CXCL8,PTPRJ,C3,PYCARD,PGF,HLA-G,ANO6,KCNJ8,TLR3,NRP1,IFIH1*
GO:0001944	vasculature development	0.02111814055266702	*CCL24,NDP,CXCL10,ASB4,FGFBP1,SOX4,ALDH1A2,ANGPT2,IL1B,NOX5,DLL1,EGF,SLC2A10,EYA1,NPR3,MYLK,PECAM1,JCAD,NPR1,SHH,ABCC9,RGCC,PDGFB,VASH2,SVEP1,SEMA3E,LEPR,PIK3CG,IL6,ITGB8,PTPN6,LRP5,CXCL8,PTPRJ,C3,PDGFA,PGF,HLA-G,HOXA13,SPRED1,AHR,ADGRF5,IL1A,KCNJ8,TLR*

			3,ISM1,PRICKLE1,NRP1,APOLD1,MDM2,IMMP2L,COL18A1
GO:0048513	animal organ development	0.021939593468835294	*ASB2,KRT83,TP63,BMP5,HSD3B1,VCAM1,DHRS3,NDP,BHLHA15,ELF3,DCHS1,AGR2,OGN,MEIS3,RP1L1,CXCL10,FGF13,BCL11B,SLITRK6,GRIN2B,ADAMTSL2,ZP3,CYP19A1,DMC1,H1-5,GSX2,SOX4,ALDH1A2,AR,ANGPT2,MEGF10,PLXNA4,PPL,LEFTY1,ERBB4,IL1B,DLL1,CADM1,EGF,MMP17,PLN,XIRP2,USH2A,SLC2A10,EYA1,GPM6A,GCNT4,ESR1,PBX1,ITGB6,TIAM1,MYLK,TMT1A,SEMA6B,LTB,CCR1,PTPRC,SPEF2,ANK2,PECAM1,EXPH5,FAM20A,LRRK2,PLXNB1,GDF5,NOTCH2NLB,GPR158,KRT81,RADIL,ENPP1,IRX5,DLC1,KRT86,SOX2,LCP1,SHH,RORB,PLAAT4,SYNE2,AIM2,ABCC9,SPRY1,INSIG1,RGCC,NR5A2,TNFSF10,PDGFB,MAGED1,PITX2,PRKG1,FOXP2,SEMA6D,CPE,FAM20C,MAP2K6,INHBB,PTCH2,IRX2,VASH2,SEMA3E,LEPR,ETV1,LAMB1,GJB2,IL6,ITGB8,LFNG,SORL1,FZD1,LRP5,EVL,CXCL8,FLG,PTPRJ,IRX1,LRIG1,TMEM135,FZD3,PDGFA,CEBPD,ILDR2,ATRX,ADGRV1,ITGB4,IL7R,GLIPR2,MYO16,ZNF430,FAT1,NNMT,SVIL,SEMA3D,HOXA13,CELSR1,SPRED1,UGCG,DIXDC1,IL1A,ANO6,MMP16,DDIT4,KCNJ8,ASPM,BICC1,SLC40A1,TLR3,TTBK2,NOTCH2NLA,PRICKLE1,NRP1,MDM2,MSX2,KIF14,IMMP2L,COL18A1*
GO:2000147	positive regulation of cell motility	0.023090656917972876	*SPN,CCL24,TNFSF18,CXCL10,NTF3,LGR6,ZP3,TNFRSF18,FGFBP1,CCL5,IL1B,EGF,TIAM1,MYLK,SEMA6B,CCR1,PTPRC,PECAM1,JCAD,PLCG2,SYNE2,PDGFB,SEMA6D,P2RY6,SEMA3E,CMKLR1,LAMB1,PIK3CG,IL6,DAPK2,CXCL8,PREX1,PTPRJ,PDGFA,PYCARD,PGF,GLIPR2,FAT1,SEMA3D,IL1A,ANO6,NRP1,MDM2*
GO:0023051	regulation of signaling	0.023927256379179904	*LZTS1,TP63,BMP5,DHRS3,NDP,JAK3,CACNG4,H2BC11,MCF2L,AGR2,LRRTM2,MEIS3,SLC30A10,RAPGEF4,SHISA3,ADAMTS20,F2RL3,NTF3,HCLS1,ARHGAP28,GRIN2B,VAV1,LGR6,LRRC19,ABCA1,ADAMTSL2,GNAZ,NTSR1,CYP19A1,FGFBP1,GSX2,SOX4,CCL5,AR,FER1L5,DDX60,PRDM16,ERBB4,SUMO4,IL1B,ADRA2C,CPLX1,DLL1,CADM1,EGF,ZBED6,PLCB4,CSF3,SLC2A10,EYA1,SNAP25,ESR1,STXBP5L,DUSP9,TIAM1,PDE3A,RELL1,SLC19A1,LTB,CCR1,PIK3AP1,PTPRC,PDE5A,ALPK1,IL20RA,ARHGAP30,LRRK2,P2RY1,GRK3,KLB,PLXNB1,GDF5,JCAD,UNC13A,NOTCH2NLB,ANO1,GPR158,NPR1,SLC4A8,ENPP1,PLCG2,DLC1,ITGA1,SOX2,GPC6,C1QTNF1,TRIM59,SHH,GRIK4,AIM2,SPRY1,INSIG1,MAP3K5,TNFSF10,STMN3,PDGFB,MAGED1,FAM20C,P2RY6,MAP2K6,INHBB,PTCH2,SRGAP3,OASL,SEMA3E,CMKLR1,BEST1,HIP1,LAMB1,PARP14,KCTD12,CARD11,PIK3CG,GNG7,KREMEN1,RBM47,IL6,DAPK2,SORCS2,CTSS,LFNG,SORL1,FZD1,RASSF2,PTPN6,LRP5,CXCL8,PREX1,PPP1R9A,PTPRJ,ARHGEF10,ADRB1,GPR55,CYTH4,CGNL1,C3,PDGFA,PYCARD,ADGRV1,HLA-G,RBMS3,IL7R,HLA-DRB1,GLIPR2,CHN2,SH3TC2,KLHL24,APBA2,HOXA13,SPRED1,MDFIC,UGCG,DIXDC1,IL1A,ADAMTS3,MVB12B,PDE9A,DDIT4,SNX13,ASPM,BICC1,TLR3,NOTCH2NLA,PDE10A,PRICKLE1,NRP1,MDM2,APOL3,RGS5,MSX2,KIF14*
GO:0048468	cell development	0.02488115129730784	*H4C2,H2BC1,SPN,LZTS1,ASB2,TP63,BMP5,ADGRF1,VCAM1,NDP,JAK3,BHLHA15,GLDN,TESC,RP1L1,TENM2,FGF13,BCL11B,SLITRK6,NTF3,HCLS1,VAV1,LGR6,SPANXB1,ZP3,NCMAP,ATP9A,C1QL1,DMC1,KIF5C,GSX2,WDR38,SOX4,ALDH1A2,AR,ANGPT2,*

			MEGF10,PLXNA4,TNFRSF9,ERBB4,IL1B,DLL1,PLN,CSF3,GPM6A,SNAP25,ESR1,PBX1,FSIP2,ITGB6,TIAM1,PDE3A,ULK2,SEMA6B,CCR1,PARD6B,PTPRC,SPEF2,ANK2,PDE5A,PECAM1,EXPH5,LRRK2,PLXNB1,MAEL,UNC13A,ANO1,GPRIN3,RADIL,PLCG2,IRX5,CRTAM,ITGA1,HLA-DOA,SHH,RORB,SPRY1,TNFSF4,STMN3,PDGFB,PITX2,PRKG1,SEMA6D,FAM20C,NEK3,INHBB,IRX2,VASH2,SEMA3E,LEPR,ETV1,LAMB1,CARD11,CLMN,KREMEN1,RBM47,GPRIN2,IL6,ITGB8,LFNG,SORL1,FZD1,RASSF2,HLA-DRA,PTPN6,LRP5,UST,PREX1,PPP1R9A,PTPRJ,IRX1,ARHGEF10,GPR55,DIAPH2,FZD3,C3,IRF2BP2,CEBPD,ATRX,ADGRV1,HLA-G,ITGB4,PAQR8,IL7R,HLA-DRB1,MYO16,SH3TC2,FAT1,HLTF,SEMA3D,HOXA13,UGCG,ADGRF5,IL1A,CRPPA,SETX,ASPM,TLR3,PRICKLE1,NRP1,VSIG1,SYT2,MDM2,MSX2,KIF14,COL18A1
GO:0002684	positive regulation of immune system process	0.025425617417496615	*SPN,CCL24,KLRC2,TNFSF18,VCAM1,COLEC12,TESC,CXCL10,HCLS1,KLRC3,VAV1,LRRC19,ZP3,TNFRSF18,SOX4,CCL5,DDX60,IL1B,CADM1,HLA-DPB1,ESR1,TRIL,SLC19A1,CCR1,PIK3AP1,PTPRC,ALPK1,LRRK2,C5,PLCG2,HLA-DPA1,CRTAM,C1R,HLA-DOA,SHH,AIM2,TNFSF4,RGCC,EPHB6,HLA-DRB5,MAP2K6,SVEP1,OASL,CMKLR1,CARD11,RBM47,IL6,DAPK2,CTSS,CFD,HLA-DRA,PTPN6,CXCL8,PTPRJ,C3,PYCARD,PGF,HLA-G,IL7R,HLA-DRB1,C1S,IL1A,ANO6,KCNJ8,C2,TLR3,IFIH1,HLA-DMB*
GO:0009653	anatomical structure morphogenesis	0.026743653809240354	*LZTS1,CCL24,ASB2,TP63,BMP5,DHRS3,NDP,ELF3,DCHS1,AGR2,MEIS3,CXCL10,FGF13,BCL11B,SLITRK6,NTF3,ASB4,LGR6,ZP3,NCMAP,ATP9A,CYP19A1,CST6,FGFBP1,KIF5C,GSX2,SOX4,ALDH1A2,AR,ANGPT2,PLXNA4,LEFTY1,ERBB4,IL1B,NOX5,DLL1,EGF,PLN,XIRP2,EYA1,GPM6A,SNAP25,GCNT4,ESR1,PBX1,ITGB6,NPR3,MPZL2,TIAM1,MYLK,TMT1A,ULK2,SEMA6B,PARD6B,SPEF2,ANK2,FAM20A,LRRK2,P2RY1,PLXNB1,GDF5,JCAD,MAEL,UNC13A,ANO1,NPR1,IRX5,DLC1,ITGA1,SOX2,SHH,RORB,ABCC9,SPRY1,INSIG1,RGCC,NR5A2,DLG3,MAGED1,PITX2,PRKG1,SEMA6D,CPE,FAM20C,NEK3,IRX2,VASH2,SVEP1,SEMA3E,LEPR,ETV1,LAMB1,PIK3CG,IL6,ITGB8,LFNG,FZD1,PTPN6,LRP5,EVL,UST,CXCL8,PTPRJ,IRX1,LRIG1,TMEM135,LIN7C,FZD3,C3,PDGFA,ATRX,PGF,HLA-G,ITGB4,IL7R,MYO16,FAT1,COCH,SEMA3D,HOXA13,CELSR1,SPRED1,ADGRF5,IL1A,MMP16,CRPPA,KCNJ8,SLC40A1,TLR3,ISM1,PRICKLE1,NRP1,APOLD1,VSIG1,SYT2,MDM2,MSX2,KIF14,COL18A1*
GO:0006959	humoral immune response	0.027222730349835217	*CCL24,H2BC21,H2BC6,H2BC11,CXCL10,ZP3,CCL5,IL1B,PRSS2,PTPRC,C5,C1R,CXCL11,RGCC,SVEP1,IL6,CFD,PTPN6,CXCL8,DMBT1,C3,LYZ,HLA-DRB1,C1S,ST6GAL1,C2*
GO:0050863	regulation of T cell activation	0.02956231185970191	*SPN,TNFSF18,VCAM1,JAK3,VAV1,ZP3,SOX4,CCL5,LGALS12,TNFRSF9,IL1B,HLA-DPB1,PTPRC,PDE5A,HLA-DPA1,CRTAM,HLA-DOA,SHH,TNFSF4,EPHB6,HLA-DRB5,CARD11,IL6,HLA-DRA,PTPN6,ILDR2,PYCARD,HLA-G,IL7R,HLA-DRB1,IL1A,HLA-DMB*

GO:0001568	blood vessel development	0.030514477965519336	*CCL24,NDP,CXCL10,ASB4,FGFBP1,SOX4,ALDH1A2,ANGPT2,IL1B,NOX5,DLL1,EGF,SLC2A10,EYA1,NPR3,MYLK,PECAM1,JCAD,NPR1,SHH,ABCC9,RGCC,PDGFB,VASH2,SEMA3E,LEPR,PIK3CG,IL6,ITGB8,PTPN6,LRP5,CXCL8,PTPRJ,C3,PDGFA,PGF,HLA-G,HOXA13,SPRED1,AHR,ADGRF5,IL1A,KCNJ8,TLR3,ISM1,PRICKLE1,NRP1,APOLD1,MDM2,COL18A1*
GO:0070663	regulation of leukocyte proliferation	0.032439775532780976	*SPN,TNFSF18,VCAM1,ZP3,CCL5,LGALS12,TNFRSF9,IL1B,HLA-DPB1,PTPRC,PDE5A,HLA-DPA1,CRTAM,SHH,TNFSF4,CARD11,IL6,PTPN6,PYCARD,HLA-G,HLA-DRB1,AHR,IL1A,ST6GAL1,HLA-DMB*
GO:0048870	cell motility	0.03347750296571937	*H2BC1,SPN,CCL24,ASB2,TNFSF18,BMP5,VCAM1,DCHS1,EPPK1,CXCL10,FGF13,BCL11B,NTF3,ZG16B,VAV1,LGR6,ZP3,S100P,CYP19A1,TNFRSF18,FGFBP1,GSX2,CCL5,ANGPT2,MEGF10,PLXNA4,ERBB4,IL1B,EGF,GPM6A,FSIP2,TIAM1,MYLK,SEMA6B,DNAH7,TMEM232,CCR1,PARD6B,PTPRC,SPEF2,PECAM1,LRRK2,P2RY1,C5,PLXNB1,JCAD,RADIL,PLCG2,DLC1,CRTAM,ITGA1,GPC6,LCP1,SHH,CXCL11,SYNE2,RGCC,PDGFB,PITX2,PRKG1,SEMA6D,SPOCK3,P2RY6,SRGAP3,SEMA3E,CMKLR1,LAMB1,PIK3CG,IL6,DAPK2,SORL1,LRP5,EVL,CXCL8,PREX1,PTPRJ,FZD3,PDGFA,PYCARD,PGF,ITGB4,GLIPR2,FAT1,SEMA3D,CELSR1,SPRED1,DIXDC1,IL1A,ANO6,RAP2B,DDIT4,ASPM,TTBK2,DNAH3,PRICKLE1,NRP1,MDM2,MSX2,KIF14*
GO:0032943	mononuclear cell proliferation	0.03422090477277647	*SPN,TNFSF18,VCAM1,ZP3,CCL5,LGALS12,TNFRSF9,IL1B,HLA-DPB1,PTPRC,PDE5A,HLA-DPA1,CRTAM,SHH,TNFSF4,EPHB6,CARD11,PIK3CG,IL6,PTPN6,PYCARD,HLA-G,IL7R,HLA-DRB1,AHR,IL1A,ST6GAL1,HLA-DMB*
GO:0070661	leukocyte proliferation	0.0359764196864644	*SPN,TNFSF18,VCAM1,NDP,ZP3,CCL5,LGALS12,TNFRSF9,IL1B,HLA-DPB1,NPR3,PTPRC,PDE5A,HLA-DPA1,CRTAM,SHH,TNFSF4,EPHB6,CARD11,PIK3CG,IL6,PTPN6,PYCARD,HLA-G,IL7R,HLA-DRB1,AHR,IL1A,ST6GAL1,HLA-DMB*
GO:0045937	positive regulation of phosphate metabolic process	0.03733847676025164	*TNFSF18,ZBTB20,NTF3,HCLS1,NTSR1,TNFRSF18,CCL5,AR,CCND2,ERBB4,IL1B,ADRA2C,EGF,CSF3,PTPRC,PECAM1,FAM20A,LRRK2,P2RY1,PLCG2,MAP3K5,RGCC,DLG3,PDGFB,P2RY6,LEPR,PARP14,PIK3CG,IL6,RASSF2,PTPRJ,C3,PDGFA,PGF,HLA-DRB1,ADGRF5,AKAP9,RAP2B,TLR3,NRP1,KIF14*
GO:0010562	positive regulation of phosphorus metabolic process	0.03733847676025164	*TNFSF18,ZBTB20,NTF3,HCLS1,NTSR1,TNFRSF18,CCL5,AR,CCND2,ERBB4,IL1B,ADRA2C,EGF,CSF3,PTPRC,PECAM1,FAM20A,LRRK2,P2RY1,PLCG2,MAP3K5,RGCC,DLG3,PDGFB,P2RY6,LEPR,PARP14,PIK3CG,IL6,RASSF2,PTPRJ,C3,PDGFA,PGF,HLA-DRB1,ADGRF5,AKAP9,RAP2B,TLR3,NRP1,KIF14*
GO:0009967	positive regulation of signal transduction	0.04279805507310348	*TP63,BMP5,NDP,MCF2L,AGR2,MEIS3,SLC30A10,ADAMTS20,F2RL3,NTF3,HCLS1,GRIN2B,LGR6,LRRC19,FGFBP1,GSX2,SOX4,CCL5,AR,DDX60,ERBB4,IL1B,ADRA2C,DLL1,EGF,CSF3,SLC2A10,RELL1,SLC19A1,LTB,CCR1,PIK3AP1,PTPRC,ALPK1,IL20RA,LRRK2,P2RY1,KLB,PLXNB1,GDF5,JCAD,NOTCH2NLB,NPR1,PLCG2,ITGA1,SOX2,C1QTNF1,SHH,MAP3K5,TNFSF10,PDGFB,MAGED1,P2RY6,MAP2K6,INHBB,OASL,SEMA3E,HIP1,LAMB1,PARP14,CARD11,PIK3CG,RBM47,IL6,CTSS,LFNG,SORL1,RASSF2,PTPN6,PTPRJ,ADRB1,GPR55,C3,PDGFA,PYCARD,ADGRV1,IL7R,HLA-DRB1,GLIPR2,SPRED1,DIXDC1,IL1A,ADAMTS3,ASPM,TLR3,NOTCH2NLA,NRP1,APOL3,MSX2*

GO:0040017	positive regulation of locomotion	0.044177731604512945	*SPN,CCL24,TNFSF18,CXCL10,NTF3,LGR6,ZP3,TNFRSF18,FGFBP1,CCL5,IL1B,EGF,TIAM1,MYLK,SEMA6B,CCR1,PTPRC,PECAM1,JCAD,PLCG2,SYNE2,PDGFB,SEMA6D,P2RY6,SEMA3E,CMKLR1,LAMB1,PIK3CG,IL6,DAPK2,CXCL8,PREX1,PTPRJ,PDGFA,PYCARD,PGF,GLIPR2,FAT1,SEMA3D,IL1A,ANO6,NRP1,MDM2*
GO:0045785	positive regulation of cell adhesion	0.04500886617461261	*SPN,TNFSF18,VCAM1,AGR2,VAV1,ZP3,TNFRSF18,SOX4,CCL5,MEGF10,IL1B,ECM2,PRSS2,HLA-DPB1,PTPRC,HLA-DPA1,SOX2,HLA-DOA,SHH,TNFSF4,EPHB6,PDGFB,HLA-DRB5,LAMB1,CARD11,IL6,HLA-DRA,PTPN6,PREX1,PTPRJ,PYCARD,HLA-G,IL7R,HLA-DRB1,IL1A,NRP1,HLA-DMB*
GO:0051049	regulation of transport	0.04992589702311115	*PTPRN2,KLRC2,ABCG1,CACNG4,TESC,LRRTM2,RAPGEF4,CXCL10,FGF13,F2RL3,NTF3,HCLS1,KCNJ13,GRIN2B,ABCA1,ZP3,GNAZ,NTSR1,ATP9A,CYP19A1,SOX4,ABCB4,CCL5,FER1L5,IL1B,ADRA2C,CPLX1,DLL1,EGF,ZBED6,LIPG,PLN,SNAP25,STXBP5L,TIAM1,MYLK,CCR1,PTPRC,ANK2,EXPH5,LRRK2,P2RY1,ANO1,GPR158,NPR1,SLC4A8,ENPP1,PLCG2,LCP1,C1QTNF1,SHH,CXCL11,SNAP91,ABCC9,INSIG1,RGCC,PDGFB,PRKG1,P2RY6,MAP2K6,INHBB,ABCA13,BEST1,HIP1,LEPR,PIK3CG,CADPS2,IL6,CTSS,SORL1,PTPN6,LRP5,PTPRJ,DENND5B,C3,PYCARD,TBC1D5,HLA-DRB1,SH3TC2,KLHL24,MDFIC,IL1A,AKAP9,ANO6,MX2,KCNJ8,C2,TTC39B,NRP1,MDM2*
CC			
GO:0071944	cell periphery	1.443958035364971e-13	*GNG2,SPN,LZTS1,F13A1,PTPRN2,SERPINA5,PCDHGA8,LAIR2,KLRC2,GPRC5C,TNFSF18,ADGRF1,SLCO2A1,VCAM1,DHRS3,NDP,JAK3,ABCG1,ALDH3A1,CACNG4,GLDN,H2BC11,COLEC12,MUC5AC,SLC2A14,TAS2R30,TESC,DCHS1,MCF2L,PCDHGA11,LRRTM2,OGN,SCN3A,PCDHGB7,SLC16A14,PCDHGB5,TRPM3,SLC30A10,RAPGEF4,SERPINA1,PROKR1,TENM2,SLC10A1,EPPK1,CXCL10,FGF13,LAMP3,ADAMTS20,RAB39A,STEAP4,F2RL3,SLITRK6,HCLS1,KLRC3,RAB37,ZG16B,SCARA5,KCNJ13,GRIN2B,VAV1,LGR6,LRRC19,KCNT2,ABCA1,ADAMTSL2,GYPC,ZP3,GNAZ,NTSR1,NCMAP,S100P,ATP9A,CST6,TNFRSF18,SNED1,TAS2R13,FGFBP1,THSD7B,MUC4,ABCB4,AR,ANGPT2,FER1L5,MEGF10,PLXNA4,PCDHGA2,PPL,SSPN,COLQ,TNFRSF9,TRPM8,PCDHGB4,ENSG00000293570,ERBB4,LRRN3,TRHR,ADRA2C,NOX5,DLL1,CADM1,SLC13A4,ECM2,EGF,PCDHGA12,MMP17,PCDHGA10,CLDN11,PLCB4,SLC9A9,USH2A,PCDHGA4,TMPRSS2,SLC2A10,COL6A2,SLCO4C1,GPR176,GPM6A,SNAP25,PRSS2,SLC16A6,PCDHB12,HLA-DPB1,GASK1A,GLIPR1L1,TNFRSF8,SLC47A1,ESR1,STXBP5L,ITGB6,NPR3,TRIL,KCNH3,TSPAN11,MPZL2,OMD,TIAM1,MYLK,LRP1B,ADAMTS14,RELL1,SEMA6B,SLC19A1,LTB,CCR1,PIK3AP1,PARD6B,PTPRC,ABCC6,ANK2,ITGBL1,PECAM1,PCDH7,IL20RA,LRRN1,LRRK2,PCDHGB6,P2RY1,SLC2A12,GRK3,C5,ADAM28,KLB,PLXNB1,GDF5,JCAD,UNC13A,GPR162,ANO1,PCDHGB3,GPRIN3,GPR158,NPR1,MUC5B,LIFR,SLC4A8,ENPP1,PLCG2,HLA-DPA1,PCDHB11,DLC1,TFPI,SLCO1B1,CRTAM,GRAMD1C,TSPAN33,RAB40A,ITGA1,GPC6,HLA-DOA,GPR85,LCP1,C1QTNF1,SHH,GRIK4,SNAP91,MAP7,QRICH1,SYNE2,ABCC9,SPRY1,TNFSF4,MAP3K5,DLG3,TNFSF10,EPHB6,PDGFB,MAGED1,PRKG1,SEMA6D,PCDHGA9,CPE,SPOCK3,HLA-*

			DRB5,TCN2,P2RY6,C10ORF90,ITGA10,INHBB,PTCH2,ABCA13,TGM5,SLC14A1,PCDHB10,FLVCR2,SEMA3E,CMKLR1,BEST1,CLIC5,HIP1,LEPR,STEAP2,LAMB1,PARP14,KCTD12,CARD11,PIK3CG,TMEM150C,GNG7,KREMEN1,PCDHGB1,GJB2,GPRIN2,IL6,ITGB8,TAS2R20,SORCS2,CTSS,SORL1,CALHM5,ITPR2,FZD1,RASSF2,HLA-DRA,CPNE7,PTPN6,CD163L1,GCA,TNFRSF10C,SLC1A7,LRP5,PREX1,PPP1R9A,FLG,PCDHGA1,PTPRJ,LRIG1,LIN7C,ADRB1,DMBT1,SLC39A11,PHACTR2,GPR55,CYTH4,PCDHGA5,FZD3,COL4A5,TRBV13,C3,TBC1D5,PCDHGA7,ADGRV1,HLA-G,ITGB4,BTC,PAQR8,IL7R,HLA-DRB1,SLC9A7,PCDHGA6,GPR153,MYO16,SH3TC2,IGSF9B,FAT1,COCH,HLTF,GMFG,SVIL,APBA2,SEMA3D,CELSR1,SPRED1,CALHM3,MDFIC,LNPEP,CLIP2,ADGRF5,ADAMTS3,AKAP9,MVB12B,ANO6,PCDHB8,RAP2B,PDE9A,MMP16,CLDN23,KCNJ8,LANCL1,ASPM,CA14,SLC40A1,TLR3,SYNJ2,GBP4,NRP1,APOLD1,VSIG1,SYT2,MDM2,HLA-DMB,RGS5,CA12,KIF14,COL18A1
GO:0005886	plasma membrane	1.414100047066491e-11	*GNG2,SPN,LZTS1,PTPRN2,SERPINA5,PCDHGA8,LAIR2,KLRC2,GPRC5C,TNFSF18,ADGRF1,SLCO2A1,VCAM1,DHRS3,JAK3,ABCG1,ALDH3A1,CACNG4,GLDN,H2BC11,COLEC12,MUC5AC,SLC2A14,TAS2R30,TESC,DCHS1,MCF2L,PCDHGA11,LRRTM2,SCN3A,PCDHGB7,SLC16A14,PCDHGB5,TRPM3,SLC30A10,RAPGEF4,PROKR1,TENM2,SLC10A1,EPPK1,CXCL10,FGF13,LAMP3,RAB39A,STEAP4,F2RL3,SLITRK6,HCLS1,KLRC3,RAB37,ZG16B,SCARA5,KCNJ13,GRIN2B,VAV1,LGR6,LRRC19,KCNT2,ABCA1,GYPC,ZP3,GNAZ,NTSR1,NCMAP,S100P,ATP9A,CST6,TNFRSF18,TAS2R13,FGFBP1,THSD7B,MUC4,ABCB4,AR,FER1L5,MEGF10,PLXNA4,PCDHGA2,PPL,SSPN,COLQ,TNFRSF9,TRPM8,PCDHGB4,ENSG00000293570,ERBB4,TRHR,ADRA2C,NOX5,DLL1,CADM1,SLC13A4,EGF,PCDHGA12,MMP17,PCDHGA10,CLDN11,PLCB4,SLC9A9,USH2A,PCDHGA4,TMPRSS2,SLC2A10,COL6A2,SLCO4C1,GPR176,GPM6A,SNAP25,SLC16A6,PCDHB12,HLA-DPB1,GASK1A,GLIPR1L1,TNFRSF8,SLC47A1,ESR1,STXBP5L,ITGB6,NPR3,KCNH3,TSPAN11,MPZL2,TIAM1,MYLK,LRP1B,RELL1,SEMA6B,SLC19A1,LTB,CCR1,PIK3AP1,PARD6B,PTPRC,ABCC6,ANK2,ITGBL1,PECAM1,PCDH7,IL20RA,LRRK2,PCDHGB6,P2RY1,SLC2A12,GRK3,C5,ADAM28,KLB,PLXNB1,GDF5,JCAD,UNC13A,GPR162,ANO1,PCDHGB3,GPRIN3,GPR158,NPR1,MUC5B,LIFR,SLC4A8,ENPP1,PLCG2,HLA-DPA1,PCDHB11,DLC1,TFPI,SLCO1B1,CRTAM,GRAMD1C,TSPAN33,RAB40A,ITGA1,GPC6,HLA-DOA,GPR85,LCP1,C1QTNF1,SHH,GRIK4,SNAP91,MAP7,QRICH1,SYNE2,ABCC9,SPRY1,TNFSF4,MAP3K5,DLG3,TNFSF10,EPHB6,PDGFB,MAGED1,PRKG1,SEMA6D,PCDHGA9,CPE,HLA-DRB5,TCN2,P2RY6,C10ORF90,ITGA10,PTCH2,ABCA13,TGM5,SLC14A1,PCDHB10,FLVCR2,SEMA3E,CMKLR1,BEST1,CLIC5,HIP1,LEPR,STEAP2,PARP14,KCTD12,CARD11,PIK3CG,TMEM150C,GNG7,KREMEN1,PCDHGB1,GJB2,GPRIN2,IL6,ITGB8,TAS2R20,SORCS2,SORL1,CALHM5,ITPR2,FZD1,RASSF2,HLA-DRA,CPNE7,PTPN6,CD163L1,GCA,TNFRSF10C,SLC1A7,LRP5,PREX1,FLG,PCDHGA1,PTPRJ,LRIG1,LIN7C,ADRB1,SLC39A11,PHACTR2,GPR55,CYTH4,PC*

			DHGA5,FZD3,TRBV13,C3,TBC1D5,PCDHGA7,ADGRV1,HLA-G,ITGB4,BTC,PAQR8,IL7R,HLA-DRB1,SLC9A7,PCDHGA6,GPR153,MYO16,SH3TC2,IGSF9B,FAT1,HLTF,SVIL,APBA2,SEMA3D,CELSR1,SPRED1,CALHM3,MDFIC,LNPEP,ADGRF5,AKAP9,MVB12B,ANO6,PCDHB8,RAP2B,PDE9A,MMP16,CLDN23,KCNJ8,LANCL1,ASPM,CA14,SLC40A1,TLR3,SYNJ2,GBP4,NRP1,APOLD1,VSIG1,SYT2,MDM2,HLA-DMB,RGS5,CA12,KIF14
GO:0000786	nucleosome	4.520346169630749e-8	*H2AC21,H2BC14,H2AC15,H2BC3,H4C2,H2BC1,H3C13,H2AC12,H1-4,H2AC20,H2BC13,H2BC17,H2BC18,H2AC14,H2AC1,H2BC21,H2BC6,H2BC11,H1-6,H2BC15,H1-5,H2BC5,H1-2,H2AC6*
GO:0009986	cell surface	0.00002412583607112 4225	*H2BC1,SPN,SERPINA5,KLRC2,TNFSF18,VCAM1,NDP,ABCG1,CACNG4,GLDN,CXCL10,LAMP3,SLITRK6,KLRC3,SCARA5,GRIN2B,ABCA1,NTSR1,TNFRSF18,FGFBP1,HYAL4,TNFRSF9,TRPM8,LIPG,HLA-DPB1,ITGB6,CCR1,PTPRC,ITGBL1,PECAM1,P2RY1,LIFR,ENPP1,HLA-DPA1,TFPI,TSPAN33,ITGA1,GPC6,SHH,TNFSF4,MAP3K5,EPHB6,PDGFB,SEMA6D,TCN2,ITGA10,LEPR,ITGB8,SORL1,FZD1,HLA-DRA,CD163L1,TNFRSF10C,PTPRJ,FZD3,C3,PDGFA,ADGRV1,HLA-G,ITGB4,IL7R,HLA-DRB1,ADGRF5,IL1A,ANO6,MMP16*
GO:0016323	basolateral plasma membrane	0.0000275760314492529 8	*SLCO2A1,SLC10A1,EPPK1,ABCA1,ERBB4,CADM1,SLCO4C1,SLC16A6,SLC47A1,SLC19A1,ABCC6,ANK2,P2RY1,SLC4A8,ENPP1,SLCO1B1,MAP7,DLG3,PDGFB,P2RY6,SLC14A1,BEST1,LEPR,LIN7C,CALHM3,CA14,SLC40A1,VSIG1,CA12*
GO:0005576	extracellular region	0.0000391835009815407 24	*H2AC21,H2BC14,H2AC15,GNG2,H4C2,H3C13,H2AC12,H2AC20,H2BC13,SPN,H2BC18,F13A1,H2AC14,SERPINA5,H2AC1,CCL24,H2BC21,H2BC6,LAIR2,GPRC5C,TNFSF18,KRT83,BMP5,RASSF9,ADGRF1,VCAM1,NDP,ALDH3A1,GLDN,H2BC11,COLEC12,MUC5AC,SCRG1,MCF2L,AGR2,LRRTM2,OGN,PCDHGB5,H2BC15,SERPINA1,CXCL10,CHI3L2,ADAMTS20,STEAP4,F2RL3,NTF3,ZG16B,ADAMTSL2,ZP3,S100P,C1QL1,CST6,TNFRSF18,SNED1,FGFBP1,MUC4,ABCB4,CCL5,ANGPT2,PLAC9,C19ORF18,PPL,LEFTY1,COLQ,H2BC5,ERBB4,LRRN3,IL1B,DLL1,ECM2,EGF,MMP17,ST8SIA4,LIPG,LVRN,CSF3,USH2A,TMPRSS2,GLB1L2,COL6A2,SLCO4C1,GPX7,GPM6A,TCN1,PRSS2,GASK1A,GLIPR1L1,TNFRSF8,ALDOC,NPR3,TRIL,OMD,TMT1A,ADAMTS14,LTB,PARD6B,PTPRC,ABCC6,SPEF2,ITGBL1,PECAM1,FAM20A,LRRN1,LRRK2,C5,ADAM28,PLXNB1,GDF5,APOL6,ADPRH,NOTCH2NLB,ANO1,KRT81,PCBP3,MUC5B,LIFR,ENPP1,PLCG2,TFPI,KRT86,SAA2,C1R,ITGA1,GPC6,LCP1,C1QTNF1,SHH,CXCL11,PRH2,VPS37D,SYNE2,ALDH1L2,TNFSF4,DLG3,TNFSF10,PDGFB,CPE,SPOCK3,HLA-DRB5,FAM20C,ARSI,TCN2,INHBB,VASH2,SVEP1,SEMA3E,CLIC5,LEPR,LAMB1,CARD11,GNG7,IL6,ITGB8,CTSS,LFNG,SORL1,CFD,HLA-DRA,CPNE7,PTPN6,CD163L1,GCA,CXCL8,PTPRJ,MOXD1,LRIG1,DMBT1,PRSS23,COL4A5,C3,H2AC6,PDGFA,PYCARD,FUT2,LYZ,ADGRV1,PGF,HLA-G,ITGB4,BTC,IL7R,HLA-DRB1,GLIPR2,ARSJ,FAT1,COCH,C1S,GMFG,SEMA3D,VPS13C,IRF2BPL,MDFIC,SLC37A2,APOM,LNPEP,PPFIBP2,IL1A,ADAMTS3,MVB12B,ANO6,RAP2B,ST6GAL1,MMP16,FRMD4B,C2,TLR3,ISM1,TTBK2,NO*

			TCH2NLA,NRP1,APOLD1,APOL3,COL18A1
GO:0045178	basal part of cell	0.0000395387025722237 5	*SLCO2A1,SLC10A1,EPPK1,ABCA1,ERBB4,CADM1,CLDN11,SLCO4C1,SLC16A6,SLC47A1,SLC19A1,ABCC6,ANK2,P2RY1,SLC4A8,ENPP1,SLCO1B1,ITGA1,MAP7,DLG3,PDGFB,P2RY6,SLC14A1,BEST1,LEPR,LIN7C,ITGB4,CALHM3,CA14,SLC40A1,VSIG1,CA12*
GO:0009925	basal plasma membrane	0.0001013630008249693 2	*SLCO2A1,SLC10A1,EPPK1,ABCA1,ERBB4,CADM1,SLCO4C1,SLC16A6,SLC47A1,SLC19A1,ABCC6,ANK2,P2RY1,SLC4A8,ENPP1,SLCO1B1,MAP7,DLG3,PDGFB,P2RY6,SLC14A1,BEST1,LEPR,LIN7C,ITGB4,CALHM3,CA14,SLC40A1,VSIG1,CA12*
GO:0005615	extracellular space	0.0002571722671497262 3	*H2AC21,H2BC14,H2AC15,GNG2,H4C2,H3C13,H2AC12,H2AC20,H2BC13,SPN,H2BC18,F13A1,H2AC14,SERPINA5,H2AC1,CCL24,H2BC21,H2BC6,GPRC5C,TNFSF18,KRT83,BMP5,RASSF9,VCAM1,NDP,ALDH3A1,GLDN,H2BC11,COLEC12,MUC5AC,SCRG1,MCF2L,AGR2,LRRTM2,OGN,PCDHGB5,H2BC15,SERPINA1,CXCL10,CHI3L2,ADAMTS20,STEAP4,NTF3,ZG16B,ZP3,S100P,CST6,FGFBP1,MUC4,ABCB4,CCL5,ANGPT2,C19ORF18,PPL,LEFTY1,COLQ,H2BC5,LRRN3,IL1B,EGF,MMP17,LIPG,LVRN,CSF3,TMPRSS2,COL6A2,SLCO4C1,GPM6A,TCN1,PRSS2,GLIPR1L1,TNFRSF8,ALDOC,NPR3,TRIL,OMD,LTB,PARD6B,PTPRC,PECAM1,FAM20A,LRRN1,LRRK2,C5,GDF5,ADPRH,ANO1,KRT81,PCBP3,MUC5B,LIFR,ENPP1,PLCG2,TFPI,KRT86,SAA2,ITGA1,LCP1,C1QTNF1,SHH,CXCL11,PRH2,VPS37D,SYNE2,ALDH1L2,TNFSF4,DLG3,TNFSF10,PDGFB,CPE,SPOCK3,HLA-DRB5,FAM20C,TCN2,INHBB,SVEP1,SEMA3E,CLIC5,LAMB1,CARD11,GNG7,IL6,ITGB8,CTSS,SORL1,CFD,HLA-DRA,CPNE7,PTPN6,GCA,CXCL8,PTPRJ,MOXD1,LRIG1,DMBT1,PRSS23,COL4A5,C3,H2AC6,PDGFA,FUT2,LYZ,ADGRV1,PGF,HLA-G,ITGB4,BTC,HLA-DRB1,GLIPR2,FAT1,C1S,SEMA3D,VPS13C,IRF2BPL,SLC37A2,APOM,LNPEP,PPFIBP2,IL1A,ADAMTS3,MVB12B,ANO6,RAP2B,MMP16,FRMD4B,C2,TLR3,TTBK2,NRP1,COL18A1*
GO:0032993	protein-DNA complex	0.0002658795872060418 3	*H2AC21,H2BC14,H2AC15,H2BC3,H4C2,H2BC1,H3C13,H2AC12,H1-4,H2AC20,H2BC13,H2BC17,H2BC18,H2AC14,H2AC1,H2BC21,H2BC6,H2BC11,H1-6,H2BC15,H1-5,H2BC5,EYA1,H1-2,H2AC6,HELB*
GO:0042613	MHC class II protein complex	0.0003864355057521548 6	*HLA-DPB1,HLA-DPA1,HLA-DOA,HLA-DRB5,HLA-DRA,HLA-DRB1,HLA-DMB*
GO:0042611	MHC protein complex	0.0004290189234620333 3	*HLA-DPB1,HLA-DPA1,HLA-DOA,HLA-DRB5,HLA-DRA,HLA-G,HLA-DRB1,HLA-DMB*
GO:0098590	plasma membrane region	0.001787013573475268	*SLCO2A1,DHRS3,CACNG4,TESC,LRRTM2,TENM2,SLC10A1,EPPK1,ZG16B,GRIN2B,ABCA1,S100P,ABCB4,SSPN,TRPM8,ERBB4,DLL1,CADM1,USH2A,SLCO4C1,GPM6A,SNAP25,SLC16A6,GASK1A,SLC47A1,TIAM1,MYLK,SLC19A1,PARD6B,ABCC6,ANK2,LRRK2,P2RY1,PLXNB1,JCAD,UNC13A,ANO1,GPR158,SLC4A8,ENPP1,PLCG2,DLC1,TFPI,SLCO1B1,LCP1,GRIK4,SNAP91,MAP7,SYNE2,DLG3,PDGFB,P2RY6,SLC14A1,BEST1,CLIC5,HIP1,LEPR,KCTD12,SORCS2,SLC1A7,PTPRJ,LIN7C,FZD3,ADGRV1,HLA-G,ITGB4,IGSF9B,FAT1,SVIL,SPRED1,CALHM3,AKAP9,PDE9A,KCNJ8,ASPM,CA14,SLC40A1,NRP1,VSIG1,CA12*

GO:0031012	extracellular matrix	0.002357640797692062	*SPN,F13A1,SERPINA5,NDP,COLEC12,MUC5AC,OGN,SERPINA1,ADAMTS20,ADAMTSL2,ZP3,SNED1,MUC4,ANGPT2,COLQ,LRRN3,ECM2,MMP17,USH2A,COL6A2,PRSS2,TRIL,OMD,ADAMTS14,LRRN1,MUC5B,GPC6,SHH,PDGFB,SPOCK3,LAMB1,CTSS,FLG,LRIG1,DMBT1,COL4A5,ITGB4,COCH,ADAMTS3,MMP16,TLR3,COL18A1*
GO:0030312	external encapsulating structure	0.0024629947960673184	*SPN,F13A1,SERPINA5,NDP,COLEC12,MUC5AC,OGN,SERPINA1,ADAMTS20,ADAMTSL2,ZP3,SNED1,MUC4,ANGPT2,COLQ,LRRN3,ECM2,MMP17,USH2A,COL6A2,PRSS2,TRIL,OMD,ADAMTS14,LRRN1,MUC5B,GPC6,SHH,PDGFB,SPOCK3,LAMB1,CTSS,FLG,LRIG1,DMBT1,COL4A5,ITGB4,COCH,ADAMTS3,MMP16,TLR3,COL18A1*
GO:0005685	U1 snRNP	0.0026564493422614886	*RNVU1-31,RNVU1-28,RNU1-1,RNVU1-7,RNVU1-34,RNVU1-8,RNVU1-29,RNVU1-3,RNU1-2,RNVU1-2,RNVU1-27,RNU1-67P,ENSG00000287979,RNVU1-15,RNVU1-4,ENSG00000287190,RNVU1-30*
GO:0016020	membrane	0.00425582643133874	*GNG2,H4C2,SPN,LZTS1,PTPRN2,B4GALNT3,SERPINA5,PCDHGA8,LAIR2,KLRC2,GPRC5C,TNFSF18,TM4SF18,RASSF9,ADGRF1,SLCO2A1,HSD3B1,VCAM1,DHRS3,JAK3,ABCG1,TMEM225B,ALDH3A1,CACNG4,GLDN,H2BC11,COLEC12,MUC5AC,SLC2A14,TAS2R30,TESC,DCHS1,MCF2L,PCDHGA11,LRRTM2,SCN3A,PCDHGB7,SLC16A14,PCDHGB5,TRPM3,SLC30A10,RAPGEF4,SERPINA1,PROKR1,TENM2,SLC10A1,B3GALT2,HOGA1,EPPK1,CXCL10,FGF13,SHISA3,LAMP3,RAB39A,STEAP4,F2RL3,TRBC2,SLITRK6,NUP210L,HCLS1,TRAC,KLRC3,RAB37,SHISAL1,ZG16B,SCARA5,KCNJ13,GRIN2B,VAV1,LGR6,B3GALT1,LRRC19,KCNT2,ABCA1,GYPC,ZP3,GNAZ,NTSR1,NDUFA4L2,NCMAP,S100P,ATP9A,CYP19A1,CST6,TNFRSF18,TAS2R13,FGFBP1,THSD7B,CKMT2,MUC4,ABCB4,NKPD1,AR,TMPRSS3,FER1L5,MEGF10,C19ORF18,PLXNA4,PCDHGA2,PPL,SSPN,C11ORF87,COLQ,HYAL4,TNFRSF9,TRPM8,MGMT,PCDHGB4,ENSG00000293570,ABCC12,CCND2,ABCD2,ERBB4,LRRN3,TRHR,ADRA2C,CPLX1,NOX5,ATP2A3,DLL1,SPTSSB,CADM1,SLC13A4,GIMAP2,EGF,PCDHGA12,SNX22,MMP17,ST8SIA4,PCDHGA10,CLDN11,PLN,PLCB4,SLC9A9,LVRN,USH2A,PCDHGA4,TMPRSS2,SLC2A10,FABP6,COL6A2,SLCO4C1,GPR176,GPM6A,SNAP25,SLC16A6,PCDHB12,GCNT4,HLA-DPB1,GASK1A,GLIPR1L1,TNFRSF8,SLC47A1,ESR1,STXBP5L,SLC37A1,ITGB6,NPR3,TRIL,KCNH3,TSPAN11,CHST6,MPZL2,TIAM1,MYLK,LRP1B,TMT1A,PDE3A,ULK2,RELL1,SEMA6B,SLC19A1,TMEM232,LTB,BEAN1,ST8SIA1,CCR1,SMIM31,PIK3AP1,PARD6B,PTPRC,ABCC6,TMEM45B,ANK2,ITGBL1,PECAM1,PCDH7,IL20RA,LRRN1,LRRK2,PCDHGB6,P2RY1,SLC2A12,GRK3,C5,ADAM28,RTP4,KLB,PLXNB1,GDF5,APOL6,JCAD,UNC13A,GPR162,ANO1,PCDHGB3,GPRIN3,GPR158,NPR1,MUC5B,LIFR,SLC4A8,ENPP1,PLCG2,HLA-DPA1,PCDHB11,DLC1,TFPI,SLCO1B1,CRTAM,GRAMD1C,TSPAN33,RAB40A,ITGA1,GPC6,HLA-DOA,GPR85,LCP1,C1QTNF1,TRIM59,SHH,GRIK4,SNAP91,VPS37D,MAP7,PLAAT4,QRICH1,NUP210,SYNE2,ABCC9,SPRY1,TNFSF4,INSIG1,MAP3K5,DLG3,TNFSF10,EPHB6,PDGFB,MAGED1,PRKG1,SEMA6D,PCDHGA9,CPE,TMEM121,COPZ2,CNTNAP3B,HLA-DRB5,FAM20C,TCN2,P2RY6,FAM171B,GALNT6,FAM169A,C10ORF90,ITGA10,PTCH2,ABCA13,TGM5,SV*

			EP1,ST6GALNAC2,OASL,TBXAS1,SLC14A1,PCDHB10,FLVCR2,FAR2,SEMA3E,CMKLR1,BEST1,CLIC5,HIP1,LEPR,STEAP2,PARP14,KCTD12,CARD11,PIK3CG,GALNT12,TMEM150C,CLMN,GNG7,KREMEN1,CADPS2,PCDHGB1,GJB2,GPRIN2,IL6,DAPK2,ITGB8,TAS2R20,SORCS2,UQCC3,MGAT2,LFNG,SORL1,ALOX5AP,CALHM5,ITPR2,FZD1,RASSF2,HLA-DRA,CPNE7,PTPN6,CD163L1,GCA,ST3GAL5,TNFRSF10C,SLC1A7,LRP5,EVL,UST,PREX1,FLG,PCDHGA1,PTPRJ,MOXD1,LRIG1,ATRNL1,TMEM135,LIN7C,ADRB1,DMBT1,SLC39A11,PHACTR2,GPR55,DENND5B,CYTH4,PCDHGA5,FZD3,COL4A5,TRBV13,C3,PDGFA,ILDR2,PYCARD,FUT2,DNAJC22,TBC1D5,PCDHGA7,ADGRV1,PGF,HLA-G,ITGB4,BTC,PAQR8,IL7R,HLA-DRB1,SLC9A7,PCDHGA6,GPR153,GLIPR2,CHN2,MYO16,ARSJ,SH3TC2,IGSF9B,FAT1,HLTF,PNPLA4,SVIL,APBA2,SEMA3D,VPS13C,CELSR1,SPRED1,CALHM3,MDFIC,UGCG,SLC37A2,LNPEP,ADGRF5,AKAP9,MVB12B,ANO6,PCDHB8,RAP2B,ST6GAL1,MX2,PDE9A,MMP16,CLDN23,KCNJ8,TMEM139,MX1,LANCL1,SNX13,ASPM,CA14,SLC40A1,PXMP4,TLR3,SYNJ2,GBP4,PRICKLE1,NRP1,APOLD1,VSIG1,SYT2,MDM2,HLA-DMB,APOL3,RGS5,NDST3,SLC35F3,CA12,KIF14,IMMP2L,ZNF546
GO:0110165	cellular anatomical structure	0.008580482318624282	*RNVU1-31,RNU4-1,RN7SL4P,RN7SL1,RN7SL3,RNU4-2,H2AC21,RNVU1-28,SCARNA5,SNORA54,H2BC14,SNORD3A,RNU5F-1,SNORA79B,SCARNA21,H2AC15,GNG2,H2BC3,SNORD13,SNORA23,H4C2,RN7SL555P,SNORA38,SCARNA7,H2BC1,H3C13,H2AC12,SCARNA6,H1-4,RNU4ATAC,H2AC20,SPANXC,RNU5B-1,SNORA73B,SNORA12,MAGEA12,H2BC13,SPN,SNORA74A,RN7SL674P,LZTS1,RNU1-1,H2BC17,RN7SL767P,H2BC18,SNORA80D,F13A1,PTPRN2,SNORA7B,SNORA38B,H2AC14,RN7SL507P,B4GALNT3,EDARADD,SNORA80A,SNORA49,SERPINA5,H2AC1,PCDHGA8,RNVU1-7,SNORA74B,CCL24,H2BC21,RNU5E-4P,SNORD15B,H2BC6,LAIR2,KLRC2,GPRC5C,RNVU1-34,RNVU1-8,SNORA80B,ASB2,RNU2-63P,TNFSF18,RNVU1-29,KRT83,TP63,SNORD17,SNORA47,TM4SF18,BMP5,RNVU1-3,RASSF9,ADGRF1,SLCO2A1,HSD3B1,VCAM1,DHRS3,NDP,JAK3,ABCG1,TMEM225B,BHLHA15,SNORA2A,ALDH3A1,RNU5D-1,CACNG4,GLDN,H2BC11,COLEC12,RNU1-2,H1-6,SNORD100,SNORD3C,MUC5AC,SLC2A14,SNORA22C,ENSG00000287774,TAS2R30,RNVU1-2,ELF3,RNVU1-27,SCRG1,TESC,DCHS1,MCF2L,PCDHGA11,AGR2,LRRTM2,OGN,SCN3A,RNU6ATAC,PCDHGB7,RNU1-67P,SPANXD,SLC16A14,MEIS3,ENSG00000287979,PCDHGB5,SCARNA3,SCARNA1,MACC1,H2BC15,SCARNA18,TRPM3,ZNF334,RIMKLA,RP1L1,SLC30A10,RAPGEF4,SERPINA1,PROKR1,TENM2,SLC10A1,ZNF460,B3GALT2,YPEL4,SNORA2C,SNORA28,HOGA1,ZBTB20,SCARNA9,EPPK1,PLD5P1,PDK4,CXCL10,FGF13,SNORD3B-2,SHISA3,CHI3L2,POU5F2,SNORA14A,INSYN2A,LAMP3,TNS4,ADAMTS20,SNORA22,RAB39A,BCL11B,*

			BLID,STEAP4,PADI3,F2RL3,TRBC2,SLITRK6,NTF3,NUP210L,SNORA79,HCLS1,ARHGAP28,RNVU1-15,TRAC,KLRC3,SNORD94,NLRP14,RAB37,SNORA74C-2,SHISAL1,RNVU1-4,ZG16B,SNORA66,SCARNA23,ASB4,SCARA5,KCNJ13,GRIN2B,PLAAT5,VAV1,LGR6,SNORD59A,SPANXB1,B3GALT1,LRRC19,KCNT2,ABCA1,ADAMTSL2,GYPC,ZP3,GNAZ,NTSR1,MYL1,NDUFA4L2,NXF3,NCMAP,S100P,ATP9A,C1QL1,C2CD4C,CYP19A1,SNORA53,CST6,CSAG1,PPP4R4,RNU2-64P,DMC1,TNFRSF18,SNED1,TAS2R13,FGFBP1,ENSG00000287190,THSD7B,SNORD105B,SNORD82,FGF14,ZNF610,DCDC2B,H1-5,KIF5C,GSX2,CKMT2,SOX4,MUC4,ABCB4,NKPD1,AK7,ALDH1A2,CCL5,AR,LGALS12,ANGPT2,TMPRSS3,PLAC9,FER1L5,MEGF10,C19ORF18,PLXNA4,PCDHGA2,PPL,SSPN,CDK15,BTBD18,LEFTY1,ANKRD7,C11ORF87,COLQ,HYAL4,KLF8,APOBEC3G,TNFRSF9,TEX15,TRPM8,MGMT,PCDHGB4,DDX60,SULT1C2,ENSG00000293570,TRIM61,ABCC12,ERP27,NKX1-2,H2BC5,PRDM16,CCND2,ABCD2,SNORD11,ERBB4,LRRN3,TRHR,SUMO4,IL1B,ADRA2C,RNU5A-1,CPLX1,NOX5,ATP2A3,DLL1,SPTSSB,CADM1,SNORD73B,SLC13A4,ECM2,GIMAP2,EGF,PCDHGA12,SNX22,MMP17,ST8SIA4,ZBED6,AHRR,LIPG,PCDHGA10,CLDN11,PLN,SNORA19,PLCB4,RNVU1-30,SLC9A9,LVRN,CSF3,XIRP2,USH2A,PCDHGA4,TMPRSS2,GLB1L2,SLC2A10,EYA1,FABP6,COL6A2,SLCO4C1,GPX7,GPR176,GPM6A,PPP1R1C,TCN1,SNAP25,PRSS2,SLC16A6,PCDHB12,SNORD12C,GCNT4,HLA-DPB1,GASK1A,CRACD,GLIPR1L1,TNFRSF8,SLC47A1,ESR1,ALDOC,STXBP5L,PBX1,FSIP2,SNORA22B,CSRNP3,HR,SNORD46,SLC37A1,DUSP9,ITGB6,NPR3,TRIL,ZMAT1,KCNH3,TSPAN11,CHST6,MPZL2,OMD,TIAM1,MYLK,SNORD89,SNORD67,SNORA71B,LRP1B,TMT1A,PDE3A,ULK2,ADAMTS14,RELL1,ZNF727,SEMA6B,SLC19A1,ASB14,DNAH7,TMEM232,LTB,LONRF1,BEAN1,ST8SIA1,CCR1,EGLN3,SMIM31,PIK3AP1,PARD6B,GLUD2,PTPRC,ABCC6,TMEM45B,XAF1,SPEF2,ANK2,ITGBL1,PDE5A,PECAM1,EXPH5,FAM20A,ALPK1,PCDH7,IL20RA,LRRN1,ARHGAP30,LRRK2,PCDHGB6,COLGALT2,P2RY1,SLC2A12,GRK3,C5,ADAM28,RTP4,KLB,PLXNB1,GDF5,APOL6,ADPRH,JCAD,MAEL,UNC13A,NOTCH2NLB,GPR162,ANO1,PCDHGB3,GPRIN3,GPR158,KRT81,NPR1,PCBP3,MUC5B,LIFR,SLC4A8,RADIL,ENPP1,PDK3,PLCG2,ZNF382,HLA-DPA1,PCDHB11,RNU6-2,IRX5,DLC1,DNAH12,TFPI,KRT86,SLCO1B1,P3H3,TPRG1,CRTAM,SAA2,GRAMD1C,TSPAN33,RAB40A,H1-2,C1R,ITGA1,SOX2,GPC6,HLA-DOA,GPR85,LCP1,C1QTNF1,ZNF737,TRIM59,SHH,GRIK4,SNORD6,CXCL11,RORB,PRH2,SNAP91,ZNF680,VPS37D,MAP7,PLAAT4,QRICH1,NUP210,SYNE2,AIM2,ABCC9,ALDH1L2,SPRY1,TNFSF4,ZNF678,SRRM3,INSIG1,MAP3K5,RGCC,NR5A2,DLG3,TNFSF10,EPHB6,STMN3,PDGFB,MAGED1,PITX2,PRKG1,FOXP2,SEMA6D,PCDHGA9,CPE,SPOCK3,ZNF254,TMEM121,COPZ2,CNTNAP3B,SNORA3B,HLA-DRB5,FAM20C,ARSI,STK31,NEK3,TCN2,P2RY6,FAM171B,GALNT6,FAM169A,C10ORF90,ITGA10,SNAI3,MAP2K6,SLFN5,FOCAD,INHBB,PTCH2,ABCA13,TGM5,DENND2A,IRX2,VASH2,TCEAL8,SRGAP3,SVEP1,ST6GALNAC2,OASL,TBXAS1,TRIM2,SLC14A1,ZF

			P3,SPOUT1,PCDHB10,FLVCR2,FAR2,SNORA5A,RNU6-8,SEMA3E,CMKLR1,BEST1,CLIC5,ZNF714,HIP1,KLHL4,LEPR,STEAP2,ETV1,FHDC1,LAMB1,PARP14,KCTD12,CARD11,PIK3CG,GALNT12,TMEM150C,CLMN,GNG7,KREMEN1,CADPS2,RBM47,PCDHGB1,GJB2,GPRIN2,IL6,DAPK2,ITGB8,TAS2R20,SORCS2,CTSS,ZBTB41,ZNF630,UQCC3,SLFN13,MGAT2,LFNG,ZNF92,SORL1,CFD,ALOX5AP,CALHM5,ITPR2,ZNF704,FZD1,RASSF2,PPP2R2C,HLA-DRA,CPNE7,ISG20,PTPN6,CD163L1,GCA,ST3GAL5,ZXDA,TNFRSF10C,HMGB3,GSAP,SLC1A7,LRP5,EVL,ALDH6A1,UST,CXCL8,PREX1,PPP1R9A,FLG,PCDHGA1,PTPRJ,MOXD1,IRX1,LRIG1,ATRNL1,ARHGEF10,TMEM135,LIN7C,JDP2,RLIM,ADRB1,DMBT1,RO60,SLC39A11,PHACTR2,DENND11,PROB1,GPR55,DENND5B,PRSS23,RPS6KA2,CYTH4,DIAPH2,ACOT1,PCDHGA5,FZD3,COL4A5,TRBV13,RDM1,CGNL1,C3,KLHL11,IRF2BP2,ZBTB10,H2AC6,CCNB3,PPP1R3C,PDGFA,SNORA14B,CEBPD,HELLPAR,ILDR2,PYCARD,NEURL1B,FUT2,ATRX,GSPT2,DNAJC22,LYZ,TBC1D5,PCDHGA7,ADGRV1,PGF,HLA-G,ITGB4,ATF7IP2,BTC,PAQR8,TNS3,RBMS3,IL7R,HLA-DRB1,SLC9A7,SUGCT,PCDHGA6,GPR153,GLIPR2,CHN2,MYO16,RCSD1,ARSJ,SH3TC2,IGSF9B,ZNF430,KLHL24,FAT1,NNMT,COCH,C1S,HLTF,GMFG,PNPLA4,SVIL,APBA2,SEMA3D,VPS13C,HOXA13,CELSR1,IRF2BPL,SPRED1,CALHM3,FAM114A1,MDFIC,AHR,UGCG,SLC37A2,BEX2,APOM,LNPEP,PPFIBP2,CLIP2,ADGRF5,DIXDC1,IL1A,ADAMTS3,AKAP9,CXXC4,MVB12B,ANO6,PCDHB8,RAP2B,HELB,ST6GAL1,MX2,PDE9A,CDKL2,MMP16,DDIT4,CLDN23,CRPPA,KCNJ8,IKZF2,SETX,TMEM139,FRMD4B,ZNF525,MX1,ZNF573,LANCL1,SNX13,ASPM,RANBP17,C2,BICC1,ZNF273,ZNF518B,CA14,SLC40A1,PXMP4,TLR3,CFAP300,ISM1,SYNJ2,TTBK2,NOTCH2NLA,ZNF805,GBP4,DNAH3,PDE10A,ZNF660,PRICKLE1,NRP1,G2E3,ZNF91,APOLD1,VSIG1,IFIH1,SYT2,MDM2,FBXL13,HLA-DMB,APOL3,ZNF766,TTC39A,RGS5,MSX2,NDST3,SLC35F3,CA12,KIF14,IMMP2L,COL18A1,ZNF546,ACSM4

Table S3. GO associations with molecular functions (MF), biological processes (BP), and cellular components (CC) of 1197 upregulated genes in MB cells grown on 3D matrix. Related to Figure 1C.

GO.ID	Description	padj	Genes
		**MF**	
GO:0005515	protein binding	2.3768444790179786e-8	*FUS,RPS16,CCN1,EIF5B,EIF1,RPS27,MYADM,MT-CO2,MT-ND2,MT-CO1,MT-ND3,MT-ND1,MT-ATP6,RPS3A,FTH1,ANP32B,PABPC1,SRRM2,HNRNPH3,ARHGDIA,SH3BGRL3,RPL21,RIPOR1,MT-ATP8,SRM,RPL7,FOSL1,ATIC,UTP4,HNRNPA3,MT2A,PRKACA,MAT2A,FXYD5,RPL9,SF3B4,USB1,RPL18A,CTDNEP1,S100A11,BRD4,MT-CO3,CCN3,ATXN2L,SF1,RPS28,HNRNPD,FKBP1A,DI*

			APH1,TAF15,PRPF19,DDX39B,USP14,LENG8,SMG7,CCDC86,PXN,ATN1,PTPN23,HNRNPA2B1,MAP1LC3B,CCDC69,RAB11B,AGPAT1,TYMS,TECR,FST,RAB7A,CIZ1,CHERP,ADM,KHSRP,PSMD2,FOXP4,AKT1S1,EHD2,NUDC,CD44,ODC1,SLC9A1,PPP1R11,ECM1,BRPF3,PPARD,RTL8A,NXF1,CMIP,ZFP36L1,LAMB3,DLGAP4,TRMT1,CCDC124,PCBP1,CDA,MDC1,WIZ,LOX,ALG3,ZYX,MAPK13,BYSL,NOB1,ENTREP3,PDLIM7,ESAM,PPP1R13L,PCGF2,C4BPB,CHAMP1,CRTC2,PACS1,DTNBP1,PSMC1,RPL17,MAL2,TONSL,PDCL3,ZRANB2,SRRM1,BCL9L,VAC14,SF3A2,NCOR2,SAMD4B,DVL3,ADAMTS1,ZNF114,NPRL3,SLAMF7,PLAGL2,BAK1,UPF3B,POM121C,TRAPPC1,STX16,NFIX,MED15,NFIC,FBXL6,NCOA6,EPS15L1,XAB2,PDE4A,VAMP2,GAR1,ZMIZ2,NRM,GPX3,PCIF1,CDKN1A,TP53INP2,EDC3,PRCC,MYD88,MCOLN1,SKI,GNG5,MAP2K7,CXXC1,RHOJ,PDE1C,ZNF521,SNRPA1,GSK3A,SCAF4,SP2,PRSS3,TINAGL1,PLEKHA7,CENPT,PBX2,CAMTA2,DUSP7,CXCR4,UPF3A,UCP2,EIF2B4,CNOT3,TPP1,RPS26,CRTC1,CC2D1A,TAF1C,ATF5,SORBS3,CDK11B,PHGDH,PROSER1,ZC3H3,NRGN,RERE,PHACTR4,GATAD2B,ALDOA,BCORL1,DGAT1,MBD6,DMAP1,PLBD2,CCBE1,BET1L,MEF2D,HMOX1,SETD1A,EGR1,LRRC61,FAM98B,COL1A1,HGH1,GABARAP,NGFR,FHL3,SYVN1,NSRP1,TENM1,ZSWIM9,PRR14,POM121,R3HDM1,LSM10,DTNA,ZNF580,ESRP2,GATA6,SERPINB8,UBALD1,CREB3L1,SCAMP5,STXBP1,EIF3C,CAMK2N1,CGN,DMD,NLGN2,NPDC1,SETD1B,PLK3,RELN,CCNK,ENO3,SLC38A7,ABTB1,FURIN,FAM222B,ALS2CL,PLXDC2,PEAR1,GSTM1,CHRDL1,SLC39A4,YJEFN3,DOK3,CTH,GPR3,NHSL3,RTKN,PRR16,EVA1A,GLI4,LIMS2,NAA80,ALPP,DOCK6,PORCN,ZNF385A,PRAF2,RAB13,KIFC2,ZFTRAF1,MLLT11,DAPK1,MRNIP,SPHK1,OSGIN1,RASEF,ZBTB12,PLEKHN1,MAGEC1,TAX1BP3,SYNGAP1,SLC29A3,ID4,ZNF703,RIN3,RGS4,MNT,RNF44,DIPK1B,ILK,BICRA,NAPRT,NAP1L3,FZD8,KRTAP2-3,GLIS2,SLC9A5,HSF4,TUBB3,LY6G5B,GAREM1,MFSD3,KDM6B,FABP5,ARHGEF19,LDOC1,PAX8,MAGEC2,TFAP2A,TNFSF15,GPRC5B,PSD4,CACNG7,ISYNA1,ANKRD13B,NR1D1,C5ORF46,ARC,ABHD16A,PDE2A,ZFP36,CYP1A1,SYT11,SIX2,AGAP4,SP140,ZNF296,BMPER,TNFSF12,PDGFD,ARSA,S1PR1,LRRC2,HSPB8,SLC25A35,ARID3B,TRIM66,C20ORF96,SLC44A5,FNDC4,ATP6V0C,RNF208,GATA3,GATA2,IKZF4,LOXL3,GEMIN7,SCNN1D,CNKSR2,MYO7B,CCDC120,SUPT4H1,KCNK1,ENTPD3,NCF2,DHPS,TTC4,RPP21,RFXAP,CTF1,LY6G5C,GSTT2B,EDN1,AMH,CCDC17,TMEM276,APBB3,IGF2,CDK11A,MMP2,SEMA6A,NPIPB3,MC1R,CORO1A,ZNF414,SLITRK1,DDT,PRKCG,MKRN3,GCKR,RAB3A,GGT7,PAK6,RNASEK,LCAT,PRRX1,ARL2BP,MTFP1,SORCS3,SEC31B,ASIC1,WDR97,GABRA3,SERPINE1,SLC52A3,POU3F2,LIN37,FAM83A,FOSB,PARP15,AOC2,RNF39,CST1,ACTRT3,RANGRF,GNG10,CDH19,RHBDL1,WASHC1,MMP3,ACTA2,LRRC15,PTPN22,ABLIM2,COL1A2,CYRIA,FBXL16,CST4,EGFL8,MEIS1,NPL,S1PR3,TCF4,PRRT2,CYSRT1,CEACAM19,TRPV4,BMP6,CLDN1,KIRREL3,BCL2L2-PABPN1,PHACTR1,ASB16,ANPEP,RAB3IL1,TGFBR3L,GPR15,PCOLCE,DPYSL3,KCNK2,SNX10,RNASEH2A,SETBP1,GNG13,ESPN,OIP5,BTBD19,ANKLE1,HLX,C10ORF67,GNMT,MYG1,VWCE,LAMA1,PTHLH,DBH,CD177,AOC3,GREM1,FOXA3,ANGPTL6,SCO2,OLR1,LYPD3,AMTN,SYT15,NRXN3,MAP1LC3B2,ADAMTSL3,ANO7,BCHE,TAS2R5,EPB41L4A,SPDYE12,KCNIP2,ATP1A

			3,TNFRSF25,ELL3,CHD5,TMPRSS15,AGAP6,TPH1,NUSAP1,KCNC3,CRLF1,GJB3,NELL2,SRCIN1,BOLA2-SMG1P6,FOXD4,VSTM1,COL3A1,SAMSN1,NELFCD,FOXD4L1,EGR4,WNK4,S100Z,USHBP1,ARHGEF16,UCHL1,ESM1,IL1RN,SFRP1,KCNAB3,ATP6V1G2,GNRH2,PAQR6,DCAF8L2,EBI3,SPNS1,FLRT1,UCP3,C2ORF74,PCSK1N,CDK5R2,TIE1,KRT14,AATK,FOXO6,GTF2H4,PLCXD3,GRIN2A,HCN2,CGB7,CNFN,SERPINB5,SULT1B1,GOLGA8A,C1ORF74,DUSP26,KCNE5,TOP3B,CPT1B,BCKDHA,PCDHB6,TBX6,MAOB,EPHA6,FBXW10B,LYG1,LRRC36,C2ORF15,DLX4,MXRA5,ADGRB3,GAL3ST2,TMEM190,CALML6,RAPSN,KIAA0319,AMPH,SLC7A8,RBM20,KCND1,BGN,ADAMTS5,PLAGL1,SPINK1,MIF,ENHO,ALOXE3,LAG3,FNDC5,GKN2,GPRC5D,TTC9B,THY1,NPFF,CGB8,SOX7
GO:0003729	mRNA binding	0.000498055979071365 6	*FUS,RPS3A,PABPC1,SRRM2,RPL7,HNRNPA3,ATXN2L,SF1,HNRNPD,TAF15,DDX39B,HNRNPA2B1,TYMS,KHSRP,NXF1,ZFP36L1,PCBP1,SAMD4B,UPF3B,EDC3,UPF3A,RPS26,PABPC1L,NSRP1,ESRP2,ZNF385A,ARC,ZFP36,MT-TL1,MT-TH,MT-TQ,MT-TN,RBM20*
GO:0009055	electron transfer activity	0.003138542527802552 8	*MT-CO2,MT-CYB,MT-ND2,MT-ND5,MT-CO1,MT-ND3,MT-ND4,MT-ND1,MT-ND4L,MT-ND6,MT-CO3,PHGDH,NCF2,AOC2,MAOB*
GO:0015453	oxidoreduction-driven active transmembrane transporter activity	0.00573801013617846	*MT-CO2,MT-CYB,MT-ND2,MT-ND5,MT-CO1,MT-ND3,MT-ND4,MT-ND1,MT-ND4L,MT-ND6,MT-CO3*
GO:0015078	proton transmembrane transporter activity	0.008212205392042312	*MT-CO2,MT-CYB,MT-ND2,MT-ND5,MT-CO1,MT-ND3,MT-ND4,MT-ND1,MT-ATP6,MT-ND4L,MT-ND6,MT-ATP8,MT-CO3,SLC9A1,UCP2,SLC9A5,ATP6V0C,ATP6V1G2,UCP3*
GO:0022853	active monoatomic ion transmembrane transporter activity	0.021039223146006706	*MT-CO2,MT-CYB,MT-ND2,MT-ND5,MT-CO1,MT-ND3,MT-ND4,MT-ND1,MT-ND4L,MT-ND6,MT-CO3,ATP6V0C,ATP1A3,ATP6V1G2*
BP			
GO:0032501	multicellular organismal process	2.7809422731636894e-8	*CCN1,MYADM,MT-CO1,MT-ND4,ANP32B,FOSL1,ATIC,PRKACA,BRD4,CCN3,SF1,HNRNPD,FKBP1A,DIAPH1,ATN1,PTPN23,RAB11B,AGPAT1,TYMS,FST,RAB7A,CHERP,ADM,KHSRP,SLC29A1,CD44,ODC1,SLC9A1,PPP1R11,ECM1,BRPF3,PPARD,CMIP,ZFP36L1,LAMB3,DLGAP4,PPDPF,LOX,MAPK13,BYSL,NOB1,PDLIM7,ESAM,PPP1R13L,PCGF2,C4BPB,PACS1,DTNBP1,MAL2,PDCL3,BCL9L,SF3A2,NCOR2,DVL3,ADAMTS1,NPRL3,SLAMF7,PLAGL2,BAK1,UPF3B,MED15,NCOA6,XAB2,PDE4A,VAMP2,CDKN1A,TP53INP2,MYD88,SKI,GNG5,RHOJ,ZNF521,GSK3A,PRSS3,PLEKHA7,PBX2,CAMTA2,CXCR4,UPF3A,UCP2,EIF2B4,CNOT3,TPP1,CRTC1,CC2D1A,ATF5,PHGDH,NRGN,RERE,PHACTR4,ALDOA,DGAT1,CCBE1,MEF2D,HMOX1,SETD1A,AFF3,EGR1,COL1A1,NGFR,SYVN1,NSRP1,TENM1,FOXQ1,GPR137,DTNA,ZNF580,ESRP2,GATA6,CREB3L1,SCAMP5,STXBP1,CAMK2N1,DMD,NLGN2,RELN,ENO3,LTB4R,FURIN,PEAR1,CHRDL1,Y*

			JEFN3,CTH,GPR3,LIMS2,ZNF385A,ZBED3,RAB13,FAT3,SPHK1,ZBTB12,SOX12,SYNGAP1,ID4,ZNF703,RGS4,EVI2B,ILK,BICRA,FZD8,MYCT1,GLIS2,HSF4,TUBB3,KDM6B,GABRE,FABP5,LDOC1,PAX8,TFAP2A,GPRC5B,CACNG7,NR1D1,ARC,PDE2A,ZFP36,CYP1A1,SYT11,SIX2,ZNF296,BMPER,TNFSF12,PDGFD,S1PR1,LTB4R2,GATA3,GATA2,LOXL3,SCNN1D,MYO7B,DHPS,TRNP1,CTF1,EDN1,AMH,IGF2,MMP2,SEMA6A,MC1R,CORO1A,SLITRK1,DDT,PRKCG,RAB3A,CCDC78,SERPINB2,PAK6,PRRT1,LCAT,PRRX1,SORCS3,RAPGEFL1,ASIC1,GABRA3,SERPINE1,SLC52A3,POU3F2,FOSB,AOC2,CST1,RANGRF,ACTA2,PTPN22,COL1A2,CST4,MEIS1,S1PR3,TCF4,PRRT2,TRPV4,BMP6,CYP2J2,CLDN1,KIRREL3,PHACTR1,SYPL2,ANPEP,GPR15,DPYSL3,KCNK2,OR2B6,SNX10,GPR35,INKA1,GNG13,ESPN,HLX,MYG1,PKDCC,LAMA1,PTHLH,DBH,CD177,GREM1,FOXA3,ANGPTL6,SCO2,OLR1,AMTN,NRXN3,BCHE,TAS2R5,KCNIP2,ATP1A3,ELL3,CHD5,TPH1,HOXD1,CRLF1,GJB3,NELL2,SRCIN1,COL3A1,STIMATE-MUSTN1,SAMSN1,WNK4,UCHL1,ESM1,IL1RN,SFRP1,GNRH2,SERPINB7,EBI3,PANX2,FLRT1,UCP3,PCSK1N,CDK5R2,TIE1,KRT14,AATK,FOXO6,GRIN2A,HSD11B2,HLA-DOB,PCDHB2,CNFN,SERPINB5,CCDC154,KCNE5,HOXB8,PCDHB6,TBX6,MAOB,EPHA6,ADGRB3,TMEM119,KIAA0319,TCHH,SLC7A8,RBM20,BGN,ADAMTS5,SPINK1,MIF,ALOXE3,LAG3,TNFRSF13C,THY1,ONECUT1,NPFF,DRGX,SLC25A34,SOX7
GO:0048522	positive regulation of cellular process	2.3224842804695828e-7	FUS,CCN1,EIF1,MYADM,ANP32B,PABPC1,RIPOR1,FOSL1,PRKACA,MAT2A,SF3B4,CTDNEP1,S100A11,BRD4,CCN3,HNRNPD,FKBP1A,TAF15,PRPF19,PXN,ATN1,PTPN23,HNRNPA2B1,AGPAT1,RAB7A,CIZ1,CHERP,ADM,KHSRP,EHD2,CD44,ODC1,SLC9A1,ECM1,BRPF3,PPARD,ZFP36L1,CCDC124,PCBP1,MDC1,WIZ,MAPK13,PPP1R13L,C4BPB,CRTC2,DTNBP1,PSMC1,PDCL3,BCL9L,SF3A2,SAMD4B,DVL3,ADAMTS1,NPRL3,PLAGL2,BAK1,UPF3B,NFIX,MED15,NFIC,NCOA6,VAMP2,ZMIZ2,PCIF1,CDKN1A,TP53INP2,MYD88,MCOLN1,SKI,GNG5,MAP2K7,CXXC1,RHOJ,ZNF521,GSK3A,PBX2,CAMTA2,CXCR4,UPF3A,UCP2,CNOT3,CRTC1,CC2D1A,ATF5,SORBS3,ZC3H3,RERE,GATAD2B,DMAP1,CCBE1,PABPC1L,MEF2D,HMOX1,EGR1,FAM98B,COL1A1,GABARAP,NGFR,TENM1,GPR137,LSM10,ZNF580,ESRP2,GATA6,CREB3L1,SCAMP5,STXBP1,EIF3C,DMD,NLGN2,PLK3,RELN,MMP1,CCNK,FURIN,PEAR1,EIF5AL1,DOK3,CTH,GPR3,PRR16,LIMS2,ZNF385A,ZBED3,MLLT11,DAPK1,MRNIP,SPHK1,PLEKHN1,SOX12,ID4,ZNF703,RGS4,EVI2B,ILK,BICRA,GLIS2,NRK,GAREM1,KDM6B,FABP5,PAX8,MAGEC2,TFAP2A,TNFSF15,GPRC5B,CACNG7,NR1D1,ARC,PDE2A,ZFP36,CYP1A1,SYT11,SIX2,BMPER,TNFSF12,PDGFD,S1PR1,HSPB8,ARID3B,ATP6V0C,HMGN5,GATA3,GATA2,IKZF4,LOXL3,SUPT4H1,DHPS,RFXAP,CTF1,EDN1,AMH,APBB3,IGF2,MMP2,SEMA6A,MC1R,CORO1A,SLITRK1,DDT,PRKCG,RAB3A,PRRT1,PRRX1,ARL2BP,SERPINE1,POU3F2,FOSB,RANGRF,WASHC1,MMP3,ACTA2,LRRC15,PTPN22,MEIS1,S1PR3,TCF4,PRRT2,TRPV4,BMP6,CLDN1,DPYSL3,KCNK2,MAMSTR,GPR35,HLX,PKDCC,LAMA1,PTHLH,DBH,TMEM198,CD177,GREM1,FOXA3,SPDYE12,KCNIP2,ELL3,CHD5,TPH1,NUSAP1,YY2,CRLF1,SRCIN1,COL3A1,EGR4,ARHGEF16,UCHL1,ESM1,SFRP1,SERPINB7,EBI3,PANX2,CDK5R2,GRIN2A,HLA-

			DOB,DUSP26,KCNE5,TBX6,MAOB,ADGRB3,TMEM119,RAPSN,KIAA0319,RBM20,PLAGL1,SPINK1,MIF,ENHO,LAG3,FNDC5,TNFRSF13C,THY1,ONECUT1,SOX7
GO:0007275	multicellular organism development	4.786691482749542e-7	*CCN1,MYADM,MT-CO1,MT-ND4,ANP32B,FOSL1,ATIC,PRKACA,BRD4,CCN3,SF1,HNRNPD,FKBP1A,ATN1,TYMS,FST,CHERP,ADM,CD44,ODC1,SLC9A1,ECM1,BRPF3,PPARD,CMIP,ZFP36L1,LAMB3,DLGAP4,PPDPF,LOX,BYSL,PDLIM7,PPP1R13L,PCGF2,DTNBP1,MAL2,PDCL3,SF3A2,NCOR2,DVL3,ADAMTS1,NPRL3,PLAGL2,BAK1,UPF3B,NCOA6,XAB2,CDKN1A,MYD88,SKI,GNG5,RHOJ,ZNF521,GSK3A,PBX2,CXCR4,UPF3A,UCP2,EIF2B4,CNOT3,TPP1,CC2D1A,ATF5,PHGDH,NRGN,RERE,PHACTR4,CCBE1,MEF2D,HMOX1,SETD1A,AFF3,EGR1,COL1A1,NGFR,NSRP1,TENM1,GPR137,ESRP2,GATA6,CREB3L1,STXBP1,DMD,NLGN2,RELN,ENO3,FURIN,CHRDL1,YJEFN3,CTH,LIMS2,ZBED3,RAB13,FAT3,SPHK1,SOX12,SYNGAP1,ID4,RGS4,EVI2B,ILK,FZD8,GLIS2,HSF4,TUBB3,KDM6B,GABRE,PAX8,TFAP2A,GPRC5B,CACNG7,NR1D1,ARC,PDE2A,ZFP36,CYP1A1,SIX2,ZNF296,BMPER,TNFSF12,PDGFD,S1PR1,GATA3,GATA2,LOXL3,TRNP1,CTF1,EDN1,AMH,IGF2,MMP2,SEMA6A,SLITRK1,PRKCG,RAB3A,PAK6,PRRX1,RAPGEFL1,ASIC1,GABRA3,SERPINE1,POU3F2,ACTA2,COL1A2,MEIS1,S1PR3,TCF4,TRPV4,BMP6,CLDN1,KIRREL3,PHACTR1,SYPL2,ANPEP,GPR15,DPYSL3,KCNK2,SNX10,INKA1,HLX,PKDCC,LAMA1,PTHLH,GREM1,ANGPTL6,SCO2,AMTN,NRXN3,BCHE,KCNIP2,ELL3,CHD5,HOXD1,CRLF1,GJB3,NELL2,SRCIN1,COL3A1,STIMATE-MUSTN1,WNK4,UCHL1,ESM1,IL1RN,SFRP1,GNRH2,SERPINB7,FLRT1,CDK5R2,TIE1,AATK,FOXO6,GRIN2A,PCDHB2,SERPINB5,CCDC154,HOXB8,PCDHB6,TBX6,MAOB,EPHA6,ADGRB3,TMEM119,KIAA0319,RBM20,BGN,ADAMTS5,LAG3,THY1,ONECUT1,DRGX,SLC25A34,SOX7*
GO:0048513	animal organ development	9.236849914486202e-7	*CCN1,MT-CO2,MT-CYB,MT-CO1,MT-ND4,ANP32B,FOSL1,ATIC,CCN3,SF1,HNRNPD,FKBP1A,ATN1,TYMS,FST,ADM,SLC29A1,CD44,ODC1,SLC9A1,ECM1,PPARD,ZFP36L1,LAMB3,PPDPF,LOX,PDLIM7,PPP1R13L,PCGF2,DTNBP1,BCL9L,NCOR2,ADAMTS1,NPRL3,BAK1,UPF3B,NCOA6,CDKN1A,MYD88,SKI,GNG5,RHOJ,GSK3A,PBX2,CXCR4,UCP2,EIF2B4,ATF5,PHGDH,RERE,PHACTR4,CCBE1,MEF2D,SETD1A,EGR1,COL1A1,NGFR,FHL3,PRR14,FOXQ1,ESRP2,GATA6,DMD,RELN,ENO3,CHRDL1,LIMS2,RAB13,FAT3,SPHK1,SOX12,ID4,ZNF703,RGS4,ILK,GLIS2,HSF4,KDM6B,LDOC1,PAX8,TFAP2A,PDE2A,ZFP36,CYP1A1,SIX2,BMPER,PDGFD,S1PR1,GATA3,GATA2,LOXL3,MYO7B,TRNP1,CTF1,EDN1,AMH,IGF2,MMP2,SEMA6A,RAB3A,PRRX1,SERPINE1,POU3F2,ACTA2,COL1A2,MEIS1,CYSRT1,TRPV4,BMP6,CLDN1,KIRREL3,PHACTR1,SYPL2,TGFBR3L,KCNK2,SNX10,HLX,PKDCC,LAMA1,PTHLH,GREM1,SCO2,AMTN,ELL3,CHD5,TPH1,CRLF1,GJB3,COL3A1,STIMATE-MUSTN1,WNK4,SFRP1,SERPINB7,CDK5R2,TIE1,KRT14,AATK,GRIN2A,CNFN,SERPINB5,CCDC154,HOXB8,TBX6,MAOB,TMEM119,TCHH,RBM20,BGN,ADAMTS5,ALOXE3,THY1,ONECUT1*
GO:0048731	system development	0.000001013687426093196	*CCN1,MT-CO1,MT-ND4,ANP32B,FOSL1,ATIC,PRKACA,CCN3,SF1,HNRNPD,FKBP1A,ATN1,TYMS,FST,CHERP,ADM,CD44,ODC1,SLC9A1,ECM1,PPARD,ZFP36L1,DLGAP4,PPDPF,LOX,PDLIM7,PPP1R13L,PCGF2,DTNBP1,MAL2,PDCL3,SF3A2,NCOR2,DVL3,ADAMTS1,NPRL3,BAK1,UPF3B*

			,NCOA6,CDKN1A,MYD88,SKI,GNG5,RHOJ,ZNF521,GSK3A,PBX2,CXCR4,UCP2,EIF2B4,TPP1,CC2D1A,ATF5,PHGDH,NRGN,RERE,PHACTR4,CCBE1,MEF2D,HMOX1,SETD1A,EGR1,COL1A1,NGFR,TENM1,ESRP2,GATA6,CREB3L1,STXBP1,DMD,NLGN2,RELN,ENO3,CHRDL1,YJEFN3,CTH,LIMS2,RAB13,FAT3,SPHK1,SOX12,SYNGAP1,ID4,RGS4,ILK,FZD8,GLIS2,HSF4,TUBB3,KDM6B,GABRE,PAX8,TFAP2A,GPRC5B,CACNG7,NR1D1,ARC,PDE2A,CYP1A1,SIX2,ZNF296,BMPER,TNFSF12,PDGFD,S1PR1,GATA3,GATA2,LOXL3,TRNP1,CTF1,EDN1,AMH,IGF2,MMP2,SEMA6A,SLITRK1,PRKCG,RAB3A,PAK6,PRRX1,RAPGEFL1,ASIC1,GABRA3,SERPINE1,POU3F2,ACTA2,COL1A2,MEIS1,TCF4,TRPV4,BMP6,CLDN1,KIRREL3,PHACTR1,SYPL2,ANPEP,GPR15,DPYSL3,KCNK2,SNX10,INKA1,HLX,PKDCC,LAMA1,PTHLH,GREM1,ANGPTL6,SCO2,NRXN3,BCHE,KCNIP2,ELL3,CHD5,HOXD1,CRLF1,NELL2,SRCIN1,COL3A1,STIMATE-MUSTN1,WNK4,UCHL1,ESM1,SFRP1,SERPINB7,FLRT1,CDK5R2,TIE1,AATK,FOXO6,GRIN2A,PCDHB2,SERPINB5,CCDC154,HOXB8,PCDHB6,TBX6,MAOB,EPHA6,ADGRB3,TMEM119,KIAA0319,RBM20,BGN,ADAMTS5,THY1,ONECUT1,DRGX
GO:0048518	positive regulation of biological process	0.0000024345656207759586	FUS,CCN1,EIF1,MYADM,ANP32B,PABPC1,RIPOR1,FOSL1,PRKACA,MAT2A,SF3B4,CTDNEP1,S100A11,BRD4,CCN3,HNRNPD,FKBP1A,TAF15,PRPF19,PXN,ATN1,PTPN23,HNRNPA2B1,AGPAT1,FST,RAB7A,CIZ1,CHERP,ADM,KHSRP,EHD2,CD44,ODC1,SLC9A1,ECM1,BRPF3,PPARD,ZFP36L1,CCDC124,PCBP1,MDC1,WIZ,MAPK13,PPP1R13L,C4BPB,CRTC2,DTNBP1,PSMC1,PDCL3,BCL9L,SF3A2,SAMD4B,DVL3,ADAMTS1,NPRL3,PLAGL2,BAK1,UPF3B,NFIX,MED15,NFIC,NCOA6,VAMP2,ZMIZ2,PCIF1,CDKN1A,TP53INP2,MYD88,MCOLN1,SKI,GNG5,MAP2K7,CXXC1,RHOJ,ZNF521,GSK3A,PBX2,CAMTA2,CXCR4,UPF3A,UCP2,EIF2B4,CNOT3,CRTC1,CC2D1A,ATF5,SORBS3,ZC3H3,RERE,GATAD2B,DMAP1,CCBE1,PABPC1L,MEF2D,HMOX1,EGR1,FAM98B,COL1A1,GABARAP,NGFR,TENM1,GPR137,LSM10,ZNF580,ESRP2,GATA6,CREB3L1,SCAMP5,STXBP1,EIF3C,CAMK2N1,DMD,NLGN2,PLK3,RELN,MMP1,CCNK,FURIN,PEAR1,EIF5AL1,DOK3,CTH,GPR3,PRR16,LIMS2,ZNF385A,ZBED3,MLLT11,DAPK1,MRNIP,SPHK1,PLEKHN1,SOX12,ID4,ZNF703,RGS4,EVI2B,ILK,BICRA,GLIS2,NRK,GAREM1,KDM6B,FABP5,PAX8,MAGEC2,TFAP2A,TNFSF15,GPRC5B,CACNG7,NR1D1,ARC,PDE2A,ZFP36,CYP1A1,SYT11,SIX2,BMPER,TNFSF12,PDGFD,S1PR1,HSPB8,ARID3B,ATP6V0C,HMGN5,GATA3,GATA2,IKZF4,LOXL3,SUPT4H1,DHPS,RFXAP,CTF1,EDN1,AMH,APBB3,IGF2,MMP2,SEMA6A,MC1R,CORO1A,SLITRK1,DDT,PRKCG,RAB3A,PRRT1,PRRX1,ARL2BP,SERPINE1,POU3F2,FOSB,RANGRF,WASHC1,MMP3,ACTA2,LRRC15,PTPN22,MEIS1,S1PR3,TCF4,PRRT2,TRPV4,BMP6,CLDN1,DPYSL3,KCNK2,MAMSTR,GPR35,HLX,PKDCC,LAMA1,PTHLH,DBH,TMEM198,CD177,GREM1,FOXA3,AMTN,SPDYE12,KCNIP2,ELL3,CHD5,TPH1,NUSAP1,YY2,CRLF1,SRCIN1,COL3A1,EGR4,ARHGEF16,UCHL1,ESM1,SFRP1,SERPINB7,EBI3,PANX2,CDK5R2,TIE1,FOXO6,GRIN2A,HLA-DOB,DUSP26,KCNE5,TBX6,MAOB,ADGRB3,TMEM119,RAPSN,KIAA0319,TMED7-TICAM2,RBM20,PLAGL1,SPINK1,MIF,ENHO,LAG3,FNDC5,TNFRSF13C,THY1,ONECUT1,SOX7
GO:0048	anatomical	0.00000723	CCN1,MYADM,MT-CO2,MT-CYB,MT-CO1,MT-

856	structure development	4240930139349	*ND4,ANP32B,HNRNPH3,FOSL1,ATIC,PRKACA,CTDNEP1,BRD4,CCN3,SF1,HNRNPD,FKBP1A,DIAPH1,ATN1,TYMS,FST,CHERP,ADM,EHD2,SLC29A1,CD44,ODC1,SLC9A1,ECM1,BRPF3,PPARD,CMIP,ZFP36L1,LAMB3,DLGAP4,PPDPF,LOX,BYSL,PDLIM7,PPP1R13L,PCGF2,DTNBP1,MAL2,PDCL3,BCL9L,SF3A2,NCOR2,DVL3,ADAMTS1,NPRL3,PLAGL2,BAK1,UPF3B,NCOA6,XAB2,CDKN1A,MYD88,SKI,GNG5,RHOJ,ZNF521,GSK3A,PBX2,CXCR4,UPF3A,UCP2,EIF2B4,CNOT3,TPP1,CC2D1A,ATF5,PHGDH,NRGN,RERE,PHACTR4,ALDOA,CCBE1,PABPC1L,MEF2D,HMOX1,SETD1A,AFF3,EGR1,COL1A1,NGFR,FHL3,SYVN1,NSRP1,TENM1,PRR14,FOXQ1,GPR137,ESRP2,GATA6,CREB3L1,STXBP1,DMD,NLGN2,RELN,ENO3,FURIN,CHRDL1,YJEFN3,CTH,LIMS2,ZNF385A,ZBED3,RAB13,FAT3,SPHK1,SOX12,SYNGAP1,ID4,ZNF703,RGS4,EVI2B,ILK,FZD8,GLIS2,HSF4,TUBB3,KDM6B,GABRE,FABP5,LDOC1,PAX8,TFAP2A,GPRC5B,CACNG7,NR1D1,ARC,PDE2A,ZFP36,CYP1A1,SIX2,ZNF296,BMPER,TNFSF12,PDGFD,S1PR1,GATA3,GATA2,LOXL3,MYO7B,TRNP1,CTF1,EDN1,AMH,IGF2,MMP2,SEMA6A,CORO1A,SLITRK1,PRKCG,RAB3A,CCDC78,PAK6,PRRX1,RAPGEFL1,ASIC1,GABRA3,SERPINE1,POU3F2,CDH19,ACTA2,PTPN22,COL1A2,MEIS1,S1PR3,TCF4,CYSRT1,TRPV4,BMP6,CLDN1,KIRREL3,PHACTR1,SYPL2,ANPEP,TGFBR3L,GPR15,DPYSL3,KCNK2,SNX10,MAMSTR,INKA1,ANKLE1,HLX,PKDCC,LAMA1,PTHLH,GREM1,FOXA3,ANGPTL6,SCO2,AMTN,NRXN3,BCHE,KCNIP2,ELL3,CHD5,TPH1,HOXD1,CRLF1,GJB3,NELL2,SRCIN1,FOXD4,COL3A1,STIMATE-MUSTN1,FOXD4L1,WNK4,UCHL1,ESM1,IL1RN,SFRP1,GNRH2,SERPINB7,FLRT1,CDK5R2,TIE1,KRT14,AATK,FOXO6,GRIN2A,PCDHB2,CNFN,SERPINB5,SULT1B1,CCDC154,HOXB8,PCDHB6,TBX6,MAOB,EPHA6,ADGRB3,TMEM190,TMEM119,KIAA0319,TCHH,RBM20,BGN,ADAMTS5,SPINK1,ALOXE3,LAG3,THY1,ONECUT1,DRGX,SLC25A34,SOX7*
GO:0072359	circulatory system development	0.00000805185378785 0731	*CCN1,ANP32B,FOSL1,CCN3,FKBP1A,ADM,SLC9A1,ECM1,ZFP36L1,LOX,PDLIM7,PPP1R13L,PDCL3,ADAMTS1,NPRL3,BAK1,NCOA6,CDKN1A,SKI,GNG5,RHOJ,GSK3A,CXCR4,CCBE1,MEF2D,HMOX1,EGR1,COL1A1,NGFR,GATA6,CREB3L1,YJEFN3,CTH,SPHK1,RGS4,ILK,FZD8,KDM6B,PAX8,PDE2A,BMPER,TNFSF12,PDGFD,S1PR1,GATA3,GATA2,EDN1,MMP2,SEMA6A,PRRX1,SERPINE1,ACTA2,COL1A2,MEIS1,SYPL2,ANPEP,GPR15,KCNK2,LAMA1,GREM1,ANGPTL6,NRXN3,COL3A1,ESM1,SFRP1,SERPINB7,TIE1,TBX6,ADGRB3,RBM20,ADAMTS5,THY1*
GO:0032502	developmental process	0.0000361671764710 4424	*CCN1,MYADM,MT-CO2,MT-CYB,MT-CO1,MT-ND4,RPS3A,ANP32B,HNRNPH3,FOSL1,ATIC,PRKACA,CTDNEP1,BRD4,CCN3,SF1,HNRNPD,FKBP1A,DIAPH1,ATN1,TYMS,FST,CHERP,ADM,EHD2,SLC29A1,CD44,ODC1,SLC9A1,ECM1,BRPF3,PPARD,CMIP,ZFP36L1,LAMB3,DLGAP4,PPDPF,LOX,BYSL,PDLIM7,PPP1R13L,PCGF2,DTNBP1,MAL2,PDCL3,BCL9L,SF3A2,NCOR2,DVL3,ADAMTS1,NPRL3,PLAGL2,BAK1,UPF3B,MED15,NCOA6,XAB2,CDKN1A,TP53INP2,MYD88,SKI,GNG5,RHOJ,ZNF521,SNRPA1,GSK3A,PBX2,CXCR4,UPF3A,UCP2,EIF2B4,CNOT3,TPP1,CC2D1A,ATF5,PHGDH,NRGN,RERE,PHACTR4,GATAD2B,ALDOA,CCBE1,PABPC1L,MEF2D,HMOX1,SETD1A,AFF3,EGR1,COL1A1,NGFR,FHL3,SYVN1,NSRP1,TENM1,PRR14,FOXQ1,GPR137,ESRP2,GATA6,CREB3L1,STXBP1,DMD,NLGN2,RELN,ENO3,FURIN,CHRDL1,YJEFN3,CTH,NHSL3,*

			LIMS2,ZNF385A,ZBED3,RAB13,FAT3,SPHK1,OSGIN1,SOX12,SYNGAP1,ID4,ZNF703,RGS4,EVI2B,ILK,BICRA,FZD8,GLIS2,HSF4,TUBB3,COX7B2,KDM6B,GABRE,FABP5,LDOC1,PAX8,TFAP2A,GPRC5B,CACNG7,NR1D1,ARC,PDE2A,ZFP36,CYP1A1,SIX2,ZNF296,BMPER,TNFSF12,PDGFD,S1PR1,ATP6V0C,GATA3,GATA2,LOXL3,MYO7B,TRNP1,CTF1,EDN1,AMH,IGF2,MMP2,SEMA6A,CORO1A,SLITRK1,PRKCG,RAB3A,CCDC78,PAK6,PRRX1,RAPGEFL1,ASIC1,GABRA3,SERPINE1,POU3F2,CDH19,ACTA2,PTPN22,COL1A2,MEIS1,S1PR3,TCF4,CYSRT1,TRPV4,BMP6,CLDN1,KIRREL3,BCL2L2-PABPN1,PHACTR1,SYPL2,ANPEP,TGFBR3L,GPR15,DPYSL3,KCNK2,SNX10,MAMSTR,INKA1,ANKLE1,HLX,PKDCC,LAMA1,PTHLH,GREM1,FOXA3,ANGPTL6,SCO2,AMTN,NRXN3,BCHE,KCNIP2,ELL3,CHD5,TPH1,HOXD1,CRLF1,GJB3,NELL2,SRCIN1,FOXD4,COL3A1,STIMATE-MUSTN1,FOXD4L1,WNK4,UCHL1,ESM1,IL1RN,SFRP1,ATP6V1G2,GNRH2,SERPINB7,FLRT1,CNBD2,CDK5R2,TIE1,KRT14,AATK,FOXO6,GRIN2A,PCDHB2,CNFN,SERPINB5,SULT1B1,CCDC154,HOXB8,PCDHB6,TBX6,MAOB,EPHA6,DLX4,ADGRB3,TMEM190,TMEM119,KIAA0319,TCHH,RBM20,BGN,ADAMTS5,SPINK1,ALOXE3,LAG3,FNDC5,THY1,ONECUT1,DRGX,SLC25A34,SOX7
GO:0001944	vasculature development	0.000041303864968600 9	*CCN1,ANP32B,FOSL1,CCN3,ADM,ECM1,ZFP36L1,LOX,PDCL3,ADAMTS1,NPRL3,BAK1,RHOJ,CXCR4,CCBE1,HMOX1,EGR1,COL1A1,NGFR,GATA6,CREB3L1,YJEFN3,CTH,SPHK1,FZD8,PDE2A,BMPER,TNFSF12,PDGFD,S1PR1,GATA2,EDN1,MMP2,SEMA6A,PRRX1,SERPINE1,ACTA2,COL1A2,MEIS1,ANPEP,GPR15,LAMA1,GREM1,ANGPTL6,NRXN3,COL3A1,ESM1,SFRP1,SERPINB7,TIE1,TBX6,ADGRB3,THY1*
GO:0009888	tissue development	0.00004328113183738 4184	*CCN1,MYADM,HNRNPH3,PRKACA,CTDNEP1,CCN3,FKBP1A,TYMS,FST,ADM,CD44,SLC9A1,ECM1,PPARD,ZFP36L1,LAMB3,LOX,PPP1R13L,BCL9L,NPRL3,CDKN1A,MYD88,SKI,GSK3A,CXCR4,TPP1,PHGDH,PHACTR4,MEF2D,EGR1,COL1A1,NGFR,FOXQ1,ESRP2,GATA6,DMD,RAB13,ID4,ZNF703,RGS4,ILK,HSF4,TUBB3,KDM6B,FABP5,PAX8,TFAP2A,ARC,PDE2A,ZFP36,CYP1A1,SIX2,BMPER,PDGFD,S1PR1,GATA3,GATA2,LOXL3,EDN1,AMH,IGF2,MMP2,SEMA6A,CCDC78,PRRX1,SERPINE1,POU3F2,ACTA2,COL1A2,MEIS1,S1PR3,CYSRT1,TRPV4,BMP6,CLDN1,TGFBR3L,KCNK2,SNX10,INKA1,HLX,PKDCC,LAMA1,PTHLH,GREM1,AMTN,ELL3,CRLF1,COL3A1,STIMATE-MUSTN1,WNK4,SFRP1,SERPINB7,TIE1,KRT14,CNFN,SERPINB5,SULT1B1,CCDC154,TBX6,TMEM119,TCHH,BGN,ADAMTS5,ALOXE3,ONECUT1,SOX7*
GO:0080090	regulation of primary metabolic process	0.00004522563121600095	*FUS,CCN1,EIF5B,EIF1,PABPC1,HNRNPH3,FOSL1,UTP4,PRKACA,SF3B4,CTDNEP1,S100A11,BRD4,SF1,HNRNPD,FKBP1A,TAF15,PRPF19,USP14,ATN1,HNRNPA2B1,TYMS,FST,RAB7A,CIZ1,ADM,KHSRP,PSMD2,FOXP4,AKT1S1,CD44,ODC1,SLC9A1,ECM1,BRPF3,PPARD,ZFP36L1,CCDC124,PCBP1,CDA,MDC1,WIZ,LOX,PPP1R13L,PCGF2,C4BPB,CRTC2,PSMC1,PDCL3,SRRM1,BCL9L,NCOR2,SAMD4B,DVL3,ZNF114,PLAGL2,BAK1,UPF3B,NFIX,MED15,NFIC,NCOA6,ZMIZ2,PCIF1,CDKN1A,TP53INP2,MYD88,SKI,MAP2K7,CXXC1,ZNF521,GSK3A,SCAF4,SP2,PBX2,CAMTA2,DUSP7,UPF3A,EIF2B4,CNOT3,RPS26,CRTC1,CC2D1A,ATF5,SORBS3,CDK11B,RERE,GATAD2B,BCORL1,DMAP1,CCBE1,MEF2D,HMOX1,AFF3,EGR1,COL1A1,GABARAP,*

			NGFR,NSRP1,TENM1,FOXQ1,ZNF580,ESRP2,GATA6,SERPINB8,CREB3L1,EIF3C,DMD,PLK3,RELN,CCNK,FURIN,EIF5AL1,PRR16,GLI4,ZNF385A,ZBED3,MLLT11,DAPK1,MRNIP,SPHK1,ZBTB12,PLEKHN1,MAGEC1,SOX12,ID4,ZNF703,MNT,ILK,BICRA,GLIS2,ZNF589,HSF4,KDM6B,FABP5,PAX8,MAGEC2,TFAP2A,TNFSF15,GPRC5B,CACNG7,NR1D1,PDE2A,ZFP36,SIX2,SP140,ZNF296,TNFSF12,PDGFD,S1PR1,ARID3B,TRIM66,HMGN5,GATA3,GATA2,IKZF4,LOXL3,SUPT4H1,RFXAP,CTF1,EDN1,APBB3,IGF2,CDK11A,MC1R,ZNF414,PRKCG,PAK6,PRRX1,ARL2BP,SERPINE1,POU3F2,LIN37,FOSB,PARP15,WASHC1,PTPN22,CST4,MEIS1,TCF4,TRPV4,BMP6,KCNK2,MAMSTR,SETBP1,ANKLE1,HLX,GNMT,GREM1,FOXA3,ZNF257,SPDYE12,ELL3,CHD5,YY2,HOXD1,CRLF1,SRCIN1,ZNF492,FOXD4,SAMSN1,NELFCD,FOXD4L1,EGR4,GPR146,UCHL1,SFRP1,PCSK1N,CDK5R2,FOXO6,GRIN2A,DUSP26,HOXB8,TBX6,MAOB,DLX4,ZNF716,TMEM119,RBM20,PLAGL1,SPINK1,MIF,THY1,ONECUT1,DRGX,SOX7
GO:0001568	blood vessel development	0.0000731906580155279 3	*CCN1,FOSL1,CCN3,ADM,ECM1,ZFP36L1,LOX,PDCL3,ADAMTS1,NPRL3,BAK1,RHOJ,CXCR4,CCBE1,HMOX1,EGR1,COL1A1,NGFR,GATA6,CREB3L1,YJEFN3,SPHK1,FZD8,PDE2A,BMPER,TNFSF12,PDGFD,S1PR1,GATA2,EDN1,MMP2,SEMA6A,PRRX1,SERPINE1,ACTA2,COL1A2,MEIS1,ANPEP,GPR15,LAMA1,GREM1,ANGPTL6,NRXN3,COL3A1,ESM1,SFRP1,SERPINB7,TIE1,TBX6,ADGRB3,THY1*
GO:0045935	positive regulation of nucleobase-containing compound metabolic process	0.0000919213107391061 8	*FUS,CCN1,EIF1,PABPC1,FOSL1,SF3B4,BRD4,HNRNPD,TAF15,PRPF19,ATN1,HNRNPA2B1,CIZ1,KHSRP,SLC9A1,BRPF3,PPARD,ZFP36L1,CCDC124,PCBP1,MDC1,WIZ,CRTC2,BCL9L,SAMD4B,DVL3,PLAGL2,UPF3B,NFIX,MED15,NFIC,NCOA6,ZMIZ2,CDKN1A,TP53INP2,MYD88,SKI,MAP2K7,CXXC1,ZNF521,GSK3A,PBX2,CAMTA2,UPF3A,CNOT3,CRTC1,ATF5,RERE,GATAD2B,DMAP1,MEF2D,EGR1,COL1A1,NGFR,ZNF580,GATA6,CREB3L1,CCNK,ZBED3,MLLT11,MRNIP,PLEKHN1,SOX12,ID4,ILK,BICRA,GLIS2,KDM6B,PAX8,TFAP2A,NR1D1,ZFP36,SIX2,TNFSF12,S1PR1,ARID3B,HMGN5,GATA3,GATA2,IKZF4,SUPT4H1,RFXAP,EDN1,IGF2,MC1R,PRKCG,PRRX1,ARL2BP,POU3F2,FOSB,MEIS1,TCF4,BMP6,MAMSTR,GREM1,FOXA3,ELL3,YY2,EGR4,UCHL1,SFRP1,TBX6,PLAGL1,ONECUT1,SOX7*
GO:0009653	anatomical structure morphogenesis	0.0001311379880135162 4	*CCN1,MYADM,PRKACA,CCN3,FKBP1A,DIAPH1,FST,ADM,EHD2,CD44,ECM1,ZFP36L1,LAMB3,LOX,BYSL,PPP1R13L,PCGF2,DTNBP1,PDCL3,BCL9L,DVL3,ADAMTS1,NPRL3,BAK1,SKI,GNG5,RHOJ,GSK3A,PBX2,CXCR4,CNOT3,CC2D1A,RERE,PHACTR4,ALDOA,CCBE1,MEF2D,HMOX1,AFF3,COL1A1,NGFR,FOXQ1,ESRP2,GATA6,CREB3L1,STXBP1,DMD,RELN,FURIN,YJEFN3,LIMS2,ZNF385A,FAT3,SPHK1,SYNGAP1,ID4,ILK,FZD8,TUBB3,KDM6B,PAX8,TFAP2A,CACNG7,ARC,SIX2,BMPER,TNFSF12,S1PR1,GATA3,GATA2,TRNP1,EDN1,AMH,IGF2,MMP2,SEMA6A,CORO1A,SLITRK1,RAB3A,PAK6,PRRX1,SERPINE1,POU3F2,CDH19,ACTA2,COL1A2,MEIS1,S1PR3,TRPV4,BMP6,KIRREL3,PHACTR1,ANPEP,GPR15,SNX10,HLX,PKDCC,LAMA1,GREM1,FOXA3,ANGPTL6,AMTN,NRXN3,NELL2,SRCIN1,FOXD4,COL3A1,FOXD4L1,WNK4,UCHL1,ESM1,IL1RN,SFRP1,FLRT1,CDK5R2,TIE1,SERPINB5,CCDC154,HOXB8,TBX6,EPHA6,ADGRB3,TMEM119,KIAA0319,RBM20,ADAMTS5,THY1,ONECUT1,DRGX,SOX7*
GO:0009	positive	0.00013170	*FUS,CCN1,EIF1,PABPC1,FOSL1,PRKACA,SF3B4,CTDNEP1,BRD4,HNRNPD,TAF15,PRPF19,ATN1,HNRNPA*

893	regulation of metabolic process	61081044091	*2B1,AGPAT1,RAB7A,CIZ1,ADM,KHSRP,CD44,SLC9A1,BRPF3,PPARD,ZFP36L1,CCDC124,PCBP1,MDC1,WIZ,MAPK13,C4BPB,CRTC2,DTNBP1,PSMC1,PDCL3,BCL9L,SAMD4B,DVL3,NPRL3,PLAGL2,BAK1,UPF3B,NFIX,MED15,NFIC,NCOA6,ZMIZ2,PCIF1,CDKN1A,TP53INP2,MYD88,SKI,MAP2K7,CXXC1,ZNF521,GSK3A,PBX2,CAMTA2,CXCR4,UPF3A,UCP2,CNOT3,CRTC1,ATF5,RERE,GATAD2B,DMAP1,CCBE1,PABPC1L,MEF2D,HMOX1,EGR1,FAM98B,COL1A1,GABARAP,NGFR,TENM1,ZNF580,GATA6,CREB3L1,SCAMP5,EIF3C,PLK3,RELN,CCNK,FURIN,EIF5AL1,GPR3,PRR16,ZBED3,MLLT11,DAPK1,MRNIP,SPHK1,PLEKHN1,SOX12,ID4,ILK,BICRA,GLIS2,KDM6B,FABP5,PAX8,MAGEC2,TFAP2A,TNFSF15,GPRC5B,NR1D1,PDE2A,ZFP36,SIX2,TNFSF12,S1PR1,HSPB8,ARID3B,HMGN5,GATA3,GATA2,IKZF4,SUPT4H1,RFXAP,CTF1,EDN1,AMH,IGF2,MC1R,DDT,PRKCG,PRRX1,ARL2BP,SERPINE1,POU3F2,FOSB,ACTA2,PTPN22,MEIS1,TCF4,TRPV4,BMP6,MAMSTR,DBH,CD177,GREM1,FOXA3,SPDYE12,ELL3,YY2,CRLF1,SRCIN1,EGR4,UCHL1,SFRP1,SERPINB7,EBI3,PANX2,TBX6,MAOB,TMEM119,RBM20,PLAGL1,MIF,ONECUT1,SOX7*
GO:0051254	positive regulation of RNA metabolic process	0.0001979374144704923 8	*FUS,CCN1,EIF1,PABPC1,FOSL1,SF3B4,BRD4,HNRNPD,TAF15,PRPF19,ATN1,KHSRP,SLC9A1,PPARD,ZFP36L1,CCDC124,PCBP1,MDC1,CRTC2,BCL9L,SAMD4B,DVL3,PLAGL2,UPF3B,NFIX,MED15,NFIC,NCOA6,ZMIZ2,TP53INP2,MYD88,SKI,MAP2K7,CXXC1,ZNF521,GSK3A,PBX2,CAMTA2,UPF3A,CNOT3,CRTC1,ATF5,RERE,GATAD2B,DMAP1,MEF2D,EGR1,COL1A1,NGFR,ZNF580,GATA6,CREB3L1,CCNK,ZBED3,MLLT11,PLEKHN1,SOX12,ID4,ILK,BICRA,GLIS2,KDM6B,PAX8,TFAP2A,NR1D1,ZFP36,SIX2,S1PR1,ARID3B,HMGN5,GATA3,GATA2,IKZF4,SUPT4H1,RFXAP,EDN1,IGF2,MC1R,PRRX1,ARL2BP,POU3F2,FOSB,MEIS1,TCF4,BMP6,MAMSTR,GREM1,FOXA3,ELL3,YY2,EGR4,SFRP1,TBX6,PLAGL1,ONECUT1,SOX7*
GO:0050896	response to stimulus	0.0002919703270115166	*FUS,RPS16,CCN1,MYADM,MT-CO2,MT-CYB,MT-ND2,MT-ND5,MT-CO1,MT-ND3,MT-ND4,MT-ND1,MT-ATP6,FTH1,MT-ND6,ARHGDIA,RIPOR1,SRM,FOSL1,ATIC,MT2A,PRKACA,MAT2A,CTDNEP1,S100A11,BRD4,CCN3,HNRNPD,FKBP1A,DIAPH1,PRPF19,USP14,PXN,ATN1,MAP1LC3B,RAB11B,AGPAT1,TYMS,FST,RAB7A,CHERP,ADM,POLR2A,KHSRP,AKT1S1,NUDC,SLC29A1,CD44,ODC1,SLC9A1,PPP1R11,ECM1,PPARD,ZFP36L1,CDA,MDC1,PPDPF,LOX,ZYX,MAPK13,PPP1R13L,PCGF2,C4BPB,DTNBP1,TONSL,BCL9L,VAC14,NCOR2,DVL3,ADAMTS1,NPRL3,SLAMF7,PLAGL2,BAK1,NCOA6,XAB2,PDE4A,VAMP2,GPX3,CDKN1A,MYD88,MCOLN1,SKI,GNG5,MAP2K7,RHOJ,PDE1C,GSK3A,SP2,PRSS3,CAMTA2,DUSP7,CXCR4,UCP2,EIF2B4,CRTC1,CC2D1A,SORBS3,ZC3H3,NRGN,PHACTR4,DMAP1,CCBE1,HMOX1,SETD1A,AFF3,EGR1,COL1A1,GABARAP,NGFR,SYVN1,TENM1,GPR137,ST3GAL6,DTNA,ZNF580,GATA6,CREB3L1,SCAMP5,STXBP1,CAMK2N1,DMD,NLGN2,PLK3,RELN,MMP1,CCNK,LTB4R,FURIN,PEAR1,GSTM1,CHRDL1,SLC39A4,YJEFN3,DOK3,CTH,GPR3,RTKN,LIMS2,DOCK6,PORCN,ZNF385A,ZBED3,RAB13,MLLT11,DAPK1,MRNIP,SPHK1,OSGIN1,PLEKHN1,TAX1BP3,SYNGAP1,SLC29A3,ZNF703,RIN3,RGS4,MNT,ILK,NAPRT,FZD8,GLIS2,TUBB3,NRK,GAREM1,KDM6B,FABP5,ARHGEF19,LDOC1,PAX8,TFAP2A,TNFSF15,GPRC5B,PSD4,CACNG7,NR1D1,ARC,PDE2A,ZFP36,CYP1A1,SYT11,SP140,BMPER,TNFSF12,PDGFD,ARSA,S1PR1*

			,LRRC2,HSPB8,LTB4R2,FNDC4,ATP6V0C,GATA3,GATA2,LOXL3,SCNN1D,CNKSR2,KCNK1,NCF2,TTC4,RPP21,CTF1,EDN1,AMH,IGF2,MMP2,SEMA6A,MC1R,CORO1A,DDT,PRKCG,GCKR,RAB3A,SERPINB2,GGT7,PAK6,ADGRE1,PRRT1,PRRX1,ARL2BP,SORCS3,RAPGEFL1,ASIC1,SERPINE1,SLC52A3,FAM83A,FOSB,AOC2,CST1,GNG10,RHBDL1,MMP3,ACTA2,LRRC15,PTPN22,COL1A2,CST4,S1PR3,TRPV4,BMP6,CYP2J2,CLDN1,BCL2L2-PABPN1,ASB16,TGFBR3L,GPR15,DPYSL3,KCNK2,OR2B6,SNX10,RNASEH2A,GPR132,GPR35,GNG13,TRBV12-4,ANKLE1,HLX,VWCE,LAMA1,PTHLH,DBH,TMEM198,CD177,AOC3,GREM1,FOXA3,SCO2,OLR1,NRXN3,MAP1LC3B2,BCHE,TAS2R5,KCNIP2,ATP1A3,TNFRSF25,ELL3,LILRA6,CHD5,TPH1,CRLF1,GJB3,RCAN2,VSTM1,COL3A1,SAMSN1,WNK4,GPR146,ARHGEF16,UCHL1,ESM1,IL1RN,SFRP1,GNRH2,EBI3,ARHGAP20,PANX2,FLRT1,UCP3,PCSK1N,TIE1,KRT14,GTF2H4,PLCXD3,GRIN2A,HSD11B2,HLA-DOB,HCN2,CGB7,SULT1B1,DUSP26,TOP3B,CPT1B,HUS1B,TBX6,MAOB,EPHA6,LYG1,GRIN3B,MXRA5,ADGRB3,KIAA0319,TMED7-TICAM2,ADAMTS5,GPR87,SPINK1,MIF,ENHO,ALOXE3,LAG3,FNDC5,GKN2,TNFRSF13C,GPRC5D,THY1,ONECUT1,NPFF,DRGX,CGB8,SOX7
GO:0051384	response to glucocorticoid	0.00038208572292512005	*MT-ND3,FOSL1,TYMS,ADM,ZFP36L1,CDKN1A,GSK3A,UCP2,ZFP36,EDN1,FOSB,BMP6,CLDN1,BCHE,IL1RN,UCP3,HSD11B2,MAOB*
GO:0008284	positive regulation of cell population proliferation	0.0003987070971584863	*CCN1,FOSL1,ADM,ODC1,ECM1,PPARD,PDCL3,ADAMTS1,CDKN1A,MYD88,GNG5,MEF2D,HMOX1,EGR1,FAM98B,NGFR,ZNF580,ESRP2,GATA6,NLGN2,SPHK1,ID4,ZNF703,ILK,BICRA,GAREM1,SIX2,TNFSF12,PDGFD,S1PR1,HMGN5,GATA2,DHPS,CTF1,EDN1,IGF2,MMP2,CORO1A,PRRX1,POU3F2,PTPN22,MEIS1,S1PR3,BMP6,CLDN1,HLX,PTHLH,GREM1,ELL3,CRLF1,EGR4,ESM1,SFRP1,SERPINB7,EBI3,TMEM119,SPINK1,MIF,TNFRSF13C*
GO:0009628	response to abiotic stimulus	0.00042906193037960306	*MT-CO2,MT-CYB,MT-ND2,MT-ND5,MT-CO1,MT-ND3,MT-ND4,MT-ND1,MT-ATP6,FOSL1,PRKACA,HNRNPD,RAB11B,ADM,POLR2A,SLC29A1,SLC9A1,PPARD,ZFP36L1,MAPK13,BAK1,CDKN1A,MYD88,MCOLN1,MAP2K7,CXCR4,UCP2,EIF2B4,CRTC1,HMOX1,EGR1,COL1A1,NGFR,GATA6,DMD,PLK3,MMP1,MRNIP,PLEKHN1,SYNGAP1,PDE2A,CYP1A1,ARSA,GATA3,SCNN1D,EDN1,MMP2,MC1R,RAB3A,ASIC1,SLC52A3,FOSB,MMP3,TRPV4,BMP6,KCNK2,DBH,COL3A1,SFRP1,UCP3,PCSK1N,KRT14,GRIN2A,HSD11B2,CPT1B,KIAA0319,DRGX*
GO:0010604	positive regulation of macromolecule metabolic process	0.0006313477426577313	*FUS,CCN1,EIF1,PABPC1,FOSL1,SF3B4,BRD4,HNRNPD,TAF15,PRPF19,ATN1,HNRNPA2B1,AGPAT1,RAB7A,CIZ1,KHSRP,CD44,SLC9A1,BRPF3,PPARD,ZFP36L1,CCDC124,PCBP1,MDC1,WIZ,MAPK13,C4BPB,CRTC2,DTNBP1,PSMC1,PDCL3,BCL9L,SAMD4B,DVL3,PLAGL2,BAK1,UPF3B,NFIX,MED15,NFIC,NCOA6,ZMIZ2,PCIF1,CDKN1A,TP53INP2,MYD88,SKI,MAP2K7,CXXC1,ZNF521,GSK3A,PBX2,CAMTA2,UPF3A,CNOT3,CRTC1,ATF5,RERE,GATAD2B,DMAP1,CCBE1,PABPC1L,MEF2D,HMOX1,EGR1,FAM98B,COL1A1,GABARAP,NGFR,TENM1,ZNF580,GATA6,CREB3L1,SCAMP5,EIF3C,PLK3,RELN,CCNK,FURIN,EIF5AL1,PRR16,ZBED3,MLLT11,MRNIP,SPHK1,PLEKHN1,SOX12,ID4,ILK,*

			BICRA,GLIS2,KDM6B,PAX8,MAGEC2,TFAP2A,TNFSF15,GPRC5B,NR1D1,PDE2A,ZFP36,SIX2,TNFSF12,S1PR1,ARID3B,HMGN5,GATA3,GATA2,IKZF4,SUPT4H1,RFXAP,CTF1,EDN1,AMH,IGF2,MC1R,DDT,PRKCG,PRRX1,ARL2BP,SERPINE1,POU3F2,FOSB,ACTA2,PTPN22,MEIS1,TCF4,TRPV4,BMP6,MAMSTR,GREM1,FOXA3,SPDYE12,ELL3,YY2,CRLF1,SRCIN1,EGR4,SFRP1,SERPINB7,EBI3,PANX2,TBX6,TMEM119,RBM20,PLAGL1,MIF,ONECUT1,SOX7
GO:0061448	connective tissue development	0.0006643731145978047	CCN1,CCN3,TYMS,CD44,ECM1,PPARD,LOX,MEF2D,EGR1,COL1A1,DMD,ID4,SIX2,PDGFD,GATA3,EDN1,PRRX1,ACTA2,TRPV4,BMP6,PKDCC,PTHLH,GREM1,COL3A1,STIMATE-MUSTN1,SERPINB7,BGN
GO:1902600	proton transmembrane transport	0.000742211164767534	MT-CO2,MT-CYB,MT-ND2,MT-ND5,MT-CO1,MT-ND3,MT-ND4,MT-ND1,MT-ATP6,MT-ND4L,MT-ND6,MT-ATP8,MT-CO3,SLC9A1,UCP2,SLC9A5,ATP6V0C,RNASEK,ATP1A3,ATP6V1G2,UCP3
GO:0031960	response to corticosteroid	0.0007946561980595567	MT-ND3,FOSL1,TYMS,ADM,ZFP36L1,CDKN1A,GSK3A,UCP2,ZFP36,SCNN1D,EDN1,FOSB,BMP6,CLDN1,BCHE,IL1RN,UCP3,HSD11B2,MAOB
GO:0019219	regulation of nucleobase-containing compound metabolic process	0.0014387866079352775	FUS,CCN1,EIF1,PABPC1,FOSL1,UTP4,PRKACA,SF3B4,S100A11,BRD4,SF1,HNRNPD,TAF15,PRPF19,ATN1,HNRNPA2B1,FST,CIZ1,KHSRP,FOXP4,SLC9A1,ECM1,BRPF3,PPARD,ZFP36L1,CCDC124,PCBP1,CDA,MDC1,WIZ,PPP1R13L,PCGF2,CRTC2,SRRM1,BCL9L,NCOR2,SAMD4B,DVL3,ZNF114,PLAGL2,UPF3B,NFIX,MED15,NFIC,NCOA6,ZMIZ2,CDKN1A,TP53INP2,MYD88,SKI,MAP2K7,CXXC1,ZNF521,GSK3A,SCAF4,SP2,PBX2,CAMTA2,UPF3A,CNOT3,CRTC1,CC2D1A,ATF5,SORBS3,CDK11B,RERE,GATAD2B,BCORL1,DMAP1,MEF2D,HMOX1,AFF3,EGR1,COL1A1,NGFR,NSRP1,TENM1,FOXQ1,ZNF580,GATA6,CREB3L1,DMD,PLK3,CCNK,GLI4,ZBED3,MLLT11,MRNIP,SPHK1,ZBTB12,PLEKHN1,MAGEC1,SOX12,ID4,ZNF703,MNT,ILK,BICRA,GLIS2,ZNF589,HSF4,KDM6B,PAX8,MAGEC2,TFAP2A,CACNG7,NR1D1,PDE2A,ZFP36,SIX2,SP140,ZNF296,TNFSF12,S1PR1,ARID3B,TRIM66,HMGN5,GATA3,GATA2,IKZF4,LOXL3,SUPT4H1,RFXAP,EDN1,APBB3,IGF2,CDK11A,MC1R,ZNF414,PRKCG,PAK6,PRRX1,ARL2BP,POU3F2,LIN37,FOSB,PARP15,MEIS1,TCF4,TRPV4,BMP6,KCNK2,MAMSTR,SETBP1,ANKLE1,HLX,GREM1,FOXA3,ZNF257,ELL3,CHD5,YY2,HOXD1,ZNF492,FOXD4,NELFCD,FOXD4L1,EGR4,UCHL1,SFRP1,FOXO6,DUSP26,HOXB8,TBX6,DLX4,ZNF716,RBM20,PLAGL1,ONECUT1,DRGX,SOX7
GO:0065007	biological regulation	0.0015217780957613462	FUS,CCN1,EIF5B,EIF1,MYADM,MT-CO2,RPS3A,FTH1,ANP32B,PABPC1,HNRNPH3,ARHGDIA,RIPOR1,FOSL1,UTP4,MT2A,PRKACA,MAT2A,FXYD5,SF3B4,CTDNEP1,S100A11,BRD4,CCN3,SF1,HNRNPD,FKBP1A,DIAPH1,TAF15,PRPF19,USP14,SMG7,PXN,ATN1,PTPN23,HNRNPA2B1,RAB11B,AGPAT1,TYMS,FST,RAB7A,CIZ1,CHERP,ADM,KHSRP,PSMD2,FOXP4,AKT1S1,EHD2,SLC29A1,CD44,ODC1,SLC9A1,PPP1R11,ECM1,BRPF3,PPARD,MIR137HG,ZFP36L1,DLGAP4,CCDC124,PCBP1,CDA,MDC1,WIZ,PPDPF,LOX,ZYX,MAPK13,BYSL,ESAM,PPP1R13L,PCGF2,C4BPB,CRTC2,DTNBP1,PSMC1,PDCL3,SRRM1,BCL9L,VAC14,SF3A2,NCOR2,SAMD4B,DVL3,ADAMTS1,ZNF114,NPRL3,PLAGL2,BAK1,UPF3B,NFIX,MED15,NFIC,NCOA6,PDE4A,VAMP2,ZMIZ2,PCIF1,CDKN1A,TP53INP2,EDC3,PRCC,MYD88,MCOLN1,SKI,GNG5,MAP2K7,CX

			XC1,RHOJ,PDE1C,ZNF521,GSK3A,SCAF4,SP2,PLEKHA7,PBX2,CAMTA2,DUSP7,CXCR4,UPF3A,UCP2,EIF2B4,CNOT3,RPS26,CRTC1,CC2D1A,ATF5,SORBS3,CDK11B,PHGDH,ZC3H3,NRGN,RERE,PHACTR4,GATAD2B,ALDOA,BCORL1,DGAT1,DMAP1,CCBE1,PABPC1L,BET1L,MEF2D,HMOX1,SETD1A,AFF3,EGR1,FAM98B,COL1A1,GABARAP,NGFR,SYVN1,NSRP1,TENM1,FOXQ1,GPR137,LSM10,DTNA,ZNF580,ESRP2,GATA6,SERPINB8,CREB3L1,SCAMP5,STXBP1,EIF3C,CAMK2N1,UCA1,DMD,NLGN2,PLK3,RELN,MMP1,CCNK,LTB4R,FURIN,PEAR1,EIF5AL1,CHRDL1,YJEFN3,DOK3,CTH,GPR3,RTKN,PRR16,GLI4,LIMS2,NAA80,DOCK6,PORCN,ZNF385A,ZBED3,RAB13,FAT3,MLLT11,DAPK1,MRNIP,SPHK1,OSGIN1,ZBTB12,PLEKHN1,MAGEC1,TAX1BP3,SOX12,SYNGAP1,ID4,ZNF703,RIN3,RGS4,MNT,EVI2B,ILK,BICRA,FZD8,GLIS2,SLC9A5,ZNF589,HSF4,TUBB3,NRK,GAREM1,KDM6B,GABRE,FABP5,ARHGEF19,LDOC1,PAX8,MAGEC2,TFAP2A,TNFSF15,GPRC5B,PSD4,CACNG7,ANKRD13B,NR1D1,ARC,PDE2A,ZFP36,CYP1A1,SYT11,SIX2,SP140,ZNF296,BMPER,TNFSF12,PDGFD,S1PR1,LRRC2,HSPB8,LTB4R2,ARID3B,TRIM66,FNDC4,ATP6V0C,HMGN5,GATA3,GATA2,IKZF4,LOXL3,SCNN1D,CNKSR2,SUPT4H1,KCNK1,DHPS,TRNP1,RFXAP,CTF1,EDN1,AMH,APBB3,IGF2,CDK11A,MMP2,SEMA6A,MC1R,CORO1A,ZNF414,SLITRK1,DDT,PRKCG,RAB3A,SERPINB2,GGT7,PAK6,ADGRE1,PRRT1,RNASEK,LCAT,PRRX1,MIR99AHG,ARL2BP,SORCS3,RAPGEFL1,ASIC1,GABRA3,SERPINE1,POU3F2,LIN37,FAM83A,FOSB,PARP15,RANGRF,GNG10,RHBDL1,WASHC1,MMP3,ACTA2,LRRC15,PTPN22,COL1A2,CYRIA,CST4,MEIS1,S1PR3,TCF4,PRRT2,TRPV4,BMP6,CYP2J2,CLDN1,BCL2L2-PABPN1,PHACTR1,ASB16,TGFBR3L,GPR15,DPYSL3,KCNK2,OR2B6,GPR132,MAMSTR,GPR35,SETBP1,GNG13,TRBV12-4,ANKLE1,HLX,GNMT,PKDCC,LAMA1,PTHLH,DBH,TMEM198,CD177,GREM1,FOXA3,LYPD3,AMTN,ZNF257,NRXN3,ANO7,BCHE,TAS2R5,SPDYE12,KCNIP2,ATP1A3,TNFRSF25,ELL3,LILRA6,CHD5,TPH1,NUSAP1,KCNC3,YY2,HOXD1,CRLF1,GJB3,RCAN2,SRCIN1,ZNF492,FOXD4,VSTM1,COL3A1,SAMSN1,NELFCD,FOXD4L1,EGR4,WNK4,GPR146,ARHGEF16,UCHL1,ESM1,IL1RN,SFRP1,KCNAB3,ATP6V1G2,GNRH2,SERPINB7,EBI3,ARHGAP20,PANX2,SPNS1,FLRT1,PCSK1N,CDK5R2,TIE1,FOXO6,PLCXD3,GRIN2A,HSD11B2,HLA-DOB,HCN2,CGB7,PCDHB2,SERPINB5,SULT1B1,DUSP26,KCNE5,ADH6,HOXB8,PCDHB6,HUS1B,TBX6,MAOB,EPHA6,GRIN3B,DLX4,ADGRB3,ZNF716,TMEM119,TMLHE,RAPSN,KIAA0319,AMPH,TMED7-TICAM2,SLC7A8,RBM20,KCND1,ADAMTS5,PLAGL1,GPR87,SPINK1,MIF,ENHO,ALOXE3,LAG3,FNDC5,GKN2,TNFRSF13C,GPRC5D,THY1,ONECUT1,NPFF,DRGX,CGB8,SOX7
GO:0070482	response to oxygen levels	0.001691884460176544	*MT-CO2,MT-CYB,MT-ND2,MT-ND5,MT-CO1,MT-ND4,MT-ND1,MT-ATP6,ADM,POLR2A,SLC29A1,SLC9A1,PPARD,ZFP36L1,CDKN1A,CXCR4,UCP2,EGR1,COL1A1,GATA6,PLEKHN1,CYP1A1,EDN1,MMP2,TRPV4,KCNK2,SFRP1,UCP3,HSD11B2*
GO:0051252	regulation of RNA metabolic process	0.001789461064579821	*FUS,CCN1,EIF1,PABPC1,FOSL1,UTP4,SF3B4,BRD4,SF1,HNRNPD,TAF15,PRPF19,ATN1,HNRNPA2B1,FST,KHSRP,FOXP4,SLC9A1,ECM1,BRPF3,PPARD,ZFP36L1,CCDC124,PCBP1,MDC1,WIZ,PPP1R13L,PCGF2,CRTC2,SRRM1,BCL9L,NCOR2,SAMD4B,DVL3,ZNF114*

			,PLAGL2,UPF3B,NFIX,MED15,NFIC,NCOA6,ZMIZ2,TP53INP2,MYD88,SKI,MAP2K7,CXXC1,ZNF521,GSK3A,SCAF4,SP2,PBX2,CAMTA2,UPF3A,CNOT3,CRTC1,CC2D1A,ATF5,SORBS3,CDK11B,RERE,GATAD2B,BCORL1,DMAP1,MEF2D,HMOX1,AFF3,EGR1,COL1A1,NGFR,NSRP1,TENM1,FOXQ1,ZNF580,GATA6,CREB3L1,DMD,PLK3,CCNK,GLI4,ZBED3,MLLT11,SPHK1,ZBTB12,PLEKHN1,MAGEC1,SOX12,ID4,ZNF703,MNT,ILK,BICRA,GLIS2,ZNF589,HSF4,KDM6B,PAX8,MAGEC2,TFAP2A,CACNG7,NR1D1,PDE2A,ZFP36,SIX2,SP140,ZNF296,S1PR1,ARID3B,TRIM66,HMGN5,GATA3,GATA2,IKZF4,LOXL3,SUPT4H1,RFXAP,EDN1,APBB3,IGF2,CDK11A,MC1R,ZNF414,PAK6,PRRX1,ARL2BP,POU3F2,LIN37,FOSB,PARP15,MEIS1,TCF4,TRPV4,BMP6,MAMSTR,SETBP1,HLX,GREM1,FOXA3,ZNF257,ELL3,CHD5,YY2,HOXD1,ZNF492,FOXD4,NELFCD,FOXD4L1,EGR4,SFRP1,FOXO6,DUSP26,HOXB8,TBX6,DLX4,ZNF716,RBM20,PLAGL1,ONECUT1,DRGX,SOX7
GO:0009719	response to endogenous stimulus	0.001822564338670748 4	CCN1,MT-CYB,MT-ND3,FOSL1,PRKACA,FKBP1A,PXN,TYMS,FST,ADM,AKT1S1,NUDC,CD44,SLC9A1,PPARD,ZFP36L1,LOX,ZYX,BCL9L,NCOR2,NCOA6,VAMP2,CDKN1A,MYD88,SKI,GSK3A,UCP2,EIF2B4,CCBE1,EGR1,COL1A1,NGFR,GATA6,CREB3L1,DMD,FURIN,CHRDL1,ZBED3,RAB13,SPHK1,ZNF703,ILK,GAREM1,PAX8,NR1D1,PDE2A,ZFP36,BMPER,PDGFD,ARSA,FNDC4,GATA3,SCNN1D,EDN1,IGF2,MMP2,SEMA6A,CORO1A,FOSB,ACTA2,COL1A2,TRPV4,BMP6,CLDN1,TGFBR3L,GREM1,BCHE,ATP1A3,COL3A1,IL1RN,SFRP1,FLRT1,UCP3,HSD11B2,MAOB,MXRA5,SPINK1,ONECUT1,CGB8
GO:0048514	blood vessel morphogenesis	0.001898983614617411 6	CCN1,CCN3,ADM,ECM1,ZFP36L1,LOX,PDCL3,ADAMTS1,NPRL3,BAK1,RHOJ,CXCR4,CCBE1,HMOX1,NGFR,GATA6,CREB3L1,YJEFN3,SPHK1,FZD8,BMPER,TNFSF12,S1PR1,GATA2,EDN1,MMP2,SEMA6A,PRRX1,SERPINE1,MEIS1,ANPEP,GPR15,LAMA1,GREM1,ANGPTL6,NRXN3,COL3A1,ESM1,SFRP1,TIE1,TBX6,ADGRB3,THY1
GO:0010557	positive regulation of macromolecule biosynthetic process	0.002920300936633980 3	FUS,CCN1,EIF1,PABPC1,FOSL1,SF3B4,BRD4,HNRNPD,TAF15,PRPF19,ATN1,AGPAT1,SLC9A1,PPARD,CCDC124,PCBP1,MDC1,MAPK13,CRTC2,DTNBP1,PDCL3,BCL9L,DVL3,PLAGL2,UPF3B,NFIX,MED15,NFIC,NCOA6,ZMIZ2,PCIF1,TP53INP2,MYD88,SKI,MAP2K7,CXXC1,ZNF521,GSK3A,PBX2,CAMTA2,UPF3A,CRTC1,ATF5,RERE,GATAD2B,DMAP1,CCBE1,PABPC1L,MEF2D,HMOX1,EGR1,FAM98B,COL1A1,NGFR,ZNF580,GATA6,CREB3L1,SCAMP5,EIF3C,CCNK,EIF5AL1,PRR16,ZBED3,MLLT11,SPHK1,SOX12,ID4,ILK,BICRA,GLIS2,KDM6B,PAX8,TFAP2A,GPRC5B,NR1D1,PDE2A,ZFP36,SIX2,TNFSF12,S1PR1,ARID3B,HMGN5,GATA3,GATA2,IKZF4,SUPT4H1,RFXAP,EDN1,AMH,IGF2,MC1R,DDT,PRRX1,ARL2BP,SERPINE1,POU3F2,FOSB,ACTA2,PTPN22,MEIS1,TCF4,TRPV4,BMP6,MAMSTR,GREM1,FOXA3,ELL3,YY2,EGR4,SFRP1,SERPINB7,EBI3,PANX2,TBX6,TMEM119,RBM20,PLAGL1,MIF,ONECUT1,SOX7
GO:0042542	response to hydrogen peroxide	0.003144913942992172 2	MT-ND5,MT-ND6,FOSL1,MAPK13,PCGF2,BAK1,HMOX1,COL1A1,ZNF580,SPHK1,KDM6B,PDGFD,EDN1,MMP2
GO:0098662	inorganic cation transmembrane	0.003455202701231953 2	MT-CO2,MT-CYB,MT-ND2,MT-ND5,MT-CO1,MT-ND3,MT-ND4,MT-ND1,MT-ATP6,MT-ND4L,MT-ND6,MT-ATP8,PRKACA,MT-CO3,FKBP1A,DIAPH1,CHERP,SLC9A1,BAK1,VAMP2,

	transport		*MCOLN1,UCP2,DMD,SLC39A4,RGS4,SLC9A5,CACNG7,ATP6V0C,SCNN1D,KCNK1,EDN1,CORO1A,RNASEK,ASIC1,RANGRF,PTPN22,TRPV4,KCNK2,GPR35,KCNIP2,ATP1A3,KCNC3,WNK4,KCNAB3,ATP6V1G2,UCP3,GRIN2A,HCN2,KCNE5,GRIN3B,KCND1,THY1*
GO:0007399	nervous system development	0.003610158591463277 7	*MT-CO1,MT-ND4,ANP32B,ATIC,PRKACA,HNRNPD,ATN1,CHERP,ADM,PPARD,ZFP36L1,DLGAP4,DTNBP1,MAL2,SF3A2,NCOR2,DVL3,UPF3B,NCOA6,MYD88,SKI,ZNF521,GSK3A,PBX2,CXCR4,EIF2B4,TPP1,CC2D1A,ATF5,PHGDH,NRGN,RERE,PHACTR4,MEF2D,SETD1A,NGFR,TENM1,STXBP1,DMD,NLGN2,RELN,ENO3,CHRDL1,RAB13,FAT3,SPHK1,SOX12,SYNGAP1,ID4,RGS4,ILK,FZD8,GLIS2,TUBB3,KDM6B,GABRE,PAX8,TFAP2A,GPRC5B,CACNG7,NR1D1,ARC,ZNF296,S1PR1,GATA3,GATA2,LOXL3,TRNP1,CTF1,EDN1,MMP2,SEMA6A,SLITRK1,PRKCG,RAB3A,PAK6,PRRX1,RAPGEFL1,ASIC1,GABRA3,POU3F2,MEIS1,TCF4,TRPV4,BMP6,CLDN1,KIRREL3,PHACTR1,SYPL2,DPYSL3,INKA1,HLX,LAMA1,NRXN3,BCHE,KCNIP2,ELL3,CHD5,HOXD1,NELL2,SRCIN1,COL3A1,UCHL1,SFRP1,FLRT1,CDK5R2,AATK,FOXO6,GRIN2A,PCDHB2,HOXB8,PCDHB6,TBX6,MAOB,EPHA6,ADGRB3,KIAA0319,THY1,ONECUT1,DRGX*
GO:0048519	negative regulation of biological process	0.003644732277726901 7	*FUS,CCN1,MYADM,RPS3A,FTH1,ANP32B,PABPC1,ARHGDIA,RIPOR1,FOSL1,MT2A,PRKACA,FXYD5,S100A11,BRD4,CCN3,SF1,HNRNPD,FKBP1A,DIAPH1,TAF15,PRPF19,USP14,SMG7,ATN1,PTPN23,HNRNPA2B1,TYMS,FST,RAB7A,CIZ1,CHERP,ADM,KHSRP,FOXP4,AKT1S1,CD44,SLC9A1,PPP1R11,ECM1,PPARD,MIR137HG,ZFP36L1,CDA,MDC1,LOX,PPP1R13L,PCGF2,C4BPB,DTNBP1,PDCL3,BCL9L,NCOR2,SAMD4B,ADAMTS1,NPRL3,BAK1,UPF3B,NFIX,NFIC,PCIF1,CDKN1A,TP53INP2,EDC3,MYD88,MCOLN1,SKI,GSK3A,SCAF4,SP2,DUSP7,UPF3A,UCP2,CNOT3,RPS26,CRTC1,CC2D1A,ATF5,SORBS3,RERE,PHACTR4,GATAD2B,BCORL1,DMAP1,HMOX1,EGR1,COL1A1,NGFR,SYVN1,TENM1,FOXQ1,GPR137,GATA6,SERPINB8,CREB3L1,SCAMP5,STXBP1,DMD,PLK3,FURIN,CHRDL1,YJEFN3,CTH,LIMS2,ZNF385A,ZBED3,FAT3,DAPK1,MRNIP,SPHK1,OSGIN1,ZBTB12,PLEKHN1,MAGEC1,TAX1BP3,SOX12,SYNGAP1,ID4,ZNF703,RIN3,RGS4,MNT,EVI2B,ILK,BICRA,GLIS2,ZNF589,HSF4,GABRE,FABP5,LDOC1,PAX8,MAGEC2,TFAP2A,CACNG7,ANKRD13B,NR1D1,ARC,PDE2A,ZFP36,SYT11,SIX2,ZNF296,BMPER,S1PR1,TRIM66,FNDC4,HMGN5,GATA3,GATA2,IKZF4,LOXL3,SUPT4H1,EDN1,AMH,IGF2,MMP2,SEMA6A,MC1R,CORO1A,DDT,PRKCG,SERPINB2,GGT7,PRRT1,PRRX1,MIR99AHG,SORCS3,ASIC1,SERPINE1,POU3F2,LIN37,FOSB,PARP15,WASHC1,MMP3,LRRC15,PTPN22,CST4,MEIS1,S1PR3,PRRT2,TRPV4,BMP6,BCL2L2-PABPN1,DPYSL3,KCNK2,GPR132,GPR35,ANKLE1,HLX,PKDCC,PTHLH,GREM1,LYPD3,BCHE,ELL3,CHD5,TPH1,CRLF1,SRCIN1,COL3A1,SAMSN1,NELFCD,WNK4,UCHL1,IL1RN,SFRP1,PCSK1N,TIE1,GRIN2A,HLA-DOB,DUSP26,KCNE5,HOXB8,HUS1B,TBX6,MAOB,DLX4,ADGRB3,TMEM119,KIAA0319,RBM20,ADAMTS5,PLAGL1,SPINK1,MIF,LAG3,THY1,ONECUT1,SOX7*
GO:0035239	tube morphogenesis	0.003660687130167146 3	*CCN1,PRKACA,CCN3,ADM,ECM1,ZFP36L1,LOX,PDCL3,ADAMTS1,NPRL3,BAK1,SKI,RHOJ,CXCR4,PHACTR4,CCBE1,HMOX1,NGFR,ESRP2,GATA6,CREB3L1,YJEFN3,SPHK1,ILK,FZD8,PAX8,SIX2,BMPER,TNFSF12,S1PR1,GATA3,GATA2,EDN1,MMP2,SEMA6A,PRRX1,SERPINE1,MEIS1,ANPEP,GPR15,HLX,LAMA1,GREM1*

			,ANGPTL6,NRXN3,COL3A1,WNK4,ESM1,SFRP1,TIE1,TBX6,ADGRB3,THY1
GO:0009410	response to xenobiotic stimulus	0.003926662664946646	MT-CYB,MT-ND1,FOSL1,TYMS,SLC29A1,LOX,DVL3,BAK1,PDE4A,CDKN1A,CXCR4,COL1A1,GATA6,DMD,GSTM1,SLC29A3,PDE2A,CYP1A1,RPP21,EDN1,AMH,MMP2,FOSB,AOC2,CYP2J2,BCHE,WNK4,UCHL1,SFRP1,GRIN2A,HSD11B2,SULT1B1,MAOB
GO:0009891	positive regulation of biosynthetic process	0.004313124413922697	FUS,CCN1,EIF1,PABPC1,FOSL1,PRKACA,SF3B4,CTDNEP1,BRD4,HNRNPD,TAF15,PRPF19,ATN1,AGPAT1,ADM,SLC9A1,PPARD,CCDC124,PCBP1,MDC1,MAPK13,CRTC2,DTNBP1,PDCL3,BCL9L,DVL3,PLAGL2,UPF3B,NFIX,MED15,NFIC,NCOA6,ZMIZ2,PCIF1,TP53INP2,MYD88,SKI,MAP2K7,CXXC1,ZNF521,GSK3A,PBX2,CAMTA2,UPF3A,CRTC1,ATF5,RERE,GATAD2B,DMAP1,CCBE1,PABPC1L,MEF2D,HMOX1,EGR1,FAM98B,COL1A1,NGFR,ZNF580,GATA6,CREB3L1,SCAMP5,EIF3C,CCNK,EIF5AL1,PRR16,ZBED3,MLLT11,SPHK1,SOX12,ID4,ILK,BICRA,GLIS2,KDM6B,PAX8,TFAP2A,GPRC5B,NR1D1,PDE2A,ZFP36,SIX2,TNFSF12,S1PR1,ARID3B,HMGN5,GATA3,GATA2,IKZF4,SUPT4H1,RFXAP,EDN1,AMH,IGF2,MC1R,DDT,PRRX1,ARL2BP,SERPINE1,POU3F2,FOSB,ACTA2,PTPN22,MEIS1,TCF4,TRPV4,BMP6,MAMSTR,GREM1,FOXA3,ELL3,YY2,EGR4,SFRP1,SERPINB7,EBI3,PANX2,TBX6,TMEM119,RBM20,PLAGL1,MIF,ONECUT1,SOX7
GO:0035295	tube development	0.00460419057143194	CCN1,PRKACA,CCN3,TYMS,ADM,ECM1,ZFP36L1,LOX,PDCL3,ADAMTS1,NPRL3,BAK1,CDKN1A,SKI,RHOJ,CXCR4,PHGDH,PHACTR4,CCBE1,HMOX1,NGFR,ESRP2,GATA6,CREB3L1,YJEFN3,SPHK1,ILK,FZD8,PAX8,CYP1A1,SIX2,BMPER,TNFSF12,S1PR1,GATA3,GATA2,LOXL3,EDN1,MMP2,SEMA6A,RAB3A,PRRX1,SERPINE1,MEIS1,ANPEP,GPR15,INKA1,HLX,PKDCC,LAMA1,GREM1,ANGPTL6,NRXN3,CRLF1,COL3A1,WNK4,ESM1,SFRP1,TIE1,TBX6,ADGRB3,THY1
GO:0042127	regulation of cell population proliferation	0.005201025651578444	CCN1,FTH1,FOSL1,S100A11,CCN3,SF1,CHERP,ADM,ODC1,ECM1,PPARD,ZFP36L1,PDCL3,ADAMTS1,BAK1,CDKN1A,MYD88,SKI,GNG5,ATF5,MEF2D,HMOX1,EGR1,FAM98B,NGFR,TENM1,ZNF580,ESRP2,GATA6,NLGN2,LIMS2,SPHK1,TAX1BP3,ID4,ZNF703,ILK,BICRA,GAREM1,LDOC1,TFAP2A,NR1D1,ZFP36,SIX2,TNFSF12,PDGFD,S1PR1,HMGN5,GATA3,GATA2,DHPS,TRNP1,CTF1,EDN1,IGF2,MMP2,CORO1A,PRRX1,POU3F2,PTPN22,MEIS1,S1PR3,BMP6,CLDN1,KCNK2,HLX,PTHLH,DBH,GREM1,BCHE,ELL3,CHD5,CRLF1,EGR4,ESM1,SFRP1,SERPINB7,EBI3,TIE1,SERPINB5,TMEM119,SPINK1,MIF,GKN2,TNFRSF13C,SOX7
GO:0050789	regulation of biological process	0.005640075381545925 5	FUS,CCN1,EIF5B,EIF1,MYADM,RPS3A,FTH1,ANP32B,PABPC1,HNRNPH3,ARHGDIA,RIPOR1,FOSL1,UTP4,MT2A,PRKACA,MAT2A,FXYD5,SF3B4,CTDNEP1,S100A11,BRD4,CCN3,SF1,HNRNPD,FKBP1A,DIAPH1,TAF15,PRPF19,USP14,SMG7,PXN,ATN1,PTPN23,HNRNPA2B1,RAB11B,AGPAT1,TYMS,FST,RAB7A,CIZ1,CHERP,ADM,KHSRP,PSMD2,FOXP4,AKT1S1,EHD2,SLC29A1,CD44,ODC1,SLC9A1,PPP1R11,ECM1,BRPF3,PPARD,MIR137HG,ZFP36L1,DLGAP4,CCDC124,PCBP1,CDA,MDC1,WIZ,PPDPF,LOX,ZYX,MAPK13,BYSL,ESAM,PPP1R13L,PCGF2,C4BPB,CRTC2,DTNBP1,PSMC1,PDCL3,SRRM1,BCL9L,VAC14,SF3A2,NCOR2,SAMD4B,DVL3,ADAMTS1,ZNF114,NPRL3,PLAGL2,BAK1,UPF3B,NFIX,MED15,NFIC,NCOA6,PDE4A,VAMP2,ZMIZ2,PCIF1,CDKN1A,TP53INP2,EDC3,PRCC,MYD88,MCOLN1,SKI,GNG5,MAP2K7,CXXC1,RHOJ,PDE1C,ZNF521,GSK3A,SCAF4,SP2,PBX2,CAMTA2,DUSP7,CXCR4,

			UPF3A,UCP2,EIF2B4,CNOT3,RPS26,CRTC1,CC2D1A,ATF5,SORBS3,CDK11B,PHGDH,ZC3H3,NRGN,RERE,PHACTR4,GATAD2B,ALDOA,BCORL1,DGAT1,DMAP1,CCBE1,PABPC1L,BET1L,MEF2D,HMOX1,SETD1A,AFF3,EGR1,FAM98B,COL1A1,GABARAP,NGFR,SYVN1,NSRP1,TENM1,FOXQ1,GPR137,LSM10,DTNA,ZNF580,ESRP2,GATA6,SERPINB8,CREB3L1,SCAMP5,STXBP1,EIF3C,CAMK2N1,UCA1,DMD,NLGN2,PLK3,RELN,MMP1,CCNK,LTB4R,FURIN,PEAR1,EIF5AL1,CHRDL1,YJEFN3,DOK3,CTH,GPR3,RTKN,PRR16,GLI4,LIMS2,NAA80,DOCK6,PORCN,ZNF385A,ZBED3,RAB13,FAT3,MLLT11,DAPK1,MRNIP,SPHK1,OSGIN1,ZBTB12,PLEKHN1,MAGEC1,TAX1BP3,SOX12,SYNGAP1,ID4,ZNF703,RIN3,RGS4,MNT,EVI2B,ILK,BICRA,FZD8,GLIS2,ZNF589,HSF4,TUBB3,NRK,GAREM1,KDM6B,GABRE,FABP5,ARHGEF19,LDOC1,PAX8,MAGEC2,TFAP2A,TNFSF15,GPRC5B,PSD4,CACNG7,ANKRD13B,NR1D1,ARC,PDE2A,ZFP36,CYP1A1,SYT11,SIX2,SP140,ZNF296,BMPER,TNFSF12,PDGFD,S1PR1,LRRC2,HSPB8,LTB4R2,ARID3B,TRIM66,FNDC4,ATP6V0C,HMGN5,GATA3,GATA2,IKZF4,LOXL3,CNKSR2,SUPT4H1,DHPS,TRNP1,RFXAP,CTF1,EDN1,AMH,APBB3,IGF2,CDK11A,MMP2,SEMA6A,MC1R,CORO1A,ZNF414,SLITRK1,DDT,PRKCG,RAB3A,SERPINB2,GGT7,PAK6,ADGRE1,PRRT1,LCAT,PRRX1,MIR99AHG,ARL2BP,SORCS3,RAPGEFL1,ASIC1,GABRA3,SERPINE1,POU3F2,LIN37,FAM83A,FOSB,PARP15,RANGRF,GNG10,RHBDL1,WASHC1,MMP3,ACTA2,LRRC15,PTPN22,COL1A2,CYRIA,CST4,MEIS1,S1PR3,TCF4,PRRT2,TRPV4,BMP6,CYP2J2,CLDN1,BCL2L2-PABPN1,PHACTR1,ASB16,TGFBR3L,GPR15,DPYSL3,KCNK2,OR2B6,GPR132,MAMSTR,GPR35,SETBP1,GNG13,TRBV12-4,ANKLE1,HLX,GNMT,PKDCC,LAMA1,PTHLH,DBH,TMEM198,CD177,GREM1,FOXA3,LYPD3,AMTN,ZNF257,NRXN3,BCHE,TAS2R5,SPDYE12,KCNIP2,ATP1A3,TNFRSF25,ELL3,LILRA6,CHD5,TPH1,NUSAP1,YY2,HOXD1,CRLF1,GJB3,RCAN2,SRCIN1,ZNF492,FOXD4,VSTM1,COL3A1,SAMSN1,NELFCD,FOXD4L1,EGR4,WNK4,GPR146,ARHGEF16,UCHL1,ESM1,IL1RN,SFRP1,KCNAB3,ATP6V1G2,GNRH2,SERPINB7,EBI3,ARHGAP20,PANX2,FLRT1,PCSK1N,CDK5R2,TIE1,FOXO6,PLCXD3,GRIN2A,HLA-DOB,HCN2,CGB7,PCDHB2,SERPINB5,DUSP26,KCNE5,HOXB8,PCDHB6,HUS1B,TBX6,MAOB,EPHA6,GRIN3B,DLX4,ADGRB3,ZNF716,TMEM119,RAPSN,KIAA0319,AMPH,TMED7-TICAM2,RBM20,ADAMTS5,PLAGL1,GPR87,SPINK1,MIF,ENHO,ALOXE3,LAG3,FNDC5,GKN2,TNFRSF13C,GPRC5D,THY1,ONECUT1,NPFF,DRGX,CGB8,SOX7
GO:0006366	transcription by RNA polymerase II	0.005698197998917074	FUS,CCN1,FOSL1,BRD4,HNRNPD,ATN1,HNRNPA2B1,FST,POLR2A,KHSRP,FOXP4,SLC9A1,ECM1,BRPF3,PPARD,CCDC124,PCBP1,MDC1,WIZ,PPP1R13L,PCGF2,CRTC2,BCL9L,NCOR2,DVL3,ZNF114,PLAGL2,NFIX,MED15,NFIC,NCOA6,ZMIZ2,MYD88,SKI,ZNF521,GSK3A,SCAF4,SP2,PBX2,CAMTA2,CRTC1,CC2D1A,TAF1C,ATF5,SORBS3,GATAD2B,BCORL1,DMAP1,PABPC1L,MEF2D,HMOX1,EGR1,FOXQ1,ZNF580,GATA6,CREB3L1,PLK3,CCNK,GLI4,ZBED3,ZBTB12,MAGEC1,SOX12,ID4,MNT,BICRA,GLIS2,ZNF589,HSF4,KDM6B,PAX8,MAGEC2,TFAP2A,NR1D1,PDE2A,ZFP36,SIX2,SP140,ZNF296,S1PR1,ARID3B,GATA3,GATA2,IKZF4,SUPT4H1,RFXAP,EDN1,IGF2,MC1R,PRRX1,POU3F2,LIN37,FOSB,PARP15,MEIS1,TCF4,TRPV4,BMP6,

			MAMSTR,HLX,GREM1,FOXA3,ZNF257,ELL3,CHD5,YY2,HOXD1,ZNF492,FOXD4,NELFCD,FOXD4L1,EGR4,FOXO6,GTF2H4,DUSP26,HOXB8,TBX6,DLX4,ZNF716,PLAGL1,ONECUT1,DRGX,SOX7
GO:0098660	inorganic ion transmembrane transport	0.006531097010618273	*MT-CO2,MT-CYB,MT-ND2,MT-ND5,MT-CO1,MT-ND3,MT-ND4,MT-ND1,MT-ATP6,MT-ND4L,MT-ND6,MT-ATP8,PRKACA,MT-CO3,FKBP1A,DIAPH1,CHERP,SLC9A1,BAK1,VAMP2,MCOLN1,UCP2,DMD,SLC39A4,RGS4,SLC9A5,GABRE,CACNG7,ATP6V0C,SCNN1D,KCNK1,EDN1,CORO1A,RNASEK,ASIC1,GABRA3,RANGRF,PTPN22,TRPV4,KCNK2,GPR35,ANO7,KCNIP2,ATP1A3,KCNC3,WNK4,KCNAB3,ATP6V1G2,UCP3,GRIN2A,HCN2,KCNE5,GRIN3B,KCND1,THY1*
GO:0098655	monoatomic cation transmembrane transport	0.007442488508552506	*MT-CO2,MT-CYB,MT-ND2,MT-ND5,MT-CO1,MT-ND3,MT-ND4,MT-ND1,MT-ATP6,MT-ND4L,MT-ND6,MT-ATP8,PRKACA,MT-CO3,FKBP1A,DIAPH1,CHERP,SLC9A1,BAK1,VAMP2,MCOLN1,UCP2,DMD,SLC39A4,RGS4,SLC9A5,CACNG7,ATP6V0C,SCNN1D,KCNK1,EDN1,CORO1A,RNASEK,ASIC1,RANGRF,PTPN22,TRPV4,KCNK2,GPR35,KCNIP2,ATP1A3,KCNC3,WNK4,KCNAB3,ATP6V1G2,UCP3,GRIN2A,HCN2,KCNE5,GRIN3B,KCND1,THY1*
GO:0006351	DNA-templated transcription	0.008003273600982077	*FUS,CCN1,FOSL1,UTP4,BRD4,SF1,HNRNPD,TAF15,ATN1,HNRNPA2B1,FST,POLR2A,KHSRP,FOXP4,SLC9A1,ECM1,BRPF3,PPARD,CCDC124,PCBP1,MDC1,WIZ,PPP1R13L,PCGF2,CRTC2,BCL9L,NCOR2,DVL3,ZNF114,PLAGL2,NFIX,MED15,NFIC,NCOA6,XAB2,ZMIZ2,TP53INP2,MYD88,SKI,MAP2K7,CXXC1,ZNF521,GSK3A,SCAF4,SP2,PBX2,CAMTA2,CNOT3,CRTC1,CC2D1A,TAF1C,ATF5,SORBS3,CDK11B,RERE,GATAD2B,BCORL1,DMAP1,PABPC1L,MEF2D,HMOX1,AFF3,EGR1,COL1A1,NGFR,TENM1,FOXQ1,ZNF580,GATA6,CREB3L1,DMD,PLK3,CCNK,GLI4,ZBED3,MLLT11,SPHK1,ZBTB12,MAGEC1,SOX12,ID4,ZNF703,MNT,ILK,BICRA,GLIS2,ZNF589,HSF4,KDM6B,PAX8,MAGEC2,TFAP2A,NR1D1,PDE2A,ZFP36,SIX2,SP140,ZNF296,S1PR1,ARID3B,TRIM66,HMGN5,GATA3,GATA2,IKZF4,LOXL3,SUPT4H1,RFXAP,EDN1,APBB3,IGF2,CDK11A,MC1R,ZNF414,PAK6,PRRX1,ARL2BP,POU3F2,LIN37,FOSB,PARP15,MEIS1,TCF4,TRPV4,BMP6,MAMSTR,SETBP1,HLX,GREM1,FOXA3,ZNF257,ELL3,CHD5,YY2,HOXD1,ZNF492,FOXD4,NELFCD,FOXD4L1,EGR4,SFRP1,FOXO6,GTF2H4,DUSP26,HOXB8,TBX6,DLX4,ZNF716,PLAGL1,ONECUT1,DRGX,SOX7*
GO:0015988	energy coupled proton transmembrane transport, against electrochemical gradient	0.00830037326805478	*MT-CYB,MT-ND5,MT-CO1,MT-ND4*
GO:0015990	electron transport coupled proton transport	0.00830037326805478	*MT-CYB,MT-ND5,MT-CO1,MT-ND4*
GO:0006355	regulation of DNA-templated	0.008422064570238986	*FUS,CCN1,FOSL1,UTP4,BRD4,SF1,HNRNPD,TAF15,ATN1,HNRNPA2B1,FST,KHSRP,FOXP4,SLC9A1,ECM1,BRPF3,PPARD,CCDC124,PCBP1,MDC1,WIZ,PPP1R1*

	transcription		*3L,PCGF2,CRTC2,BCL9L,NCOR2,DVL3,ZNF114,PLAGL2,NFIX,MED15,NFIC,NCOA6,ZMIZ2,TP53INP2,MYD88,SKI,MAP2K7,CXXC1,ZNF521,GSK3A,SCAF4,SP2,PBX2,CAMTA2,CNOT3,CRTC1,CC2D1A,ATF5,SORBS3,CDK11B,RERE,GATAD2B,BCORL1,DMAP1,MEF2D,HMOX1,AFF3,EGR1,COL1A1,NGFR,TENM1,FOXQ1,ZNF580,GATA6,CREB3L1,DMD,PLK3,CCNK,GLI4,ZBED3,MLLT11,SPHK1,ZBTB12,MAGEC1,SOX12,ID4,ZNF703,MNT,ILK,BICRA,GLIS2,ZNF589,HSF4,KDM6B,PAX8,MAGEC2,TFAP2A,NR1D1,PDE2A,ZFP36,SIX2,SP140,ZNF296,S1PR1,ARID3B,TRIM66,HMGN5,GATA3,GATA2,IKZF4,LOXL3,SUPT4H1,RFXAP,EDN1,APBB3,IGF2,CDK11A,MC1R,ZNF414,PAK6,PRRX1,ARL2BP,POU3F2,LIN37,FOSB,PARP15,MEIS1,TCF4,TRPV4,BMP6,MAMSTR,SETBP1,HLX,GREM1,FOXA3,ZNF257,ELL3,CHD5,YY2,HOXD1,ZNF492,FOXD4,NELFCD,FOXD4L1,EGR4,SFRP1,FOXO6,DUSP26,HOXB8,TBX6,DLX4,ZNF716,PLAGL1,ONECUT1,DRGX,SOX7*
GO:0045934	negative regulation of nucleobase-containing compound metabolic process	0.009383662538845032	*FUS,PABPC1,PRKACA,S100A11,SF1,HNRNPD,TAF15,ATN1,HNRNPA2B1,FST,FOXP4,PPARD,CDA,PPP1R13L,PCGF2,NCOR2,NFIX,NFIC,CDKN1A,MYD88,SKI,GSK3A,SCAF4,SP2,UPF3A,CC2D1A,ATF5,SORBS3,RERE,GATAD2B,BCORL1,DMAP1,EGR1,FOXQ1,GATA6,CREB3L1,PLK3,ZBTB12,MAGEC1,SOX12,ID4,ZNF703,MNT,GLIS2,ZNF589,HSF4,MAGEC2,TFAP2A,NR1D1,PDE2A,ZFP36,ZNF296,TRIM66,GATA3,GATA2,IKZF4,LOXL3,SUPT4H1,EDN1,IGF2,PRRX1,LIN37,FOSB,PARP15,TRPV4,BMP6,KCNK2,ANKLE1,GREM1,NELFCD,SFRP1,DUSP26,HOXB8,TBX6,DLX4,RBM20,PLAGL1,SOX7*
GO:0048869	cellular developmental process	0.010098099021415539	*CCN1,MYADM,RPS3A,ANP32B,HNRNPH3,PRKACA,BRD4,CCN3,SF1,HNRNPD,TYMS,FST,ADM,EHD2,SLC9A1,ECM1,BRPF3,PPARD,ZFP36L1,LAMB3,PPDPF,LOX,BYSL,PDLIM7,PPP1R13L,DTNBP1,BCL9L,SF3A2,DVL3,BAK1,UPF3B,NCOA6,CDKN1A,TP53INP2,MYD88,SKI,ZNF521,GSK3A,PBX2,CXCR4,UCP2,EIF2B4,CNOT3,TPP1,CC2D1A,ATF5,PHGDH,RERE,PHACTR4,GATAD2B,PABPC1L,MEF2D,SETD1A,EGR1,COL1A1,NGFR,SYVN1,TENM1,FOXQ1,GPR137,GATA6,CREB3L1,STXBP1,DMD,NLGN2,RELN,CHRDL1,YJEFN3,CTH,NHSL3,ZNF385A,RAB13,FAT3,OSGIN1,SOX12,SYNGAP1,ID4,ZNF703,RGS4,EVI2B,ILK,BICRA,FZD8,GLIS2,HSF4,TUBB3,KDM6B,PAX8,TFAP2A,GPRC5B,CACNG7,NR1D1,ARC,PDE2A,ZFP36,CYP1A1,SIX2,ZNF296,TNFSF12,S1PR1,GATA3,GATA2,LOXL3,MYO7B,CTF1,EDN1,AMH,IGF2,MMP2,SEMA6A,SLITRK1,RAB3A,CCDC78,PAK6,PRRX1,SERPINE1,POU3F2,ACTA2,PTPN22,MEIS1,S1PR3,TCF4,TRPV4,BMP6,CLDN1,KIRREL3,PHACTR1,SYPL2,ANPEP,TGFBR3L,DPYSL3,SNX10,MAMSTR,ANKLE1,HLX,PKDCC,LAMA1,PTHLH,GREM1,FOXA3,ANGPTL6,NRXN3,BCHE,KCNIP2,ELL3,CHD5,TPH1,HOXD1,NELL2,SRCIN1,FOXD4,COL3A1,STIMATE-MUSTN1,FOXD4L1,UCHL1,SFRP1,FLRT1,CDK5R2,TIE1,KRT14,FOXO6,GRIN2A,CNFN,SULT1B1,HOXB8,TBX6,EPHA6,DLX4,ADGRB3,TMEM190,TMEM119,KIAA0319,TCHH,ADAMTS5,SPINK1,ALOXE3,LAG3,FNDC5,THY1,ONECUT1,DRGX,SOX7*
GO:0006357	regulation of transcription by RNA polymerase	0.010443043926274775	*FUS,CCN1,FOSL1,BRD4,HNRNPD,ATN1,HNRNPA2B1,FST,KHSRP,FOXP4,SLC9A1,ECM1,BRPF3,PPARD,PCBP1,MDC1,WIZ,PPP1R13L,PCGF2,CRTC2,BCL9L,NCOR2,DVL3,ZNF114,PLAGL2,NFIX,MED15,NFIC,NCOA6,ZMIZ2,MYD88,SKI,ZNF521,GSK3A,SCAF4,SP2,PBX2,CAMTA2,CRTC1,CC2D1A,ATF5,SORBS3,GATAD*

	II		*2B,BCORL1,DMAP1,MEF2D,HMOX1,EGR1,FOXQ1,ZNF580,GATA6,CREB3L1,PLK3,CCNK,GLI4,ZBED3,ZBTB12,MAGEC1,SOX12,ID4,MNT,BICRA,GLIS2,ZNF589,HSF4,KDM6B,PAX8,MAGEC2,TFAP2A,NR1D1,PDE2A,ZFP36,SIX2,SP140,ZNF296,S1PR1,ARID3B,GATA3,GATA2,IKZF4,SUPT4H1,RFXAP,EDN1,IGF2,MC1R,PRRX1,POU3F2,LIN37,FOSB,PARP15,MEIS1,TCF4,TRPV4,BMP6,MAMSTR,HLX,GREM1,FOXA3,ZNF257,ELL3,CHD5,YY2,HOXD1,ZNF492,FOXD4,FOXD4L1,EGR4,FOXO6,DUSP26,HOXB8,TBX6,DLX4,ZNF716,PLAGL1,ONECUT1,DRGX,SOX7*
GO:0016477	cell migration	0.010571408589225954	*CCN1,MYADM,RIPOR1,S100A11,CCN3,PXN,ATN1,PTPN23,CD44,SLC9A1,ECM1,PPARD,LAMB3,LOX,ADAMTS1,MYD88,RHOJ,GSK3A,PRSS3,CXCR4,RERE,PHACTR4,CCBE1,HMOX1,COL1A1,NGFR,ZNF580,RELN,RAB13,FAT3,SPHK1,ZNF703,RIN3,ILK,ARC,SIX2,BMPER,TNFSF12,PDGFD,S1PR1,LTB4R2,GATA3,GATA2,EDN1,MMP2,SEMA6A,CORO1A,DDT,SERPINE1,POU3F2,CDH19,WASHC1,MMP3,ACTA2,LRRC15,PTPN22,TRPV4,CLDN1,KIRREL3,PHACTR1,TGFBR3L,GPR15,DPYSL3,LAMA1,DBH,CD177,GREM1,SRCIN1,COL3A1,ARHGEF16,SFRP1,CDK5R2,TIE1,KIAA0319,MIF,THY1,ONECUT1,DRGX*
GO:2001141	regulation of RNA biosynthetic process	0.011444701715907139	*FUS,CCN1,FOSL1,UTP4,BRD4,SF1,HNRNPD,TAF15,ATN1,HNRNPA2B1,FST,KHSRP,FOXP4,SLC9A1,ECM1,BRPF3,PPARD,CCDC124,PCBP1,MDC1,WIZ,PPP1R13L,PCGF2,CRTC2,BCL9L,NCOR2,DVL3,ZNF114,PLAGL2,NFIX,MED15,NFIC,NCOA6,ZMIZ2,TP53INP2,MYD88,SKI,MAP2K7,CXXC1,ZNF521,GSK3A,SCAF4,SP2,PBX2,CAMTA2,CNOT3,CRTC1,CC2D1A,ATF5,SORBS3,CDK11B,RERE,GATAD2B,BCORL1,DMAP1,MEF2D,HMOX1,AFF3,EGR1,COL1A1,NGFR,TENM1,FOXQ1,ZNF580,GATA6,CREB3L1,DMD,PLK3,CCNK,GLI4,ZBED3,MLLT11,SPHK1,ZBTB12,MAGEC1,SOX12,ID4,ZNF703,MNT,ILK,BICRA,GLIS2,ZNF589,HSF4,KDM6B,PAX8,MAGEC2,TFAP2A,NR1D1,PDE2A,ZFP36,SIX2,SP140,ZNF296,S1PR1,ARID3B,TRIM66,HMGN5,GATA3,GATA2,IKZF4,LOXL3,SUPT4H1,RFXAP,EDN1,APBB3,IGF2,CDK11A,MC1R,ZNF414,PAK6,PRRX1,ARL2BP,POU3F2,LIN37,FOSB,PARP15,MEIS1,TCF4,TRPV4,BMP6,MAMSTR,SETBP1,HLX,GREM1,FOXA3,ZNF257,ELL3,CHD5,YY2,HOXD1,ZNF492,FOXD4,NELFCD,FOXD4L1,EGR4,SFRP1,FOXO6,DUSP26,HOXB8,TBX6,DLX4,ZNF716,PLAGL1,ONECUT1,DRGX,SOX7*
GO:0065008	regulation of biological quality	0.011732885885400791	*FUS,MYADM,MT-CO2,PABPC1,PRKACA,CCN3,HNRNPD,DIAPH1,TAF15,RAB11B,RAB7A,ADM,KHSRP,AKT1S1,SLC29A1,SLC9A1,ZFP36L1,DLGAP4,WIZ,ESAM,C4BPB,DTNBP1,PDCL3,VAC14,NCOR2,SAMD4B,DVL3,BAK1,VAMP2,MYD88,RHOJ,GSK3A,PLEKHA7,CXCR4,UCP2,CNOT3,CRTC1,CC2D1A,ALDOA,DGAT1,EGR1,GABARAP,SYVN1,TENM1,STXBP1,DMD,NLGN2,RELN,FURIN,PEAR1,CHRDL1,PRR16,NAA80,PORCN,ZNF385A,ZBED3,MLLT11,PLEKHN1,SYNGAP1,RIN3,RGS4,ILK,SLC9A5,GABRE,PAX8,GPRC5B,CACNG7,NR1D1,ARC,PDE2A,ZFP36,CYP1A1,ATP6V0C,GATA3,GATA2,SCNN1D,KCNK1,EDN1,MMP2,CORO1A,SLITRK1,PRKCG,RAB3A,SERPINB2,PRRT1,RNASEK,SORCS3,ASIC1,GABRA3,SERPINE1,RANGRF,ACTA2,COL1A2,CYRIA,S1PR3,PRRT2,TRPV4,BMP6,GPR15,KCNK2,GPR35,DBH,NRXN3,ANO7,BCHE,KCNIP2,ATP1A3,TPH1,KCNC3,SRCIN1,COL3A1,WNK4,IL1RN,SFRP1,SPNS1,PCSK1N,GRIN2A,HSD11B2,HCN2,SULT1B1,KCNE5,ADH6,GRIN3B,ADGRB3,RAPSN,KIAA0319,SLC7A8,KCND1,SPINK1,NPFF*

GO:1901700	response to oxygen-containing compound	0.011927074236893206	*MT-CYB,MT-ND5,MT-ND4,MT-ND1,MT-ND6,FOSL1,PRKACA,MAT2A,HNRNPD,PXN,RAB11B,TYMS,ADM,NUDC,SLC29A1,SLC9A1,ZFP36L1,CDA,MAPK13,PCGF2,DTNBP1,NCOR2,BAK1,VAMP2,GPX3,CDKN1A,MYD88,MAP2K7,GSK3A,CXCR4,UCP2,EIF2B4,DMAP1,HMOX1,EGR1,COL1A1,NGFR,ZNF580,GATA6,STXBP1,PLK3,ZBED3,RAB13,DAPK1,SPHK1,ZNF703,RGS4,KDM6B,LDOC1,NR1D1,ARC,PDE2A,ZFP36,CYP1A1,PDGFD,ARSA,SCNN1D,EDN1,IGF2,MMP2,GCKR,SERPINE1,FOSB,MMP3,PTPN22,TRPV4,BMP6,CLDN1,KCNK2,DBH,BCHE,ATP1A3,GJB3,COL3A1,SFRP1,UCP3,TIE1,GRIN2A,HSD11B2,HCN2,MAOB,SPINK1,MIF*
GO:0045893	positive regulation of DNA-templated transcription	0.012431035095977061	*FUS,CCN1,FOSL1,BRD4,HNRNPD,TAF15,ATN1,SLC9A1,PPARD,CCDC124,PCBP1,MDC1,CRTC2,BCL9L,DVL3,PLAGL2,NFIX,MED15,NFIC,NCOA6,ZMIZ2,TP53INP2,MYD88,SKI,MAP2K7,CXXC1,ZNF521,GSK3A,PBX2,CAMTA2,CRTC1,ATF5,RERE,GATAD2B,DMAP1,MEF2D,EGR1,COL1A1,NGFR,ZNF580,GATA6,CREB3L1,CCNK,ZBED3,MLLT11,SOX12,ID4,ILK,BICRA,GLIS2,KDM6B,PAX8,TFAP2A,NR1D1,SIX2,S1PR1,ARID3B,HMGN5,GATA3,GATA2,IKZF4,SUPT4H1,RFXAP,EDN1,IGF2,MC1R,PRRX1,ARL2BP,POU3F2,FOSB,MEIS1,TCF4,BMP6,MAMSTR,GREM1,FOXA3,ELL3,YY2,EGR4,SFRP1,TBX6,PLAGL1,ONECUT1,SOX7*
GO:1902680	positive regulation of RNA biosynthetic process	0.012998996986022516	*FUS,CCN1,FOSL1,BRD4,HNRNPD,TAF15,ATN1,SLC9A1,PPARD,CCDC124,PCBP1,MDC1,CRTC2,BCL9L,DVL3,PLAGL2,NFIX,MED15,NFIC,NCOA6,ZMIZ2,TP53INP2,MYD88,SKI,MAP2K7,CXXC1,ZNF521,GSK3A,PBX2,CAMTA2,CRTC1,ATF5,RERE,GATAD2B,DMAP1,MEF2D,EGR1,COL1A1,NGFR,ZNF580,GATA6,CREB3L1,CCNK,ZBED3,MLLT11,SOX12,ID4,ILK,BICRA,GLIS2,KDM6B,PAX8,TFAP2A,NR1D1,SIX2,S1PR1,ARID3B,HMGN5,GATA3,GATA2,IKZF4,SUPT4H1,RFXAP,EDN1,IGF2,MC1R,PRRX1,ARL2BP,POU3F2,FOSB,MEIS1,TCF4,BMP6,MAMSTR,GREM1,FOXA3,ELL3,YY2,EGR4,SFRP1,TBX6,PLAGL1,ONECUT1,SOX7*
GO:0001666	response to hypoxia	0.01319093255311506	*MT-CO2,MT-CYB,MT-ND2,MT-ND5,MT-CO1,MT-ND4,MT-ND1,ADM,SLC29A1,SLC9A1,PPARD,ZFP36L1,CXCR4,UCP2,EGR1,GATA6,PLEKHN1,CYP1A1,EDN1,MMP2,TRPV4,KCNK2,SFRP1,UCP3,HSD11B2*
GO:0007267	cell-cell signaling	0.014415443017379664	*PRKACA,CCN3,USP14,PTPN23,RAB11B,SLC29A1,ZYX,DTNBP1,VAMP2,GSK3A,UCP2,GABARAP,NGFR,DTNA,STXBP1,DMD,NLGN2,RELN,CHRDL1,PORCN,SYNGAP1,RGS4,GABRE,FABP5,PAX8,CACNG7,NR1D1,ARC,SYT11,GATA3,CTF1,EDN1,AMH,PRKCG,RAB3A,PRRT1,SORCS3,ASIC1,GABRA3,RANGRF,PRRT2,TRPV4,BMP6,KCNK2,PTHLH,DBH,GREM1,NRXN3,BCHE,KCNIP2,GJB3,WNK4,IL1RN,SFRP1,PANX2,GRIN2A,HCN2,CGB7,PCDHB2,KCNE5,PCDHB6,MAOB,GRIN3B,RAPSN,AMPH,SPINK1,MIF,THY1,NPFF,CGB8*
GO:0006812	monoatomic cation transport	0.015130805200540722	*MT-CO2,MT-CYB,MT-ND2,MT-ND5,MT-CO1,MT-ND3,MT-ND4,MT-ND1,MT-ATP6,MT-ND4L,FTH1,MT-ND6,MT-ATP8,PRKACA,FXYD5,MT-CO3,FKBP1A,DIAPH1,RAB11B,CHERP,SLC9A1,BAK1,VAMP2,MCOLN1,CXCR4,UCP2,DMD,SLC38A7,SLC39A4,RGS4,SLC9A5,CACNG7,ATP6V0C,SCNN1D,KCNK1,EDN1,CORO1A,RNASEK,ASIC1,RANGRF,PTPN22,TRPV4,KCNK2,GPR35,KCNIP2,ATP1A3,KCNC3,WNK4,KCNAB3,ATP6V1G2,PANX2,UCP3,GRIN2A,HCN2,KCNE5,GRIN3B,KCND1,SPINK1,THY1*
GO:0030	cell	0.01517542	*CCN1,MYADM,RPS3A,ANP32B,HNRNPH3,PRKACA,BRD*

154	differentiation	9390429198	*4,CCN3,SF1,TYMS,FST,ADM,EHD2,SLC9A1,ECM1,BRPF3,PPARD,ZFP36L1,LAMB3,PPDPF,LOX,BYSL,PDLIM7,PPP1R13L,DTNBP1,BCL9L,SF3A2,DVL3,BAK1,UPF3B,NCOA6,CDKN1A,TP53INP2,MYD88,SKI,ZNF521,GSK3A,PBX2,CXCR4,UCP2,EIF2B4,CNOT3,TPP1,CC2D1A,ATF5,PHGDH,RERE,PHACTR4,GATAD2B,PABPC1L,MEF2D,SETD1A,EGR1,COL1A1,NGFR,SYVN1,TENM1,FOXQ1,GPR137,GATA6,CREB3L1,STXBP1,DMD,NLGN2,RELN,CHRDL1,YJEFN3,CTH,NHSL3,ZNF385A,RAB13,FAT3,OSGIN1,SOX12,SYNGAP1,ID4,ZNF703,RGS4,EVI2B,ILK,BICRA,FZD8,GLIS2,HSF4,TUBB3,KDM6B,PAX8,TFAP2A,GPRC5B,CACNG7,NR1D1,ARC,PDE2A,ZFP36,CYP1A1,SIX2,ZNF296,TNFSF12,S1PR1,GATA3,GATA2,LOXL3,MYO7B,CTF1,EDN1,AMH,IGF2,MMP2,SEMA6A,SLITRK1,RAB3A,CCDC78,PAK6,PRRX1,SERPINE1,POU3F2,ACTA2,PTPN22,MEIS1,S1PR3,TCF4,TRPV4,BMP6,CLDN1,KIRREL3,PHACTR1,SYPL2,ANPEP,TGFBR3L,DPYSL3,SNX10,MAMSTR,ANKLE1,HLX,PKDCC,LAMA1,PTHLH,GREM1,FOXA3,ANGPTL6,NRXN3,BCHE,KCNIP2,ELL3,CHD5,TPH1,HOXD1,NELL2,SRCIN1,FOXD4,COL3A1,STIMATE-MUSTN1,FOXD4L1,UCHL1,SFRP1,FLRT1,CDK5R2,TIE1,KRT14,FOXO6,GRIN2A,CNFN,SULT1B1,HOXB8,TBX6,EPHA6,DLX4,ADGRB3,TMEM190,TMEM119,KIAA0319,TCHH,ADAMTS5,SPINK1,ALOXE3,LAG3,FNDC5,THY1,ONECUT1,DRGX,SOX7*
GO:0023051	regulation of signaling	0.016034037046528737	*CCN1,ARHGDIA,RIPOR1,PRKACA,MAT2A,CTDNEP1,BRD4,CCN3,FKBP1A,PXN,PTPN23,AGPAT1,FST,RAB7A,CHERP,ADM,AKT1S1,CD44,SLC9A1,ECM1,PPARD,LOX,PCGF2,DTNBP1,BCL9L,NCOR2,DVL3,NPRL3,PLAGL2,BAK1,PDE4A,VAMP2,MYD88,SKI,MAP2K7,GSK3A,DUSP7,CXCR4,UCP2,CC2D1A,SORBS3,ZC3H3,PHACTR4,CCBE1,HMOX1,EGR1,COL1A1,GABARAP,NGFR,SYVN1,TENM1,GPR137,GATA6,CREB3L1,STXBP1,DMD,NLGN2,PLK3,RELN,CCNK,FURIN,CHRDL1,YJEFN3,DOK3,CTH,GPR3,LIMS2,ZNF385A,ZBED3,DAPK1,SPHK1,TAX1BP3,SYNGAP1,ZNF703,RGS4,MNT,ILK,GLIS2,NRK,GAREM1,FABP5,ARHGEF19,PAX8,GPRC5B,PSD4,CACNG7,NR1D1,ARC,PDE2A,SYT11,BMPER,TNFSF12,PDGFD,ATP6V0C,GATA3,GATA2,LOXL3,CNKSR2,EDN1,AMH,IGF2,MMP2,SEMA6A,MC1R,DDT,PRKCG,RAB3A,PAK6,PRRT1,PRRX1,SORCS3,ASIC1,SERPINE1,MMP3,ACTA2,PTPN22,PRRT2,TRPV4,BMP6,TGFBR3L,KCNK2,GPR35,LAMA1,DBH,TMEM198,CD177,GREM1,NRXN3,BCHE,ELL3,CHD5,COL3A1,UCHL1,ESM1,IL1RN,SFRP1,ARHGAP20,GRIN2A,DUSP26,GRIN3B,RAPSN,KIAA0319,SPINK1,MIF,ENHO,THY1,ONECUT1,SOX7*
GO:0010646	regulation of cell communication	0.01816348539797518	*CCN1,ARHGDIA,RIPOR1,PRKACA,MAT2A,CTDNEP1,BRD4,CCN3,FKBP1A,PXN,PTPN23,AGPAT1,FST,RAB7A,CHERP,ADM,AKT1S1,CD44,SLC9A1,ECM1,PPARD,LOX,PCGF2,DTNBP1,BCL9L,NCOR2,DVL3,NPRL3,PLAGL2,BAK1,PDE4A,VAMP2,MYD88,SKI,MAP2K7,GSK3A,DUSP7,CXCR4,UCP2,CC2D1A,SORBS3,ZC3H3,PHACTR4,CCBE1,HMOX1,EGR1,COL1A1,GABARAP,NGFR,SYVN1,TENM1,GPR137,GATA6,CREB3L1,STXBP1,DMD,NLGN2,PLK3,RELN,CCNK,FURIN,CHRDL1,YJEFN3,DOK3,CTH,GPR3,LIMS2,ZNF385A,ZBED3,DAPK1,SPHK1,TAX1BP3,SYNGAP1,ZNF703,RGS4,MNT,ILK,GLIS2,NRK,GAREM1,FABP5,ARHGEF19,PAX8,GPRC5B,PSD4,CACNG7,NR1D1,ARC,PDE2A,SYT11,BMPER,TNFSF12,PDGFD,ATP6V0C,GATA3,GATA2,LOXL3,CNKSR2,EDN1,AMH,IGF2,MMP2,SEMA6A,MC1R,DDT,PR*

			KCG,RAB3A,PAK6,PRRT1,PRRX1,SORCS3,ASIC1,SERPINE1,MMP3,ACTA2,PTPN22,PRRT2,TRPV4,BMP6,TGFBR3L,KCNK2,GPR35,LAMA1,DBH,TMEM198,CD177,GREM1,NRXN3,BCHE,ELL3,CHD5,COL3A1,UCHL1,ESM1,IL1RN,SFRP1,ARHGAP20,GRIN2A,DUSP26,GRIN3B,RAPSN,KIAA0319,SPINK1,MIF,ENHO,THY1,ONECUT1,SOX7
GO:0009966	regulation of signal transduction	0.019607053206721875	*CCN1,ARHGDIA,RIPOR1,PRKACA,MAT2A,CTDNEP1,BRD4,CCN3,FKBP1A,PXN,AGPAT1,FST,RAB7A,CHERP,ADM,AKT1S1,CD44,SLC9A1,ECM1,PPARD,LOX,PCGF2,DTNBP1,BCL9L,NCOR2,DVL3,NPRL3,PLAGL2,BAK1,PDE4A,MYD88,SKI,MAP2K7,GSK3A,DUSP7,CXCR4,CC2D1A,SORBS3,ZC3H3,PHACTR4,CCBE1,HMOX1,EGR1,COL1A1,GABARAP,NGFR,SYVN1,TENM1,GPR137,GATA6,CREB3L1,DMD,NLGN2,PLK3,RELN,CCNK,FURIN,CHRDL1,YJEFN3,DOK3,CTH,GPR3,LIMS2,ZNF385A,ZBED3,DAPK1,SPHK1,TAX1BP3,SYNGAP1,ZNF703,RGS4,MNT,ILK,GLIS2,NRK,GAREM1,FABP5,ARHGEF19,GPRC5B,PSD4,CACNG7,NR1D1,ARC,PDE2A,BMPER,TNFSF12,PDGFD,ATP6V0C,GATA3,GATA2,LOXL3,CNKSR2,EDN1,AMH,IGF2,MMP2,SEMA6A,MC1R,DDT,PAK6,PRRT1,PRRX1,SERPINE1,MMP3,ACTA2,PTPN22,TRPV4,BMP6,TGFBR3L,GPR35,LAMA1,DBH,TMEM198,CD177,GREM1,ELL3,CHD5,COL3A1,UCHL1,ESM1,IL1RN,SFRP1,ARHGAP20,GRIN2A,DUSP26,KIAA0319,SPINK1,MIF,ENHO,THY1,ONECUT1,SOX7*
GO:0048523	negative regulation of cellular process	0.020074234121184826	*FUS,CCN1,MYADM,RPS3A,FTH1,ANP32B,PABPC1,ARHGDIA,RIPOR1,FOSL1,PRKACA,FXYD5,S100A11,BRD4,CCN3,SF1,HNRNPD,FKBP1A,DIAPH1,TAF15,PRPF19,USP14,SMG7,ATN1,PTPN23,HNRNPA2B1,TYMS,FST,RAB7A,CIZ1,CHERP,ADM,KHSRP,FOXP4,AKT1S1,CD44,SLC9A1,PPP1R11,ECM1,PPARD,MIR137HG,ZFP36L1,CDA,MDC1,LOX,PPP1R13L,PCGF2,DTNBP1,PDCL3,BCL9L,NCOR2,SAMD4B,ADAMTS1,NPRL3,BAK1,UPF3B,NFIX,NFIC,PCIF1,CDKN1A,TP53INP2,EDC3,MYD88,MCOLN1,SKI,GSK3A,SCAF4,SP2,DUSP7,UPF3A,UCP2,CNOT3,RPS26,CC2D1A,ATF5,SORBS3,RERE,PHACTR4,GATAD2B,BCORL1,DMAP1,HMOX1,EGR1,COL1A1,NGFR,SYVN1,TENM1,FOXQ1,GPR137,GATA6,SERPINB8,CREB3L1,SCAMP5,STXBP1,DMD,PLK3,FURIN,CHRDL1,CTH,LIMS2,ZNF385A,ZBED3,FAT3,DAPK1,MRNIP,SPHK1,OSGIN1,ZBTB12,PLEKHN1,MAGEC1,TAX1BP3,SOX12,SYNGAP1,ID4,ZNF703,RIN3,RGS4,MNT,EVI2B,ILK,BICRA,GLIS2,ZNF589,HSF4,FABP5,LDOC1,PAX8,MAGEC2,TFAP2A,CACNG7,ANKRD13B,NR1D1,ARC,PDE2A,ZFP36,SYT11,SIX2,ZNF296,BMPER,S1PR1,TRIM66,HMGN5,GATA3,GATA2,IKZF4,LOXL3,SUPT4H1,EDN1,IGF2,MMP2,SEMA6A,MC1R,CORO1A,DDT,PRKCG,SERPINB2,PRRT1,PRRX1,MIR99AHG,SORCS3,ASIC1,SERPINE1,POU3F2,LIN37,FOSB,PARP15,WASHC1,MMP3,LRRC15,PTPN22,CST4,MEIS1,S1PR3,PRRT2,TRPV4,BMP6,BCL2L2-PABPN1,DPYSL3,KCNK2,GPR132,GPR35,ANKLE1,HLX,PKDCC,PTHLH,GREM1,LYPD3,BCHE,ELL3,CHD5,CRLF1,SRCIN1,COL3A1,SAMSN1,NELFCD,WNK4,UCHL1,IL1RN,SFRP1,PCSK1N,TIE1,GRIN2A,DUSP26,KCNE5,HOXB8,HUS1B,TBX6,MAOB,DLX4,TMEM119,KIAA0319,RBM20,ADAMTS5,PLAGL1,SPINK1,MIF,LAG3,THY1,ONECUT1,SOX7*
GO:0070848	response to growth factor	0.020881128297572826	*CCN1,FKBP1A,PXN,FST,AKT1S1,CD44,ZFP36L1,LOX,ZYX,BCL9L,SKI,CCBE1,EGR1,COL1A1,NGFR,GATA6,CREB3L1,DMD,FURIN,CHRDL1,SPHK1,ZNF703,ILK,GAREM1,PDE2A,ZFP36,BMPER,PDGFD,FNDC4,GA*

			TA3,EDN1,SEMA6A,CORO1A,ACTA2,COL1A2,BMP6,CLDN1,TGFBR3L,GREM1,COL3A1,SFRP1,FLRT1,MXRA5,ONECUT1
GO:0045944	positive regulation of transcription by RNA polymerase II	0.02120113396993068	*CCN1,FOSL1,BRD4,HNRNPD,SLC9A1,PPARD,PCBP1,MDC1,CRTC2,BCL9L,DVL3,PLAGL2,NFIX,MED15,NFIC,NCOA6,ZMIZ2,MYD88,SKI,ZNF521,GSK3A,PBX2,CAMTA2,CRTC1,ATF5,MEF2D,EGR1,ZNF580,GATA6,CREB3L1,CCNK,ZBED3,SOX12,ID4,GLIS2,KDM6B,PAX8,TFAP2A,NR1D1,SIX2,S1PR1,ARID3B,GATA3,GATA2,IKZF4,SUPT4H1,RFXAP,EDN1,IGF2,MC1R,PRRX1,POU3F2,FOSB,MEIS1,TCF4,BMP6,MAMSTR,GREM1,FOXA3,ELL3,YY2,EGR4,TBX6,PLAGL1,ONECUT1,SOX7*
GO:0050794	regulation of cellular process	0.022029727965232074	*FUS,CCN1,EIF5B,EIF1,MYADM,RPS3A,FTH1,ANP32B,PABPC1,HNRNPH3,ARHGDIA,RIPOR1,FOSL1,UTP4,PRKACA,MAT2A,FXYD5,SF3B4,CTDNEP1,S100A11,BRD4,CCN3,SF1,HNRNPD,FKBP1A,DIAPH1,TAF15,PRPF19,USP14,SMG7,PXN,ATN1,PTPN23,HNRNPA2B1,RAB11B,AGPAT1,TYMS,FST,RAB7A,CIZ1,CHERP,ADM,KHSRP,PSMD2,FOXP4,AKT1S1,EHD2,SLC29A1,CD44,ODC1,SLC9A1,PPP1R11,ECM1,BRPF3,PPARD,MIR137HG,ZFP36L1,DLGAP4,CCDC124,PCBP1,CDA,MDC1,WIZ,PPDPF,LOX,ZYX,MAPK13,BYSL,ESAM,PPP1R13L,PCGF2,C4BPB,CRTC2,DTNBP1,PSMC1,PDCL3,SRRM1,BCL9L,VAC14,SF3A2,NCOR2,SAMD4B,DVL3,ADAMTS1,ZNF114,NPRL3,PLAGL2,BAK1,UPF3B,NFIX,MED15,NFIC,NCOA6,PDE4A,VAMP2,ZMIZ2,PCIF1,CDKN1A,TP53INP2,EDC3,PRCC,MYD88,MCOLN1,SKI,GNG5,MAP2K7,CXXC1,RHOJ,PDE1C,ZNF521,GSK3A,SCAF4,SP2,PBX2,CAMTA2,DUSP7,CXCR4,UPF3A,UCP2,EIF2B4,CNOT3,RPS26,CRTC1,CC2D1A,ATF5,SORBS3,CDK11B,PHGDH,ZC3H3,NRGN,RERE,PHACTR4,GATAD2B,BCORL1,DMAP1,CCBE1,PABPC1L,BET1L,MEF2D,HMOX1,SETD1A,AFF3,EGR1,FAM98B,COL1A1,GABARAP,NGFR,SYVN1,NSRP1,TENM1,FOXQ1,GPR137,LSM10,DTNA,ZNF580,ESRP2,GATA6,SERPINB8,CREB3L1,SCAMP5,STXBP1,EIF3C,UCA1,DMD,NLGN2,PLK3,RELN,MMP1,CCNK,LTB4R,FURIN,PEAR1,EIF5AL1,CHRDL1,YJEFN3,DOK3,CTH,GPR3,RTKN,PRR16,GLI4,LIMS2,NAA80,DOCK6,PORCN,ZNF385A,ZBED3,RAB13,FAT3,MLLT11,DAPK1,MRNIP,SPHK1,OSGIN1,ZBTB12,PLEKHN1,MAGEC1,TAX1BP3,SOX12,SYNGAP1,ID4,ZNF703,RIN3,RGS4,MNT,EVI2B,ILK,BICRA,FZD8,GLIS2,ZNF589,HSF4,TUBB3,NRK,GAREM1,KDM6B,FABP5,ARHGEF19,LDOC1,PAX8,MAGEC2,TFAP2A,TNFSF15,GPRC5B,PSD4,CACNG7,ANKRD13B,NR1D1,ARC,PDE2A,ZFP36,CYP1A1,SYT11,SIX2,SP140,ZNF296,BMPER,TNFSF12,PDGFD,S1PR1,LRRC2,HSPB8,LTB4R2,ARID3B,TRIM66,ATP6V0C,HMGN5,GATA3,GATA2,IKZF4,LOXL3,CNKSR2,SUPT4H1,DHPS,TRNP1,RFXAP,CTF1,EDN1,AMH,APBB3,IGF2,CDK11A,MMP2,SEMA6A,MC1R,CORO1A,ZNF414,SLITRK1,DDT,PRKCG,RAB3A,SERPINB2,PAK6,ADGRE1,PRRT1,LCAT,PRRX1,MIR99AHG,ARL2BP,SORCS3,RAPGEFL1,ASIC1,SERPINE1,POU3F2,LIN37,FAM83A,FOSB,PARP15,RANGRF,GNG10,RHBDL1,WASHC1,MMP3,ACTA2,LRRC15,PTPN22,COL1A2,CYRIA,CST4,MEIS1,S1PR3,TCF4,PRRT2,TRPV4,BMP6,CLDN1,BCL2L2-PABPN1,PHACTR1,ASB16,TGFBR3L,GPR15,DPYSL3,KCNK2,OR2B6,GPR132,MAMSTR,GPR35,SETBP1,GNG13,TRBV12-4,ANKLE1,HLX,GNMT,PKDCC,LAMA1,PTHLH,DBH,TMEM198,CD177,GREM1,FOXA3,LYPD3,ZNF257,NRXN3*

			,BCHE,TAS2R5,SPDYE12,KCNIP2,TNFRSF25,ELL3,LILRA6,CHD5,TPH1,NUSAP1,YY2,HOXD1,CRLF1,RCAN2,SRCIN1,ZNF492,FOXD4,VSTM1,COL3A1,SAMSN1,NELFCD,FOXD4L1,EGR4,WNK4,GPR146,ARHGEF16,UCHL1,ESM1,IL1RN,SFRP1,KCNAB3,ATP6V1G2,GNRH2,SERPINB7,EBI3,ARHGAP20,PANX2,FLRT1,PCSK1N,CDK5R2,TIE1,FOXO6,PLCXD3,GRIN2A,HLA-DOB,HCN2,CGB7,SERPINB5,DUSP26,KCNE5,HOXB8,HUS1B,TBX6,MAOB,EPHA6,GRIN3B,DLX4,ADGRB3,ZNF716,TMEM119,RAPSN,KIAA0319,TMED7-TICAM2,RBM20,ADAMTS5,PLAGL1,GPR87,SPINK1,MIF,ENHO,ALOXE3,LAG3,FNDC5,GKN2,TNFRSF13C,GPRC5D,THY1,ONECUT1,NPFF,DRGX,CGB8,SOX7
GO:0045471	response to ethanol	0.022164861906758423	MT-CYB,MT-ND4,TYMS,BAK1,MYD88,DMAP1,RGS4,ARC,ARSA,FOSB,CLDN1,DBH,GRIN2A,MAOB,SPINK1
GO:0000302	response to reactive oxygen species	0.0229982766339583	MT-ND5,MT-ND6,FOSL1,PXN,MAPK13,PCGF2,BAK1,UCP2,HMOX1,COL1A1,ZNF580,PLK3,SPHK1,KDM6B,PDGFD,EDN1,MMP2,MMP3,UCP3
GO:0000122	negative regulation of transcription by RNA polymerase II	0.023516740505188444	ATN1,HNRNPA2B1,FST,FOXP4,PPARD,PPP1R13L,PCGF2,NCOR2,NFIX,NFIC,SKI,SCAF4,SP2,CC2D1A,SORBS3,GATAD2B,BCORL1,DMAP1,EGR1,FOXQ1,GATA6,CREB3L1,PLK3,ZBTB12,MAGEC1,SOX12,ID4,MNT,GLIS2,ZNF589,HSF4,MAGEC2,TFAP2A,NR1D1,PDE2A,ZFP36,ZNF296,GATA3,GATA2,SUPT4H1,EDN1,IGF2,PRRX1,LIN37,FOSB,PARP15,TRPV4,BMP6,DUSP26,HOXB8,TBX6,DLX4,PLAGL1
GO:0007154	cell communication	0.02547941170337644	CCN1,MYADM,ARHGDIA,RIPOR1,FOSL1,PRKACA,MAT2A,CTDNEP1,S100A11,BRD4,CCN3,FKBP1A,PRPF19,USP14,PXN,PTPN23,RAB11B,AGPAT1,FST,RAB7A,CHERP,ADM,AKT1S1,SLC29A1,CD44,SLC9A1,ECM1,PPARD,ZFP36L1,CDA,MDC1,PPDPF,LOX,ZYX,MAPK13,PCGF2,DTNBP1,BCL9L,VAC14,NCOR2,DVL3,ADAMTS1,NPRL3,PLAGL2,BAK1,PDE4A,VAMP2,CDKN1A,MYD88,MCOLN1,SKI,GNG5,MAP2K7,RHOJ,PDE1C,GSK3A,DUSP7,CXCR4,UCP2,EIF2B4,CC2D1A,SORBS3,ZC3H3,NRGN,PHACTR4,CCBE1,HMOX1,EGR1,COL1A1,GABARAP,NGFR,SYVN1,TENM1,GPR137,DTNA,GATA6,CREB3L1,STXBP1,DMD,NLGN2,PLK3,RELN,CCNK,LTB4R,FURIN,PEAR1,CHRDL1,YJEFN3,DOK3,CTH,GPR3,RTKN,LIMS2,DOCK6,PORCN,ZNF385A,ZBED3,RAB13,MLLT11,DAPK1,MRNIP,SPHK1,OSGIN1,TAX1BP3,SYNGAP1,ZNF703,RIN3,RGS4,MNT,ILK,FZD8,GLIS2,TUBB3,NRK,GAREM1,GABRE,FABP5,ARHGEF19,PAX8,TNFSF15,GPRC5B,PSD4,CACNG7,NR1D1,ARC,PDE2A,ZFP36,SYT11,BMPER,TNFSF12,PDGFD,S1PR1,LRRC2,LTB4R2,ATP6V0C,GATA3,GATA2,LOXL3,CNKSR2,CTF1,EDN1,AMH,IGF2,MMP2,SEMA6A,MC1R,CORO1A,DDT,PRKCG,RAB3A,PAK6,ADGRE1,PRRT1,PRRX1,ARL2BP,SORCS3,RAPGEFL1,ASIC1,GABRA3,SERPINE1,FAM83A,RANGRF,GNG10,RHBDL1,MMP3,ACTA2,PTPN22,COL1A2,S1PR3,PRRT2,TRPV4,BMP6,BCL2L2-PABPN1,ASB16,TGFBR3L,GPR15,KCNK2,OR2B6,GPR132,GPR35,GNG13,TRBV12-4,OIP5,HLX,LAMA1,PTHLH,DBH,TMEM198,CD177,GREM1,NRXN3,BCHE,TAS2R5,KCNIP2,ATP1A3,TNFRSF25,ELL3,LILRA6,CHD5,CRLF1,GJB3,RCAN2,VSTM1,COL3A1,WNK4,GPR146,UCHL1,ESM1,IL1RN,SFRP1,GNRH2,EBI3,ARHGAP20,PANX2,FLRT1,PCSK1N,TIE1,PLCXD3,GRIN2A,HCN2,CGB7,PCDHB2,DUSP26,

			KCNE5,PCDHB6,HUS1B,TBX6,MAOB,EPHA6,GRIN3B,ADGRB3,RAPSN,KIAA0319,AMPH,TMED7-TICAM2,GPR87,SPINK1,MIF,ENHO,ALOXE3,LAG3,FNDC5,TNFRSF13C,GPRC5D,THY1,ONECUT1,NPFF,CGB8,SOX7
GO:0023052	signaling	0.02737722732615492	*CCN1,MYADM,ARHGDIA,RIPOR1,FOSL1,PRKACA,MAT2A,CTDNEP1,S100A11,BRD4,CCN3,FKBP1A,PRPF19,USP14,PXN,PTPN23,RAB11B,AGPAT1,FST,RAB7A,CHERP,ADM,AKT1S1,SLC29A1,CD44,SLC9A1,ECM1,PPARD,ZFP36L1,DLGAP4,CDA,MDC1,PPDPF,LOX,ZYX,MAPK13,PCGF2,DTNBP1,BCL9L,VAC14,NCOR2,DVL3,ADAMTS1,NPRL3,PLAGL2,BAK1,PDE4A,VAMP2,CDKN1A,MYD88,MCOLN1,SKI,GNG5,MAP2K7,RHOJ,PDE1C,GSK3A,DUSP7,CXCR4,UCP2,EIF2B4,CC2D1A,SORBS3,ZC3H3,NRGN,PHACTR4,CCBE1,HMOX1,EGR1,COL1A1,GABARAP,NGFR,SYVN1,TENM1,GPR137,DTNA,GATA6,CREB3L1,STXBP1,DMD,NLGN2,PLK3,RELN,CCNK,LTB4R,FURIN,PEAR1,CHRDL1,YJEFN3,DOK3,CTH,GPR3,RTKN,LIMS2,DOCK6,PORCN,ZNF385A,ZBED3,RAB13,MLLT11,DAPK1,MRNIP,SPHK1,OSGIN1,TAX1BP3,SYNGAP1,ZNF703,RIN3,RGS4,MNT,ILK,FZD8,GLIS2,TUBB3,NRK,GAREM1,GABRE,FABP5,ARHGEF19,PAX8,TNFSF15,GPRC5B,PSD4,CACNG7,NR1D1,ARC,PDE2A,ZFP36,SYT11,BMPER,TNFSF12,PDGFD,S1PR1,LRRC2,LTB4R2,ATP6V0C,GATA3,GATA2,LOXL3,CNKSR2,CTF1,EDN1,AMH,IGF2,MMP2,SEMA6A,MC1R,CORO1A,DDT,PRKCG,RAB3A,PAK6,ADGRE1,PRRT1,PRRX1,ARL2BP,SORCS3,RAPGEFL1,ASIC1,GABRA3,SERPINE1,FAM83A,RANGRF,GNG10,RHBDL1,MMP3,ACTA2,PTPN22,COL1A2,S1PR3,PRRT2,TRPV4,BMP6,BCL2L2-PABPN1,ASB16,TGFBR3L,GPR15,KCNK2,OR2B6,GPR132,GPR35,GNG13,TRBV12-4,HLX,LAMA1,PTHLH,DBH,TMEM198,CD177,GREM1,NRXN3,BCHE,TAS2R5,KCNIP2,TNFRSF25,ELL3,LILRA6,CHD5,CRLF1,GJB3,RCAN2,VSTM1,COL3A1,WNK4,GPR146,UCHL1,ESM1,IL1RN,SFRP1,GNRH2,EBI3,ARHGAP20,PANX2,FLRT1,PCSK1N,TIE1,PLCXD3,GRIN2A,HCN2,CGB7,PCDHB2,DUSP26,KCNE5,PCDHB6,HUS1B,TBX6,MAOB,EPHA6,GRIN3B,ADGRB3,RAPSN,KIAA0319,AMPH,TMED7-TICAM2,GPR87,SPINK1,MIF,ENHO,ALOXE3,LAG3,FNDC5,TNFRSF13C,GPRC5D,THY1,ONECUT1,NPFF,CGB8,SOX7*
GO:0036293	response to decreased oxygen levels	0.02881115428123199	*MT-CO2,MT-CYB,MT-ND2,MT-ND5,MT-CO1,MT-ND4,MT-ND1,ADM,SLC29A1,SLC9A1,PPARD,ZFP36L1,CXCR4,UCP2,EGR1,GATA6,PLEKHN1,CYP1A1,EDN1,MMP2,TRPV4,KCNK2,SFRP1,UCP3,HSD11B2*
GO:0051253	negative regulation of RNA metabolic process	0.03167869675718867	*FUS,PABPC1,SF1,HNRNPD,TAF15,ATN1,HNRNPA2B1,FST,FOXP4,PPARD,PPP1R13L,PCGF2,NCOR2,NFIX,NFIC,MYD88,SKI,SCAF4,SP2,UPF3A,CC2D1A,ATF5,SORBS3,RERE,GATAD2B,BCORL1,DMAP1,EGR1,FOXQ1,GATA6,CREB3L1,PLK3,ZBTB12,MAGEC1,SOX12,ID4,ZNF703,MNT,GLIS2,ZNF589,HSF4,MAGEC2,TFAP2A,NR1D1,PDE2A,ZFP36,ZNF296,TRIM66,GATA3,GATA2,IKZF4,LOXL3,SUPT4H1,EDN1,IGF2,PRRX1,LIN37,FOSB,PARP15,TRPV4,BMP6,GREM1,NELFCD,SFRP1,DUSP26,HOXB8,TBX6,DLX4,RBM20,PLAGL1,SOX7*
GO:0034220	monoatomic ion transmembr	0.033176608908874104	*MT-CO2,MT-CYB,MT-ND2,MT-ND5,MT-CO1,MT-ND3,MT-ND4,MT-ND1,MT-ATP6,MT-ND4L,MT-ND6,MT-ATP8,PRKACA,MT-*

	ane transport		CO3,FKBP1A,DIAPH1,CHERP,SLC9A1,BAK1,VAMP2,MCOLN1,UCP2,DMD,SLC39A4,RGS4,SLC9A5,GABRE,CACNG7,ARC,ATP6V0C,SCNN1D,KCNK1,EDN1,CORO1A,RNASEK,ASIC1,GABRA3,RANGRF,PTPN22,TRPV4,KCNK2,GPR35,ANO7,KCNIP2,ATP1A3,KCNC3,WNK4,KCNAB3,ATP6V1G2,PANX2,UCP3,GRIN2A,HCN2,KCNE5,GRIN3B,KCND1,THY1
GO:0071363	cellular response to growth factor stimulus	0.034084958571250885	CCN1,FKBP1A,PXN,FST,AKT1S1,CD44,ZFP36L1,LOX,ZYX,BCL9L,SKI,CCBE1,EGR1,COL1A1,NGFR,GATA6,CREB3L1,DMD,FURIN,CHRDL1,SPHK1,ZNF703,ILK,GAREM1,PDE2A,ZFP36,BMPER,PDGFD,GATA3,EDN1,SEMA6A,CORO1A,ACTA2,COL1A2,BMP6,CLDN1,TGFBR3L,GREM1,COL3A1,SFRP1,FLRT1,ONECUT1
GO:0006810	transport	0.03432563548583034	MT-CO2,MT-CYB,MT-ND2,MT-ND5,MT-CO1,MT-ND3,MT-ND4,MT-ND1,MT-ATP6,MT-ND4L,FTH1,MT-ND6,ANP32B,ARHGDIA,RIPOR1,MT-ATP8,PRKACA,FXYD5,MT-CO3,CCN3,FKBP1A,DIAPH1,DDX39B,SMG7,PTPN23,HNRNPA2B1,RAB11B,RAB7A,CHERP,ADM,KHSRP,EHD2,NUDC,SLC29A1,G6PC3,SLC9A1,PPARD,NXF1,ZFP36L1,PDLIM7,C4BPB,DTNBP1,VAC14,NCOR2,BAK1,UPF3B,POM121C,TRAPPC1,STX16,EPS15L1,VAMP2,CDKN1A,MYD88,MCOLN1,RHOJ,GSK3A,TINAGL1,CXCR4,UPF3A,UCP2,ZC3H3,DMAP1,BET1L,COL1A1,GABARAP,NGFR,SYVN1,TENM1,POM121,CREB3L1,SCAMP5,STXBP1,DMD,NLGN2,PLK3,RELN,SLC38A7,FURIN,ALS2CL,PEAR1,SLC39A4,YJEFN3,PORCN,PRAF2,RAB13,TOMM5,DAPK1,SPHK1,RASEF,SLC29A3,RIN3,RGS4,SLC9A5,MFSD3,GABRE,FABP5,PAX8,CACNG7,ANKRD13B,NR1D1,ARC,ZFP36,SYT11,SIX2,SLC44A5,ATP6V0C,GATA3,GATA2,SCNN1D,MYO7B,KCNK1,NCF2,EDN1,APBB3,CORO1A,PRKCG,GCKR,RAB3A,SLC22A15,PRRT1,RNASEK,LCAT,ANXA8L1,SEC31B,ASIC1,GABRA3,SERPINE1,SLC52A3,RANGRF,WASHC1,NPIPA1,PTPN22,PRRT2,TRPV4,BMP6,CLDN1,RAB3IL1,KCNK2,SNX10,GPR35,ANKLE1,PKDCC,CD177,GREM1,OLR1,SYT15,NRXN3,ANO7,KCNIP2,ATP1A3,TPH1,KCNC3,GJB3,SRCIN1,TMED6,WNK4,UCHL1,IL1RN,SFRP1,KCNAB3,ATP6V1G2,PANX2,SPNS1,UCP3,CDK5R2,GRIN2A,HCN2,KCNE5,CPT1B,MAOB,GRIN3B,AMPH,TMED7-TICAM2,SLC7A8,KCND1,SPINK1,MIF,THY1
GO:0048545	response to steroid hormone	0.035375049814645954	MT-ND3,FOSL1,TYMS,ADM,PPARD,ZFP36L1,LOX,NCOR2,CDKN1A,GSK3A,UCP2,COL1A1,NR1D1,ZFP36,SCNN1D,EDN1,FOSB,BMP6,CLDN1,BCHE,ATP1A3,IL1RN,SFRP1,UCP3,HSD11B2,MAOB
GO:0008283	cell population proliferation	0.036363420032163425	CCN1,FTH1,FOSL1,S100A11,CCN3,SF1,FST,CHERP,ADM,ODC1,ECM1,PPARD,ZFP36L1,BYSL,PDCL3,ADAMTS1,BAK1,CDKN1A,MYD88,SKI,GNG5,ATF5,RERE,MEF2D,HMOX1,EGR1,FAM98B,NGFR,TENM1,ZNF580,ESRP2,GATA6,NLGN2,YJEFN3,LIMS2,SPHK1,TAX1BP3,ID4,ZNF703,ILK,BICRA,GAREM1,LDOC1,TFAP2A,NR1D1,ZFP36,SIX2,BMPER,TNFSF12,PDGFD,S1PR1,HMGN5,GATA3,GATA2,DHPS,TRNP1,CTF1,EDN1,IGF2,MMP2,CORO1A,PRRX1,POU3F2,FAM83A,PTPN22,MEIS1,S1PR3,BMP6,CLDN1,KCNK2,HLX,PTHLH,DBH,GREM1,BCHE,ELL3,CHD5,CRLF1,COL3A1,STIMATE-MUSTN1,EGR4,UCHL1,ESM1,SFRP1,SERPINB7,EBI3,TIE1,SERPINB5,TMEM119,SPINK1,MIF,GKN2,TNFRSF13C,SOX7
GO:0019	aerobic	0.04009233	MT-CO2,MT-CYB,MT-ND2,MT-ND5,MT-CO1,MT-ND3,MT-ND4,MT-ND1,MT-ND4L,MT-ND6,MT-

646	electron transport chain	561272127	*CO3,COX7B2*
GO:0060429	epithelium development	0.04798360956385631	*CCN1,MYADM,HNRNPH3,PRKACA,TYMS,FST,ADM,CD44,ZFP36L1,CDKN1A,MYD88,SKI,GSK3A,CXCR4,TPP1,PHGDH,PHACTR4,NGFR,FOXQ1,ESRP2,GATA6,DMD,RAB13,ID4,ZNF703,ILK,HSF4,KDM6B,PAX8,TFAP2A,PDE2A,ZFP36,CYP1A1,SIX2,BMPER,S1PR1,GATA3,GATA2,LOXL3,EDN1,MMP2,CCDC78,SERPINE1,POU3F2,ACTA2,S1PR3,CYSRT1,BMP6,CLDN1,INKA1,LAMA1,GREM1,CRLF1,WNK4,SFRP1,TIE1,KRT14,CNFN,SERPINB5,SULT1B1,TBX6,TCHH,ALOXE3,ONECUT1*
		CC	
GO:0030054	cell junction	0.000003945413340296 0194	*FUS,RPS16,EIF5B,RPS27,MYADM,RPS3A,PABPC1,ARHGDIA,RPL21,RPL7,FOSL1,PRKACA,RPL9,S100A11,CCN3,RPS28,HNRNPD,USP14,PXN,ATN1,RAB11B,RAB7A,SLC29A1,CD44,SLC9A1,DLGAP4,PCBP1,MDC1,ZYX,PDLIM7,ESAM,PPP1R13L,DTNBP1,MAL2,VAC14,STX16,VAMP2,GSK3A,PLEKHA7,CXCR4,RPS26,CC2D1A,SORBS3,GABARAP,NGFR,FHL3,DTNA,SCAMP5,STXBP1,CAMK2N1,CGN,DMD,NLGN2,CHRDL1,LIMS2,PORCN,PRAF2,RAB13,DAPK1,SPHK1,SYNGAP1,ILK,SLC9A5,GABRE,FABP5,CACNG7,NR1D1,NPTXR,ARC,PDE2A,SYT11,S1PR1,ATP6V0C,CNKSR2,KCNK1,CORO1A,SLITRK1,PRKCG,RAB3A,PAK6,PRRT1,SORCS3,ASIC1,GABRA3,RANGRF,CDH19,S1PR3,PRRT2,TRPV4,CLDN1,KIRREL3,PHACTR1,SYPL2,DPYSL3,KCNK2,GNG13,LAMA1,DBH,AMTN,NRXN3,ANO7,KCNIP2,ATP1A3,ELL3,KCNC3,GJB3,SRCIN1,WNK4,ATP6V1G2,FLRT1,CDK5R2,PLCXD3,GRIN2A,PCDHB2,PCDHB6,GRIN3B,ADGRB3,RAPSN,AMPH,KCND1,THY1,NPFF*
GO:0005654	nucleoplasm	0.000028980563625576 8	*FUS,RPS16,RPS27,RPS3A,ANP32B,SRRM2,HNRNPH3,SH3BGRL3,FOSL1,UTP4,HNRNPA3,PRKACA,SF3B4,USB1,BRD4,ATXN2L,SF1,RPS28,HNRNPD,TAF15,PRPF19,DDX39B,CCDC86,ATN1,PTPN23,HNRNPA2B1,CIZ1,CHERP,POLR2A,KHSRP,PSMD2,AKT1S1,NUDC,SLC9A1,PPARD,NXF1,CMIP,TRMT1,PCBP1,MDC1,WIZ,BYSL,NOB1,PDLIM7,PPP1R13L,PCGF2,CHAMP1,CRTC2,PSMC1,TONSL,PDCL3,ZRANB2,SRRM1,BCL9L,SF3A2,NCOR2,UPF3B,POM121C,NFIX,MED15,NFIC,NCOA6,XAB2,PDE4A,GAR1,HCFC1R1,ZMIZ2,PCIF1,CDKN1A,TP53INP2,PRCC,MCOLN1,SKI,CXXC1,ZNF521,SNRPA1,SCAF4,SP2,PLEKHA7,CENPT,DUSP7,UPF3A,RPS26,CRTC1,TAF1C,ATF5,RERE,GATAD2B,BCORL1,MBD6,DMAP1,MEF2D,HMOX1,SETD1A,AFF3,EGR1,FAM98B,NGFR,SYVN1,NSRP1,TENM1,PRR14,POM121,LSM10,DTNA,MRPL2,ZNF580,ESRP2,GATA6,CREB3L1,STXBP1,SETD1B,PLK3,CCNK,FAM222B,ZNF385A,MLLT11,MRNIP,SPHK1,ZBTB12,SOX12,ID4,MNT,MYCT1,GLIS2,ZNF589,HSF4,KDM6B,FABP5,LDOC1,PAX8,MAGEC2,TFAP2A,GPRC5B,NR1D1,SP140,S1PR1,HSPB8,LTB4R2,ARID3B,NEAT1,HMGN5,RNF208,GATA3,GATA2,IKZF4,GEMIN7,SUPT4H1,TTC4,RPP21,RFXAP,APBB3,GCKR,PAK6,PRRX1,ARL2BP,U2AF1L4,POU3F2,LIN37,FOSB,RANGRF,RNASEH2A,SETBP1,OIP5,ANKLE1,MYG1,PTHLH,FOXA3,OLR1,ELL3,CHD5,HOXD1,NELFCD,EGR4,STAG3,UCHL1,IL1RN,FOXO6,GTF2H4,DUSP26,HOXB8,EPHA6,DLX4,PLAGL1,MIF,SOX7*
GO:0045202	synapse	0.000031198682273535 264	*FUS,RPS16,EIF5B,RPS27,RPS3A,ARHGDIA,RPL21,RPL7,FOSL1,PRKACA,RPS28,HNRNPD,USP14,RAB11B,RAB7A,SLC29A1,DLGAP4,PCBP1,DTNBP1,MAL2,V*

			AC14,STX16,VAMP2,GSK3A,RPS26,CC2D1A,GABARAP,NGFR,DTNA,SCAMP5,STXBP1,CAMK2N1,DMD,NLGN2,CHRDL1,PORCN,PRAF2,RAB13,DAPK1,SPHK1,SYNGAP1,SLC9A5,GABRE,FABP5,CACNG7,NR1D1,NPTXR,ARC,PDE2A,SYT11,S1PR1,ATP6V0C,CNKSR2,KCNK1,CORO1A,SLITRK1,PRKCG,RAB3A,PAK6,PRRT1,SORCS3,ASIC1,GABRA3,S1PR3,PRRT2,KIRREL3,PHACTR1,SYPL2,DPYSL3,KCNK2,GNG13,DBH,NRXN3,KCNIP2,ATP1A3,KCNC3,SRCIN1,ATP6V1G2,CDK5R2,PLCXD3,GRIN2A,PCDHB2,PCDHB6,GRIN3B,ADGRB3,RAPSN,AMPH,KCND1,THY1,NPFF
GO:1902495	transmembrane transporter complex	0.000178720012154267 9	*MT-CO2,MT-CYB,MT-ND2,MT-ND5,MT-CO1,MT-ND3,MT-ND4,MT-ND1,MT-ATP6,MT-ND4L,MT-ATP8,PRKACA,MT-CO3,FKBP1A,SLC9A1,VAMP2,PORCN,COX7B2,GABRE,CACNG7,ATP6V0C,SCNN1D,KCNK1,RNASEK,GABRA3,KCNK2,KCNIP2,ATP1A3,KCNC3,KCNAB3,ATP6V1G2,GRIN2A,HCN2,TMEM249,KCNE5,GRIN3B,KCND1*
GO:1990351	transporter complex	0.000673461180147552	*MT-CO2,MT-CYB,MT-ND2,MT-ND5,MT-CO1,MT-ND3,MT-ND4,MT-ND1,MT-ATP6,MT-ND4L,MT-ATP8,PRKACA,MT-CO3,FKBP1A,SLC9A1,VAMP2,PORCN,COX7B2,GABRE,CACNG7,ATP6V0C,SCNN1D,KCNK1,RNASEK,GABRA3,KCNK2,KCNIP2,ATP1A3,KCNC3,KCNAB3,ATP6V1G2,GRIN2A,HCN2,TMEM249,KCNE5,GRIN3B,KCND1*
GO:0016604	nuclear body	0.002793310539117233 3	*SRRM2,SH3BGRL3,PRKACA,ATXN2L,SF1,PRPF19,DDX39B,PTPN23,HNRNPA2B1,NXF1,PCBP1,MDC1,PCGF2,CHAMP1,TONSL,SRRM1,SF3A2,NCOR2,GAR1,CDKN1A,TP53INP2,PRCC,SKI,CXXC1,SNRPA1,CENPT,CRTC1,RERE,GATAD2B,SETD1A,AFF3,NSRP1,TENM1,LSM10,SETD1B,MYCT1,GLIS2,HSF4,NR1D1,SP140,NEAT1,IKZF4,GEMIN7,RFXAP,APBB3,U2AF1L4,SETBP1,OIP5,ELL3,CHD5,GTF2H4,PLAGL1*
GO:0098803	respiratory chain complex	0.005129336052556453	*MT-CO2,MT-CYB,MT-ND2,MT-ND5,MT-CO1,MT-ND3,MT-ND4,MT-ND1,MT-ATP6,MT-ND4L,MT-ATP8,MT-CO3,COX7B2*
GO:0099572	postsynaptic specialization	0.005205371857191358	*RPS27,RPL7,HNRNPD,DLGAP4,PCBP1,DTNBP1,NGFR,CAMK2N1,DMD,NLGN2,DAPK1,SYNGAP1,FABP5,CACNG7,ARC,SYT11,CNKSR2,SLITRK1,PRKCG,PAK6,PRRT1,SORCS3,ASIC1,PRRT2,SRCIN1,GRIN2A,GRIN3B,ADGRB3,RAPSN*
GO:0005737	cytoplasm	0.006606697696080317 4	*FUS,RPS16,CCN1,EIF5B,EIF1,RPS27,MYADM,MT-CO2,MT-CYB,MT-ND2,MT-ND5,MT-CO1,MT-ND3,MT-ND4,MT-ND1,MT-ATP6,MT-ND4L,RPS3A,FTH1,MT-ND6,ANP32B,PABPC1,ARHGDIA,SH3BGRL3,RPL21,RIPOR1,MT-ATP8,SRM,RPL7,ATIC,MT2A,PRKACA,MAT2A,RPL9,RPL18A,CTDNEP1,S100A11,MT-CO3,CCN3,ATXN2L,RPS28,HNRNPD,FKBP1A,DIAPH1,TAF15,PRPF19,DDX39B,USP14,SMG7,PXN,ATN1,PTPN23,HNRNPA2B1,MAP1LC3B,CCDC69,RAB11B,AGPAT1,TYMS,TECR,FST,RAB7A,CHERP,ADM,KHSRP,PSMD2,AKT1S1,EHD2,NUDC,CD44,ODC1,G6PC3,SLC9A1,PPP1R11,ECM1,BRPF3,NXF1,CMIP,ZFP36L1,TRMT1,CCDC124,PCBP1,CDA,PPDPF,ALG3,ZYX,MAPK13,BYSL,NOB1,PDLIM7,PPP1R13L,NT5DC2,CHAMP1,CRTC2,PACS1,DTNBP1,PSMC1,RPL17,MAL2,TONSL,PDCL3,SRRM1,VAC14,SAMD4B,DVL3,ADAMTS1,NPRL3,SLAMF7,BAK1,UPF3B,POM121C,TRAPPC1,STX16,MED15,NCOA6,EPS15L1,PDE4A,VAMP2,HCFC1R1,ZMIZ2,CDKN1A,TP53INP2,EDC3,MYD88,MCOLN1,SKI,ACTMAP,MAP2K7,CXXC1,PDE1C,GSK3A,PRSS3,TINAGL1,PLEKHA7,CENPT,DUSP7,CXCR4,UPF3A,UCP2,EI*

			F2B4,CNOT3,TPP1,RPS26,CRTC1,CC2D1A,ATF5,SORBS3,CDK11B,PHGDH,NRGN,PHACTR4,ALDOA,DGAT1,DMAP1,PLBD2,PABPC1L,BET1L,MEF2D,HMOX1,SETD1A,AFF3,EGR1,LRRC61,FAM98B,COL1A1,GABARAP,NGFR,FHL3,SYVN1,TENM1,HEBP1,MARCHF4,POM121,GPR137,ST3GAL6,LSM10,DTNA,MRPL2,SERPINB8,CREB3L1,SCAMP5,STXBP1,EIF3C,DMD,SETD1B,PLK3,RELN,ENO3,SLC38A7,ABTB1,FURIN,FAM222B,ALS2CL,EIF5AL1,GSTM1,CHRDL1,SLC39A4,DOK3,CTH,GPR3,RTKN,EVA1A,LIMS2,NAA80,DOCK6,PORCN,ZNF385A,ZBED3,PRAF2,RAB13,KIFC2,TOMM5,ZFTRAF1,MLLT11,DAPK1,SPHK1,RASEF,PLEKHN1,TAX1BP3,SYNGAP1,SLC29A3,ID4,ZNF703,RIN3,TUBG2,RGS4,DIPK1B,ILK,NAPRT,ARL10,FZD8,KRTAP2-3,GLIS2,SLC9A5,TUBB3,COX7B2,NRK,FABP5,ARHGEF19,MAGEC2,GPRC5B,CACNG7,ISYNA1,ANKRD13B,NR1D1,ARC,PDE2A,ZFP36,CYP1A1,SYT11,MUC20-OT1,SP140,TNFSF12,PDGFD,ARSA,S1PR1,HSPB8,SLC25A35,FNDC4,ATP6V0C,HMGN5,RNF208,GATA2,MRPS24,LOXL3,GEMIN7,CNKSR2,MROH6,MYO7B,CCDC120,KCNK1,NCF2,DHPS,TTC4,GSTT2B,EDN1,APBB3,IGF2,CDK11A,MMP2,MC1R,GALNT14,CORO1A,DDT,PRKCG,GCKR,RAB3A,CCDC78,SERPINB2,PAK6,PRRT1,RNASEK,KRTCAP2,PRRX1,ARL2BP,MTFP1,ANXA8L1,SDR39U1,U2AF1L4,SEC31B,ASIC1,SERPINE1,SLC52A3,FAM83A,FOSB,PARP15,AOC2,RNF39,CST1,ACTRT3,RANGRF,WASHC1,MMP3,ACTA2,PTPN22,ABLIM2,COL1A2,FBXL16,CST4,FKBP1C,NPL,S1PR3,PRRT2,TRPV4,CYP2J2,KIRREL3,BCL2L2-PABPN1,PHACTR1,SYPL2,ENSG00000286001,ASB16,ANPEP,RAB3IL1,GPR15,DPYSL3,KCNK2,SNX10,RNASEH2A,SETBP1,CAPN11,ESPN,ANKLE1,C10ORF67,GNMT,MYG1,VWCE,PKDCC,PTHLH,DBH,TMEM198,CD177,AOC3,ANGPTL6,SCO2,OLR1,AMTN,SYT15,MAP1LC3B2,ANO7,BCHE,EPB41L4A,KCNIP2,ATP1A3,TNFRSF25,ELL3,CHD5,TPH1,NUSAP1,CMC4,KCNC3,CRLF1,GJB3,RCAN2,NELL2,SRCIN1,COL3A1,TMED6,SAMSN1,WNK4,ARHGEF16,UCHL1,IL1RN,SFRP1,KCNAB3,ATP6V1G2,SERPINB7,DCAF8L2,EBI3,ARHGAP20,PANX2,SPNS1,FLRT1,UCP3,PCSK1N,CNBD2,HAL,CDK5R2,KRT14,AATK,FOXO6,PLCXD3,GRIN2A,HSD11B2,HLA-DOB,CGB7,CNFN,SERPINB5,SULT1B1,GOLGA8A,CCDC154,DUSP26,CPT1B,ADH6,BCKDHA,MAOB,GAL3ST2,TMEM190,CALML6,TMEM119,TMLHE,GDPD3,RAPSN,KIAA0319,AMPH,TMED7-TICAM2,TCHH,RBM20,BGN,ADAMTS5,PLAGL1,MIF,ALOXE3,FNDC5,THY1,CGB8,SLC25A34,SOX7
GO:0098793	presynapse	0.006667546147510328	*FUS,FOSL1,USP14,RAB11B,RAB7A,SLC29A1,DTNBP1,MAL2,VAC14,STX16,VAMP2,NGFR,SCAMP5,STXBP1,NLGN2,PRAF2,RAB13,SPHK1,PDE2A,SYT11,S1PR1,ATP6V0C,PRKCG,RAB3A,PRRT1,ASIC1,S1PR3,PRRT2,KIRREL3,SYPL2,KCNK2,NRXN3,KCNC3,SRCIN1,ATP6V1G2,CDK5R2,GRIN2A,AMPH,THY1,NPFF*
GO:0031252	cell leading edge	0.01118282864808934	*MYADM,PABPC1,SH3BGRL3,RIPOR1,S100A11,DIAPH1,PXN,CD44,SLC9A1,PDLIM7,PDE4A,CXCR4,PHACTR4,PEAR1,RAB13,ILK,SLC9A5,TUBB3,GABRE,PSD4,CORO1A,GABRA3,ACTA2,TRPV4,DPYSL3,CD177,KCNC3,SAMSN1,AMPH,THY1*
GO:0070382	exocytic vesicle	0.013525245685706351	*RAB11B,RAB7A,DTNBP1,MAL2,STX16,VAMP2,SCAMP5,RAB13,SYT11,ATP6V0C,RAB3A,PRRT1,PRRT2,KIRREL3,SYPL2,RAB3IL1,DPYSL3,SYT15,ATP6V1G2,GRIN2A,AMPH*

GO:0098984	neuron to neuron synapse	0.026154216123684407	*RPS27,RPL7,HNRNPD,PCBP1,DTNBP1,MAL2,NGFR,CAMK2N1,NLGN2,DAPK1,SYNGAP1,FABP5,CACNG7,ARC,SYT11,CNKSR2,SLITRK1,PRKCG,PAK6,PRRT1,SORCS3,ASIC1,PRRT2,ATP1A3,SRCIN1,GRIN2A,GRIN3B,ADGRB3*
GO:0005615	extracellular space	0.04093389500793416	*RPS16,CCN1,RPS3A,FTH1,ANP32B,PABPC1,ARHGDIA,SH3BGRL3,RIPOR1,SERINC2,ATIC,PRKACA,S100A11,CCN3,RPS28,USP14,PTPN23,HNRNPA2B1,RAB11B,FST,RAB7A,ADM,PSMD2,EHD2,CD44,SLC9A1,ECM1,PCBP1,WIZ,LOX,C4BPB,CRTC2,MAL2,ZNF114,GPX3,GNG5,RHOJ,PRSS3,TINAGL1,PLEKHA7,CXCR4,TPP1,RPS26,CC2D1A,PHGDH,ALDOA,PLBD2,CCBE1,PABPC1L,HMOX1,COL1A1,HEBP1,ST3GAL6,SERPINB8,STXBP1,RELN,MMP1,ENO3,FURIN,CTH,ZBED3,RAB13,TAX1BP3,NAPRT,TUBB3,FABP5,TNFSF15,GPRC5B,C5ORF46,BMPER,TNFSF12,PDGFD,ARSA,FNDC4,ATP6V0C,LOXL3,CNKSR2,CTF1,GSTT2B,EDN1,AMH,IGF2,MMP2,CORO1A,DDT,SERPINB2,LCAT,SERPINE1,CST1,MMP3,ACTA2,LRRC15,COL1A2,CST4,CYSRT1,BMP6,CYP2J2,ANPEP,PCOLCE,DPYSL3,LAMA1,CILP2,PTHLH,DBH,CD177,GREM1,ANGPTL6,LYPD3,BCHE,CRLF1,NELL2,VSTM1,COL3A1,STAG3,IL1RN,SFRP1,GNRH2,SERPINB7,EBI3,FLRT1,PCSK1N,KRT14,CGB7,SERPINB5,DUSP26,ADH6,MXRA5,GDPD3,BGN,ADAMTS5,SPINK1,MIF,GKN2,GPRC5D,THY1,NPFF,CGB8*
GO:0014069	postsynaptic density	0.04245828722464248	*RPS27,RPL7,HNRNPD,PCBP1,DTNBP1,NGFR,CAMK2N1,DAPK1,SYNGAP1,FABP5,CACNG7,ARC,SYT11,CNKSR2,SLITRK1,PRKCG,PAK6,PRRT1,SORCS3,ASIC1,PRRT2,SRCIN1,GRIN2A,GRIN3B,ADGRB3*

Table S4. The Venn diagram shows the intersections of downregulated genes detected in MB and Mel Z cells growing on 3D matrix. Related to the Figure 2.

Names	total	elements
down MBA-MBA down Mel Z	98	*ENSG00000286540 ENSG00000288993 ENSG00000286482 TNS3 TRIM2 SLC13A4 ENSG00000287609 ZBTB20 TARID KIF14 MEGF10 LINC00601 C5 SLC2A10 BEX2 PCDHGA5 HEXIM2-AS1 ESR1 PRECSIT GNG2 ALPK1 PCDHGA4 NNMT VASH2 PCDHGB7 PLCB4 SPRY1 ZMAT1 LINC00589 CRACD CAPS2 LINC00243 PTPN6 NKPD1 PCDHGA11 MX1 SRP14-DT PTPRJ DLC1 SCARNA7 PCDHGB3 NEURL1B ST6GAL1 CKMT2 JDP2 ETV1 ERBB4 SYNE2 FAM20A CDK15 PIGAP1 ARHGAP30 ZNF704 CCNB3 ALDH1A2 MIR3681HG MAP2K6 PDE9A ST8SIA1 HOGA1 BEST1 ZNF546 ADAMTS20 CLMN CASC2 RN7SL3 SLITRK6 ALDH1A3-AS1 RAPGEF4 HLA-F-AS1 ARSI ZNF610 PCDHGA2 TMEM150C IKZF2 YPEL4 ASPM DDIT4 LAMB1 RETREG1-AS1 EXPH5 L3MBTL2-AS1 NR5A2 RBM47 SEMA3D SRGAP3 LINC02882 SLFN5 PLAC4 LINC01234 PRICKLE1 HIP1 NECTIN3-AS1 PCDHGA6 AR ARHGEF10 P3H3 FRMD4B*
down MBA-MBA	1413	*AL138820.1 C10orf90 RN7SKP172 KCNMB2-AS1 ABCC6 H3C12 RMRP CRTAM LOC105373335 AC025031.5 AC058791.1 RNU4-2 TMEM139 EEF1A1P10 NTSR1 COL4A5 G2E3 RN7SL143P TSEN15P1 CRACDL ABALON EEF1A1P14 AC005070.3 AC005831.1 SLC16A14 SLC4A8 RN7SKP148 PLCG1-AS1 RNU5F-1 AK7 HLA-DRB5 SPN FER1L5 LRRN1 RN7SL504P HOXA13 AC115618.1 SORL1 RN7SL664P DMC1 RN7SKP291 SUGCT SNORD3B-2 GPR153 IRX5 SNORA80D ENSG00000290524 SCAT1 AL356273.3 RN7SL308P AL356968.2 ENSG00000290399 FKBP14-AS1 LINC02487 Metazoa_SRP ALDOC TAS2R30 AC112721.1 SLC1A7 AC015849.4 GPR55 LINC03040 ENSG00000289259 Z93241.1 GAPDHP55 TNFRSF9 RN7SKP227 ENSG00000290003 ISM1 LINC02869 AL139241.1 ENSG00000289283 VAT1L GYPC ST8SIA4 RN7SKP44 H2BC21 C3 RN7SL508P SLCO2A1 ADGRF1 PRDM16 SNORD67 LAMP3 FAM182B AC069542.1 AC133644.2 SNORA2A PCDH7 MMP16 RN7SL836P SNORA19*

		MIR3667HG COL18A1 PPP1R9A C1QL1 H2AC8 LINC02939 HLA-DRA RNU2-63P NPR1 TMEM225B SLC9A9 DNAH12 RCSD1 AC008700.1 AC112907.3 AL157938.2 RN7SL144P ZNF334 PAQR8 GMFG PIK3AP1 CHST6 LRRK2 LOC105374101 AC131571.1 PCDHGA1 AC012625.1 SNORD46 CALHM5 RN7SL452P Z68871.1 ENSG00000290074 SULT1C2 FCGBP CRPPA GCA QRICH1 FIRRE H2BC11 AP001429.1 RN7SL424P ALDH1L2 RN7SKP137 GPRC5C RN7SL803P AC018628.1 KCNJ13 FOCAD TIAM1 AC108463.2 RN7SL505P FBXO36 PBX1 AHR RPS2P47 LOC100131496 AL132642.1 EEF1A1P29 AL021578.1 SORCS2 H3C1 ZNF805 MFSD1P1 SNORA54 ENSG00000286997 ENSG00000289866 RN7SL646P AL033530.1 ENSG00000289447 AC110769.2 SOWAHB AC132219.2 MIR29B2CHG SNORA49 SCN3A PPL C1QTNF7-AS1 MSNP1 H2BC18 AHRR RF00493 AC132192.1 CYP19A1 RN7SKP224 SNORA74A MEIS3P2 PCDHGB4 AL021155.2 RN7SKP68 CEBPD ADRB1 FHDC1 GPR158 ALDH6A1 MUC5B OMD H2AC21 SVIL SVEP1 ENSG00000288997 APBA2 DIXDC1 PCBP3 CADPS2 CASC19 SPOUT1 GLDN EGF VSIG1 IL1A LINC00639 SNORA38B Z69733.1 RN7SKP255 RN7SL752P TRIL LOC124902027 RELL1 ABCC12 AL391421.1 AC005906.3 ZNF254 SNORD11 RN7SKP115 SNX13 ANKRD22 HLA-DOA AC009303.2 RN7SKP213 PCDHGA10 ZNF518B SHH LINC00239 CNTNAP3B GAS5-AS1 ENSG00000286786 MAP7 H2AC17 DDN-AS1 KLRK1-AS1 RNU6-2 ENSG00000287735 AC084262.2 SHISAL1 FLJ13224 RMEL3 AC006206.2 LRP1B XAF1 LINC02506 NAV2-AS2 PLAC9 USH2A LINC02331 LRP5 RN7SL230P GLIPR2 H2AC6 AL162274.1 LOC254896 H2AC13 ENSG00000286742 AC005180.2 IL20RA CHN2 CLIP2 AC138028.2 RN7SL394P RN7SL181P KRT83 AC097626.1 RASSF9 LINC02739 RN7SKP230 LGALS12 LRRC19 SLCO1B1 AC103591.3 AC026124.1 AL133230.1 H2AC1 CMKLR1 AL359094.1 LNROP AC005261.5 DENND2A GPRIN2 H4C16 RPL41P2 MAGEA3 CLIC5 EPSTI1 LRIG1 ITGB8 LINC00881 ENSG00000289121 TNFRSF10C PDE3A FLJ42393 RNY3 AC104964.2 SLC19A1 ENSG00000287774 ZBTB41 AC009486.1 ARRDC3-AS1 EEF1A1P50 RN7SKP125 ZBED6 AC243965.2 SLC37A1 TNFRSF8 BCL11B WDR38 AC144450.1 RNU5D-1 RN7SKP8 RN7SKP166 COCH ENSG00000288899 AC127024.8 LFNG RN7SKP80 AC103740.2 ZC3H11B MIR6077 RN7SL200P KIF5C LOC124906232 PDK4 GBP4 AC080038.3 GPM6A CSAG1 APOM CA12 RN7SL444P RN7SL411P AL445472.1 SNORA79 HNRNPA1P49 COLEC12 CHCHD3P3 KRT86 IRF2BPL ATP2A3 RPS6KA2 SH3PXD2A-AS1 AC092828.1 FGFBP1 CYTH4 PLXNA4 RN7SL678P PTPRC GRAMD1C ADGRF5 B3GALT2 AC138409.1 RN7SKP160 ITGA10 ABCC9 ZNF727 MCF2L FAM87A AC245014.3 AP002762.1 AC131902.1 KCNH3 ASB4 SNORA23 C1S CGNL1 FOXP2 SNORA74D RN7SL38P AC141930.1 AC092171.1 ENSG00000288744 F13A1 SLC2A14 SLC10A1 DNAH7 OGN AC026124.2 DSCAM-AS1 KIF18B-DT LINC01182 LINC01393 RNU1-88P RN7SL277P SH3TC2 PXMP4 TIPARP-AS1 PRSS2 FAM171B CNTNAP3P2 PGF ATP9A F11-AS1 SCARNA18 LIFR-AS1 AL162724.1 BTBD10P2 ALOX5AP ZNF430 C19orf18 AC107027.3 GNAZ ENSG00000287453 MDM2 AC091868.2 LINC01730 AC099513.1 LVRN ITGBL1 NDST3 OVCH1-AS1 EEF1A1P22 GALNT6 LINC01473 ADAM28 RN7SL515P MYLK B4GALNT3 SAP30BP-AS1 LOC124903002 CHI3L2 ANKRD36C TTC39A RN7SL242P ENSG00000288971 AC008894.2 UQCC3 AC002074.1 RN7SL381P RN7SL498P RNU1-2 RPS7P11 CELF2-AS2 ENSG00000287302 TMPRSS3 LRRTM2 RN7SKP281 SLC37A2 RN7SKP93 PDGFA AC092139.2 ERP27 TRHR RPSAP18 RNU6ATAC SNORD6 MPRIP-AS1 LOC124902301 RN7SL648P STK31 DIAPH2-AS1 ENSG00000290091 HLA-DPB1 TTC39B RN7SL263P ENSG00000287190 C2orf72 SCARNA1 PSMC2P1 HELB PHACTR2 LIPG CD163L1 RN7SL709P RN7SKP174 APOLD1 RN7SKP71 PDE5A NOX5 EEF1A1P1 H3C2 BCAR3-AS1 RN7SL300P KLB LOC100419503 RN7SL643P SMIM31 SNORA3B AC016727.3 EEF1A1P3 HYAL4 HLA-G RN7SL334P ENSG00000293592 UNC13A ENSG00000286797 SEMA6D RN7SL219P SNORD89 ZNF573 C2 PCDHGA8 NTF3 RBMS3-AS2 AL591866.1 ENSG00000289210 MACC1 LINC00865 ENSG00000289872 MCMDC2 AC084262.1 BTBD18 ZBTB10 RNU4-1 AC068790.2 H2AC11 SNORA63D AC006064.2 RN7SL482P ENSG00000289044 AC078923.1 AC051619.8 AL031009.1 PDE10A MAGI1-AS1 FSIP2 AC090791.1 RN7SKP292 GNG7 AP000560.1 AC007823.1 RN7SL302P SNX22 ENSG00000287978

		LOC124902629 TFPI QRSL1P3 FABP6 RN7SKP22 AC109326.1 TNFRSF18 CXCL10 TRPM3 GPR85 ENSG00000289280 ASB14 HAND2-AS1 RN7SL494P ADAMTS3 RPL7P23 SNORD3C PCDHB12 SNORD82 CCL5 SNORA74C-2 SNORA80A TAS2R13 PRDM10-DT RNVU1-29 FGF13 RN7SL712P RN7SKP48 SPANXB1 ILDR2 ENSG00000290040 AC020687.1 IMMP2L DNAH3 AL353748.2 AC116345.1 SSPN IGSF9B LYZ RN7SL5P RN7SL325P FAT1 ZNF660 H2AC12 RN7SL124P LINC00456 RPL21P83 ENSG00000289341 MIRLET7A2 ALG1L5P LCP1 RN7SKP203 SNORD94 PCDHGB6 GUSBP9 RASSF8 MAGEA12 RN7SL825P RNU1-1 RAB39A ZNF737 AC110058.1 IQCJ-SCHIP1-AS1 VN1R83P FZD3 IFIH1 RN7SL127P ENSG00000288755 BCL2L1-AS1 ATRNL1 TMEM232 RNU5A-8P TMEM135 LINC01962 IL1B PREX1 RIMKLA CPE DCHS1 LINC02453 NSUN7 CFAP300 SHROOM3-AS1 AC145207.5 UST AC073569.1 AC006330.1 AC068580.2 H3C3 KLHL11 SPANXC ST3GAL5 NDUFV2P1 AL390318.1 RUNX1-AS1 LOC100421558 SNORD73B SNORD105B AP003555.2 AL355312.1 LINC01204 AL022323.2 N4BP2L1 ZNF91 SEMA6B LIN7C LONRF1 SNORA71B FUT2 ANKRD7 FAM20C PDK3 AP000864.1 MAGED1 SNORA73B AL031733.2 SPTSSB LOC388282 TCN2 H2BC3 AC022395.1 BMP5 NDP ENSG00000291338 ENSG00000289267 CRYBG1 CCDC148 ENSG00000290035 H2AC5P AF131215.4 SERPINA5 FBXL13 KCNJ8 H4C1 CPNE7 FKBP9P1 EVL PLAAT5 RN7SKP180 INSIG1 ARSJ CFD TEX15 PYCARD PARD6B ENSG00000288012 SOX2 FLVCR2 LINC02937 H1-4 EEF1A1P17 LOC124901856 C1QTNF1 COLQ RN7SKP124 H1-2 ABCA13 AGR2 AC092757.3 RN7SKP79 BEAN1 RDM1 RN7SL477P SNORA14B ENSG00000289602 AC134349.1 RN7SL610P RN7SL130P AC092645.1 RPSAP53 RN7SL398P UGCG TSPAN11 ATRX ADRA2C AC007684.1 STK24-AS1 MPZL2 MDFIC C1R RFX3-DT DENND11 PPIAP42 S100P RN7SKP36 RN7SL635P ACOT1 LGR6 SCARNA23 RN7SL97P HMGB3 HLTF PADI3 RN7SKP127 RNU6-8 RO60 MIR181A1HG AC051619.7 ENSG00000287169 RNVU1-8 IRF2BP2 NUP210L SOX4 PLAAT4 LOC105375170 LOC105373682 NRP1 DOCK4-AS1 RN7SL564P GPR162 ZFP3 TSC22D1-AS1 TRPM8 H2AC14 RN7SL735P AC005180.1 ENSG00000292256 DIAPH2 H3C10 GPX7 SVIL2P PNPLA4 MAP3K5 H4C8 HMGB1P14 KAZN-AS1 DNAJC22 CA14 CLDN11 AC100814.1 SNORA5A H2BC13 RN7SL118P ABCG1 7SK DANT2 SCARNA9 SNORD3A GLUD2 RN7SL146P ENSG00000290076 PLN ZBTB47-AS1 RN7SL322P PLD5P1 RAC1P7 LNPEP CARD11 SHISA3 APOL6 GASK1A LAIR2 SLC38A2-AS1 SYT2 H1-6 ITGB4 RNVU1-31 LOC100506071 ENSG00000287352 MEIS3P1 LOC731157 PRKCA-AS1 TBC1D5 ENSG00000289396 RN7SL169P LGALS8-AS1 RAB37 LINC01504 EEF1A1P4 AC079305.3 AC006141.1 SEPTIN14P12 RN7SL833P RN7SL778P ABCA1 SEMA3E TNFSF10 PCDHGA7 SLC40A1 PCDHB8 AC003681.1 MAEL CPLX1 RN7SKP61 CCSER1 RN7SL396P EEF1A1P9 ELF3 COPZ2 RN7SL732P LOXL2-AS1 RN7SKP184 STMN3 EEF1A1P30 ENSG00000287932 AL603756.1 GRK3 PLXNB1 MIR4521 EMBP1 AP000577.1 RNVU1-3 TCEAL8 RN7SL838P GIHCG LOC101928053 GSAP RN7SL8P ZNF382 AL442663.1 RN7SL116P ITGA1 H2BC5 TENM2 ENSG00000291189 DAZAP2P1 Z95331.1 RN7SL726P CENPN-AS1 COLGALT2 RN7SL801P SLC47A1 PCDHB11 GRIK4 CXCL11 DLL1 RN7SL507P SERPINA1 CACNG4 RN7SKP76 ENSG00000289507 TTBK2 LINC02972 ENSG00000286092 AP001350.1 SCRG1 MYO16 CXCL8 NOTCH2NLB PARP14 RN7SL178P VTRNA2-1 RN7SKP102 MSX2 PROKR1 KLRC2 DLG3 FAR2 ENSG00000286360 LINC01465 RN7SL15P BX323046.1 FAM169A TGM5 ABCD2 C11orf86 LEFTY1 ENSG00000289851 LOC339059 ENSG00000287697 ARHGAP28 MIR378D2HG ZNF92 RN7SL660P LEPR RN7SL278P PRH2 RNU2-64P NDUFA4L2 SLCO1B7 RNU1-67P RN7SL107P LOC100289495 STEAP4 EYA1 PITX2 RORB AP003049.2 SLC9A7 POU5F2 EIF2S2P3 RNY1 BLID P2RY1 C2CD4C RNVU1-4 H1-5 SNAP25 CTSS ZXDA COL6A2 ANO1 SPOCK3 ENSG00000289123 HLA-DPA1 CLDN23 GLB1L2 RN7SKP62 H4C2 ANK2 RN7SL447P PRC1-AS1 MIR100HG DCDC2B ITGB6 PDGFB LTB HLA-DMB CSF3 LZTS1 AC005183.1 DDX60 HSD3B1 AC004466.2 GRIN2B ADAMTS14 HR RNVU1-30 SNORA53 AC009318.3 SYNJ2 LINC01091 RN7SL702P AC005480.1 AL035071.2 TBXAS1 GPR176 SNORA14A RN7SL748P RN7SL792P RN7SL674P HMGA2-AS1 ENSG00000290879 AC110792.2 SPRED1 NPR3 TMEM132D-AS1 IRX1 LOC105377862 TSPAN33 PPP2R2C RN7SL408P RGCC TM4SF18 GALNT12

		RPS4XP22 VPS37D RN7SL122P SYNM-AS2 DAPK2 SPANXD PCDHGA9 PRPF31-AS1 RN7SL521P ADAMTSL2 AC145676.1 MYL1 LINC01844 TNFSF18 TRIM61 GTF2I-AS1 KLHL4 ENSG00000287134 DENND5B RN7SKP90 H2BC14 AC024909.2 RNY3P1 RN7SL516P RN7SL769P SCARNA6 RNU4ATAC ZNF630 CAP1P1 AC025569.1 AC234772.2 SNORA47 SNORA38 JCAD ENSG00000290121 PCDHGA12 CDKL2 RN7SKP130 RN7SL749P ABHD17AP3 DANT1 ITPR2 ANXA2R-AS1 ENSG00000286473 SH3TC1 LINC01303 MEIS3 ENSG00000286242 MUC4 SLC35F3 LINC02104 EEF1A1P8 INHBB IER3-AS1 ZG16B MGMT TRBC2 DHRS3 BHLHA15 TUBAP2 MIR3609 RPL21P89 ENSG00000286313 AC108134.2 RN7SKP269 AC087521.2 OASL KRT81 RN7SKP119 RNVU1-28 RAP2B LINC01623 TMPRSS2 IL6 TLR3 SNAI3 FGF14 CCL24 ENSG00000289437 CSRNP3 RN7SL125P H2AC4 LRRC37A15P H4C11 SNORD12C AC135279.3 RN7SKP178 AL606763.1 H2AC15 SNORA22C RPL7AP11 MVB12B MX2 TMEM14EP ZP3 NUP210 LOC124904917 ANGPT2 THSD7B HLA-DRB6 AC007952.4 U3 RN7SL634P SCARNA3 NPM1P26 RN7SL70P AC008739.5 GIMAP2 RN7SL555P RGS5 ENSG00000289482 ENSG00000289634 AC084082.1 EPPK1 MOXD1 CARS1-AS1 AC110285.7 KCNT2 LOC128125822 TMEM45B RN7SL587P AC007878.1 AL390195.2 ECM2 PECAM1 ENSG00000286835 EEF1A1P24 RN7SL481P TPRG1 RN7SKP23 RNVU1-34 RN7SKP173 STXBP5L PLCG2 RNU5A-1 SCARA5 SNORA22 ENSG00000289357 CCR1 PTPRN2 UBE2D3P1 RN7SL43P ENSG00000288955 SNORD13 TNFSF4 ENSG00000288900 ENSG00000288865 RALGAPA1P1 H3C13 BTC MGAT2 TCN1 RN7SKP163 EEF1A1P47 RN7SL359P RN7SL364P ANKRD19P LINC00513 SLC2A12 GSX2 ADPRH H4C12 KLF8 RN7SL151P ENSG00000287979 PRKG1 P2RY6 AC063960.2 SLC16A6 H2BC1 MIR5087 SNORD15B AC004817.3 PIK3CG AL162171.2 WHSC1L2P KREMEN1 GSPT2 LIFR AC131971.1 AC114760.2 SNORA7B DMBT1 SRRM3 RN7SL663P ENSG00000289347 LINC02983 AL137782.1 SNORD100 RN7SL575P AC001226.2 ALDH3A1 CALHM3 H2BC6 C11orf87 PLBD1-AS1 ZNF766 ACSM4 ENEMAL HELLPAR LINC00654 RAB40A RN7SL126P MRPS35-DT AC130651.1 ERRFI1-DT RNU5E-4P RN7SL260P AL355075.2 SNORA66 AC013451.2 ENSG00000289017 SNORA74B MIR133A1HG PCDHB10 ZNF460 NOTCH2NLA AC084117.1 RN7SL566P RN7SL416P CST6 AKAP9 LINC02873 RN7SL479P RASSF2 AC005884.2 SLC30A10 GJB2 RN7SL30P KLRC3 SRP14P3 RN7SL1 PCDHGB5 TNS4 SCARNA21 LAMA5-AS1 SNORD59A IRX2 CELSR1 ENSG00000291073 AL080276.2 RANBP17 AP001442.1 PRSS23 SNORA80B ISG20 ABCB4 RN7SKP205 PROB1 STEAP2 RNVU1-2 AC097639.1 BICC1 RNVU1-15 SLFN13 ENSG00000289152 RN7SL49P ENSG00000286523 TRAC MMP17 NXF3 GDF5 AC006511.3 AC010320.4 RTP4 RIPOR3 RN7SKP185 ZNF273 RHOA-IT1 RBMXP2 AC007686.4 HSPD1P11 TMEM121 NKX1-2 CADM1 LRRN3 GCNT4 RN7SKP140 LOC124904611 AL162385.2 RN7SKP95 LINC02475 IL7R ENSG00000287167 AL355336.1 LINC01505 LINC00989 RN7SL559P RNU5B-1 SNORA2C EEF1A1P13 PPFIBP2 AC009812.3 VCAM1 RBMS3 EDARADD LINC02915 RN7SKP214 RN7SL4P LOC105374114 THOC7-AS1 SNORA28 ST6GALNAC2 ENSG00000287712 PRADX ZNF525 FLG ZNF680 SNORD17 AC016831.4 HLA-DRB1 RPS4XP11 ENSG00000288806 LINC02454 TRBV13 AC104692.1 JAK3 ENSG00000288924 LINC01714 RPL7AP45 SLC39A11 Z97198.1 NCMAP ENPP1 PPP1R1C ULK2 RADIL CCND2 LINC01338 ENSG00000287643 APOBEC3G RP1L1 EPHB6 DNMBP-AS1 U73166.1 GPRIN3 ENSG00000286341 CXXC4 AC087257.1 FAM114A1 EGLN3 ENSG00000291336 ENSG00000293570 TTC32-DT H4C3 SNORA79B RN7SKP189 MUC5AC AC099343.2 PPP1R3C CT70 ZNF217-AS1 AIM2 TMT1A LOC124904122 AC092053.3 LOC124904332 F2RL3 LCMT1-AS2 PDE8B RN7SKP186 RN7SL166P ZNF714 ENSG00000288025 AC073333.1 RLIM VTRNA1-3 AF131216.1 SUMO4 AC093484.4 SETX RN7SL128P KCTD12 RN7SKP55 RN7SKP268 FZD1 RN7SL210P SAA2 MAGEA6-DT SNORA12 ENSG00000287857 AC021106.3 TRIM59 AC002558.3 AF015262.1 RNVU1-27 GPC6 SNED1 RN7SKP30 SNORA22B ADGRV1 VPS13C XIRP2 AC005921.3 TESC SLC14A1 AC011912.1 B3GALT1 ENSG00000287884 LOC124905233 RNVU1-7 LINC02809 H2BC15 RASSF6 APOL3 AC055811.3 SLCO4C1 KRT8P39 DUSP9 SNAP91 RN7SL665P PPP4R4 TAS2R20 RN7SKP209 ANO6 NEK3 AF121898.1 H2AC20 SCARNA5 RN7SL767P PCDHGB1 GLIPR1L1

		AC008277.1 RN7SKP9 LINC00216 ZNF678 RN7SL105P Z82217.1 VAV1 INSYN2A TP63 ATF7IP2 LANCL1 LOC124900638 NLRP14 PTCH2 RN7SL480P ENSG00000286171 ASB2 LINC02701 ENSG00000288888 RN7SKP170 HCLS1 KLHL24 H2BC17 SPEF2
down Mel Z	1697	*SAMD4A PKNOX2 APBB2 CNTNAP1 ENSG00000268912 MGC16275 TESMIN ENSG00000256433 CMTM8 ENSG00000270116 ENSG00000236013 CSPG4 NHLRC3 FAM214A CYP39A1 GALNT3 LDB3 GP1BA PELI2 SIDT1 CHRNA10 TECTA ENSG00000279407 KIRREL1 ENSG00000233539 ASNSP1 IGF2BP3 TRIB3 DNAI1 ENSG00000239291 ZEB1 CDK14 DNAJC3-DT TBC1D8-AS1 FAT4 LINC01569 HCN1 SYCP3 ENSG00000279278 PDE1C ITLN2 ENSG00000279149 ENSG00000241634 ENSG00000250041 LRRC37A4P KNDC1 MED13L TMEM254-AS1 TAGAP-AS1 NFIA TEAD1 TCEANC2 ENSG00000227741 HEIH ENSG00000280384 ENSG00000269954 ENSG00000276900 INSIG1-DT ENSG00000261770 BRCA1 MCPH1-DT PEF1-AS1 NUDT9P1 BAHCC1 PAPSS2 ACSL3 TRIQK CDC14A UTRN ENSG00000224691 ENSG00000267248 TLR1 ENTPD4-DT ENSG00000258634 TMEM240 LINC01198 ENSG00000279253 GPR37L1 CCDC92B ZFP14 CECR2 CBX3P2 SCX ARHGEF26-AS1 ENSG00000213963 ANKRD6 NEIL1 ENSG00000282885 ENSG00000286990 SCUBE3 ENSG00000263235 RAB30-DT GRASLND ENSG00000271327 ENSG00000279386 ROR2 CORO2A MAOA ENSG00000284471 ENSG00000275582 ENSG00000238018 TMPO-AS1 BTN3A1 ENSG00000287997 ENSG00000280239 LINC01011 ENSG00000259720 ATOH8 ENSG00000272320 ENTPD8 RFX3 ENSG00000273442 PPM1K HMGN1P1 CARMIL1 STARD13 ZNF75A KCNMA1-AS1 HESX1 MYO15B IFT140 IL24 ZNF284 WDFY2 ELAPOR2 ENSG00000283341 C14orf132 TOX3 THRB RALY-AS1 CDC42BPA WDR19 GTF2IP20 PKD1P5 RAG1 IRS1 GPR19 ENSG00000254528 IGSF11 EDRF1-DT RPL13AP12 SENP7 ENSG00000277050 MBD5 JARID2-DT CROCCP3 MAPK10 ENSG00000271992 CACNB1 RPL5P18 ZNF467 HES7 APRG1 GLIS2 ENSG00000223522 ENSG00000215068 POU6F1 ENSG00000280537 ENSG00000289183 CTC-338M12.4 LINC02895 PLD1 MNX1-AS1 CAPN12 AKNA HPS4 RARB ZNF320 KCNQ5 IMPDH1P8 ENSG00000267002 IDI2 IGDCC3 ENSG00000258744 FAM218A ENSG00000232995 CPAMD8 STON1 NOTCH2NLC DENND3-AS1 RTN4RL1 MSANTD2-AS1 TTTY14 LCA5L ENSG00000286158 PTPRH ENSG00000233967 ENSG00000280353 RARA-AS1 SORBS2 PCDHGC5 ACAD11 GGTA2P CHL1-AS1 ARMH1 ENSG00000279989 GNAO1 ENSG00000289132 KRCC1 ENSG00000224950 GDF9 TJP3 ENSG00000268858 TMEM140 ENSG00000280435 ZC3H6 FXYD2 TWF2-DT ASAP2 LINC01786 GABBR1 PSMD6-AS2 DISC1 ENSG00000248161 ENSG00000286830 ST7-AS1 JPX DCX PLSCR4 DUS4L PPARGC1A MIR503HG ENSG00000271734 FMN1 ENSG00000272498 ZMIZ1-AS1 CARD14 KCTD21-AS1 ENSG00000263551 PCAT1 ARHGAP42 SLC16A1-AS1 ENSG00000267340 KLHL6 HRH2 PLA2G6 SLC46A2 SMIM38 MALINC1 ZFPM2 ENSG00000288979 CCDC113 ENSG00000287202 ATP6AP1L ENSG00000285672 CELSR3 ZNF137P NOXA1 GRIP1 SAMD10 GVQW3 BMP8A ENSG00000276698 R3HDM2 NEDD4L ENSG00000285804 WNT6 ENSG00000278875 CCDC188 GNRH1 FBLL1 UPK3B ENSG00000189229 CFAP44 ENSG00000280057 ENSG00000232876 TANC2 ENSG00000253200 DLGAP1 RCAN1 FBXL20 ZNF827 ENSG00000241889 DSE MST1P2 LINC02614 GABPB1-AS1 LINC00887 GLI1 ENSG00000263089 ARHGAP25 ANKRD20A5P WNT5A ENSG00000274292 SLC25A34 SLC7A4 ENSG00000267404 AGER CHROMR ENSG00000232545 NAV2 ENSG00000261056 IL21R RNF150 DNMT3A FOXN2 ENSG00000285774 WNK4 LINC00174 HHIPL1 CHD1-DT KLRG1 AGAP4 MEIOB ZFP90 FRG1-DT KDM6B TTC28-AS1 NET1 ENSG00000270090 TRPC1 ENSG00000266718 NPIPP1 LMNTD2-AS1 SRGAP2C FAM186B MSS51 ITPR1-DT PAPLN-AS1 NOL4L ENSG00000228327 PPIEL DICER1-AS1 PTP4A3 CDC42-AS1 ENSG00000258811 ZNF514 SLC48A1 YPEL1 ZFHX2 PRSS27 FRS3 NR2F2-AS1 HCG27 ZNF536 GTF2IP13 ENSG00000198358 HS3ST5 NYAP1 OR2L13 NBPF17P SETBP1 NDC80 LINC00622 SLC7A11 L3MBTL4 SLC6A16 ACKR2 SCARF2 ADIRF-AS1 NATD1 SCART1 MCTP2 YJEFN3 ENSG00000270021 ENSG00000288839 SOCS3 ENSG00000231305 ZNF839 NLGN3 TM9SF5P EGFEM1P DACT3-AS1 ARID1B ENSG00000268218 BAIAP2L2 FIGN TMEM178A MAGI1 SLC23A3 PJVK C1orf220 TRMT9B*

		CKMT2-AS1 BTBD8 LINC00672 BNIPL AMOTL2 PSRC1 ZFPM2-AS1 GPT ENSG00000276952 MATN4 ENSG00000257027 AMPD3 ENSG00000270605 LRRC39 ENSG00000228852 ST6GALNAC3 BEND5 TMEM263-DT ENSG00000285813 LINC02381 TMED2-DT SSBP2 CREB3L4 SLC44A3-AS1 TNFRSF25 COPB2-DT CCR7 RECQL5 PROCA1 ENSG00000280077 MIS18A-AS1 ADCY1 ENSG00000270933 INHCAP RGS1 GRID1 STK32A-AS1 ZNF862 KLHDC1 BBX ARHGAP8 RERE ZNF608 ENSG00000288808 ENSG00000235902 SLC25A42 CBX3P7 ENSG00000277978 KIF23 ATL1 KIAA1109 ENSG00000259495 ENSG00000287562 MOCS2-DT HFM1 ENSG00000111788 ACSS3 SPEG ZNF577 NR3C2 INPP5F IQGAP3 ZNNT1 ENSG00000287837 CACNA1C DOC2A MIR4453HG GSEC TPTE2P1 DBP DCST1 LRRC63 ZNF563 ARHGAP32 EGFL8 CNN3-DT RENO1 TAS2R31 PCED1B LINC00964 ENSG00000282556 EFNA5 ZNF385C IMPG2 ENSG00000233110 CENPE ENSG00000239467 TMCO1-AS1 ENSG00000253476 KRT80 ANO4 SASH1 KCNQ4 SLC22A23 TUBB2B C9orf72 ENSG00000215838 ENSG00000271780 INSM1 ABCA10 ZNF493 WBP2NL NPIPB15 AFDN-DT ENSG00000289067 MKLN1-AS FOXN3 DSTNP1 ENSG00000266934 CD109-AS1 TAS2R4 PNMA2 C6orf163 PRKACB-DT TBCE ENSG00000228137 LINC02251 ENSG00000223884 ENSG00000258559 KRTCAP3 ENSG00000286004 HOXA2 ENSG00000245552 ARHGEF2-AS2 HIC1 KC6 RWDD3-DT LRP1 ZNF790-AS1 ENSG00000261026 ADHFE1 LINC02352 DMXL2 LINC02391 LINC01909 ENSG00000255429 KIF12 NBEA ENSG00000285684 CASC15 BTN2A2 ENSG00000278058 LINC02405 MUC6 CACNA1C-AS1 MEX3A CLASP1 MRPL23-AS1 ENOX1 SPRY4-AS1 BACE1-AS FCHO1 SIRT5 LINC02525 OR2L2 TRIM66 AKAP6 LBX2 INTS6-AS1 CHD2 ZNF132 ENSG00000230699 TIGD4 CSGALNACT1 MFF-DT ERBB2 FAM225A RAB3GAP2 SRGAP2B PABIR3 SLC22A15 ENSG00000262703 ZSWIM9 PIF1 LINC00523 ZHX2 NAPSA LINC01102 BAZ2B C1QTNF6 ENSG00000284946 LINC00858 SLC25A27 FNBP1P1 LRRC37B ZNF501 GTF2IP23 CASP16P CEP170P1 ENSG00000267683 CCDC17 TBL1XR1 ENSG00000274414 FBLN2 ELOVL2 CBX7 LINC01144 ENSG00000223945 ENSG00000271590 ENSG00000286159 DNPEP-AS1 CCDC9B ANKRD44 SRGAP1 MBTD1 MAP3K12 ARHGEF35-AS1 ENSG00000255521 PRX RAD50 PCMTD1 SYNE1 ENSG00000289177 PSMG3-AS1 FER GSDMB ENSG00000255557 CRELD1 MRPS31P5 ENSG00000260563 WDR27 KRT89P ENSG00000270540 FAM72A CDK5RAP3 PDZD2 CEP192 IFT88 ENSG00000289059 CAMK4 SLC24A1 FAM66C HDAC11 CACNB2 TENT5B ENSG00000267546 SMIM2-AS1 TBC1D32 ZNF606 ENSG00000278918 ENSG00000276449 ENSG00000205444 RUNX2 LINC00659 ZNF169 KRT7 DLGAP5 RAC3 ZNF396 SOX6 WEE2-AS1 LINC01687 SGCD LIMD1-AS1 LINC00339 ENSG00000286757 ENSG00000227885 ENSG00000257298 TEX52 ENSG00000262691 FRG1JP ENSG00000288538 LRRC20 VCAN ENSG00000232611 PCYOX1L LURAP1L-AS1 LINC01447 NHS GLI2 SHROOM4 ZNF594 ADAM8 HSPA7 ICA1 NAV3 ENSG00000288748 NUDT13 ZSCAN23 MT1F ENSG00000273374 MFSD14C C2orf68 ACOXL PRIMPOL MAMDC4 CLIP3 ENSG00000198580 CNIH3-AS1 DDX11-AS1 NLRP1 TGFBR3 BEND3P3 CCN1 LINC01583 GRIN3B ZNF615 ZNF252P-AS1 FRG1GP CLEC2D CPB2-AS1 ATXN1 ENSG00000273243 APH1B PDLIM1P4 ENSG00000273203 TPM2 MGAT3 ENSG00000213062 ENSG00000272502 CT75 NCOA2 ENSG00000279518 S100PBP ENSG00000286909 ENSG00000280157 ZNF37BP FAXDC2 SGMS1-AS1 ACACB ENSG00000230490 ENSG00000286364 ANKRD33B KCNQ3 SPRY4 KATNAL2 ENSG00000279833 ZNF135 TP53TG3D ZKSCAN8P1 PER1 ENSG00000262587 HDAC10 ENSG00000285943 ENSG00000270171 CUTALP NKILA ENSG00000222044 ENSG00000237807 SHISA7 ZNF620 OIP5-AS1 FNDC3B RGS17 JAKMIP3 LINC02728 CLDN1 GNG12-AS1 SMG1P7 ENSG00000285184 LINC01341 PLEKHG1 ZNF789 LINC00638 GEMIN7-AS1 ATP8B3 SNX18P1Y ENSG00000274276 SNTB1 LINC02163 COL24A1 SLC20A1-DT ARHGAP24 ENSG00000251143 CHIAP1 JMJD7-PLA2G4B FAM47E FHIP1A LTK ALKBH6 ZNF860 RN7SKP296 NBPF14 ENSG00000273325 ENSG00000280099 ENSG00000266313 C2orf88 LINC00663 C13orf46 LINC01622 ENSG00000274220 P2RX7 ENSG00000253854 PLEKHA5 TBX6 SPTBN4 NOXRED1 ENSG00000244560 TMTC1 TSHR GSTM2 EPHA10 IGSF22 SLC35E2A BACH2 ENSG00000256139 NALT1 PHACTR2P1 ENSG00000280053

		COL27A1 PPP1CB-DT FXYD6P2 PHLDB2 BAIAP2-DT LENG8-AS1 ZNF486 ESR2 HSD3BP5 DNAAF9 NEMP1 MIR3189 JRKL ENSG00000238260 ZCWPW2 ENSG00000277007 KMT2C ENSG00000261474 ENSG00000277287 ZNF45-AS1 TMEM147 GULP1 NR1D1 ENPP3 NEK2 C1orf162 SATB1 ENSG00000283959 YPEL3 LINC01320 COL12A1 ENSG00000279794 XYLT1 YPEL2 ENSG00000272720 ENSG00000260855 ENSG00000286388 ENSG00000263280 BCDIN3D-AS1 EYA2 ZNF471 ENSG00000272529 LINC02293 LINC00847 LYPLAL1-DT MYADM TMTC2 DUSP8 ZNF519 PPP3CB-AS1 L3MBTL1 ATXN7L3-AS1 MEIS1-AS3 MOB3B LINC01219 ENSG00000284526 AFF3 PCP2 ESPL1 ARHGEF10L ENSG00000271971 DNASE1 LPP ENSG00000271533 ZNF554 HECA ENSG00000287855 SCML2P2 TNIK ENSG00000212978 ENSG00000272264 ENSG00000272054 ZSCAN18 MACF1 MPPED2 MEF2C-AS1 EPB41L4A ENSG00000278869 MBNL2 SLC28A3 ARVCF STARD4-AS1 MEX3B ENSG00000260051 LINC00308 RASSF3 ENSG00000273451 ENSG00000288884 HSPE1P26 PPM1L-DT CCDC110 NBEAL1 ENSG00000276317 TFDP2 CLHC1 UCN KCNAB3 ENSG00000225931 TUG1 STX18-AS1 ENSG00000267868 PTK2B KEL C22orf46 ENSG00000280399 ENSG00000277938 SLC9A5 SH2D1A SH3RF3 ENSG00000286753 B4GALNT4 LINC02343 MDGA2 ENSG00000285725 NAV2-AS6 ENSG00000279161 ZSCAN30 DIP2C CLDN4 ENSG00000287766 MSH5-SAPCD1 FAM13A-AS1 ODAD1 ENSG00000268970 STAG2 ZNF277 MAML2 ENSG00000262877 NFATC1 NMRK1 SH3GL1P2 ZNF197 NEURL2 GABPB2 ENSG00000228793 FAHD2CP LINC00482 HTR2C RPS4XP16 MIR635 ENSG00000277496 SYPL1P2 CFAP61 CMTM1 CTIF LINC00173 LINC00906 LINC00898 COL15A1 RAP1GAP2 TRPV6 LMLN SLCO4A1-AS1 ENSG00000260285 ATP4A ENSG00000236829 LDLRAD4 CHMP1B2P IQCH HSD17B1P1 CCDC26 ENSG00000277152 NLRP6 SAMD13 NEIL3 ZNF8 CEBPB-AS1 FZD2 TEAD3 FAM72B MYO5B MATN1-AS1 NOVA2 FAM86B1 PIBF1 PRKG2 LRRC37A2 ENSG00000272625 ENSG00000232098 MTND5P1 SEC31B SLC12A6 NUDT7 ZNF665 ENSG00000271947 TLR6 ANKRD28 ENSG00000278041 ENSG00000259820 CAMK2D KCTD13-DT CCDC122 ENSG00000232470 NTNG2 HMCN1 ENSG00000286174 LAMB2P1 HOXB3 GSN-AS1 ENSG00000261468 KLHDC7B ZMYND8 ZNF716 KIF16B ARHGEF2 ENSG00000259868 ENSG00000274225 CDC25C PAXIP1-AS2 ENSG00000177788 ZNF609 LINC01695 SIRT4 RSKR FAM24B ENSG00000280149 LINC01517 ARHGAP33 TCF12 CCNG2 ENSG00000286985 ENSG00000251194 KTN1-AS1 PLEKHG2 LINC01356 WDR88 C15orf65 FAM86B3P HOXB13 PPARG TMEM44 ODAD4 TANC1 CORO2B LINC01366 LINC01572 NUPR1 ENSG00000267681 SH3RF3-AS1 ENSG00000227329 LINC00565 RBMS2 COL4A3 EPM2A-DT ENSG00000289378 ENSG00000273472 DNAJA2-DT UNC5C-AS1 ENSG00000232528 ENSG00000279080 SYNE4 ENSG00000285763 TSHZ2 EGR1 CCL17 HDAC9 DLX4 MAGOH-DT HSD3BP4 NR4A1AS RPL23P2 ZCCHC18 KIAA1549L MYT1 MSI1 ENSG00000233817 ENSG00000279381 ENSG00000280332 ENSG00000278390 CYP4V2 ENSG00000258853 SCNN1D AGO4 ZBED3-AS1 EN2-DT EWSAT1 TM7SF2 MAP4K1 ACCS HECW2 PKD1L2 PPM1M KAZALD1 UBR5 ENSG00000272372 HERPUD2-AS1 PCMTD1-DT PEAK1 ENSG00000276564 C11orf71 TOB1-AS1 CBLB LINC01322 UGDH-AS1 ENSG00000286535 CDK6 SNORC TEAD2 KLLN PKD1P3 CHL1 VANGL2 DLG4 LYST RAD51-AS1 EOLA2-DT ENSG00000264112 ENSG00000259341 STPG3 GCAWKR ZNF516 UNC13D LINC01530 LMOD1 ENSG00000285999 ENSG00000235119 NUDT6 CSRP1-AS1 ENSG00000287262 RGL3 LNP1 ZEB1-AS1 PRKCZ-AS1 ZNF221 SAMD9L ENSG00000286373 ACRBP RBM20 MAML3 EDNRB ENSG00000231703 ENSG00000267666 SHANK1 ARHGEF39 ENSG00000287878 ENSG00000250961 CPT1B SRGAP2 ANXA2R ENSG00000269925 STK32A ENSG00000279048 CELF2-AS1 ENSG00000285980 SPATA6 TSSK3 ZNF234 ENSG00000239415 ENSG00000255182 SUCLG2-DT TPRXL IHO1 ZNRD1ASP EBF3 KBTBD11 ENSG00000287110 CNTNAP4 ENSG00000287737 PAK3 ENSG00000288880 LINC00393 PDGFA-DT TUBA3D ENSG00000215493 AGPAT4 LNCOC1 SFMBT2 NOX4 ENSG00000271259 ENSG00000269918 NPIPA1 UACA GTSE1-DT LINGO1 SETD5 TMEM169 EBF1 SALL2 TNKS CYP2E1 LAMTOR5-AS1 NRG2 CTH ENSG00000273355 ENSG00000279315 GABARAPL1 C5orf34 APPBP2-

		DT ENSG00000289506 ZNF818P KLF12 ENSG00000289042 ENSG00000269397 RUNDC3B ENSG00000255028 EIF1B-AS1 ENSG00000253573 RIMKLB AHSA2P OBSCN AACSP1 TNS1 IL1RAP SMPX ENSG00000285679 PPP1R10 SSR4P1 ZNF436-AS1 ENSG00000259668 ENSG00000230454 SEMA6A-AS1 ENSG00000260077 DEF6 OSGEPL1 RPH3AL FCHSD2 SLC4A7 PLEKHH2 TRERF1 HERC2P4 ENSG00000244055 SNRPGP14 GLI3 PTCH1 KRTAP5-AS1 CAPN10-DT ENSG00000261959 VARS2 ATP6V0E2-AS1 MST1 FOXD2-AS1 JAZF1 BRPF3-AS1 ENSG00000288756 ENSG00000287729 DYNC2H1 ENSG00000267199 MTUS1 ENSG00000270110 PTPRVP PHBP13 ENSG00000272791 ENSG00000273373 TNFRSF13C CPEB1-AS1 INTS4P1 MST1L GAB2 LINC01249 STX16-NPEPL1 ENSG00000241886 GK-AS1 GDPD1 ENSG00000279926 FLJ43315 ENSG00000289318 RGS9BP ENSG00000197813 ENSG00000279759 SNRPGP4 ENSG00000277639 ANKRD31 C16orf86 ENSG00000273893 ENSG00000286220 AGAP2 ZNF521 LHFPL3-AS1 ENSG00000275910 CHAD ENSG00000253395 CPED1 ERVW-1 ENKD1 HMGB2 ENSG00000286207 FBXL2 PDXDC2P-NPIPB14P CORO6 ENSG00000279360 LPAR2 PRICKLE2 ANGPT1 USP38-DT PPT2-EGFL8 LINC02656 LINC01531 FBXO43 CHAC1 MAST1 KLHL32 TMEM100 LTB4R RNF165 ZMYM3 CREB5 SHF FAM200B ZNF429 NNT-AS1 ADGRG6 RNF213 PHLPP1 PLIN1 ENSG00000284634 SPTY2D1OS MIR3936HG ENSG00000278998 ENSG00000093100 PRKCE FGGY-DT WDR31 SRCIN1 MIR3142HG TIGD7 GLIS1 LINC01625 PDE4DIPP6 CACNA1D UHRF1BP1 TMEM51-AS1 IVNS1ABP ID2-AS1 CD37 DUSP19 OGT ENSG00000272002 KRT8P46 GRB10 CCDC154 TBL1X ADGRD1 EPHA3 FRG1DP BSG-AS1 ENSG00000259772 LINC02604 EPB41 SCAPER PARGP1 ENSG00000216775 ENSG00000279672 MCPH1-AS1 ENSG00000274460 CLDN15 DOCK9 RPS6KA2-IT1 ENSG00000259088 LEAP2 SEPTIN5 MKRN2OS ENSG00000225649 EZH1 PPARA TLE1 ENSG00000242861 NCKAP5 LNCTAM34A ENSG00000260604 LINC00933 LINC02918 CACNB4 ENSG00000261786 CCDC140 ENSG00000257252 PRIM1 SDAD1P1 ERICH2 ENSG00000287385 ADGRB3 INPP4B AFAP1L2 KLF7 ENSG00000261476 SPATA25 ENSG00000275481 ENSG00000261799 MAP2K5 MSH5 PLCB1 HIVEP2 ENSG00000279930 ADM2 BLACAT1 RPS10P7 ENSG00000272983 ENSG00000223947 MPRIPP1 KCNJ4 SOX5 GRID2IP PARP11-AS1 SBF2 ENSG00000286017 ADRA2B PCF11 IQCA1 SEPSECS TRIM7 NFIB CCDC191 MTMR9LP COL4A4 PCSK4 ENSG00000261114 PABPC1L TMPRSS5 ANKFN1 ENPP5 ENSG00000287957 CACNG8 ENSG00000259065 SKOR1 CCDC18-AS1 ENSG00000271833 TTC28 SGCA SEPTIN7-DT FGD5P1 SLC16A13 LINC00920 PLCL1 TEX22 DDIT3 ST20 LINC01355 ENSG00000287236 GDPD3 STK4-DT CYP2D7 EDA CNTRL IFIT1 ZNF460-AS1 DENND3 ENSG00000226744 ENSG00000282897 ENSG00000262580 ENSG00000267504 GMDS-DT HTR6 DOCK10 GNMT TMEM198B EPG5 COL2A1 CNTNAP3 FCGR2C ENSG00000236529 VPS13B-DT ENSG00000286485 ARAP2 IGF1R PRKD1 LRRC4B EFEMP2 LOH12CR2 NLGN1 ZNF674 ENSG00000289161 RAPGEF3 LINC00342 SHROOM3 FBXO41 ITIH2 PDCL3P4 PRR5L TTC21A LINC02021 SLC12A5-AS1 ENSG00000285925 ENSG00000272425 ENSG00000278635 WNT2B LINC00205 ENSG00000250397 EXTL3-AS1 ENSG00000272668 ENSG00000268575 HOXB6 ZNF528-AS1 TMEM161B-DT ENSG00000255026 ENSG00000261094 FRK TBX19 CDK5R1 GNAO1-DT PAX8 TMEM116 ENSG00000273261 IRF1-AS1 ENSG00000251417 ABHD12B SLX4IP KIF18A LINC01002 LINC02615 TNFRSF11A ENSG00000289370 ZNF280B C19orf54 ENSG00000269896 LINC02878 KRT8P12 ENSG00000287650 KMT2A ZEB2 ENSG00000269978 ENSG00000279588 DBIL5P2 ENSG00000258101 JMJD1C ENSG00000261270 PRKAR1B-AS1 COX6B2 CICP14 ENSG00000205041 PDE11A PAIP2B ACBD4 HJURP KANSL1L-AS1 ARID4A MTCYBP21 ENSG00000286248 KIAA1614 OR1F1 ADAMTS13 AZIN2 C21orf62-AS1 CBLN3 LINC02289 C3orf18 CYB5RL DNASE1L2 RHOQ-AS1 ENSG00000289250 LINC02649 RNF157-AS1 OFCC1 CREBRF IFT81 ENSG00000279041 ENSG00000251364 KLC2-AS1 INSR TTN NBR2 PHF13 ENSG00000260279 ENSG00000286198 ZNF93 ENSG00000280128 CLDN20 ETFBKMT CIT BTBD19 ZNF607 MTSS1 ENSG00000197815 ENSG00000267277 SLC9A3-AS1 WNT5A-AS1 MYO1F KIF28P KLF15 HMMR ENSG00000274922 MORN4 CEP295NL GBAP1 CCDC146

		RN7SL67P ENSG00000275180 ENSG00000272405 FAM227A HOXB9 CDK19 BRME1 ENSG00000257176 Y_RNA GOT1-DT RHOBTB3 LINC02099 RNU6-850P ENSG00000261118 INTS6L PKDCC ENSG00000280225 CCDC171 FOXP1 CATSPER2 ENSG00000289405 SCN8A RAP2C-AS1 CDNF ZFHX3 HEMK1 FBXO48 PAPLN PAX3 EFCAB13 ANKRD27 TCP11L2 TMCC1-DT ANK3 SLC16A8 ENSG00000284968 LINC02175 MMP25-AS1 BMPR1B ENSG00000243243 GATA6-AS1 CARF GTF2IRD1 TUBG1P ZNF19 TEX21P ENSG00000261211 DUSP16 ANKRD34A BCL11A ENSG00000287910 DDR2 LHFPL3 ZRANB2-DT LINC00648 SAMMSON AGAP1 GPT2 PTK6 RIPOR2 CFP THBS3-AS1 ZNF37A DOCK3 ENSG00000227775 ENSG00000235381 ENSG00000264548 PDK4-AS1 BCAT1 MCF2L2 PARD3B RN7SL262P ENSG00000280061 CD63-AS1 SMG7-AS1 NKX3-2 RASGRF2 FMNL2 ENSG00000284959 TRPS1 ANKRD55 GTF2IP12 ERN1 KANTR ENSG00000223393 ENSG00000286545 GAB1 SNHG14 FBXO32 ENSG00000267649 CEP57 MYH7B ENSG00000287820 GATM JPH3 C20orf204 ENSG00000245317 ENSG00000279811 FRG2B KLHL3 HSPG2 ENSG00000279838 PTPN13 C21orf58 DAPK1 VAV3 RMI2 RNF139-DT ATP10B CYP24A1 RFTN2 NPAS3 ENSG00000287036 TNS2 HCG20 ATP8B1 ICAM5 C2orf74 SEMA4C CCNYL2 EIF2AK3-DT UBE2Q2P1 PDGFC ENSG00000268204 CSPG4P13 POU5F1B C8orf44 CENPF ENSG00000275719 PHF21A DACT1 ANKRD23 ENSG00000232546 PPIL6 PRKAR2A-AS1 RNF32-AS1 LINC01252 VPS13B N4BP3 ENSG00000265298 LINC02610 HCG25 CNOT6L CHKB-CPT1B GOLGA8B ERVH48-1 OSBPL5 CDHR2 AMZ1 ELOA-AS1 PIK3IP1 APBA1

Table S5. The Venn diagram shows the intersections of upregulated genes detected in MB and Mel Z cells formimg VM. Related to the Figure 3.

**Names**	**total**	**elements**
up 976 mel z up mba-mba	51	*BYSL PDCL3 CCDC97 BAK1 FAM98B HGH1 PHACTR1 NGFR PSMD2 H2BC4 HMOX1 SERPINB8 CYRIA OSGIN1 PSD4 TAX1BP3 NUDC SLAMF7 POLR2A SCO2 CDKN1A ADM SRM TTC4 MT2A PRCC MRNIP S1PR3 ENSG00000288873 PRPF19 LSM10 SPHK1 CCDC86 KCNC3 PLEKHA7 ATF5 LRRC15 NXF1 GAR1 SF1 PDE2A NAP1L3 CORO1A GEMIN7 TMED7-TICAM2 NDUFB2-AS1 SERPINE1 ARC BRPF3 CCN3 SF3B4*
up 976 mel z	925	*PNMA1 KCNJ2 GSKIP GNL3 UTP20 IFNA20P ENSG00000264577 PDF PMPCA STOX1 ENSG00000289164 AEN PRADC1 IRAK1 BRIX1 SSC4D CYCS COPRS KBTBD8 CLUH PNPT1 ENSG00000264666 ING3 LYAR ZNF576 RRP7A MRPL1 PHACTR3-AS1 NOC4L POLR3K PRDX1P1 UFSP1 PUF60 BLOC1S2 CMPK2 ITGA3 RIOK1 RABGGTA CACTIN POU3F1 SNHG25 GRPEL1 RELT LMNB2 SDAD1 ENSG00000279249 KPNA2 KPNA3 ENSG00000279692 EOLA2 CHRNA5 ENSG00000279443 ENSG00000248968 CAVIN3 GFPT2 ATP5MC1 TMEM250 PPIL1 BCLAF1 FAM98A PGBD5 TPI1 H2BC21 IPO4 SRSF6 ALG2 POLE3 DUS3L BOP1 PRR19 PLIN2 PMF1 HSP90AB2P MIR193A HNRNPR GCLM METTL25 ID1 PSMD3 EEF1AKMT4 TIMM22 AVEN ZNF35 PSMD11 ELAC2 EMC8 LINC01679 ZNHIT2 NCDN MRPS12 C3orf52 DOHH ENDOG SETMAR RFX5-AS1 FGFR3 ENSG00000261762 PTGES ENSG00000279965 ABCF2 CDC6 ASNSD1 TENT5C HSPA8 TMEM11 MRPL15 LYSMD2 MAFA SAC3D1 H2BC11 COMMD5 ENSG00000256955 SLC9A3R1 PRR5 FXN CHMP7 FKBP4 LONRF2 SF3A3 ENSG00000267405 RABEP2 NOC3L BST1 HCFC1 ACTR5 KRT15 TRMT6 ARMC6 KRT17 EIF4A3 RBM38 PXN-AS1 RDH8 ALYREF ZNF672 LINC00475 HEXIM1 IFITM10 RPL5P8 SNHG9 SNORD88A PPP1R14BP3 LANCL2 H2BC18 CHMP6 CYP19A1 MANF EIF4E2 ENTPD7 S100A3 FAM174C GRWD1 ENSG00000275106 FETUB MRTO4 C1QBP TIMM17A PDSS1 SMG8 PGAM1 ENSG00000275993 SHLD3 PSTK SEC13 PAQR3 NECAB2 ENSG00000267505 SCRN1 PSMC4 PTMAP4 UCN2 ZNF511 IRF7 HSPA2 FNTB EIF2S1 SERTAD1 HRCT1 ENSG00000217275 FDX1 EIF5A MAP1A MCM10 DNAJC25 TRPM2 NOL11 USP18 ENSG00000223461 PSMD12 E2F4 POP7 ENSG00000262140 SURF4 BCAS2 TCF15 LDHAP3 GCH1 ENSG00000289117 SRRT RBM3 SNRPG BMS1 PODXL GEMIN4 SELENOS CTU2 CDR2L POR CSRNP1 ZBTB7C NSUN5 SF3B5 MPDU1 ZNF18 NAA15 LCMT2 TOMM22 CHPF2 MCAT MRPL9 RUNDC1 VCP GAL*

		ABT1 RAVER1 RCC1 ENSG00000261889 PDXP MBLAC1 B9D2 GCLC HYAL2 ENSG00000257497 SEC61A1 ENSG00000279212 ENSG00000279953 ENSG00000249050 GALNT10 SLC2A1 BANF1 INTS5 PES1 TIMM8A WFS1 MMP11 NUP35 URB2 NCOA5 H2BC12 CTR9 INTS7 AP1S3 HAS3 SRCAP ENSG00000286181 UBASH3B MAN1A1 PYCR3 RABIF CCDC103 ASH1L-AS1 ABHD5 U2AF2 ZMYND19 CBWD1 TUBB8P7 TSSC4 AAR2 GPS2P1 ZPR1 ENSG00000280486 LINC01979 ZNF582 PRMT5 DCAF4 RNF126 TMEM70 RAP1BL SHQ1 SERPINI1 RBM14 FASTKD5 COA4 ENSG00000280010 YRDC IPPK EFHD2 SESN1 ADAMTS15 SDHAF2 RRP1 FSCN1 PUS3 NKX3-1 RINL NOC2LP1 CTLA4 WDR74 GLMN ABCB9 CCNQ NECTIN4 ABCF1 UFD1 CFL1 MRPL14 MPHOSPH6 HS6ST1 WDR77 LINC01615 TIMM23 PSMC2P1 ENSG00000260136 DEGS1 DYNLL1 DDIAS CTPS1 ENSG00000286570 EMP3 EIF3J SNX11 CKMT1A LDHAP7 FARSA TUBA1C H2AX TRMO SLC30A1 KLHL18 CCNE2 SNRNP25 DPF1 TBX3 SWSAP1 STIP1 FUT11 C15orf39 GPER1 MYDGF CHST7 CCT3 CSKMT S100A2 NOP14 NCL DMRT2 COPS3 PCCA-DT SOCS1 MCM4 NPLOC4 ID2 TRIM28 DDX21 PHLDA2 TST POP1 COA7 UTP11 KTI12 MESP1 MRPL27 RIOX1 CANT1 ENSG00000227218 SEC14L2 ENSG00000263823 RPUSD2 SMTN EMC6 CYB5R2 DNTTIP2 ZNF143 SAMD15 MED31 GOSR2 ENSG00000279539 CA8 ISOC1 BRMS1L PET117 UQCRFS1P1 CLSPN LENG1 BCL7B SHPK C14orf119 C1orf53 TMEM115 TMEM41A ZNF628 EXOSC4 POLR1G EHD1 SRA1 BRD2 ASH2LP1 TRIML2 LAMC2 ARPC5L OLFML3 FAM118B USP5 RPP25 MAGEA12 EBNA1BP2 CIAPIN1 PTRH2 SNORA33 ZNF668 STAMBPL1 LAP3 AKAP8 TUBB4B ENSG00000286996 TIMM23B IMP3 MIRLET7BHG ZNRD2 SEMA3F CAPN15 RIMKLA TOMM40 RPL23A SBSN ENSG00000215014 PTMAP5 ZMPSTE24 H3C3 PSMB3 MARS2 NFKBIA SDF2L1 AP5S1 ENSG00000287721 MED19 ENSG00000271851 ARMC5 BEND3 MRPL36 KRT12 MON1A ZNF804A RSL1D1 WDR3 SNORA73B NAT1 ZFY EPHB2 MIEN1 ALG1 CALM1 PLEKHA6 C8orf33 NME1 DDX56 FAM222A TMEM11-DT GMEB1 GEMIN5 ENSG00000263826 ENSG00000276931 DDX20 UTP15 EEF2KMT MYBBP1A CCT6A PIM2 CPNE7 GINS2 RCBTB2 NOP56P1 MIR210HG H1-2 PVR PNP ST6GALNAC1 METTL1 STEAP1 BLOC1S4 ANKRD37 SIAH2-AS1 LTV1 ENSG00000232748 RYR3 CHST8 SNORD83A EIF6 SEC24D RNF25 POLR2L MRPL4 JPT1 LINC02119 ATP6V0D1 POLR1C ID3 TM9SF1 MSC ATXN7L2 PTRHD1 SPATA5L1 NOSIP UNC5B EIF3B RPL36A L3MBTL2 FASN ILF2 SFPQ AKR1B10 RARS1 DOLK SMIM3 SQSTM1 HPS6 RPP40 C7orf26 YARS2 HMOX2 ALMS1-IT1 CDK12 TAGLN2 H2AW PUSL1 ATP6V0B BRMS1 XIRP1 ATP1B1 PELO MRPL12 CCDC137 CKB PSMB6 ENSG00000260912 C2orf27A ENSG00000289554 HM13-AS1 CCT2 MRPS17 MRGPRX4 SPRTN ENSG00000259704 ENSG00000276853 NOP2 SYN1 LETM1 SLC25A44 FAM135B ENSG00000273568 CLP1 TMEM273 ENSG00000266651 ENSG00000178412 VSNL1 DHX30 PRDX1 IGFL2-AS1 MRPL54 H2BC5 LSG1 MERTK PPP2R1B RRP9 PUS1 ENSG00000279467 TUSC2 OLFM1 STON2 TUBA1B CRY1 MSX2 SCAND1 FBXO33 LINC01465 FAM169A ENSG00000222032 CHCHD7 SLBP SRP68 NRBP1 SLC25A22 CARD6 ENSG00000255224 YJU2 NPTX2 RPUSD1 RRP36 RAB27B TMEM47 MFSD2A CNTF ENSG00000261737 FA2H ENSG00000279605 RPLP0P6 MPV17L2 SMIM12 LRATD1 HSPA8P1 CALR SNRPD1 MEPCE BZW1 RRAGA PCNA ABCE1 GPATCH4 LDHA FER1L4 CHUK HR POLH-AS1 ENSG00000274270 STX11 DNAJA1 ALDH1B1 INPP1 NOP16 CYB561D2 TMEM214 IER5L WDR4 SZRD1 RRS1 KDM8 NOC2L SPTBN5 HSPA1A POLR3E PDCD2L ISG20L2 CCNYL1 OGFRP1 ENSG00000274015 NT5C1B DHX37 PRMT6 ICOSLG C3orf80 FTSJ3 GABPB1 UTP14A ENSG00000233388 RRP1B DKC1 MRPL17 THOP1 TRIAP1 ZNF653 ENSG00000265749 ENSG00000213087 TFRC MRPS26 DHX34 BRINP1 HSPA8P9 MT1X KEAP1 EEF1A1P8 NOP56P3 ENSG00000267397 NUP50 ARRDC4 ENSG00000279641 SRP19 RN7SL832P ADRM1 DIMT1 PYM1 AIMP2 ENSG00000240652 TNFRSF12A INO80C ENSG00000272990 KPNA1 TXNIP TAF6L TMPRSS2 NXT1 SRSF8 PTBP1 DGCR11 ENSG00000272768 ENSG00000278974 NSDHL SNORD12C ADPRS EIF4G1 LINC02735 LYPD1 IDH3A HSP90AA1 LRRC47 DHX9 SMIM24 ENSG00000233825 PHF5A PHRF1 IQCN PGP ABCG2 MRPL18 PGAM5 RBM12 CBLN1 TYROBP NIP7 TMEM158 PECAM1 PRPF4 NOCT HNRNPD-DT DPP3 TUBB2A DDX28 SACS UBE2S MYH15 TBCC CDYL2 PLK2 CCR1 NCS1 CSTF2 POMP GMPPB AHSA1 TFB2M MGAT2 NUP188 NOP56 SRXN1 TATDN2 LINC02577 SYT6 TAF13 NUBP1 SHLD1

		TRIM21 EIF5 PREB MB C12orf43 FHOD1 GTPBP4 SPNS2 UTP3 ANKRD30B PCDH19 C16orf91 ARRDC3 MUL1 TCAF2 TPT1P9 PUS7 TUBB6 DCTPP1 FDXACB1 XPA ZBTB9 NUP153-AS1 CLU DPH2 DDN AUNIP MSX1 EPHX1 ZNF784 NCBP2 HNRNPAB SELENOTP1 VPS18 ENSG00000264985 GADD45GIP1 SLC27A4 FEN1 PPIF HSPBP1 EFNB2 VPREB3 SLC20A2 ENSG00000260273 BAG2 ELOF1 PCDH10 PRR7 SRPRB GLYR1 ADAT3 NOL6 SGMS2 SLC25A33 MYH7 LRWD1 PTS LRRC59 TXNDC9 TRAC DDX54 MROH9 RTP4 GLRX5 ENSG00000259041 CEBPA TMEM201 SURF6 PA2G4 NOS1AP RRP12 MRPS2 POLR3H RILP ENSG00000289307 HSPH1 CARD8-AS1 ACER2 ACTB SRSF2 SLC39A3 NSMCE3 NAP1L1P3 SMN1 FAAP100 PSMD1 DSEL ENSG00000237493 SRFBP1 BCL10-AS1 PDCD11 PRELID1 EXOG C8orf76 ATP6V0D2 NFKBIE ENSG00000272604 PKM HSPA1B DDX23 GAPDHP63 SPRR3 NOLC1 VWA5B2 HSP90AB3P ENSG00000286064 UBIAD1 NPTX1 SNORD104 FAM217B SCAMP2 ENSG00000228477 TSR1 PRR7-AS1 RGMA GMNN KRT10 ZNF295-AS1 CCT5 FDXR MCRIP2 POLR2C TLCD3A PSMD7 SHISA2 ENSG00000283064 SNRPB ENC1 PNO1 MICALL2 TIMM21 TLCD1 PSMC3 PIGW MFSD5 RASL11A ENSG00000261888 MIR22HG TMEM199 EXOSC6 LHFPL3-AS2 TRMT10C PMEPA1 TNFSF9 TACO1 NUDT15 ZNFX1 KCNK5 DCTN5 PSME3 FUNDC2 PDCD6IP-DT ENSG00000279133 CHAC2 TOE1 TMED1 GEM CCL3 TM4SF19 ZNF341 ONECUT2 CALHM2 CRISPLD2 DDX3X PGD RND1 ENSG00000277117 CCDC71 FAM180A SLC52A2 LINC00973 RAB5IF EIF4A1P7 SLC10A3 SAR1B SLC25A5-AS1 PNPLA2 UBE2J2 GNL2 CFAP45 PDE12 SPOCD1 ZNF622 ENSG00000250031 POLR3B LIPT2
up mba-mba	1146	*CREB3L1 AC010761.4 CD44 KRTCAP2 AC026740.1 MMP2 LINC02377 AL390719.1 EDN1 ZNF492 NAPRT DDT CXCR4 H3-3A AL645608.7 ENSG00000290535 OR2B6 AC109588.1 MT-TG XAB2 AL137058.2 PRKCG ZBTB12 LYG1 AC090498.1 DTNBP1 ANKRD13B PRKAG2-AS1 AATK PDE1C LINC00665 AGPAT1 AC020558.2 ENSG00000290989 SYNGAP1 AP006623.1 HERC2P2 PCOLCE BET1L AC016596.1 PTPN22 HSD11B2 AC010542.5 HNRNPA3P5 LIMS2 ENTPD3 CNBD2 CDK11A ENSG00000288937 ENSG00000287593 AC067930.5 MT-ATP6 ENSG00000286067 COL1A1 AC084036.1 RNVU1-25 SUPT4H1 AL645933.2 C10orf67 ZNF606-AS1 PRR16 ZFTRAF1 CCDC124 MT-ND4L ENSG00000290999 AC233699.1 NCOR2 LINC01121 RPL21P16 ZNF257 AC245041.1 USP14 AC007485.2 GATA6 RFXAP F8A1 EIF1 ATP2C2-AS1 RPS28 RPL18AP3 FABP5 LINC02777 MIR3176 BCHE TONSL FOXD4L1 AC022154.1 AC092143.3 SLC25A35 AC011337.1 LCT-AS1 SAMSN1 ENSG00000286104 RAB3A FUS RNF39 RBFADN AC005479.1 RGS4 PKD1P5 LINC02688 RPS3AP26 ENSG00000291068 CYP1A1 MAN1B1-DT NAA80 UPF3A ENSG00000291211 DLGAP4 AC007040.2 ENSG00000289074 AC018553.1 ENSG00000289043 PI4KAP1 ENSG00000289207 GLIS2 SAMD12-AS1 DBH ARL2BP AC090809.1 RHEBP2 MTND2P28 ENHO SMG1P2 ENSG00000286451 TCF4 LILRA6 MT-ND2 LLPH-DT TNFSF12 NOP53-AS1 LOC107984685 ALPP GPR132 PRRX1 MRPS24 FAM229A ARHGEF16 DUBR AP005018.2 AC068025.1 BMP6 LAMB3 PCAT1 BRD4 ENSG00000288772 ENSG00000289850 S100Z FTH1 NAIPP2 HOXB8 ZFP36 LINC02964 PXN CHAMP1 ENTREP3 MEIS1 PCBP1 LRRC2 MAGEC1 RPL17P39 CCDC188 LINC00960 AC147067.1 PDGFD FST VWCE RPS27AP5 AC034236.1 SIX2 AL450124.1 UBALD1 MAP1LC3B THY1 GTF2IP1 C5orf46 UCA1 RPL38P4 AC104695.2 CD177 DGAT1 BOLA2-SMG1P6 LINC01619 RPL7P9 PRRT1 ZNF414 LINC00842 PHGDH SLC29A3 CSNK2B OLR1 HCFC1R1 SLC25A34 EHD2 LYPD3 NELFCD LOC100130357 ENSG00000290018 GABRE NPIPB5 GS1-204I12.4 LINC01291 CCNK ESRP2 WNK4 SLC39A13-AS1 LRRC61 AL451042.1 AGAP4 KDM6B MARCHF4 CNFN LOC124900584 CILP2 TYMS PLCXD3 PPDPF SLC44A5 AL353622.1 AC245041.2 RPL17 MXRA5 RAPSN SDR39U1 LAMA1 MT-TC SETBP1 TINAGL1 ACTRT3 AP001453.3 GRIN2A TBC1D3L MYG1 LINC02783 FAM230C YJEFN3 ARSA ENSG00000286129 WASH9P OIP5 NELL2 POU3F2 AL132989.1 SPNS1 SNRPGP2 MT-ND6 AC020978.9 BGN ENSG00000287168 LINC00997 SNORD62B RPS28P7 CNKSR2 PAIP1P2 MORF4L1P1 AP000525.1 BEND4 AL162258.1 ELL3 RHBDL1 ENSG00000286324 MMP1 MAGI2-AS3 NCOA6 LRRC36 TNFRSF25 MT-RNR1 RHOXF1-AS1 ZFP91-CNTF HAGLR LINC01409 UCP2 RERE SRRM1 PCSK1N FBRS AMTN DOCK6 PCDHB6 AC245297.3 AL627309.6 FAM83A ENSG00000286001 ENSG00000287562 PPARD*

		ARHGAP20 LOC124901333 PTPN23 EGR4 AMPH PLBD2 DBH-AS1 CST4 VAC14 HNRNPA3P6 HEBP1 PPP1R13L PARP15 SLC39A4 MDC1 EGFL8 FNDC4 ENSG00000286614 ALOXE3 HNRNPD GAREM1 EBI3 AC137630.5 ZNF114 TRMT1 MMP3 EIF2B4 EIF5B RPL41P5 MT-RNR2 AC022400.7 GATA3 AL713998.1 PPP1R14B-AS1 TAS2R5 THAP12P8 ESAM RIPOR1 NPIPB15 LOC114841035 WASH7P GSTM1 TMEM276 APBB3 HNRNPA2B1 ENSG00000290569 YWHAZP10 PKD1P6-NPIPP1 GPRC5D MT-TS2 PACS1 ZFP36L1 AC010809.2 FXYD5 TRIM66 AC104958.2 ECI2-DT MIR1254-1 AGAP6 EZRP1 RPS3A SLC22A15 AC090004.1 ZSWIM9 SORCS3 RPS15P4 AC006058.1 MIR99AHG SRRM5 AC010422.8 ATP6V0C SKI CAPN11 RPL18A CCDC17 SP140 EVI2B MRPS30-DT TRPV4 DUXAP9 ZNF589 NRGN GPR35 BCKDHA ENSG00000291208 MAP2K7 CCDC9B MT-TQ AL034417.4 LINC00452 SP2 ENSG00000293594 LINC00370 MTFP1 NT5DC2 LINC01293 ACTMAP IKZF4 HERC2P9 MAPK13 FOXF2-DT GCKR MT-TH LOC107985911 SYT11 FZD8 RNVU1-24 ZNF426-DT RBM38-AS1 RPL9 GALNT14 LINC01089 LINC00910 ANKLE1 MT-ND5 NCAL1 DDX11L2 AC009237.6 SLC29A1 SMIM2-AS1 AC010336.2 MED15 SETD1B TFAP2A RHOJ MT-CO1 LINC00659 IL1RN ODC1 CROCCP2 CYSRT1 CDK11B ENSG00000288823 NRBF2P6 CAMTA2 ENO3 AC016405.3 RPL29P26 DNM1P35 ENSG00000288838 ARPC1AP4 NPIPB4 FBXL16 FNDC10 PPIAP31 EIF4BP6 CCDC69 LNCOG MNT ATP6V1G2 TOMM5 SMG7 ARHGAP27P1-BPTFP1-KPNA2P3 SH3BGRL3 LY6G5B ENSG00000292994 MIR570 SETD1A LOX SMPD4BP LINC02392 CCN1 GRIN3B AL110292.1 MSLNL SLFNL1-AS1 C2orf15 AC245140.3 PTOV1-AS2 CEACAM19 PCOTH AC004233.3 KIRREL3 EVA1A PDLIM1P4 NPIPB6 FKBP1C PPP1R11 ENSG00000288913 AC090673.2 GATA2 RABL2B NRXN3 TAF1C STXBP1 ZMIZ2 AC006058.4 DMD LINC02263 CTDNEP1 RPP21 HMGB1P10 PRKACA CDH19 CCBE1 ZKSCAN8P1 ZNG1DP RPSAP9 THBS1-IT1 CLDN1 MROH6 FOSB DTX2P1 CLCA4-AS1 RPL5P12 UPF3B HLA-DOB AGAP13P ENSG00000287250 LCAT POM121C MT-TR DDX39B TMED6 RNY4P10 AC009237.9 CNOT3 KCNIP2 TTC9B CRTC1 TAF15 SORBS3 ADM-DT KRT8P3 UCP3 ZNF703 R3HDM1 MT-TD AC005077.4 RPS2P5 MLLT11 C13orf46 CGB8 TMLHE MAP1LC3B2 ARHGEF19 BICRA TBX6 AL645608.8 LDOC1 NRK RPL18AP6 PEAR1 CDA S1PR1-DT AL627309.7 CENPT LINC01001 LOC101927888 SAMD4B CCDC78 MT-TV ATXN2L C20orf96 SCAF4 NPIPB12 AC006254.1 LOC124902694 RPS16 ENSG00000289810 AC008735.2 AP001029.2 SYT15B SRRM2 COL1A2 DTNA ENSG00000290728 PBX2 AL049757.1 ABTB1 CCDC183-AS1 ATP13A3-DT CYLD-AS1 ATP6V1B1-AS1 NR1D1 PDE4A GKN2 AOC2 AC095050.1 MT-TL1 AC132872.1 ADGRE1 MYADM DSP-AS1 PDE7B-AS1 MFSD3 GNRH2 AFF3 ESM1 NPDC1 MIR137HG VAMP2 SPINK1 RTL8A MEF2D RPL13AP25 LYRM4-AS1 AC019077.1 SOX7 ENSG00000286905 LINC00839 CHRDL1 EPB41L4A HSPB8 MTX1LP ATP1A3 LINC02474 MATR3 CYCSP6 UTP4 HNRNPA1P16 SERPINB5 AC245060.6 ZBED3 FOXQ1 KCNAB3 FHL3 NRM GABARAP SNHG4 FBXL6 SLC9A5 DUSP7 GPR3 RPL23AP42 DYNC1LI2-DT ZNF516-DT NUS1P1 AL122023.1 AC253536.6 RPL10AP6 TOP3B AL390037.1 PDLIM7 TCHH RPS18P5 DUXAP8 SCUBE3-AS1 KCND1 RNF208 PLK3 CMIP GTF3C2-AS2 MIR3682 UCHL1 AC018653.3 LINC00623 AC134312.5 TEKT4P2 PAQR6 AC018521.6 MT-TN AL356481.3 AC244453.3 ENSG00000293320 RNASEK TGFBR3L LINC02303 GREP1 LIF-AS2 ZC3H3 LOC105369165 WIZ PAK6 SWINGN EDC3 LOC124909397 PTHLH GNG5 LOC155060 TNFSF15 TMEM35B NCF2 SEC31B HUS1B GNG10 COX6A1P2 ODC1-DT CACNG7 SYPL2 CXXC1 MYOSLID NHSL3 ZNF716 TMEM217 CMC4 WASH8P PINLYP ENSG00000286974 COX7B2 MIR647 LAG3 PRAF2 HAL ENSG00000286966 RCAN2 LINC01356 DHCR24-DT AC093218.1 SERPINB7 TECR NEAT1 RAPGEFL1 CST1 TP53INP2 BCL9L CTXN1 GJB3 SNX10 PRICKLE2-DT FOXO6 RASEF MT-CYB MAMSTR EGR1 MTND4P12 ECM1 DUSP26 GBA1LP DLX4 MIR3685 NLGN2 AGAP9 MT-TI AC125437.1 CC2D1A PLAGL1 CTF1 SCNN1D AL365184.1 PROSER1 LOC102724594 MT-ATP8 SERINC2 FAM222B AC010319.4 RNF5P1 VPS9D1-AS1 NPIPB11 TENM1 ILK SERPINB2 AOC3 CCDC120 LINC01322 AC040160.1 NPRL3 AC087741.1 RPL7 FLJ45513 MT-TL2 SOX12 FURIN ARL10 LTB4R2 ZYX AC090948.3 SYT15 KCNE5 SF3A2 ENSG00000291215 GOLGA8A PRKCZ-AS1 PCDHB2 RBM20 SDHAP1 ASIC1 TECRP1 MYCT1 CPT1B ENSG00000291147 ATN1 ST3GAL6 RPS26 TPH1 HSF4 ZNF296 DIPK1B MT-ND4 MT-CO3 PLD5 WASHC1 CCR5AS NPIPA1 CRLF1 DUSP5P1 MAGEA3-DT

		CTH MT-TW CDK5R2 CRTC2 TIE1 GTF2H4 KCNK1 SCAMP5 GSTT2 MC1R DCAF8L2 AC073575.2 DUX4L50 LOC124902477 PCGF2 SLC52A3 TMSB4XP4 LENG8 AC018529.2 LINC02327 PRSS3 DRGX TUBB3 ASB16 NSUN5P1 ANO7 CAMK2N1 GREM1 NFIC AC105020.1 AC103746.1 NPIPB3 FOXD2-AS1 ABLIM2 TNFRSF13C HOXD1 HNRNPA3P12 ALDOA MT-ND1 SYT15-AS1 HNRNPH3 MYO7B PRR14 BMPER PSMC1 SLC7A8 AC135048.4 ZRANB2 S100A11 ZNF521 FOXA3 EIF5AL1 PDXDC2P-NPIPB14P AL353150.1 PPT2-EGFL8 DUXAP10 HMGB1P6 REELD1 SEMA6A STAG3 ZNF584-DT CCNY-AS1 MTATP6P1 TMPRSS15 LTB4R ISYNA1 ENSG00000286284 RAB11B MT-TE LY6G5C LOC105370409 MIR6772 MAGEC2 ENSG00000291201 FGGY-DT SRCIN1 LOC728485 AC092118.2 AC093515.1 PHACTR4 COL3A1 FOXP4 ENSG00000286366 PPT2 DNAH10OS AC107983.1 LSP1P5 AC067930.6 KCNK2 CGB7 LOC100505851 KRT14 PCBP2P2 ENSG00000293106 RPS28P4 CCDC154 ST3GAL6-AS1 HMGN5 LINC02604 PDCD4-AS1 NPFF AC129510.1 MYD88 ZNF580 DVL3 RNA5SP317 AC104453.1 RPS27P8 LINC02918 MAOB H2BP1 ONECUT1 TMSB15B ADGRB3 AC090109.1 ENSG00000287238 AARSD1 AC020917.4 RPL41P1 BMS1P4 IGF2 RPL21 ENSG00000286331 AL645608.6 SNRPA1 ID4 ADAMTS1 BCL2L2-PABPN1 PLXDC2 MCOLN1 RIN3 NFIX ENSG00000286017 GAL3ST2 AL662899.2 MBD6 PSORS1C1 BCORL1 MHENCR TPP1 PABPC1L DHPS GSTT2B ANXA8L1 S1PR1 TRAPPC1 HCN2 CKLF LINC01518 PLEKHN1 LRRC8C-DT GGT7 KIFC2 DOK3 ENSG00000289365 GDPD3 ENSG00000289981 FNDC5 AC026691.1 AC006111.2 ENSG00000290032 TMEM190 FAM27C AC009303.4 AMH EPHA6 GNMT TMEM198B FTH1P20 DIAPH1 MRPL2 MT-ND3 RPL10P16 TRBV12-4 SNRPGP15 MAGEA6 NOB1 RAB3IL1 PLAGL2 SLC9A1 LINC02009 C1orf74 SRD5A3-AS1 ENSG00000290126 LINC00926 GPR146 U2AF1L4 USB1 AL031673.1 EIF3C PAX8 AC090409.1 TRAPPC12-AS1 LOC100129434 MTCO1P12 LINC01002 KHSRP ANPEP KRTAP2-3 PANX2 ANGPTL6 MIR1244-4 LOXL3 LOC101927745 FAT3 SLFNL1 LINC02878 NPIPB13 PGM5P3-AS1 TMEM249 TBCAP1 GLIDR RAB13 PYY2 AKT1S1 ENSG00000289985 LINC00707 ENSG00000288752 WDR97 LBX2-AS1 AC107375.1 AC099850.1 NSRP1 ARID3B MIR4477B TMEM198 C6orf136 DMAP1 MKRN3 TUBG2 ENSG00000289971 RNASEH2A LIN37 AC097451.1 RHOQ-AS1 AC132812.1 WASH4P PORCN ALG3 MRPL20-DT INKA1 GNRHR2 ADGRL1-AS1 AL356535.1 CIZ1 GPR137 MT-TK USHBP1 SLITRK1 ALS2CL C6orf132 BTBD19 KRT8P45 SLC9A3-AS1 AC009831.1 RANGRF KIAA0319 CHD5 AL031846.2 MT-CO2 ARHGDIA FBXW10B HNRNPA3 RPSAP47 Y_RNA CHERP ENSG00000290832 FAM88F PKDCC RTKN RPL5P34 MUC20-OT1 PABPC1 PITPNA-AS1 AL022393.1 LOC729998 LINC01446 GPX3 RPLP0P9 ENSG00000287222 GFOD3P C11orf98 MIF AL049629.2 C4BPB ATP2A1-AS1 SPDYE12 ENSG00000288810 CGN EIF1AXP1 MMP25-AS1 GATA6-AS1 SYVN1 ENSG00000290823 ADH6 GPRC5B GNG13 FOXD4 GSK3A STIMATE-MUSTN1 ENSG00000287910 GPR87 ACTA2 AL365205.1 ZNF385A STX16 TMEM119 INHBA-AS1 HNRNPA1P33 AC060780.1 EPS15L1 DDX12P AC067751.1 RELN GATAD2B VDAC1P8 AC084018.1 AC010207.1 ADAMTS5 ATIC LINC01224 NPL ADAMTSL3 POM121 PRRT2 ESPN SULT1B1 PAM16 TRNP1 AC016924.1 ANP32B AC242376.2 LOC124907726 AC008687.6 GLI4 SLC38A7 GABRA3 CYP2J2 AC233266.2 TRHDE-AS1 YY2 CALML6 G6PC3 SFRP1 DAPK1 AC008870.2 STAG3L5P-PVRIG2P-PILRB FKBP1A FOSL1 AC011448.1 MAT2A MAL2 C2orf74 AC068985.1 RNF44 H3P6 NPTXR DPYSL3 AC012676.1 TFAP2A-AS2 NUSAP1 ABHD16A HLX FLRT1 AC145098.2 FTH1P11 VSTM1 GPR15 RAB7A CHKB-CPT1B PCIF1 ENSG00000291100 RPS27 LOC124904009

Table S6. GO associations with biological processes of 9 upregulated genes common for MB and Mel Z cells forming VM phenotype.

GO.ID	Description	padj	Genes
GO:0001568	blood vessel development	7.95882699585203e-11	*PDCL3,BAK1,NGFR,HMOX1,ADM,SPHK1,PDE2A,SERPINE1,CCN3*
GO:0001944	vasculature development	1.1446992774816197e-10	*PDCL3,BAK1,NGFR,HMOX1,ADM,SPHK1,PDE2A,SERPINE1,C*

			CN3
GO:0072359	circulatory system development	4.542051208666478e-9	*PDCL3,BAK1,NGFR,HMOX1,ADM,SPHK1,PDE2A,SERPINE1,CCN3*
GO:0048514	blood vessel morphogenesis	6.453829580008994e-9	*PDCL3,BAK1,NGFR,HMOX1,ADM,SPHK1,SERPINE1,CCN3*
GO:0035239	tube morphogenesis	8.714904047216382e-8	*PDCL3,BAK1,NGFR,HMOX1,ADM,SPHK1,SERPINE1,CCN3*
GO:0001525	angiogenesis	2.969092680768523e-7	*PDCL3,NGFR,HMOX1,ADM,SPHK1,SERPINE1,CCN3*
GO:0035295	tube development	5.262592688842451e-7	*PDCL3,BAK1,NGFR,HMOX1,ADM,SPHK1,SERPINE1,CCN3*
GO:0045766	positive regulation of angiogenesis	0.000003940661311141707	*PDCL3,HMOX1,ADM,SPHK1,SERPINE1*
GO:1904018	positive regulation of vasculature development	0.000004312481692801628 5	*PDCL3,HMOX1,ADM,SPHK1,SERPINE1*
GO:0045765	regulation of angiogenesis	0.0000679284344718263	*PDCL3,HMOX1,ADM,SPHK1,SERPINE1*
GO:0048646	anatomical structure formation involved in morphogenesis	0.00006968222009495181	*PDCL3,NGFR,HMOX1,ADM,SPHK1,SERPINE1,CCN3*
GO:1901342	regulation of vasculature development	0.0000751540359774686	*PDCL3,HMOX1,ADM,SPHK1,SERPINE1*
GO:0009968	negative regulation of signal transduction	0.00013966332961648961	*BAK1,NGFR,HMOX1,ADM,PDE2A,SERPINE1,CCN3*
GO:0051094	positive regulation of developmental process	0.00015249202530144988	*PDCL3,NGFR,HMOX1,ADM,SPHK1,SERPINE1,CCN3*
GO:0023057	negative regulation of signaling	0.00024076258118318477	*BAK1,NGFR,HMOX1,ADM,PDE2A,SERPINE1,CCN3*
GO:0010648	negative regulation of cell communication	0.0002419205701174297	*BAK1,NGFR,HMOX1,ADM,PDE2A,SERPINE1,CCN3*
GO:0022603	regulation of anatomical structure morphogenesis	0.00031550206643804035	*PDCL3,NGFR,HMOX1,ADM,SPHK1,SERPINE1*
GO:0006954	inflammatory response	0.0003917598530670225	*HMOX1,ADM,SPHK1,PDE2A,SERPINE1,CCN3*
GO:0048731	system development	0.00040534090283263907	*PDCL3,BAK1,NGFR,HMOX1,ADM,SPHK1,PDE2A,SERPINE1,CCN3*
GO:0050673	epithelial cell proliferation	0.0005183169758443531	*PDCL3,BAK1,NGFR,HMOX1,CCN3*
GO:0009653	anatomical structure morphogenesis	0.0006743340413835693	*PDCL3,BAK1,NGFR,HMOX1,ADM,SPHK1,SERPINE1,CCN3*
GO:1901700	response to oxygen-containing compound	0.0006802698160655087	*BAK1,NGFR,HMOX1,ADM,SPHK1,PDE2A,SERPINE1*
GO:0048585	negative regulation of response to stimulus	0.0007116407526171604	*BAK1,NGFR,HMOX1,ADM,PDE2A,SERPINE1,CCN3*
GO:0042127	regulation of cell population proliferation	0.0007263019686279983	*PDCL3,BAK1,NGFR,HMOX1,ADM,SPHK1,CCN3*
GO:0007275	multicellular organism development	0.001620511660571507	*PDCL3,BAK1,NGFR,HMOX1,ADM,SPHK1,PDE2A,SERPINE1,CCN3*
GO:000	regulation of signal	0.0016261754460	*BAK1,NGFR,HMOX1,ADM,SPHK*

9966	transduction	743288	*1,PDE2A,SERPINE1,CCN3*
GO:0006915	apoptotic process	0.0017953761333718763	*PDCL3,BAK1,NGFR,HMOX1,ADM,SPHK1,SERPINE1*
GO:0012501	programmed cell death	0.0022455567777920037	*PDCL3,BAK1,NGFR,HMOX1,ADM,SPHK1,SERPINE1*
GO:0008219	cell death	0.002276615211737289	*PDCL3,BAK1,NGFR,HMOX1,ADM,SPHK1,SERPINE1*
GO:0008283	cell population proliferation	0.002470733262363122	*PDCL3,BAK1,NGFR,HMOX1,ADM,SPHK1,CCN3*
GO:0023051	regulation of signaling	0.004750484647363641	*BAK1,NGFR,HMOX1,ADM,SPHK1,PDE2A,SERPINE1,CCN3*
GO:0030336	negative regulation of cell migration	0.004829328938831673	*NGFR,HMOX1,SERPINE1,CCN3*
GO:0010646	regulation of cell communication	0.004836777972873242	*BAK1,NGFR,HMOX1,ADM,SPHK1,PDE2A,SERPINE1,CCN3*
GO:0008285	negative regulation of cell population proliferation	0.005266343543349691	*BAK1,NGFR,HMOX1,ADM,CCN3*
GO:2000146	negative regulation of cell motility	0.005966102262703138	*NGFR,HMOX1,SERPINE1,CCN3*
GO:0009893	positive regulation of metabolic process	0.006182604177311404	*PDCL3,BAK1,NGFR,HMOX1,ADM,SPHK1,PDE2A,SERPINE1*
GO:0048584	positive regulation of response to stimulus	0.006664598648576019	*BAK1,NGFR,HMOX1,SPHK1,PDE2A,SERPINE1,CCN3*
GO:2000026	regulation of multicellular organismal development	0.00727945610310891	*PDCL3,NGFR,HMOX1,ADM,SPHK1,SERPINE1*
GO:0040013	negative regulation of locomotion	0.007470099726977958	*NGFR,HMOX1,SERPINE1,CCN3*
GO:0048523	negative regulation of cellular process	0.007812563558852651	*PDCL3,BAK1,NGFR,HMOX1,ADM,SPHK1,PDE2A,SERPINE1,CCN3*
GO:0042475	odontogenesis of dentin-containing tooth	0.008177123682759099	*NGFR,ADM,SERPINE1*
GO:0009605	response to external stimulus	0.00847562266621464	*BAK1,NGFR,ADM,SPHK1,PDE2A,SERPINE1,CCN3*
GO:0042981	regulation of apoptotic process	0.008697377542453498	*BAK1,NGFR,HMOX1,ADM,SPHK1,SERPINE1*
GO:0042542	response to hydrogen peroxide	0.00897957272504422	*BAK1,HMOX1,SPHK1*
GO:0050793	regulation of developmental process	0.009527221389149833	*PDCL3,NGFR,HMOX1,ADM,SPHK1,SERPINE1,CCN3*
GO:0043067	regulation of programmed cell death	0.010334842456563503	*BAK1,NGFR,HMOX1,ADM,SPHK1,SERPINE1*
GO:0009889	regulation of biosynthetic process	0.010373154927466667	*PDCL3,BAK1,NGFR,HMOX1,ADM,SPHK1,PDE2A,SERPINE1,CCN3*
GO:0048519	negative regulation of biological process	0.010782211431566392	*PDCL3,BAK1,NGFR,HMOX1,ADM,SPHK1,PDE2A,SERPINE1,CCN3*
GO:0042221	response to chemical	0.011847709633827589	*BAK1,NGFR,HMOX1,ADM,SPHK1,PDE2A,SERPINE1,CCN3*
GO:0048522	positive regulation of cellular process	0.012301111355069706	*PDCL3,BAK1,NGFR,HMOX1,ADM,SPHK1,PDE2A,SERPINE1,CCN3*

GO:2001233	regulation of apoptotic signaling pathway	0.013684262969026017	*BAK1,NGFR,HMOX1,SERPINE1*
GO:0048856	anatomical structure development	0.013819405829614028	*PDCL3,BAK1,NGFR,HMOX1,ADM,SPHK1,PDE2A,SERPINE1,CCN3*
GO:0048583	regulation of response to stimulus	0.013986770199725714	*BAK1,NGFR,HMOX1,ADM,SPHK1,PDE2A,SERPINE1,CCN3*
GO:0050727	regulation of inflammatory response	0.015159534427052966	*SPHK1,PDE2A,SERPINE1,CCN3*
GO:0009891	positive regulation of biosynthetic process	0.017858389502270904	*PDCL3,NGFR,HMOX1,ADM,SPHK1,PDE2A,SERPINE1*
GO:0051240	positive regulation of multicellular organismal process	0.01863250552647818	*PDCL3,NGFR,HMOX1,ADM,SPHK1,SERPINE1*
GO:0048518	positive regulation of biological process	0.020457845794752114	*PDCL3,BAK1,NGFR,HMOX1,ADM,SPHK1,PDE2A,SERPINE1,CCN3*
GO:0008284	positive regulation of cell population proliferation	0.022868003552780902	*PDCL3,NGFR,HMOX1,ADM,SPHK1*
GO:0001936	regulation of endothelial cell proliferation	0.02348700635782713	*PDCL3,NGFR,HMOX1*
GO:0030334	regulation of cell migration	0.023574536509332705	*NGFR,HMOX1,SPHK1,SERPINE1,CCN3*
GO:0042476	odontogenesis	0.024011631512934013	*NGFR,ADM,SERPINE1*
GO:1903587	regulation of blood vessel endothelial cell proliferation involved in sprouting angiogenesis	0.02452957335616609	*NGFR,HMOX1*
GO:0032502	developmental process	0.030709012016642456	*PDCL3,BAK1,NGFR,HMOX1,ADM,SPHK1,PDE2A,SERPINE1,CCN3*
GO:0043116	negative regulation of vascular permeability	0.030828638994871593	*ADM,PDE2A*
GO:2000145	regulation of cell motility	0.03192929320061722	*NGFR,HMOX1,SPHK1,SERPINE1,CCN3*
GO:0051239	regulation of multicellular organismal process	0.03322049002781654	*PDCL3,NGFR,HMOX1,ADM,SPHK1,SERPINE1,CCN3*
GO:0035556	intracellular signal transduction	0.033906828879966745	*BAK1,NGFR,HMOX1,ADM,SPHK1,PDE2A,CCN3*
GO:0006952	defense response	0.03433180911508897	*HMOX1,ADM,SPHK1,PDE2A,SERPINE1,CCN3*
GO:0001935	endothelial cell proliferation	0.03759007508719031	*PDCL3,NGFR,HMOX1*
GO:0040012	regulation of locomotion	0.03932935098020491	*NGFR,HMOX1,SPHK1,SERPINE1,CCN3*
GO:0141124	intracellular signaling cassette	0.041124301043652396	*NGFR,HMOX1,ADM,SPHK1,PDE2A,CCN3*
GO:0048513	animal organ development	0.044266034062238196	*BAK1,NGFR,ADM,SPHK1,PDE2A,SERPINE1,CCN3*
GO:000	blood vessel	0.0497032971697	*NGFR,HMOX1*

2043	endothelial cell proliferation involved in sprouting angiogenesis	41555	
GO:0043065	positive regulation of apoptotic process	0.04979064583686417	*BAK1,NGFR,HMOX1,ADM*

Table S7. The 98 common downregulated genes common for MB and Mel Z cells forming VM phenotype are simultaneously regulated by 980 different transcription factors. Data were obtained by a search of corresponding genes in Enrichr Submissions TF-Gene Cooccurrence (https://maayanlab.cloud/Enrichr/enrich#, accessed on 24 December 2025).

Term	Overlap	Adjusted P-value	Genes
ZNF396	15/299	1.4527240458503337E-8	SLFN5;PCDHGA5;IKZF2;ESR1;C5;ZMAT1;TRIM2;DLC1;DDIT4;ZNF546;SPRY1;SRGAP3;ALPK1;ZNF610;JDP2
ZNF717	15/299	1.4527240458503337E-8	PCDHGA5;SEMA3D;ZBTB20;FRMD4B;SYNE2;ASPM;ZMAT1;PLCB4;ERBB4;ZNF704;DLC1;DDIT4;SLITRK6;TNS3;EXPH5
ZNF618	13/299	1.3644833614291123E-6	SLFN5;HIP1;MX1;ZBTB20;SYNE2;CLMN;ZNF704;TRIM2;DLC1;DDIT4;SRGAP3;TNS3;MAP2K6
NR3C2	12/299	6.09610604200636E-6	AR;PLCB4;ERBB4;TRIM2;DLC1;DDIT4;ZBTB20;ETV1;SRGAP3;TNS3;ESR1;RAPGEF4
RAPGEF5	12/299	6.09610604200636E-6	ST6GAL1;HIP1;PLCB4;TRIM2;DLC1;DDIT4;ZBTB20;ETV1;SPRY1;PRICKLE1;TNS3;SYNE2
ZNF354C	12/299	6.09610604200636E-6	AR;ST6GAL1;PCDHGA5;PLCB4;NR5A2;MEGF10;ZNF704;TRIM2;DLC1;ZBTB20;ETV1;ESR1
ZNF599	12/299	6.09610604200636E-6	SLFN5;PCDHGA5;GNG2;ZNF704;DLC1;DDIT4;VASH2;ZBTB20;ZNF546;FRMD4B;SRGAP3;ZNF610
PROX2	11/299	2.700702748037383E-5	ST6GAL1;PCDHGA5;NR5A2;CLMN;DLC1;VASH2;ZBTB20;ESR1;SYNE2;MAP2K6;RAPGEF4
ZNF214	11/299	2.700702748037383E-5	AR;ASPM;PCDHGA5;ERBB4;DLC1;DDIT4;ETV1;SPRY1;TNS3;ESR1;SYNE2
ZNF221	11/299	2.700702748037383E-5	ASPM;PCDHGA5;SEMA3D;DLC1;DDIT4;ZNF546;PTPRJ;ETV1;ZNF610;SYNE2;EXPH5
ZNF429	11/299	2.700702748037383E-5	SLFN5;ST6GAL1;ZMAT1;HIP1;CLMN;DLC1;DDIT4;ZBTB20;IKZF2;ZNF610;MAP2K6
ZNF546	11/299	2.700702748037383E-5	SLFN5;ZMAT1;PLCB4;ZNF704;TRIM2;DLC1;DDIT4;ZBTB20;IKZF2;ESR1;SYNE2
ZNF610	11/299	2.700702748037383E-5	AR;SLFN5;ZMAT1;PCDHGA5;PLCB4;TRIM2;DLC1;DDIT4;MX1;ZBTB20;PRICKLE1
ZNF704	11/299	2.700702748037383E-5	ST6GAL1;CLMN;TRIM2;DLC1;DDIT4;ZBTB20;ETV1;SPRY1;FRMD4B;PRICKLE1;TNS3
ZNF709	11/299	2.700702748037383E-5	PCDHGA5;GNG2;ZNF704;DLC1;DDIT4;ZBTB20;ZNF546;IKZF2;ZNF610;ESR1;SYNE2
CPSF4L	10/299	8.922860501425652E-5	RBM47;NR5A2;CLMN;TRIM2;DLC1;DDIT4;ETV1;TNS3;PDE9A;MAP2K6
DPF3	10/299	8.922860501425652E-5	PCDHGA5;CLMN;DLC1;DDIT4;ZBTB20;FRMD4B;SRGAP3;PRICKLE1;TNS3;ESR1
ESRRG	10/299	8.922860501425652E-5	AR;PCDHGA5;PLCB4;NR5A2;ERBB4;DLC1;ZBTB20;ETV1;ESR1;RAPGEF4
GTF2IRD2	10/299	8.92286050142	SLFN5;ZMAT1;PCDHGA5;TRIM2;DLC1;DDIT4

B		5652E-5	;ZBTB20;SPRY1;SRGAP3;SYNE2
KLF12	10/299	8.922860501425652E-5	PLCB4;GNG2;TRIM2;DLC1;DDIT4;ZBTB20;ETV1;PRICKLE1;IKZF2;TNS3
PRDM5	10/299	8.922860501425652E-5	PCDHGA5;PLCB4;SEMA3D;DLC1;ZBTB20;ETV1;LAMB1;SPRY1;FRMD4B;TNS3
PROX1	10/299	8.922860501425652E-5	NR5A2;ERBB4;CLMN;TRIM2;DLC1;DDIT4;ZBTB20;ETV1;FRMD4B;TNS3
RARB	10/299	8.922860501425652E-5	PLCB4;ERBB4;ALDH1A2;DLC1;ZBTB20;ETV1;SPRY1;PRICKLE1;TNS3;ESR1
RFX3	10/299	8.922860501425652E-5	ST6GAL1;PLCB4;GNG2;TRIM2;ZBTB20;ETV1;SRGAP3;IKZF2;TNS3;SYNE2
SCML4	10/299	8.922860501425652E-5	SLFN5;ST6GAL1;GNG2;ST8SIA1;DLC1;DDIT4;ZBTB20;ESR1;SYNE2;RAPGEF4
SOX6	10/299	8.922860501425652E-5	PCDHGA5;SEMA3D;ERBB4;TRIM2;DLC1;ZBTB20;ETV1;FRMD4B;TNS3;SYNE2
ZFP2	10/299	8.922860501425652E-5	ZMAT1;PCDHGA5;TRIM2;DLC1;DDIT4;ZBTB20;PRICKLE1;IKZF2;MAP2K6;RAPGEF4
ZNF135	10/299	8.922860501425652E-5	AR;ZMAT1;PCDHGA5;PLCB4;ZNF704;DLC1;MX1;PRICKLE1;ZNF610;TNS3
ZNF235	10/299	8.922860501425652E-5	ZMAT1;PCDHGA5;TRIM2;DLC1;DDIT4;ZBTB20;ETV1;SPRY1;IKZF2;MAP2K6
ZNF343	10/299	8.922860501425652E-5	ST6GAL1;HIP1;PCDHGA5;PLCB4;DLC1;DDIT4;MX1;PTPRJ;TNS3;SYNE2
ZNF385B	10/299	8.922860501425652E-5	PCDHGA5;ERBB4;ALDH1A2;DLC1;ZBTB20;ETV1;PRICKLE1;TNS3;ESR1;RAPGEF4
ZNF425	10/299	8.922860501425652E-5	SLFN5;BEX2;TRIM2;DLC1;DDIT4;MX1;SRGAP3;PRICKLE1;TNS3;JDP2
ZNF491	10/299	8.922860501425652E-5	SLFN5;PCDHGA5;ZNF704;DLC1;DDIT4;ZBTB20;ZNF546;SRGAP3;IKZF2;ZNF610
ZNF608	10/299	8.922860501425652E-5	ZNF704;TRIM2;DLC1;DDIT4;ZBTB20;ETV1;SPRY1;PRICKLE1;TNS3;MAP2K6
ZNF619	10/299	8.922860501425652E-5	ASPM;SLFN5;HIP1;PCDHGA5;DLC1;DDIT4;PTPRJ;IKZF2;TNS3;SYNE2
ZNF660	10/299	8.922860501425652E-5	AR;ZMAT1;HIP1;PCDHGA5;GNG2;CLMN;ZNF704;DLC1;ZBTB20;SRGAP3
ZNF671	10/299	8.922860501425652E-5	SLFN5;ST6GAL1;PCDHGA5;GNG2;TRIM2;DLC1;DDIT4;MX1;SRGAP3;ZNF610
ZNF774	10/299	8.922860501425652E-5	SLFN5;HIP1;PCDHGA5;ZNF704;TRIM2;DLC1;DDIT4;ZBTB20;SRGAP3;PRICKLE1
ZNF793	10/299	8.922860501425652E-5	BEX2;PCDHGA5;ZNF704;TRIM2;DLC1;MX1;ZNF546;PTPRJ;IKZF2;ZNF610
CTCFL	9/299	4.4743209966215125E-4	RBM47;NNMT;PCDHGA5;NR5A2;DLC1;DDIT4;ZBTB20;LAMB1;ESR1
DMRTA1	9/299	4.4743209966215125E-4	AR;SEMA3D;DLC1;DDIT4;SLITRK6;ETV1;SPRY1;PRICKLE1;TNS3
KIAA1549	9/299	4.4743209966215125E-4	HIP1;PLCB4;ZNF704;TRIM2;DLC1;ETV1;SRGAP3;TNS3;SYNE2
NR2E3	9/299	4.4743209966215125E-4	AR;PCDHGA5;NR5A2;DLC1;DDIT4;PTPRJ;ETV1;TNS3;ESR1
SATB2	9/299	4.4743209966215125E-4	PLCB4;NR5A2;DLC1;DDIT4;ZBTB20;ETV1;LAMB1;PRICKLE1;TNS3
SIM1	9/299	4.4743209966215125E-4	PCDHGA5;NR5A2;ERBB4;ALDH1A2;DLC1;ZBTB20;ETV1;TNS3;ESR1
SOX5	9/299	4.4743209966215125E-4	PLCB4;GNG2;ERBB4;TRIM2;DLC1;ZBTB20;ETV1;TNS3;ESR1
TOX	9/299	4.4743209966215125E-4	ST6GAL1;PLCB4;GNG2;DLC1;DDIT4;ZBTB20;ETV1;PRICKLE1;TNS3
ZMAT1	9/299	4.4743209966215125E-4	AR;SLFN5;CLMN;DLC1;DDIT4;ZBTB20;IKZF2;TNS3;SYNE2

ZNF19	9/299	4.47432099662 15125E-4	PCDHGA5;DLC1;DDIT4;MX1;SPRY1;SRGAP3; ZNF610;TNS3;EXPH5
ZNF20	9/299	4.47432099662 15125E-4	PCDHGA5;TRIM2;DLC1;DDIT4;MX1;ZBTB20; SPRY1;TNS3;PDE9A
ZNF311	9/299	4.47432099662 15125E-4	PCDHGA5;NR5A2;DLC1;DDIT4;MX1;SPRY1;Z NF610;TNS3;ESR1
ZNF391	9/299	4.47432099662 15125E-4	SLFN5;ST6GAL1;HIP1;ZNF704;TRIM2;DLC1 ;DDIT4;ETV1;SRGAP3
ZNF418	9/299	4.47432099662 15125E-4	PCDHGA5;ZNF704;DLC1;MX1;ZBTB20;SPRY1 ;ZNF610;TNS3;SYNE2
ZNF438	9/299	4.47432099662 15125E-4	SLFN5;RBM47;PCDHGA5;DLC1;DDIT4;ZBTB2 0;PTPRJ;ALPK1;TNS3
ZNF568	9/299	4.47432099662 15125E-4	SLFN5;PCDHGA5;PLCB4;TRIM2;DLC1;ZBTB2 0;ZNF610;TNS3;SYNE2
ZNF613	9/299	4.47432099662 15125E-4	AR;SLFN5;ST6GAL1;RBM47;DLC1;DDIT4;MX 1;ZBTB20;PDE9A
ZNF615	9/299	4.47432099662 15125E-4	ASPM;SLFN5;RBM47;DLC1;DDIT4;MX1;ZBTB 20;SPRY1;PRICKLE1
ZNF711	9/299	4.47432099662 15125E-4	ASPM;BEX2;PLCB4;GNG2;DLC1;DDIT4;VASH 2;ETV1;SYNE2
ZNF713	9/299	4.47432099662 15125E-4	NEURL1B;ZNF704;DLC1;DDIT4;MX1;ZBTB20 ;ETV1;TNS3;RAPGEF4
ZSCAN23	9/299	4.47432099662 15125E-4	AR;PCDHGB7;PCDHGA5;ZNF704;DLC1;ZBTB2 0;SPRY1;SRGAP3;ZNF610
ADAMTS17	8/299	0.00152334314 77595953	PCDHGA5;ALDH1A2;DLC1;ZBTB20;SRGAP3;P RICKLE1;TNS3;ESR1
BACH2	8/299	0.00152334314 77595953	ST6GAL1;DLC1;DDIT4;ZBTB20;ETV1;SPRY1 ;FRMD4B;TNS3
CHD7	8/299	0.00152334314 77595953	ST6GAL1;DLC1;DDIT4;ZBTB20;PTPRJ;FRMD 4B;TNS3;SYNE2
DZIP1	8/299	0.00152334314 77595953	GNG2;ST8SIA1;TRIM2;DLC1;DDIT4;ETV1;L AMB1;PRICKLE1
FMNL2	8/299	0.00152334314 77595953	ST6GAL1;DLC1;DDIT4;ZBTB20;PTPRJ;LAMB 1;FRMD4B;TNS3
HDX	8/299	0.00152334314 77595953	AR;SLFN5;PLCB4;DLC1;MX1;ZBTB20;ETV1; IKZF2
HNF4G	8/299	0.00152334314 77595953	AR;RBM47;NR5A2;TRIM2;DLC1;ZBTB20;ETV 1;ESR1
HOXA6	8/299	0.00152334314 77595953	PCDHGA5;NR5A2;ALDH1A2;DLC1;DDIT4;SPR Y1;TNS3;ESR1
IKZF2	8/299	0.00152334314 77595953	SLFN5;ST6GAL1;DLC1;DDIT4;ZBTB20;FRMD 4B;TNS3;SYNE2
JAZF1	8/299	0.00152334314 77595953	GNG2;TRIM2;DLC1;DDIT4;ZBTB20;PTPRJ;P RICKLE1;TNS3
MEIS2	8/299	0.00152334314 77595953	TRIM2;DLC1;DDIT4;ZBTB20;ETV1;LAMB1;S PRY1;PRICKLE1
MLLT3	8/299	0.00152334314 77595953	PLCB4;TRIM2;DLC1;DDIT4;ZBTB20;FRMD4B ;IKZF2;TNS3
NCOA1	8/299	0.00152334314 77595953	TRIM2;DLC1;DDIT4;ZBTB20;PTPRJ;TNS3;E SR1;SYNE2
NFIA	8/299	0.00152334314 77595953	TRIM2;DLC1;DDIT4;ZBTB20;ETV1;SPRY1;T NS3;SYNE2
NR5A2	8/299	0.00152334314 77595953	ERBB4;TRIM2;DLC1;DDIT4;ETV1;PRICKLE1 ;TNS3;ESR1
PBX1	8/299	0.00152334314 77595953	TRIM2;DLC1;DDIT4;ZBTB20;ETV1;PRICKLE 1;TNS3;SYNE2
POU6F1	8/299	0.00152334314 77595953	ST6GAL1;DLC1;DDIT4;ZBTB20;SRGAP3;PRI CKLE1;TNS3;JDP2
PRDM9	8/299	0.00152334314	PCDHGA5;DLC1;DDIT4;ETV1;TNS3;ESR1;SY

		77595953	NE2;ADAMTS20
SALL4	8/299	0.00152334314 77595953	ASPM;NR5A2;DLC1;DDIT4;KIF14;SPRY1;PRICKLE1;TNS3
SATB1	8/299	0.00152334314 77595953	ST6GAL1;GNG2;DLC1;DDIT4;ZBTB20;ETV1;TNS3;SYNE2
SPDEF	8/299	0.00152334314 77595953	RBM47;SLC2A10;TRIM2;DLC1;DDIT4;KIF14;ETV1;ESR1
SSH1	8/299	0.00152334314 77595953	SLFN5;HIP1;DLC1;DDIT4;ZBTB20;PTPRJ;TNS3;SYNE2
SSH2	8/299	0.00152334314 77595953	SLFN5;ST6GAL1;DLC1;DDIT4;ZBTB20;PTPRJ;TNS3;SYNE2
TFDP2	8/299	0.00152334314 77595953	ASPM;ST6GAL1;TRIM2;DLC1;DDIT4;ZBTB20;SYNE2;MAP2K6
THRB	8/299	0.00152334314 77595953	AR;PLCB4;ERBB4;TRIM2;DLC1;ZBTB20;TNS3;ESR1
TIGD6	8/299	0.00152334314 77595953	C5;RBM47;DLC1;DDIT4;PTPRJ;FRMD4B;TNS3;SYNE2
TRERF1	8/299	0.00152334314 77595953	ST6GAL1;HIP1;DLC1;DDIT4;PTPRJ;IKZF2;TNS3;SYNE2
ZBED3	8/299	0.00152334314 77595953	ST6GAL1;HIP1;DLC1;DDIT4;ZBTB20;LAMB1;TNS3;JDP2
ZBTB38	8/299	0.00152334314 77595953	SLFN5;ST6GAL1;TRIM2;DLC1;DDIT4;ZBTB20;TNS3;SYNE2
ZFP14	8/299	0.00152334314 77595953	ZMAT1;PLCB4;TRIM2;DLC1;DDIT4;ZBTB20;IKZF2;MAP2K6
ZNF10	8/299	0.00152334314 77595953	ZMAT1;TRIM2;DLC1;DDIT4;ZBTB20;SPRY1;IKZF2;SYNE2
ZNF117	8/299	0.00152334314 77595953	SLFN5;ZMAT1;HIP1;TRIM2;DLC1;DDIT4;ZBTB20;SYNE2
ZNF177	8/299	0.00152334314 77595953	PCDHGA5;DLC1;DDIT4;ZBTB20;LAMB1;SPRY1;PRICKLE1;ZNF610
ZNF233	8/299	0.00152334314 77595953	PCDHGA5;PLCB4;DLC1;DDIT4;ZBTB20;ZNF546;ZNF610;SYNE2
ZNF280B	8/299	0.00152334314 77595953	ASPM;ST6GAL1;PLCB4;TRIM2;DLC1;DDIT4;ETV1;PRICKLE1
ZNF284	8/299	0.00152334314 77595953	SLFN5;PCDHGA5;DLC1;DDIT4;ZBTB20;ZNF546;SRGAP3;ZNF610
ZNF287	8/299	0.00152334314 77595953	SLFN5;PLCB4;TRIM2;DLC1;DDIT4;ZBTB20;ETV1;IKZF2
ZNF345	8/299	0.00152334314 77595953	SLFN5;ZMAT1;DLC1;DDIT4;ZBTB20;IKZF2;ZNF610;SYNE2
ZNF382	8/299	0.00152334314 77595953	SLFN5;ZMAT1;PCDHGA5;ZNF704;DLC1;DDIT4;ZBTB20;ZNF610
ZNF43	8/299	0.00152334314 77595953	ASPM;PLCB4;TRIM2;DLC1;DDIT4;ZBTB20;ETV1;ZNF610
ZNF441	8/299	0.00152334314 77595953	ASPM;SLFN5;PCDHGA5;GNG2;DLC1;DDIT4;ZBTB20;ZNF546
ZNF462	8/299	0.00152334314 77595953	TRIM2;DLC1;DDIT4;ZBTB20;ETV1;LAMB1;TNS3;SYNE2
ZNF483	8/299	0.00152334314 77595953	ST6GAL1;PCDHGA5;PLCB4;ERBB4;DLC1;DDIT4;ETV1;SYNE2
ZNF506	8/299	0.00152334314 77595953	SLFN5;ZMAT1;PCDHGA5;MX1;ZBTB20;IKZF2;ZNF610;SYNE2
ZNF518B	8/299	0.00152334314 77595953	SLFN5;ST6GAL1;RBM47;GNG2;DLC1;DDIT4;ZBTB20;PRICKLE1
ZNF528	8/299	0.00152334314 77595953	SLFN5;ZMAT1;PCDHGA5;DLC1;DDIT4;MX1;ZBTB20;ZNF610
ZNF532	8/299	0.00152334314 77595953	HIP1;TRIM2;DLC1;DDIT4;ZBTB20;LAMB1;TNS3;SYNE2

ZNF552	8/299	0.0015233431477595953	AR;ST6GAL1;RBM47;CLMN;DDIT4;MX1;TNS3;ESR1
ZNF571	8/299	0.0015233431477595953	SLFN5;ZMAT1;DLC1;DDIT4;MX1;ZBTB20;IKZF2;ZNF610
ZNF583	8/299	0.0015233431477595953	SLFN5;PLCB4;DLC1;DDIT4;ZBTB20;SPRY1;IKZF2;ZNF610
ZNF594	8/299	0.0015233431477595953	ASPM;SLFN5;ZMAT1;TRIM2;DLC1;DDIT4;ZNF546;ZNF610
ZNF596	8/299	0.0015233431477595953	SLFN5;ZMAT1;DLC1;DDIT4;ZBTB20;ZNF546;SRGAP3;ZNF610
ZNF624	8/299	0.0015233431477595953	ASPM;SLFN5;C5;TRIM2;DLC1;DDIT4;PRICKLE1;IKZF2
ZNF645	8/299	0.0015233431477595953	ASPM;ERBB4;ZNF704;DLC1;DDIT4;ZBTB20;ETV1;ESR1
ZNF699	8/299	0.0015233431477595953	SLFN5;PCDHGA5;TRIM2;DLC1;DDIT4;LAMB1;SPRY1;FRMD4B
ZNF763	8/299	0.0015233431477595953	SLFN5;ZMAT1;PCDHGA5;ZNF704;DLC1;DDIT4;ZNF546;ZNF610
ZNF77	8/299	0.0015233431477595953	ASPM;PCDHGA5;DLC1;DDIT4;MX1;KIF14;SPRY1;TNS3
ZNF790	8/299	0.0015233431477595953	SLFN5;ZMAT1;PCDHGA5;DLC1;DDIT4;ZNF546;PRICKLE1;ZNF610
ZNF792	8/299	0.0015233431477595953	SLFN5;ST6GAL1;ZNF704;DLC1;DDIT4;MX1;SPRY1;TNS3
ZNF808	8/299	0.0015233431477595953	SLFN5;PCDHGA5;DLC1;DDIT4;MX1;PTPRJ;PRICKLE1;TNS3
ZNF846	8/299	0.0015233431477595953	SLFN5;ZMAT1;TRIM2;DLC1;DDIT4;ZBTB20;ZNF546;ETV1
AFF1	7/299	0.00518201064462339	RBM47;DLC1;DDIT4;ZBTB20;PTPRJ;TNS3;SYNE2
AFF3	7/299	0.00518201064462339	ST6GAL1;PLCB4;DLC1;ZBTB20;SRGAP3;PRICKLE1;TNS3
ATOH1	7/299	0.00518201064462339	NEURL1B;NR5A2;ERBB4;DLC1;DDIT4;ETV1;ESR1
BCL11A	7/299	0.00518201064462339	ST6GAL1;DLC1;DDIT4;ZBTB20;ETV1;SRGAP3;TNS3
CAMTA1	7/299	0.00518201064462339	PCDHGA5;TRIM2;DLC1;ZBTB20;SRGAP3;TNS3;ESR1
CHD6	7/299	0.00518201064462339	TRIM2;DLC1;DDIT4;ZBTB20;SRGAP3;TNS3;SYNE2
CREB3L4	7/299	0.00518201064462339	ASPM;ST6GAL1;RBM47;SLC2A10;DLC1;DDIT4;ESR1
DEPDC5	7/299	0.00518201064462339	PCDHGA5;PLCB4;DLC1;DDIT4;ZBTB20;TNS3;SYNE2
ELF5	7/299	0.00518201064462339	AR;PLCB4;ERBB4;DLC1;DDIT4;ETV1;ESR1
ESRRB	7/299	0.00518201064462339	NR5A2;ERBB4;DLC1;ETV1;PRICKLE1;TNS3;ESR1
FGD1	7/299	0.00518201064462339	ASPM;HIP1;DLC1;DDIT4;LAMB1;PRICKLE1;TNS3
FOXN3	7/299	0.00518201064462339	ST6GAL1;TRIM2;DLC1;DDIT4;ZBTB20;TNS3;SYNE2
FOXP2	7/299	0.00518201064462339	PLCB4;SEMA3D;ERBB4;DLC1;ZBTB20;ETV1;ESR1
GRHL2	7/299	0.00518201064462339	RBM47;NR5A2;TRIM2;DLC1;DDIT4;TNS3;ESR1
HLF	7/299	0.00518201064462339	AR;PLCB4;DLC1;DDIT4;ZBTB20;ETV1;SPRY1
HOXA11	7/299	0.00518201064	NNMT;NR5A2;ALDH1A2;DLC1;DDIT4;ETV1;E

		462339	SR1
HOXA4	7/299	0.00518201064462339	NNMT;NR5A2;ALDH1A2;DLC1;DDIT4;SPRY1;PRICKLE1
HOXC4	7/299	0.00518201064462339	PCDHGA5;NR5A2;ALDH1A2;DLC1;DDIT4;TNS3;ESR1
KLF8	7/299	0.00518201064462339	AR;SLFN5;ST6GAL1;ST8SIA1;DLC1;DDIT4;IKZF2
MEF2C	7/299	0.00518201064462339	GNG2;DLC1;DDIT4;ZBTB20;ETV1;PRICKLE1;TNS3
MITF	7/299	0.00518201064462339	AR;DLC1;ZBTB20;ETV1;FRMD4B;TNS3;ESR1
MSGN1	7/299	0.00518201064462339	RBM47;NR5A2;ST8SIA1;ALDH1A2;DLC1;DDIT4;ESR1
NFIB	7/299	0.00518201064462339	TRIM2;DLC1;DDIT4;ZBTB20;ETV1;TNS3;SYNE2
NOBOX	7/299	0.00518201064462339	AR;NR5A2;ERBB4;DLC1;DDIT4;ETV1;ESR1
NPAS3	7/299	0.00518201064462339	ERBB4;DLC1;ZBTB20;ETV1;SRGAP3;TNS3;ESR1
NR2F2	7/299	0.00518201064462339	DLC1;DDIT4;ZBTB20;ETV1;LAMB1;SPRY1;TNS3
NR3C1	7/299	0.00518201064462339	AR;DLC1;DDIT4;ZBTB20;TNS3;ESR1;SYNE2
ONECUT1	7/299	0.00518201064462339	AR;NNMT;NR5A2;ERBB4;DLC1;DDIT4;ESR1
PAX3	7/299	0.00518201064462339	PCDHGA5;NR5A2;ERBB4;ALDH1A2;DLC1;ETV1;ESR1
PAX6	7/299	0.00518201064462339	NR5A2;ERBB4;DLC1;DDIT4;ZBTB20;ETV1;ESR1
PBX3	7/299	0.00518201064462339	GNG2;DLC1;DDIT4;ZBTB20;ETV1;SPRY1;TNS3
PKHD1	7/299	0.00518201064462339	PCDHGA5;NR5A2;ERBB4;DLC1;ZBTB20;ESR1;SYNE2
PLXNA4	7/299	0.00518201064462339	ERBB4;DLC1;ZBTB20;LAMB1;SRGAP3;PRICKLE1;TNS3
PLXNB1	7/299	0.00518201064462339	CLMN;TRIM2;DLC1;DDIT4;LAMB1;TNS3;SYNE2
POU1F1	7/299	0.00518201064462339	AR;NR5A2;ERBB4;DLC1;DDIT4;ETV1;ESR1
PRB3	7/299	0.00518201064462339	AR;ASPM;RBM47;DLC1;DDIT4;MX1;KIF14
PRDM7	7/299	0.00518201064462339	ASPM;DLC1;DDIT4;MX1;KIF14;SPRY1;BEST1
RNF113B	7/299	0.00518201064462339	PCDHGA5;DLC1;DDIT4;ZBTB20;SPRY1;TNS3;ESR1
RREB1	7/299	0.00518201064462339	RBM47;DLC1;DDIT4;ZBTB20;PTPRJ;TNS3;SYNE2
SALL2	7/299	0.00518201064462339	TRIM2;DLC1;DDIT4;VASH2;ETV1;SPRY1;PRICKLE1
SETBP1	7/299	0.00518201064462339	DLC1;DDIT4;ZBTB20;SRGAP3;PRICKLE1;TNS3;SYNE2
SIX4	7/299	0.00518201064462339	ASPM;TRIM2;DLC1;DDIT4;ETV1;PRICKLE1;TNS3
SOX13	7/299	0.00518201064462339	TRIM2;DLC1;DDIT4;SPRY1;TNS3;PDE9A;SYNE2
SOX2	7/299	0.00518201064462339	AR;MEGF10;TRIM2;DLC1;DDIT4;ETV1;ESR1
ST18	7/299	0.00518201064462339	GNG2;ERBB4;DLC1;ZBTB20;ETV1;FRMD4B;RAPGEF4

TCF7L1	7/299	0.00518201064462339	DLC1;DDIT4;ETV1;LAMB1;SPRY1;PRICKLE1;TNS3
TFAP2B	7/299	0.00518201064462339	AR;ERBB4;ALDH1A2;DLC1;SLITRK6;ETV1;ESR1
TFAP2C	7/299	0.00518201064462339	RBM47;NR5A2;DLC1;DDIT4;ETV1;TNS3;ESR1
TFEB	7/299	0.00518201064462339	ST6GAL1;DLC1;DDIT4;PTPRJ;PTPN6;FRMD4B;TNS3
TP63	7/299	0.00518201064462339	AR;ERBB4;DLC1;DDIT4;ZBTB20;ETV1;ESR1
WT1	7/299	0.00518201064462339	AR;NR5A2;ERBB4;ALDH1A2;DLC1;DDIT4;ESR1
ZBTB37	7/299	0.00518201064462339	ASPM;HIP1;DLC1;DDIT4;ZBTB20;IKZF2;SYNE2
ZBTB47	7/299	0.00518201064462339	HIP1;PCDHGA5;ZNF704;DLC1;DDIT4;ZBTB20;TNS3
ZBTB7C	7/299	0.00518201064462339	ST6GAL1;RBM47;PCDHGA5;PLCB4;DLC1;DDIT4;TNS3
ZC3H6	7/299	0.00518201064462339	SLFN5;ZMAT1;TRIM2;DLC1;DDIT4;ZBTB20;SYNE2
ZFP37	7/299	0.00518201064462339	PCDHGA5;PLCB4;TRIM2;DLC1;DDIT4;ZBTB20;ETV1
ZFPM2	7/299	0.00518201064462339	PLCB4;ERBB4;DLC1;ZBTB20;ETV1;LAMB1;PRICKLE1
ZIC3	7/299	0.00518201064462339	AR;NR5A2;ALDH1A2;DLC1;DDIT4;VASH2;ETV1
ZIM2	7/299	0.00518201064462339	PCDHGA5;ERBB4;DLC1;ZBTB20;ESR1;SYNE2;RAPGEF4
ZNF2	7/299	0.00518201064462339	PLCB4;DLC1;DDIT4;KIF14;ETV1;PRICKLE1;JDP2
ZNF253	7/299	0.00518201064462339	SLFN5;ST6GAL1;ZNF704;DLC1;ZBTB20;ZNF610;SYNE2
ZNF300	7/299	0.00518201064462339	ASPM;BEX2;PCDHGA5;DLC1;KIF14;LAMB1;TNS3
ZNF329	7/299	0.00518201064462339	ST6GAL1;BEX2;DLC1;DDIT4;MX1;PRICKLE1;TNS3
ZNF347	7/299	0.00518201064462339	SLFN5;PCDHGA5;ZNF704;DLC1;ZBTB20;IKZF2;ZNF610
ZNF41	7/299	0.00518201064462339	AR;ASPM;SLFN5;PCDHGA5;ZBTB20;IKZF2;TNS3
ZNF415	7/299	0.00518201064462339	AR;PLCB4;DLC1;DDIT4;MX1;ZBTB20;ZNF610
ZNF439	7/299	0.00518201064462339	ZMAT1;PCDHGA5;DLC1;DDIT4;MX1;ZBTB20;ZNF610
ZNF44	7/299	0.00518201064462339	SLFN5;PCDHGA5;CLMN;DLC1;DDIT4;ZBTB20;SYNE2
ZNF440	7/299	0.00518201064462339	RBM47;ZMAT1;CLMN;ZNF704;DLC1;DDIT4;IKZF2
ZNF471	7/299	0.00518201064462339	AR;PCDHGA5;PLCB4;ZNF704;DLC1;ZBTB20;ZNF610
ZNF501	7/299	0.00518201064462339	SLFN5;BEX2;PCDHGA5;DLC1;DDIT4;SPRY1;ZNF610
ZNF514	7/299	0.00518201064462339	NEURL1B;ZMAT1;HIP1;ZNF704;DLC1;DDIT4;ZBTB20
ZNF570	7/299	0.00518201064462339	SLFN5;DLC1;DDIT4;ZNF546;SPRY1;ZNF610;PDE9A
ZNF573	7/299	0.00518201064462339	ASPM;PCDHGA5;DLC1;DDIT4;ZBTB20;TNS3;SYNE2
ZNF578	7/299	0.00518201064	PCDHGA11;ASPM;PCDHGA5;NR5A2;ERBB4;DL

		462339	C1;ZNF610
ZNF585A	7/299	0.00518201064462339	SLFN5;ZMAT1;DLC1;DDIT4;ZBTB20;ZNF546;ZNF610
ZNF605	7/299	0.00518201064462339	SLFN5;PLCB4;TRIM2;DDIT4;ZBTB20;IKZF2;SYNE2
ZNF626	7/299	0.00518201064462339	AR;SLFN5;ST6GAL1;RBM47;DLC1;ZBTB20;ZNF610
ZNF648	7/299	0.00518201064462339	PCDHGA5;NR5A2;DLC1;DDIT4;KIF14;TNS3;ESR1
ZNF649	7/299	0.00518201064462339	BEX2;RBM47;DLC1;DDIT4;MX1;VASH2;PRICKLE1
ZNF662	7/299	0.00518201064462339	AR;ZMAT1;PCDHGA5;PLCB4;DLC1;ESR1;RAPGEF4
ZNF667	7/299	0.00518201064462339	AR;PCDHGA5;DLC1;DDIT4;SPRY1;ZNF610;TNS3
ZNF678	7/299	0.00518201064462339	ASPM;ZNF704;TRIM2;DLC1;DDIT4;KIF14;SYNE2
ZNF682	7/299	0.00518201064462339	AR;ASPM;RBM47;DLC1;DDIT4;MX1;PDE9A
ZNF70	7/299	0.00518201064462339	SLFN5;PCDHGA5;ZNF704;TRIM2;DLC1;DDIT4;TNS3
ZNF710	7/299	0.00518201064462339	RBM47;DLC1;DDIT4;ZBTB20;PTPRJ;FRMD4B;TNS3
ZNF718	7/299	0.00518201064462339	ST6GAL1;PCDHGA5;PLCB4;DLC1;DDIT4;TNS3;SYNE2
ZNF782	7/299	0.00518201064462339	ASPM;DLC1;DDIT4;ZBTB20;ZNF546;TNS3;SYNE2
ZNF827	7/299	0.00518201064462339	SLFN5;ZNF704;TRIM2;DLC1;DDIT4;ZBTB20;TNS3
ZNF90	7/299	0.00518201064462339	ASPM;RBM47;DLC1;MX1;VASH2;KIF14;SYNE2
ZNF91	7/299	0.00518201064462339	ASPM;PLCB4;TRIM2;DDIT4;ZBTB20;TNS3;SYNE2
ZSCAN16	7/299	0.00518201064462339	ASPM;BEX2;RBM47;DLC1;DDIT4;MX1;PRICKLE1
ZSCAN2	7/299	0.00518201064462339	ST6GAL1;DLC1;DDIT4;ZBTB20;SPRY1;PRICKLE1;TNS3
ZXDA	7/299	0.00518201064462339	AR;BEX2;PCDHGA5;TRIM2;DLC1;DDIT4;ZBTB20
AKNA	6/299	0.01620251814116195	SLFN5;ST6GAL1;ARHGAP30;DLC1;DDIT4;PTPN6
ALX1	6/299	0.01620251814116195	ERBB4;ALDH1A2;DLC1;DDIT4;ETV1;PRICKLE1
ALX4	6/299	0.01620251814116195	PCDHGA5;NR5A2;ALDH1A2;DLC1;TNS3;ESR1
AR	6/299	0.01620251814116195	ERBB4;DLC1;DDIT4;ZBTB20;ETV1;ESR1
ARID5B	6/299	0.01620251814116195	DLC1;DDIT4;ZBTB20;SPRY1;TNS3;SYNE2
ARNT2	6/299	0.01620251814116195	TRIM2;DLC1;DDIT4;ETV1;SRGAP3;TNS3
ASCL1	6/299	0.01620251814116195	ERBB4;ALDH1A2;DLC1;DDIT4;ETV1;ESR1
BARX2	6/299	0.01620251814116195	PCDHGA5;NR5A2;ALDH1A2;DLC1;DDIT4;ESR1
BAZ2B	6/299	0.01620251814116195	TRIM2;DLC1;DDIT4;ZBTB20;FRMD4B;SYNE2
BCL11B	6/299	0.01620251814116195	GNG2;DLC1;DDIT4;ZBTB20;TNS3;SYNE2

BNC2	6/299	0.01620251814116195	NNMT;DLC1;ZBTB20;ETV1;PRICKLE1;TNS3
CASZ1	6/299	0.01620251814116195	RBM47;DLC1;DDIT4;ZBTB20;TNS3;SYNE2
CHD9	6/299	0.01620251814116195	TRIM2;DLC1;DDIT4;ZBTB20;TNS3;SYNE2
CREB3L2	6/299	0.01620251814116195	HIP1;DLC1;DDIT4;ZBTB20;LAMB1;TNS3
CREB5	6/299	0.01620251814116195	RBM47;DLC1;DDIT4;ZBTB20;ETV1;TNS3
CUX1	6/299	0.01620251814116195	ST6GAL1;DLC1;ZBTB20;PTPRJ;TNS3;SYNE2
DLX1	6/299	0.01620251814116195	ALDH1A2;DLC1;DDIT4;ETV1;SPRY1;PRICKLE1
EMX2	6/299	0.01620251814116195	AR;ALDH1A2;DLC1;DDIT4;ZBTB20;ETV1
FOXD3	6/299	0.01620251814116195	NR5A2;MEGF10;ALDH1A2;DLC1;DDIT4;ETV1
FOXD4L6	6/299	0.01620251814116195	PCDHGA5;NR5A2;ALDH1A2;DLC1;TNS3;ESR1
FOXG1	6/299	0.01620251814116195	AR;ERBB4;DLC1;DDIT4;ZBTB20;ETV1
FOXO1	6/299	0.01620251814116195	DLC1;DDIT4;ZBTB20;SPRY1;TNS3;ESR1
GBX2	6/299	0.01620251814116195	NR5A2;ALDH1A2;DLC1;DDIT4;ETV1;SPRY1
GLI2	6/299	0.01620251814116195	DLC1;DDIT4;ETV1;PRICKLE1;TNS3;ESR1
GTF2IRD2	6/299	0.01620251814116195	DLC1;DDIT4;ZBTB20;FRMD4B;SRGAP3;TNS3
HIVEP1	6/299	0.01620251814116195	DLC1;DDIT4;ZBTB20;PTPRJ;TNS3;SYNE2
HKR1	6/299	0.01620251814116195	HIP1;PCDHGA5;DLC1;DDIT4;ZBTB20;ESR1
HMBOX1	6/299	0.01620251814116195	DLC1;DDIT4;ZBTB20;ETV1;TNS3;SYNE2
HNF1A	6/299	0.01620251814116195	AR;NR5A2;DLC1;DDIT4;ETV1;ESR1
HNF4A	6/299	0.01620251814116195	AR;RBM47;NNMT;NR5A2;DLC1;ESR1
HOXA1	6/299	0.01620251814116195	NR5A2;ALDH1A2;DLC1;DDIT4;LAMB1;PRICKLE1
HOXA2	6/299	0.01620251814116195	NR5A2;ALDH1A2;DLC1;LAMB1;SPRY1;PRICKLE1
HOXC11	6/299	0.01620251814116195	PCDHGA5;ALDH1A2;DLC1;ETV1;TNS3;ESR1
HOXC5	6/299	0.01620251814116195	PCDHGA5;NR5A2;ALDH1A2;DLC1;DDIT4;TNS3
IRF2	6/299	0.01620251814116195	SLFN5;DLC1;DDIT4;ZBTB20;PTPRJ;TNS3
KLF14	6/299	0.01620251814116195	PCDHGA5;NR5A2;SEMA3D;ALDH1A2;DLC1;ESR1
LBX2	6/299	0.01620251814116195	PCDHGA5;NR5A2;ALDH1A2;DLC1;DDIT4;ESR1
LCORL	6/299	0.01620251814116195	TRIM2;DLC1;DDIT4;ZBTB20;TNS3;SYNE2
MEIS1	6/299	0.01620251814116195	DLC1;DDIT4;ZBTB20;ETV1;PRICKLE1;TNS3
MKRN3	6/299	0.01620251814	ASPM;ERBB4;ALDH1A2;DLC1;DDIT4;ETV1

		116195	
MTA3	6/299	0.01620251814 116195	ST6GAL1;RBM47;DLC1;DDIT4;TNS3;SYNE2
MYCN	6/299	0.01620251814 116195	DLC1;DDIT4;ETV1;SPRY1;TNS3;ESR1
MYOD1	6/299	0.01620251814 116195	AR;PCDHGA5;NR5A2;DLC1;DDIT4;ESR1
NANOG	6/299	0.01620251814 116195	AR;NR5A2;DLC1;DDIT4;ETV1;ESR1
NCOA2	6/299	0.01620251814 116195	DLC1;ZBTB20;PTPRJ;TNS3;ESR1;SYNE2
NFATC2	6/299	0.01620251814 116195	ST6GAL1;DLC1;DDIT4;ZBTB20;PTPRJ;TNS3
NFATC4	6/299	0.01620251814 116195	DLC1;DDIT4;ETV1;LAMB1;SPRY1;P3H3
NR0B1	6/299	0.01620251814 116195	AR;BEX2;NR5A2;ALDH1A2;DLC1;ESR1
NR1H4	6/299	0.01620251814 116195	AR;NNMT;NR5A2;DLC1;DDIT4;ESR1
NR1I2	6/299	0.01620251814 116195	AR;NR5A2;DLC1;DDIT4;ESR1;RAPGEF4
NR1I3	6/299	0.01620251814 116195	AR;NNMT;DLC1;DDIT4;ESR1;RAPGEF4
OTX2	6/299	0.01620251814 116195	RBM47;NR5A2;ALDH1A2;DLC1;DDIT4;ETV1
PAX7	6/299	0.01620251814 116195	PCDHGA5;NR5A2;ERBB4;DLC1;ETV1;ESR1
PAX8	6/299	0.01620251814 116195	PCDHGA5;NR5A2;TRIM2;DLC1;DDIT4;ESR1
PAX9	6/299	0.01620251814 116195	PCDHGA5;NR5A2;ALDH1A2;DLC1;DDIT4;ESR 1
PGR	6/299	0.01620251814 116195	AR;NR5A2;ERBB4;DLC1;ETV1;ESR1
PITX1	6/299	0.01620251814 116195	NNMT;NR5A2;DLC1;DDIT4;LAMB1;ESR1
PKHD1L1	6/299	0.01620251814 116195	ASPM;SEMA3D;ERBB4;ALDH1A2;DLC1;SYNE2
PLAG1	6/299	0.01620251814 116195	DLC1;DDIT4;ZBTB20;ETV1;SPRY1;IKZF2
PLEKHA4	6/299	0.01620251814 116195	SLFN5;NNMT;DLC1;DDIT4;MX1;LAMB1
PLXNA1	6/299	0.01620251814 116195	HIP1;DLC1;DDIT4;PTPRJ;LAMB1;TNS3
PLXNC1	6/299	0.01620251814 116195	ST6GAL1;GNG2;DLC1;DDIT4;PTPRJ;TNS3
POU5F1	6/299	0.01620251814 116195	AR;RBM47;NR5A2;DLC1;DDIT4;ESR1
PPARA	6/299	0.01620251814 116195	AR;TRIM2;DLC1;DDIT4;ZBTB20;ESR1
PRB4	6/299	0.01620251814 116195	ASPM;ALDH1A2;DLC1;DDIT4;MX1;TMEM150C
PRDM16	6/299	0.01620251814 116195	PCDHGA5;ERBB4;DLC1;ZBTB20;TNS3;ESR1
PROP1	6/299	0.01620251814 116195	AR;NR5A2;DLC1;DDIT4;ETV1;ESR1
RAG1	6/299	0.01620251814 116195	ASPM;DLC1;DDIT4;ZBTB20;IKZF2;ESR1
RBPJL	6/299	0.01620251814 116195	PCDHGA5;NR5A2;DLC1;DDIT4;SPRY1;ESR1

RNF125	6/299	0.01620251814116195	SLFN5;ST6GAL1;RBM47;CLMN;DLC1;DDIT4
RORA	6/299	0.01620251814116195	PLCB4;DLC1;DDIT4;ZBTB20;SPRY1;SYNE2
RPA4	6/299	0.01620251814116195	DLC1;DDIT4;MX1;SLITRK6;ZBTB20;PRICKLE1
SALL1	6/299	0.01620251814116195	NR5A2;DLC1;DDIT4;ETV1;SPRY1;TNS3
SORBS2	6/299	0.01620251814116195	PLCB4;TRIM2;DLC1;ZBTB20;TNS3;SYNE2
SOX10	6/299	0.01620251814116195	MEGF10;TRIM2;ALDH1A2;DLC1;DDIT4;ETV1
SP4	6/299	0.01620251814116195	DLC1;DDIT4;ZBTB20;ETV1;IKZF2;SYNE2
TAL2	6/299	0.01620251814116195	ASPM;PCDHGA5;ALDH1A2;DLC1;DDIT4;ETV1
TBX19	6/299	0.01620251814116195	ASPM;NR5A2;DLC1;DDIT4;KIF14;ESR1
TBX2	6/299	0.01620251814116195	DLC1;DDIT4;ETV1;LAMB1;SPRY1;TNS3
TEAD1	6/299	0.01620251814116195	TRIM2;DLC1;DDIT4;ZBTB20;LAMB1;TNS3
TFCP2L1	6/299	0.01620251814116195	RBM47;CLMN;TRIM2;DLC1;DDIT4;TNS3
TFDP3	6/299	0.01620251814116195	AR;PLCB4;SEMA3D;DLC1;ZBTB20;ESR1
THRA	6/299	0.01620251814116195	TRIM2;DLC1;DDIT4;ZBTB20;TNS3;ESR1
TIGD4	6/299	0.01620251814116195	ST6GAL1;RBM47;ZMAT1;PCDHGA5;TRIM2;DLC1
TIGD7	6/299	0.01620251814116195	ASPM;BEX2;PCDHGA5;DDIT4;PRICKLE1;TNS3
TOX2	6/299	0.01620251814116195	GNG2;DLC1;DDIT4;SPRY1;PRICKLE1;TNS3
TUB	6/299	0.01620251814116195	NEURL1B;PCDHGA5;GNG2;TRIM2;DLC1;ETV1
ZBTB10	6/299	0.01620251814116195	DLC1;DDIT4;ZBTB20;SPRY1;TNS3;SYNE2
ZBTB4	6/299	0.01620251814116195	ST6GAL1;TRIM2;DLC1;DDIT4;ZBTB20;TNS3
ZDHHC11	6/299	0.01620251814116195	NNMT;NR5A2;DLC1;DDIT4;LAMB1;JDP2
ZFAT	6/299	0.01620251814116195	DLC1;DDIT4;ZBTB20;PTPRJ;TNS3;SYNE2
ZFHX2	6/299	0.01620251814116195	TRIM2;DLC1;DDIT4;ZBTB20;SRGAP3;TNS3
ZHX3	6/299	0.01620251814116195	HIP1;TRIM2;DLC1;DDIT4;ZBTB20;TNS3
ZIC4	6/299	0.01620251814116195	PCDHGA5;NR5A2;ERBB4;ALDH1A2;DLC1;ESR1
ZIK1	6/299	0.01620251814116195	ASPM;PCDHGA5;DLC1;DDIT4;VASH2;MAP2K6
ZNF114	6/299	0.01620251814116195	NEURL1B;DLC1;DDIT4;MX1;KIF14;TNS3
ZNF141	6/299	0.01620251814116195	AR;ASPM;DLC1;DDIT4;IKZF2;SYNE2
ZNF169	6/299	0.01620251814116195	ASPM;DLC1;DDIT4;ZNF546;TNS3;EXPH5
ZNF208	6/299	0.01620251814	PCDHGA5;NR5A2;ST8SIA1;ERBB4;DLC1;SYN

		116195	E2
ZNF215	6/299	0.01620251814 116195	ASPM;DLC1;DDIT4;KIF14;ETV1;SYNE2
ZNF234	6/299	0.01620251814 116195	SLFN5;DDIT4;MX1;ZNF546;TNS3;SYNE2
ZNF239	6/299	0.01620251814 116195	ASPM;DLC1;DDIT4;MX1;KIF14;PRICKLE1
ZNF248	6/299	0.01620251814 116195	PCDHGA5;DLC1;DDIT4;PRICKLE1;IKZF2;SY NE2
ZNF254	6/299	0.01620251814 116195	ASPM;PLCB4;TRIM2;DLC1;DDIT4;ZBTB20
ZNF30	6/299	0.01620251814 116195	SLFN5;DLC1;DDIT4;MX1;ZNF610;EXPH5
ZNF333	6/299	0.01620251814 116195	SLFN5;PCDHGA5;DLC1;DDIT4;ZBTB20;TNS3
ZNF337	6/299	0.01620251814 116195	ASPM;DDIT4;KIF14;SPRY1;TNS3;SYNE2
ZNF33B	6/299	0.01620251814 116195	RBM47;PLCB4;DDIT4;SPRY1;TNS3;SYNE2
ZNF366	6/299	0.01620251814 116195	RBM47;PCDHGA5;ALDH1A2;DLC1;ZBTB20;TN S3
ZNF385D	6/299	0.01620251814 116195	PCDHGA5;PLCB4;SEMA3D;DLC1;ZBTB20;ETV 1
ZNF407	6/299	0.01620251814 116195	PCDHGA5;DLC1;ZBTB20;PTPRJ;TNS3;SYNE2
ZNF470	6/299	0.01620251814 116195	HIP1;PCDHGA5;DLC1;DDIT4;MX1;ZNF610
ZNF488	6/299	0.01620251814 116195	NEURL1B;DLC1;DDIT4;VASH2;ETV1;PRICKL E1
ZNF493	6/299	0.01620251814 116195	ST6GAL1;ZMAT1;DDIT4;ZBTB20;ZNF610;SY NE2
ZNF502	6/299	0.01620251814 116195	SLFN5;BEX2;PCDHGA5;DLC1;DDIT4;ZBTB20
ZNF521	6/299	0.01620251814 116195	DLC1;DDIT4;ZBTB20;ETV1;SPRY1;PRICKLE 1
ZNF540	6/299	0.01620251814 116195	ZMAT1;PCDHGA5;ST8SIA1;DLC1;ZBTB20;ZN F610
ZNF544	6/299	0.01620251814 116195	RBM47;PCDHGA5;DLC1;DDIT4;MX1;SYNE2
ZNF547	6/299	0.01620251814 116195	PCDHGA5;DLC1;DDIT4;ZBTB20;ZNF610;JDP 2
ZNF554	6/299	0.01620251814 116195	SLFN5;PCDHGA5;DDIT4;MX1;ZBTB20;EXPH5
ZNF555	6/299	0.01620251814 116195	CLMN;DLC1;DDIT4;MX1;ZBTB20;ZNF610
ZNF556	6/299	0.01620251814 116195	ASPM;DLC1;DDIT4;KIF14;ZBTB20;TNS3
ZNF563	6/299	0.01620251814 116195	SLFN5;ZMAT1;DLC1;DDIT4;PRICKLE1;MAP2 K6
ZNF565	6/299	0.01620251814 116195	PCDHGA5;DLC1;DDIT4;ZBTB20;LAMB1;PDE9 A
ZNF572	6/299	0.01620251814 116195	SLFN5;PCDHGA5;DLC1;DDIT4;ZNF610;PDE9 A
ZNF577	6/299	0.01620251814 116195	ZMAT1;PCDHGA5;DLC1;DDIT4;ZBTB20;ZNF6 10
ZNF589	6/299	0.01620251814 116195	ST6GAL1;NR5A2;DLC1;DDIT4;ETV1;SYNE2
ZNF606	6/299	0.01620251814 116195	PCDHGA5;DLC1;DDIT4;ZBTB20;ZNF610;SYN E2

ZNF611	6/299	0.01620251814 116195	HIP1;DLC1;DDIT4;ZBTB20;TNS3;SYNE2
ZNF616	6/299	0.01620251814 116195	ASPM;SLFN5;RBM47;DLC1;DDIT4;ZBTB20
ZNF641	6/299	0.01620251814 116195	SLFN5;RBM47;HIP1;DLC1;DDIT4;ZBTB20
ZNF658	6/299	0.01620251814 116195	NEURL1B;DLC1;DDIT4;KIF14;SLITRK6;ZBT B20
ZNF665	6/299	0.01620251814 116195	DLC1;DDIT4;ZBTB20;ZNF610;TNS3;SYNE2
ZNF681	6/299	0.01620251814 116195	ASPM;PCDHGA5;DLC1;KIF14;ZNF610;EXPH5
ZNF701	6/299	0.01620251814 116195	ASPM;SLFN5;DLC1;DDIT4;ZBTB20;ZNF610
ZNF75D	6/299	0.01620251814 116195	SLFN5;TRIM2;DLC1;DDIT4;ZBTB20;SYNE2
ZNF761	6/299	0.01620251814 116195	SLFN5;ST6GAL1;DLC1;DDIT4;IKZF2;SYNE2
ZNF8	6/299	0.01620251814 116195	HIP1;DLC1;DDIT4;ZBTB20;PRICKLE1;ESR1
ZNF83	6/299	0.01620251814 116195	ZMAT1;PLCB4;DLC1;DDIT4;ZBTB20;SYNE2
ZNF836	6/299	0.01620251814 116195	SLFN5;PCDHGA5;DLC1;MX1;ZBTB20;ZNF610
ZNF845	6/299	0.01620251814 116195	ASPM;SLFN5;ST6GAL1;RBM47;DDIT4;SYNE2
ZSCAN10	6/299	0.01620251814 116195	RBM47;NR5A2;MEGF10;DLC1;DDIT4;VASH2
ZSCAN18	6/299	0.01620251814 116195	BEX2;PCDHGA5;GNG2;DLC1;DDIT4;ZBTB20
ZSCAN20	6/299	0.01620251814 116195	ASPM;DLC1;DDIT4;PTPRJ;TNS3;SYNE2
ZSCAN22	6/299	0.01620251814 116195	NEURL1B;DLC1;DDIT4;ZBTB20;ETV1;TNS3
ZXDB	6/299	0.01620251814 116195	ST6GAL1;BEX2;TRIM2;DLC1;DDIT4;ZBTB20
AHDC1	5/299	0.04521396089 5433794	DLC1;DDIT4;ZBTB20;SRGAP3;TNS3
ASCL4	5/299	0.04521396089 5433794	NR5A2;ALDH1A2;DLC1;TNS3;ESR1
ATOH8	5/299	0.04521396089 5433794	NNMT;DLC1;DDIT4;SPRY1;TNS3
BNC1	5/299	0.04521396089 5433794	NNMT;ALDH1A2;DLC1;DDIT4;SPRY1
CDX4	5/299	0.04521396089 5433794	AR;RBM47;NR5A2;ALDH1A2;DLC1
CPXCR1	5/299	0.04521396089 5433794	AR;ST8SIA1;SEMA3D;DLC1;SLITRK6
CREBL2	5/299	0.04521396089 5433794	SLFN5;ST6GAL1;DLC1;DDIT4;TNS3
DEPDC4	5/299	0.04521396089 5433794	ASPM;PCDHGA5;DLC1;DDIT4;KIF14
DLX4	5/299	0.04521396089 5433794	NR5A2;ALDH1A2;DLC1;DDIT4;ESR1
DMBX1	5/299	0.04521396089 5433794	NR5A2;ALDH1A2;DLC1;DDIT4;SPRY1
DMRT1	5/299	0.04521396089 5433794	NR5A2;DLC1;DDIT4;TNS3;ESR1
DMRTB1	5/299	0.04521396089	NR5A2;ERBB4;ALDH1A2;DLC1;DDIT4

		5433794	
DMTF1	5/299	0.04521396089 5433794	DLC1;DDIT4;ZBTB20;ETV1;SYNE2
E2F5	5/299	0.04521396089 5433794	ST6GAL1;DLC1;DDIT4;ETV1;SPRY1
EBF1	5/299	0.04521396089 5433794	GNG2;DLC1;ZBTB20;SPRY1;TNS3
EBF4	5/299	0.04521396089 5433794	NEURL1B;PCDHGA5;DLC1;DDIT4;PRICKLE1
ELK3	5/299	0.04521396089 5433794	SLFN5;DLC1;DDIT4;LAMB1;TNS3
EN2	5/299	0.04521396089 5433794	NR5A2;ALDH1A2;DLC1;DDIT4;ETV1
ERG	5/299	0.04521396089 5433794	AR;DLC1;ZBTB20;TNS3;ESR1
ESR1	5/299	0.04521396089 5433794	AR;ERBB4;DLC1;DDIT4;ZBTB20
ESR2	5/299	0.04521396089 5433794	AR;NR5A2;ERBB4;DLC1;ESR1
ETV1	5/299	0.04521396089 5433794	PLCB4;GNG2;DLC1;DDIT4;SPRY1
ETV4	5/299	0.04521396089 5433794	ASPM;DLC1;DDIT4;ETV1;SPRY1
ETV5	5/299	0.04521396089 5433794	DLC1;DDIT4;ETV1;SPRY1;TNS3
ETV6	5/299	0.04521396089 5433794	DLC1;DDIT4;ZBTB20;PTPRJ;TNS3
FAM170A	5/299	0.04521396089 5433794	MEGF10;ERBB4;DLC1;SLITRK6;ESR1
FEZF1	5/299	0.04521396089 5433794	PCDHGA5;NR5A2;MEGF10;ERBB4;ALDH1A2
FOXA1	5/299	0.04521396089 5433794	AR;RBM47;DLC1;DDIT4;ESR1
FOXA3	5/299	0.04521396089 5433794	RBM47;NR5A2;DLC1;DDIT4;PDE9A
FOXC1	5/299	0.04521396089 5433794	ALDH1A2;DLC1;DDIT4;SPRY1;TNS3
FOXF2	5/299	0.04521396089 5433794	NNMT;DLC1;DDIT4;LAMB1;PRICKLE1
FOXI2	5/299	0.04521396089 5433794	PCDHGA5;NR5A2;ALDH1A2;DLC1;ESR1
FOXJ2	5/299	0.04521396089 5433794	HIP1;DLC1;DDIT4;ZBTB20;TNS3
FOXL1	5/299	0.04521396089 5433794	NNMT;DLC1;DDIT4;TNS3;ESR1
FOXO3	5/299	0.04521396089 5433794	DLC1;DDIT4;ZBTB20;TNS3;ESR1
GATA4	5/299	0.04521396089 5433794	RBM47;NR5A2;ALDH1A2;DLC1;ESR1
GLIS3	5/299	0.04521396089 5433794	DLC1;DDIT4;ZBTB20;LAMB1;TNS3
GPR155	5/299	0.04521396089 5433794	ST6GAL1;GNG2;DLC1;DDIT4;ZBTB20
GRHL1	5/299	0.04521396089 5433794	RBM47;CLMN;DLC1;DDIT4;IKZF2
GRM6	5/299	0.04521396089 5433794	HIP1;PCDHGA5;DLC1;ETV1;ESR1
H1FOO	5/299	0.04521396089 5433794	NNMT;ERBB4;DLC1;DDIT4;PDE9A

HAND1	5/299	0.04521396089 5433794	NR5A2;ALDH1A2;DLC1;DDIT4;PRICKLE1
HELT	5/299	0.04521396089 5433794	PCDHGA5;NR5A2;ERBB4;DLC1;ESR1
HEY1	5/299	0.04521396089 5433794	DLC1;DDIT4;ETV1;SPRY1;PRICKLE1
HEY2	5/299	0.04521396089 5433794	DLC1;DDIT4;ETV1;SPRY1;PRICKLE1
HIVEP2	5/299	0.04521396089 5433794	DLC1;DDIT4;ZBTB20;PTPRJ;TNS3
HIVEP3	5/299	0.04521396089 5433794	DLC1;DDIT4;ZBTB20;PTPRJ;TNS3
HLX	5/299	0.04521396089 5433794	RBM47;DLC1;DDIT4;SPRY1;TNS3
HMG20A	5/299	0.04521396089 5433794	ST6GAL1;DLC1;DDIT4;ZBTB20;TNS3
HMGA2	5/299	0.04521396089 5433794	ASPM;DLC1;DDIT4;KIF14;ETV1
HOMEZ	5/299	0.04521396089 5433794	DLC1;DDIT4;ETV1;PRICKLE1;JDP2
HOXA13	5/299	0.04521396089 5433794	NR5A2;ALDH1A2;DLC1;DDIT4;TNS3
HOXA3	5/299	0.04521396089 5433794	NR5A2;ALDH1A2;DLC1;DDIT4;LAMB1
HOXB1	5/299	0.04521396089 5433794	NR5A2;ALDH1A2;DLC1;TNS3;ESR1
HOXB3	5/299	0.04521396089 5433794	DLC1;DDIT4;PRICKLE1;TNS3;ESR1
HOXD13	5/299	0.04521396089 5433794	NR5A2;ALDH1A2;DLC1;ETV1;ESR1
HOXD3	5/299	0.04521396089 5433794	PCDHGA5;NR5A2;ALDH1A2;DLC1;ETV1
HOXD4	5/299	0.04521396089 5433794	PCDHGA5;ALDH1A2;DLC1;ETV1;SPRY1
ID4	5/299	0.04521396089 5433794	TRIM2;DLC1;DDIT4;ETV1;SPRY1
IFI16	5/299	0.04521396089 5433794	SLFN5;NNMT;DLC1;DDIT4;MX1
IKZF4	5/299	0.04521396089 5433794	DLC1;DDIT4;ZBTB20;ETV1;IKZF2
IRF4	5/299	0.04521396089 5433794	ST6GAL1;DLC1;DDIT4;MX1;ESR1
IRF8	5/299	0.04521396089 5433794	ARHGAP30;DLC1;DDIT4;MX1;PTPN6
ISL1	5/299	0.04521396089 5433794	ALDH1A2;DLC1;DDIT4;SLITRK6;ETV1
KLF11	5/299	0.04521396089 5433794	DLC1;DDIT4;ZBTB20;SPRY1;TNS3
KLF3	5/299	0.04521396089 5433794	DLC1;DDIT4;ZBTB20;FRMD4B;TNS3
LGR4	5/299	0.04521396089 5433794	TRIM2;DLC1;DDIT4;LAMB1;TNS3
LHX4	5/299	0.04521396089 5433794	PCDHGA5;NR5A2;ERBB4;DLC1;ESR1
LHX6	5/299	0.04521396089 5433794	NEURL1B;NR5A2;ALDH1A2;DLC1;DDIT4
LHX9	5/299	0.04521396089 5433794	NR5A2;ALDH1A2;DLC1;ETV1;ESR1
LIN28B	5/299	0.04521396089	DLC1;DDIT4;VASH2;KIF14;ETV1

		5433794	
LMX1A	5/299	0.04521396089 5433794	NR5A2;ERBB4;ALDH1A2;DLC1;ESR1
LMX1B	5/299	0.04521396089 5433794	NR5A2;ERBB4;ALDH1A2;DLC1;ESR1
MACF1	5/299	0.04521396089 5433794	DLC1;DDIT4;ZBTB20;TNS3;SYNE2
MAEL	5/299	0.04521396089 5433794	ASPM;NR5A2;ERBB4;DLC1;VASH2
MBNL2	5/299	0.04521396089 5433794	TRIM2;DLC1;DDIT4;ZBTB20;TNS3
MEIS3	5/299	0.04521396089 5433794	GNG2;DLC1;DDIT4;LAMB1;PRICKLE1
MESP2	5/299	0.04521396089 5433794	PCDHGA5;ALDH1A2;DLC1;DDIT4;ESR1
MLXIP	5/299	0.04521396089 5433794	DLC1;DDIT4;ZBTB20;PTPRJ;TNS3
MSX2	5/299	0.04521396089 5433794	DLC1;DDIT4;PRICKLE1;TNS3;ESR1
MXD1	5/299	0.04521396089 5433794	SLFN5;RBM47;DLC1;DDIT4;MX1
MXD4	5/299	0.04521396089 5433794	ST6GAL1;DLC1;DDIT4;ZBTB20;TNS3
MYB	5/299	0.04521396089 5433794	ASPM;DLC1;DDIT4;KIF14;ESR1
NFATC1	5/299	0.04521396089 5433794	ST6GAL1;DLC1;DDIT4;PTPRJ;TNS3
NFIX	5/299	0.04521396089 5433794	TRIM2;DLC1;DDIT4;ZBTB20;TNS3
NR2C1	5/299	0.04521396089 5433794	DLC1;DDIT4;ZBTB20;ESR1;SYNE2
NR6A1	5/299	0.04521396089 5433794	RBM47;NR5A2;DLC1;DDIT4;ZBTB20
NRL	5/299	0.04521396089 5433794	PCDHGA5;NR5A2;DLC1;DDIT4;ESR1
OLIG3	5/299	0.04521396089 5433794	NR5A2;ERBB4;ALDH1A2;DLC1;ESR1
ONECUT2	5/299	0.04521396089 5433794	NR5A2;DLC1;DDIT4;ZBTB20;ETV1
PAX1	5/299	0.04521396089 5433794	NR5A2;ERBB4;ALDH1A2;DLC1;ESR1
PAX5	5/299	0.04521396089 5433794	ST6GAL1;ERBB4;DLC1;TNS3;ESR1
PHOX2B	5/299	0.04521396089 5433794	PCDHGA5;NR5A2;ERBB4;ALDH1A2;DLC1
PITX2	5/299	0.04521396089 5433794	NR5A2;ALDH1A2;DLC1;DDIT4;ETV1
PITX3	5/299	0.04521396089 5433794	PCDHGA5;NR5A2;DLC1;DDIT4;ESR1
PLAGL1	5/299	0.04521396089 5433794	DLC1;DDIT4;ZBTB20;LAMB1;TNS3
PLXNA2	5/299	0.04521396089 5433794	DLC1;DDIT4;ZBTB20;PTPRJ;TNS3
POGK	5/299	0.04521396089 5433794	TRIM2;DLC1;DDIT4;ZBTB20;TNS3
POU2F2	5/299	0.04521396089 5433794	ARHGAP30;DLC1;DDIT4;MX1;PTPN6
PRDM10	5/299	0.04521396089 5433794	PLCB4;DLC1;ZBTB20;TNS3;SYNE2

PRDM14	5/299	0.04521396089 5433794	NR5A2;DLC1;VASH2;TNS3;ESR1
PRDM15	5/299	0.04521396089 5433794	HIP1;DLC1;DDIT4;TNS3;SYNE2
PRDM6	5/299	0.04521396089 5433794	NR5A2;ALDH1A2;DLC1;PRICKLE1;ESR1
PRDM8	5/299	0.04521396089 5433794	PCDHGA5;GNG2;DLC1;DDIT4;SPRY1
RAPGEF3	5/299	0.04521396089 5433794	DLC1;DDIT4;SPRY1;TNS3;JDP2
RAPGEF4	5/299	0.04521396089 5433794	PLCB4;TRIM2;DLC1;DDIT4;ZBTB20
RBM20	5/299	0.04521396089 5433794	ALDH1A2;DLC1;DDIT4;PRICKLE1;TNS3
RERE	5/299	0.04521396089 5433794	DLC1;DDIT4;ZBTB20;TNS3;SYNE2
RFX4	5/299	0.04521396089 5433794	MEGF10;DLC1;DDIT4;SRGAP3;ESR1
RGS6	5/299	0.04521396089 5433794	PCDHGA5;PLCB4;ERBB4;DLC1;ZBTB20
RGS7	5/299	0.04521396089 5433794	PCDHGA5;PLCB4;ERBB4;DLC1;RAPGEF4
RORC	5/299	0.04521396089 5433794	AR;NNMT;DLC1;DDIT4;ESR1
RXRG	5/299	0.04521396089 5433794	AR;DLC1;DDIT4;ETV1;ESR1
SCAPER	5/299	0.04521396089 5433794	TRIM2;DLC1;ZBTB20;TNS3;SYNE2
SETDB2	5/299	0.04521396089 5433794	SLFN5;DLC1;DDIT4;MX1;ZBTB20
SHOX	5/299	0.04521396089 5433794	AR;NR5A2;DLC1;DDIT4;ESR1
SIX1	5/299	0.04521396089 5433794	NNMT;ALDH1A2;DLC1;DDIT4;SPRY1
SMAD6	5/299	0.04521396089 5433794	DLC1;DDIT4;SPRY1;PRICKLE1;TNS3
SMARCA1	5/299	0.04521396089 5433794	TRIM2;DLC1;DDIT4;ETV1;LAMB1
SOX14	5/299	0.04521396089 5433794	NR5A2;ERBB4;ALDH1A2;DLC1;ESR1
SP140	5/299	0.04521396089 5433794	SLFN5;ARHGAP30;DLC1;MX1;PTPN6
SP5	5/299	0.04521396089 5433794	NR5A2;ALDH1A2;DLC1;DDIT4;PRICKLE1
SRY	5/299	0.04521396089 5433794	AR;NR5A2;MEGF10;DLC1;ESR1
TBX1	5/299	0.04521396089 5433794	ALDH1A2;DLC1;DDIT4;PRICKLE1;ESR1
TBX15	5/299	0.04521396089 5433794	NNMT;NR5A2;ALDH1A2;DLC1;LAMB1
TBX18	5/299	0.04521396089 5433794	PCDHGA5;SEMA3D;ALDH1A2;DLC1;TNS3
TBX22	5/299	0.04521396089 5433794	AR;SEMA3D;ERBB4;DLC1;SLITRK6
TBX4	5/299	0.04521396089 5433794	NR5A2;ALDH1A2;DLC1;TNS3;ESR1
TBX5	5/299	0.04521396089 5433794	PCDHGA5;ALDH1A2;DLC1;ETV1;ESR1
TCF4	5/299	0.04521396089	DLC1;DDIT4;ZBTB20;ETV1;TNS3

		5433794	
TCF7	5/299	0.04521396089 5433794	SLFN5;ST6GAL1;DLC1;DDIT4;SPRY1
TCFL5	5/299	0.04521396089 5433794	ASPM;RBM47;TRIM2;DLC1;DDIT4
TGIF2	5/299	0.04521396089 5433794	ASPM;DLC1;DDIT4;SPRY1;TNS3
TGIF2LX	5/299	0.04521396089 5433794	AR;NR5A2;SEMA3D;ETV1;ESR1
TOX3	5/299	0.04521396089 5433794	AR;TRIM2;DLC1;DDIT4;ETV1
TP73	5/299	0.04521396089 5433794	NR5A2;ERBB4;DLC1;DDIT4;ESR1
TRIM3	5/299	0.04521396089 5433794	TRIM2;DLC1;DDIT4;ETV1;TNS3
TRPS1	5/299	0.04521396089 5433794	DLC1;DDIT4;ZBTB20;ETV1;TNS3
VDR	5/299	0.04521396089 5433794	AR;NNMT;DLC1;DDIT4;ESR1
VENTX	5/299	0.04521396089 5433794	RBM47;NR5A2;DLC1;DDIT4;MX1
VSX1	5/299	0.04521396089 5433794	AR;PCDHGA5;NR5A2;DLC1;ESR1
WNT8B	5/299	0.04521396089 5433794	NR5A2;ERBB4;ALDH1A2;DLC1;DDIT4
YY2	5/299	0.04521396089 5433794	AR;ASPM;DLC1;DDIT4;ETV1
ZBED2	5/299	0.04521396089 5433794	ASPM;DLC1;DDIT4;MX1;KIF14
ZBTB16	5/299	0.04521396089 5433794	AR;NNMT;DLC1;DDIT4;ZBTB20
ZBTB20	5/299	0.04521396089 5433794	TRIM2;DLC1;DDIT4;TNS3;SYNE2
ZBTB40	5/299	0.04521396089 5433794	NR5A2;DDIT4;ZBTB20;TNS3;SYNE2
ZBTB46	5/299	0.04521396089 5433794	HIP1;DLC1;DDIT4;TNS3;JDP2
ZDHHC19	5/299	0.04521396089 5433794	RBM47;DLC1;DDIT4;FAM20A;TNS3
ZEB2	5/299	0.04521396089 5433794	GNG2;DLC1;DDIT4;ZBTB20;TNS3
ZFHX3	5/299	0.04521396089 5433794	DLC1;DDIT4;ZBTB20;TNS3;SYNE2
ZFP3	5/299	0.04521396089 5433794	SLFN5;DLC1;DDIT4;MX1;ZBTB20
ZFP30	5/299	0.04521396089 5433794	TRIM2;DLC1;DDIT4;PRICKLE1;IKZF2
ZFP41	5/299	0.04521396089 5433794	NEURL1B;ST6GAL1;HIP1;DLC1;DDIT4
ZFP64	5/299	0.04521396089 5433794	PLCB4;TRIM2;DLC1;DDIT4;TNS3
ZFP90	5/299	0.04521396089 5433794	ST6GAL1;TRIM2;DLC1;DDIT4;JDP2
ZHX2	5/299	0.04521396089 5433794	ST6GAL1;DLC1;DDIT4;ZBTB20;TNS3
ZKSCAN3	5/299	0.04521396089 5433794	ST6GAL1;DLC1;DDIT4;ZBTB20;SYNE2
ZKSCAN4	5/299	0.04521396089 5433794	ST6GAL1;DLC1;DDIT4;SPRY1;TNS3

ZMAT3	5/299	0.04521396089 5433794	TRIM2;DLC1;DDIT4;ZBTB20;TNS3
ZNF107	5/299	0.04521396089 5433794	ASPM;DDIT4;MX1;KIF14;SYNE2
ZNF132	5/299	0.04521396089 5433794	PCDHGA5;DLC1;DDIT4;MX1;ZNF610
ZNF154	5/299	0.04521396089 5433794	PCDHGA5;DLC1;DDIT4;ZBTB20;IKZF2
ZNF165	5/299	0.04521396089 5433794	ASPM;RBM47;DDIT4;MX1;KIF14
ZNF174	5/299	0.04521396089 5433794	HIP1;PCDHGA5;DLC1;DDIT4;ESR1
ZNF175	5/299	0.04521396089 5433794	SLFN5;ST6GAL1;DLC1;DDIT4;TNS3
ZNF182	5/299	0.04521396089 5433794	ZMAT1;PLCB4;DLC1;DDIT4;ZBTB20
ZNF184	5/299	0.04521396089 5433794	ASPM;DLC1;DDIT4;KIF14;SPRY1
ZNF211	5/299	0.04521396089 5433794	DLC1;DDIT4;MX1;ZBTB20;SYNE2
ZNF23	5/299	0.04521396089 5433794	PCDHGA5;PLCB4;DLC1;DDIT4;ZBTB20
ZNF230	5/299	0.04521396089 5433794	ASPM;SLFN5;DLC1;DDIT4;ZNF546
ZNF256	5/299	0.04521396089 5433794	BEX2;DLC1;DDIT4;ZBTB20;ZNF610
ZNF257	5/299	0.04521396089 5433794	ASPM;ALDH1A2;DLC1;KIF14;ZNF610
ZNF268	5/299	0.04521396089 5433794	PLCB4;TRIM2;DDIT4;ZBTB20;SYNE2
ZNF280A	5/299	0.04521396089 5433794	ASPM;PCDHGA5;DLC1;DDIT4;KIF14
ZNF280C	5/299	0.04521396089 5433794	ASPM;PLCB4;TRIM2;DLC1;DDIT4
ZNF280D	5/299	0.04521396089 5433794	TRIM2;DLC1;DDIT4;ZBTB20;SYNE2
ZNF320	5/299	0.04521396089 5433794	SLFN5;DLC1;DDIT4;PRICKLE1;ZNF610
ZNF341	5/299	0.04521396089 5433794	DLC1;DDIT4;PTPRJ;ETV1;TNS3
ZNF362	5/299	0.04521396089 5433794	DLC1;DDIT4;ZBTB20;TNS3;JDP2
ZNF383	5/299	0.04521396089 5433794	SLFN5;DDIT4;ZBTB20;ZNF546;SPRY1
ZNF398	5/299	0.04521396089 5433794	RBM47;DLC1;DDIT4;VASH2;SYNE2
ZNF404	5/299	0.04521396089 5433794	SLFN5;ZMAT1;DLC1;DDIT4;ZNF610
ZNF417	5/299	0.04521396089 5433794	SLFN5;CLMN;DLC1;ZNF546;SYNE2
ZNF419	5/299	0.04521396089 5433794	SLFN5;DDIT4;MX1;ZBTB20;JDP2
ZNF432	5/299	0.04521396089 5433794	SLFN5;DLC1;DDIT4;MX1;ZBTB20
ZNF433	5/299	0.04521396089 5433794	SLFN5;PCDHGA5;DLC1;DDIT4;ZNF610
ZNF449	5/299	0.04521396089 5433794	SLFN5;ZMAT1;DLC1;DDIT4;ZBTB20
ZNF454	5/299	0.04521396089	PCDHGA5;ERBB4;DLC1;ETV1;ZNF610

		5433794	
ZNF461	5/299	0.04521396089 5433794	SLFN5;PCDHGA5;DLC1;DDIT4;ZNF610
ZNF467	5/299	0.04521396089 5433794	ST6GAL1;DLC1;DDIT4;ZBTB20;TNS3
ZNF486	5/299	0.04521396089 5433794	RBM47;DLC1;DDIT4;MX1;TNS3
ZNF487	5/299	0.04521396089 5433794	PCDHGA5;DLC1;DDIT4;ZBTB20;TNS3
ZNF516	5/299	0.04521396089 5433794	RBM47;DLC1;DDIT4;ZBTB20;TNS3
ZNF518A	5/299	0.04521396089 5433794	ASPM;TRIM2;DDIT4;ZBTB20;SYNE2
ZNF519	5/299	0.04521396089 5433794	ASPM;DDIT4;VASH2;KIF14;MAP2K6
ZNF527	5/299	0.04521396089 5433794	HIP1;PCDHGA5;DDIT4;ZNF546;LAMB1
ZNF536	5/299	0.04521396089 5433794	MEGF10;ERBB4;TRIM2;DLC1;ETV1
ZNF586	5/299	0.04521396089 5433794	C5;DDIT4;KIF14;PTPN6;SYNE2
ZNF595	5/299	0.04521396089 5433794	ASPM;ST6GAL1;DLC1;DDIT4;SYNE2
ZNF600	5/299	0.04521396089 5433794	SLFN5;DLC1;DDIT4;MX1;ZNF610
ZNF609	5/299	0.04521396089 5433794	HIP1;DDIT4;ZBTB20;TNS3;SYNE2
ZNF620	5/299	0.04521396089 5433794	ZNF704;DLC1;DDIT4;KIF14;MAP2K6
ZNF677	5/299	0.04521396089 5433794	AR;PCDHGA5;DLC1;ZBTB20;ZNF610
ZNF74	5/299	0.04521396089 5433794	HIP1;DLC1;DDIT4;ZBTB20;ESR1
ZNF749	5/299	0.04521396089 5433794	ASPM;DLC1;DDIT4;MX1;EXPH5
ZNF75A	5/299	0.04521396089 5433794	SLFN5;PCDHGA5;TRIM2;DLC1;DDIT4
ZNF772	5/299	0.04521396089 5433794	CLMN;DDIT4;ZBTB20;IKZF2;ZNF610
ZNF773	5/299	0.04521396089 5433794	SLFN5;CLMN;DLC1;DDIT4;JDP2
ZNF783	5/299	0.04521396089 5433794	RBM47;PCDHGA5;DLC1;DDIT4;ZBTB20
ZNF788	5/299	0.04521396089 5433794	ASPM;DLC1;DDIT4;MX1;KIF14
ZNF829	5/299	0.04521396089 5433794	BEX2;PCDHGA5;ZBTB20;IKZF2;ZNF610
ZNF835	5/299	0.04521396089 5433794	PCDHGA5;ST8SIA1;DLC1;TNS3;PDE9A
ZNF84	5/299	0.04521396089 5433794	TRIM2;DLC1;DDIT4;ZBTB20;SYNE2
ZNF841	5/299	0.04521396089 5433794	SLFN5;DLC1;DDIT4;ZBTB20;SYNE2
ZNF843	5/299	0.04521396089 5433794	PCDHGA5;ST8SIA1;DLC1;DDIT4;ZBTB20
ZNF98	5/299	0.04521396089 5433794	PCDHGA11;PCDHGA5;SEMA3D;DLC1;TNS3
ZSCAN1	5/299	0.04521396089 5433794	PCDHGA11;PCDHGA6;PCDHGA5;ALDH1A2;DLC 1

ZSCAN12	5/299	0.045213960895433794	TRIM2;DLC1;DDIT4;IKZF2;MAP2K6

Table S8. The 51 common upregulated genes common for MB and Mel Z cells forming VM phenotype are simultaneously regulated by 795 different transcription factors. Data were obtained by a search of corresponding genes in Enrichr Submissions TF-Gene Cooccurrence (https://maayanlab.cloud/Enrichr/enrich#, accessed on 24 December 2025).

Term	Overlap	Adjusted P-value	Genes
SNAI3	10/299	1.4575943960679035E-6	NGFR;CDKN1A;MT2A;ARC;SPHK1;SERPINE1;HMOX1;ADM;SERPINB8;CORO1A
TUT1	10/299	1.4575943960679035E-6	NXF1;CDKN1A;NUDC;POLR2A;CCDC86;ADM;PRPF19;SF1;SRM;BYSL
ZNF296	10/299	1.4575943960679035E-6	CDKN1A;MT2A;SERPINE1;CCDC86;HMOX1;ADM;ATF5;SERPINB8;CORO1A;SRM
ZNF593	10/299	1.4575943960679035E-6	LSM10;CDKN1A;MT2A;NUDC;GAR1;CCDC86;HMOX1;ADM;SRM;BYSL
BATF3	9/299	1.0535571784712402E-5	CDKN1A;MT2A;SPHK1;SERPINE1;HMOX1;SLAMF7;ADM;SERPINB8;CORO1A
HES2	9/299	1.0535571784712402E-5	NGFR;CDKN1A;MT2A;ARC;SPHK1;SERPINE1;HMOX1;ADM;SERPINB8
MAFK	9/299	1.0535571784712402E-5	CDKN1A;MT2A;ARC;SPHK1;SERPINE1;OSGIN1;HMOX1;ADM;SERPINB8
RELL2	9/299	1.0535571784712402E-5	CDKN1A;MT2A;ARC;KCNC3;PDE2A;HMOX1;ADM;SERPINB8;CORO1A
SP6	9/299	1.0535571784712402E-5	NGFR;CDKN1A;MT2A;ARC;SPHK1;SERPINE1;HMOX1;ADM;SERPINB8
ATF3	8/299	3.3829109397827925E-5	CDKN1A;MT2A;ARC;SPHK1;SERPINE1;HMOX1;ADM;SERPINB8
CXXC1	8/299	3.3829109397827925E-5	NXF1;CDKN1A;NUDC;POLR2A;PRPF19;SERPINB8;CORO1A;SRM
EGR2	8/299	3.3829109397827925E-5	CDKN1A;MT2A;ARC;SPHK1;SERPINE1;HMOX1;ADM;SERPINB8
EGR3	8/299	3.3829109397827925E-5	CDKN1A;MT2A;ARC;SPHK1;SERPINE1;HMOX1;ADM;SERPINB8
EGR4	8/299	3.3829109397827925E-5	CDKN1A;MT2A;ARC;SPHK1;SERPINE1;HMOX1;ADM;SERPINB8
FOSL1	8/299	3.3829109397827925E-5	CDKN1A;MT2A;SPHK1;SERPINE1;CCDC86;HMOX1;ADM;SERPINB8
FOXJ1	8/299	3.3829109397827925E-5	NGFR;CDKN1A;MT2A;ARC;SPHK1;SERPINE1;ADM;SERPINB8
FOXS1	8/299	3.3829109397827925E-5	CDKN1A;MT2A;SPHK1;SERPINE1;HMOX1;ADM;S1PR3;SERPINB8
HES7	8/299	3.3829109397827925E-5	NGFR;CDKN1A;MT2A;ARC;SPHK1;SERPINE1;HMOX1;ADM
HILS1	8/299	3.3829109397827925E-5	NGFR;CDKN1A;MT2A;SPHK1;SERPINE1;ADM;PHACTR1;SERPINB8
MAFA	8/299	3.3829109397827925E-5	NGFR;CDKN1A;MT2A;ARC;KCNC3;SPHK1;ADM;SERPINB8
MAFF	8/299	3.3829109397827925E-5	CDKN1A;MT2A;ARC;SPHK1;SERPINE1;HMOX1;ADM;SERPINB8
PLEKHA4	8/299	3.3829109397827925E-5	NGFR;CDKN1A;MT2A;SPHK1;SERPINE1;HMOX1;ADM;SERPINB8
PLXNB3	8/299	3.3829109397827925E-5	NGFR;CDKN1A;MT2A;ARC;SERPINE1;HMOX1;ADM;SERPINB8
POU2F2	8/299	3.38291093978279	CDKN1A;MT2A;SERPINE1;PDE2A;HM

		25E-5	OX1;SLAMF7;SERPINB8;CORO1A
POU3F1	8/299	3.3829109397827925E-5	NGFR;CDKN1A;MT2A;ARC;KCNC3;PDE2A;ADM;SERPINB8
PPP1R10	8/299	3.3829109397827925E-5	NXF1;CDKN1A;MT2A;POLR2A;SERPINE1;HMOX1;ADM;SF1
RAPGEF3	8/299	3.3829109397827925E-5	CDKN1A;MT2A;SPHK1;SERPINE1;PDE2A;HMOX1;ADM;SERPINB8
RELB	8/299	3.3829109397827925E-5	CDKN1A;MT2A;SPHK1;SERPINE1;HMOX1;ADM;SERPINB8;CORO1A
SNAI1	8/299	3.3829109397827925E-5	CDKN1A;MT2A;SPHK1;SERPINE1;HMOX1;ADM;S1PR3;SERPINB8
SP110	8/299	3.3829109397827925E-5	CDKN1A;MT2A;SERPINE1;HMOX1;SLAMF7;ADM;SERPINB8;CORO1A
STAT4	8/299	3.3829109397827925E-5	CDKN1A;MT2A;SERPINE1;HMOX1;SLAMF7;ADM;SERPINB8;CORO1A
TEAD4	8/299	3.3829109397827925E-5	CDKN1A;MT2A;SPHK1;SERPINE1;HMOX1;ADM;SERPINB8;SRM
ZNF324	8/299	3.3829109397827925E-5	NXF1;CDKN1A;SPHK1;OSGIN1;HMOX1;ADM;SERPINB8;CORO1A
ZNF442	8/299	3.3829109397827925E-5	NGFR;CDKN1A;ARC;SPHK1;SERPINE1;HMOX1;ADM;SERPINB8
ZNF473	8/299	3.3829109397827925E-5	CDKN1A;SPHK1;SERPINE1;OSGIN1;CCDC86;HMOX1;ADM;SERPINB8
ZNF574	8/299	3.3829109397827925E-5	CDKN1A;NUDC;POLR2A;CCDC86;PRPF19;SF1;SRM;BYSL
ZNF622	8/299	3.3829109397827925E-5	CDKN1A;MT2A;NUDC;PSMD2;HMOX1;ADM;SERPINB8;BYSL
ZNF746	8/299	3.3829109397827925E-5	CDKN1A;MT2A;POLR2A;SERPINE1;OSGIN1;HMOX1;ADM;SERPINB8
ZNF777	8/299	3.3829109397827925E-5	CDKN1A;POLR2A;PRCC;CCDC86;HMOX1;PRPF19;ATF5;SERPINB8
ZNF830	8/299	3.3829109397827925E-5	NXF1;POLR2A;GAR1;CCDC86;HMOX1;ADM;SF1;BYSL
ARNTL2	7/299	1.4828502984283866E-4	CDKN1A;MT2A;SPHK1;SERPINE1;HMOX1;ADM;SERPINB8
ATF5	7/299	1.4828502984283866E-4	CDKN1A;MT2A;SERPINE1;HMOX1;ADM;SERPINB8;SRM
ATOH8	7/299	1.4828502984283866E-4	CDKN1A;MT2A;SPHK1;SERPINE1;HMOX1;ADM;SERPINB8
BATF	7/299	1.4828502984283866E-4	CDKN1A;MT2A;SERPINE1;HMOX1;SLAMF7;ADM;CORO1A
BCL6B	7/299	1.4828502984283866E-4	CDKN1A;SERPINE1;PDE2A;HMOX1;ADM;S1PR3;SERPINB8
BRD9	7/299	1.4828502984283866E-4	NXF1;CDKN1A;NUDC;POLR2A;PRPF19;SERPINB8;SF1
CEBPA	7/299	1.4828502984283866E-4	CDKN1A;MT2A;SERPINE1;HMOX1;ADM;SERPINB8;CORO1A
CEBPE	7/299	1.4828502984283866E-4	CDKN1A;SPHK1;SERPINE1;HMOX1;ADM;SERPINB8;CORO1A
CEBPG	7/299	1.4828502984283866E-4	CDKN1A;MT2A;SERPINE1;HMOX1;ADM;ATF5;SERPINB8
DDIT3	7/299	1.4828502984283866E-4	CDKN1A;MT2A;SERPINE1;HMOX1;ADM;ATF5;SERPINB8
DHX34	7/299	1.4828502984283866E-4	CDKN1A;MT2A;POLR2A;CCDC86;HMOX1;SERPINB8;SRM
DUS3L	7/299	1.4828502984283866E-4	CDKN1A;MT2A;CCDC86;PRPF19;SERPINB8;SRM;BYSL
E2F4	7/299	1.4828502984283866E-4	SF3B4;CDKN1A;MT2A;NUDC;POLR2A;CCDC86;SRM

EGR1	7/299	1.4828502984283866E-4	CDKN1A;MT2A;ARC;SERPINE1;HMOX1;ADM;SERPINB8
EOMES	7/299	1.4828502984283866E-4	CDKN1A;MT2A;HMOX1;SLAMF7;ADM;SERPINB8;CORO1A
ETV4	7/299	1.4828502984283866E-4	CDKN1A;MT2A;SPHK1;SERPINE1;HMOX1;ADM;SERPINB8
FOS	7/299	1.4828502984283866E-4	CDKN1A;MT2A;ARC;SERPINE1;HMOX1;ADM;SERPINB8
FOSB	7/299	1.4828502984283866E-4	CDKN1A;MT2A;ARC;SERPINE1;HMOX1;ADM;SERPINB8
FOXC2	7/299	1.4828502984283866E-4	CDKN1A;MT2A;SPHK1;SERPINE1;ADM;S1PR3;SERPINB8
FOXO6	7/299	1.4828502984283866E-4	NGFR;CDKN1A;MT2A;ARC;PDE2A;ADM;SERPINB8
GLI1	7/299	1.4828502984283866E-4	NGFR;CDKN1A;SPHK1;SERPINE1;HMOX1;ADM;SERPINB8
HES4	7/299	1.4828502984283866E-4	CDKN1A;MT2A;SPHK1;SERPINE1;HMOX1;ADM;SERPINB8
HHEX	7/299	1.4828502984283866E-4	CDKN1A;MT2A;SERPINE1;HMOX1;ADM;SERPINB8;CORO1A
HOXB7	7/299	1.4828502984283866E-4	CDKN1A;MT2A;SPHK1;SERPINE1;HMOX1;ADM;SERPINB8
HOXB9	7/299	1.4828502984283866E-4	NGFR;CDKN1A;MT2A;SPHK1;SERPINE1;ADM;SERPINB8
IFI16	7/299	1.4828502984283866E-4	CDKN1A;MT2A;SERPINE1;HMOX1;ADM;SERPINB8;CORO1A
JUNB	7/299	1.4828502984283866E-4	CDKN1A;MT2A;ARC;SERPINE1;HMOX1;ADM;SERPINB8
KLF10	7/299	1.4828502984283866E-4	CDKN1A;MT2A;ARC;SERPINE1;HMOX1;ADM;SERPINB8
KLF2	7/299	1.4828502984283866E-4	CDKN1A;MT2A;SERPINE1;HMOX1;ADM;SERPINB8;CORO1A
LARP6	7/299	1.4828502984283866E-4	CDKN1A;MT2A;SPHK1;SERPINE1;HMOX1;ADM;SERPINB8
LBX2	7/299	1.4828502984283866E-4	NGFR;CDKN1A;MT2A;SERPINE1;OSGIN1;ADM;SERPINB8
LHX6	7/299	1.4828502984283866E-4	NGFR;CDKN1A;ARC;SERPINE1;PDE2A;ADM;SERPINB8
MAFG	7/299	1.4828502984283866E-4	CDKN1A;MT2A;SERPINE1;OSGIN1;HMOX1;ADM;SERPINB8
MESP2	7/299	1.4828502984283866E-4	NGFR;CDKN1A;SPHK1;SERPINE1;HMOX1;ADM;SERPINB8
MSC	7/299	1.4828502984283866E-4	CDKN1A;MT2A;SPHK1;SERPINE1;HMOX1;ADM;SERPINB8
NFKB2	7/299	1.4828502984283866E-4	CDKN1A;MT2A;SPHK1;SERPINE1;HMOX1;ADM;SERPINB8
NKRF	7/299	1.4828502984283866E-4	CDKN1A;GAR1;CCDC86;PRPF19;SERPINB8;SRM;BYSL
NPAS1	7/299	1.4828502984283866E-4	NGFR;CDKN1A;MT2A;ARC;SERPINE1;ADM;SERPINB8
NR1D1	7/299	1.4828502984283866E-4	CDKN1A;MT2A;SERPINE1;OSGIN1;HMOX1;ADM;SERPINB8
NR1H3	7/299	1.4828502984283866E-4	CDKN1A;MT2A;SPHK1;SERPINE1;HMOX1;ADM;SERPINB8
NR2F6	7/299	1.4828502984283866E-4	CDKN1A;MT2A;SERPINE1;HMOX1;SERPINB8;CORO1A;SRM
NR4A1	7/299	1.4828502984283866E-4	CDKN1A;MT2A;ARC;SERPINE1;HMOX1;ADM;SERPINB8
NR4A2	7/299	1.48285029842838	CDKN1A;MT2A;ARC;SERPINE1;HMOX

		66E-4	1;ADM;SERPINB8
NR4A3	7/299	1.4828502984283866E-4	CDKN1A;MT2A;ARC;SERPINE1;HMOX1;ADM;SERPINB8
PBX4	7/299	1.4828502984283866E-4	CDKN1A;MT2A;SPHK1;SERPINE1;HMOX1;ADM;SERPINB8
PLEK2	7/299	1.4828502984283866E-4	CDKN1A;MT2A;SPHK1;SERPINE1;HMOX1;ADM;SERPINB8
PPP1R13L	7/299	1.4828502984283866E-4	CDKN1A;MT2A;SPHK1;SERPINE1;HMOX1;ADM;SERPINB8
PRDM1	7/299	1.4828502984283866E-4	CDKN1A;MT2A;SERPINE1;HMOX1;SLAMF7;ADM;SERPINB8
PRRX2	7/299	1.4828502984283866E-4	CDKN1A;MT2A;SPHK1;SERPINE1;HMOX1;ADM;SERPINB8
RFX2	7/299	1.4828502984283866E-4	CDKN1A;MT2A;SPHK1;SERPINE1;HMOX1;ADM;SERPINB8
RGS11	7/299	1.4828502984283866E-4	CDKN1A;MT2A;ARC;PDE2A;HMOX1;ADM;SERPINB8
RUNX3	7/299	1.4828502984283866E-4	CDKN1A;MT2A;SERPINE1;HMOX1;SLAMF7;ADM;CORO1A
SLC22A4	7/299	1.4828502984283866E-4	CDKN1A;MT2A;SPHK1;SERPINE1;HMOX1;ADM;SERPINB8
SMAD7	7/299	1.4828502984283866E-4	CDKN1A;MT2A;SPHK1;SERPINE1;HMOX1;ADM;SERPINB8
SNAPC4	7/299	1.4828502984283866E-4	CDKN1A;MT2A;POLR2A;CCDC86;PRPF19;SERPINB8;SF1
SOX8	7/299	1.4828502984283866E-4	NGFR;CDKN1A;MT2A;ARC;SERPINE1;ADM;SERPINB8
TBX2	7/299	1.4828502984283866E-4	NGFR;CDKN1A;SERPINE1;HMOX1;ADM;S1PR3;SERPINB8
TCF7	7/299	1.4828502984283866E-4	CDKN1A;MT2A;SERPINE1;HMOX1;ADM;SERPINB8;CORO1A
TCFL5	7/299	1.4828502984283866E-4	CDKN1A;MT2A;SERPINE1;HMOX1;ADM;ATF5;SERPINB8
THAP3	7/299	1.4828502984283866E-4	LSM10;CDKN1A;MT2A;NUDC;OSGIN1;SERPINB8;SRM
TSC22D3	7/299	1.4828502984283866E-4	CDKN1A;MT2A;SERPINE1;HMOX1;ADM;SERPINB8;CORO1A
VDR	7/299	1.4828502984283866E-4	CDKN1A;MT2A;SPHK1;SERPINE1;HMOX1;ADM;SERPINB8
YBX2	7/299	1.4828502984283866E-4	NGFR;CDKN1A;MT2A;ARC;HMOX1;ADM;SERPINB8
ZBP1	7/299	1.4828502984283866E-4	CDKN1A;MT2A;SERPINE1;HMOX1;SLAMF7;ADM;CORO1A
ZNF202	7/299	1.4828502984283866E-4	CDKN1A;SERPINE1;CCDC86;HMOX1;ADM;SERPINB8;BYSL
ZNF239	7/299	1.4828502984283866E-4	CDKN1A;MT2A;SERPINE1;CCDC86;HMOX1;ADM;SERPINB8
ZNF274	7/299	1.4828502984283866E-4	CDKN1A;SERPINE1;HMOX1;ADM;ATF5;SERPINB8;CORO1A
ZNF280A	7/299	1.4828502984283866E-4	NGFR;CDKN1A;SERPINE1;HMOX1;ADM;SERPINB8;CORO1A
ZNF282	7/299	1.4828502984283866E-4	CDKN1A;CCDC86;ADM;ATF5;SERPINB8;SF1;SRM
ZNF331	7/299	1.4828502984283866E-4	CDKN1A;MT2A;SERPINE1;HMOX1;ADM;PHACTR1;SERPINB8
ZNF408	7/299	1.4828502984283866E-4	NXF1;CDKN1A;SPHK1;OSGIN1;HMOX1;ADM;SERPINB8
ZNF697	7/299	1.4828502984283866E-4	CDKN1A;MT2A;SPHK1;SERPINE1;HMOX1;ADM;SERPINB8

ZNF784	7/299	1.48285029842838 66E-4	CDKN1A;MT2A;OSGIN1;HMOX1;ADM; ATF5;SERPINB8
AHR	6/299	6.36224056035047 E-4	CDKN1A;MT2A;SERPINE1;HMOX1;AD M;SERPINB8
ANAPC2	6/299	6.36224056035047 E-4	CDKN1A;NUDC;POLR2A;PSMD2;PRPF 19;SF1
ARID5B	6/299	6.36224056035047 E-4	CDKN1A;MT2A;SERPINE1;HMOX1;AD M;SERPINB8
ARNTL	6/299	6.36224056035047 E-4	CDKN1A;MT2A;SERPINE1;HMOX1;AD M;SERPINB8
ASCL2	6/299	6.36224056035047 E-4	CDKN1A;MT2A;SPHK1;SERPINE1;AD M;SERPINB8
ASCL3	6/299	6.36224056035047 E-4	NGFR;CDKN1A;ARC;SERPINE1;HMOX 1;ADM
BATF2	6/299	6.36224056035047 E-4	CDKN1A;MT2A;SERPINE1;HMOX1;SL AMF7;ADM
BCL6	6/299	6.36224056035047 E-4	CDKN1A;MT2A;SERPINE1;HMOX1;AD M;SERPINB8
BMP2	6/299	6.36224056035047 E-4	CDKN1A;MT2A;SERPINE1;HMOX1;AD M;SERPINB8
CD36	6/299	6.36224056035047 E-4	CDKN1A;MT2A;SERPINE1;HMOX1;AD M;SERPINB8
CEBPB	6/299	6.36224056035047 E-4	CDKN1A;MT2A;SERPINE1;HMOX1;AD M;SERPINB8
CENPT	6/299	6.36224056035047 E-4	CDKN1A;MT2A;HMOX1;SERPINB8;CO RO1A;SRM
CPSF4	6/299	6.36224056035047 E-4	CDKN1A;NUDC;PSMD2;CCDC86;HMOX 1;PRPF19
CREB3	6/299	6.36224056035047 E-4	CDKN1A;MT2A;SERPINE1;HMOX1;AD M;SERPINB8
CREB3L1	6/299	6.36224056035047 E-4	CDKN1A;MT2A;SERPINE1;HMOX1;AD M;SERPINB8
CREB3L2	6/299	6.36224056035047 E-4	CDKN1A;MT2A;SERPINE1;HMOX1;AD M;SERPINB8
CREB5	6/299	6.36224056035047 E-4	CDKN1A;MT2A;SERPINE1;HMOX1;AD M;SERPINB8
CREM	6/299	6.36224056035047 E-4	CDKN1A;MT2A;SERPINE1;HMOX1;AD M;SERPINB8
CSDC2	6/299	6.36224056035047 E-4	CDKN1A;ARC;SERPINE1;PDE2A;ADM ;SERPINB8
DBP	6/299	6.36224056035047 E-4	CDKN1A;MT2A;SERPINE1;HMOX1;AD M;SERPINB8
DEPDC7	6/299	6.36224056035047 E-4	CDKN1A;MT2A;SERPINE1;HMOX1;AD M;SERPINB8
DLX2	6/299	6.36224056035047 E-4	CDKN1A;SPHK1;SERPINE1;HMOX1;A DM;SERPINB8
DLX3	6/299	6.36224056035047 E-4	NGFR;CDKN1A;SPHK1;SERPINE1;AD M;SERPINB8
DPF1	6/299	6.36224056035047 E-4	CDKN1A;ARC;SERPINE1;PDE2A;SER PINB8;CORO1A
DUSP12	6/299	6.36224056035047 E-4	CDKN1A;MT2A;CCDC86;PRPF19;SER PINB8;SRM
ELF3	6/299	6.36224056035047 E-4	CDKN1A;MT2A;SERPINE1;HMOX1;AD M;SERPINB8
ELF4	6/299	6.36224056035047 E-4	CDKN1A;MT2A;SERPINE1;HMOX1;AD M;SERPINB8
ELK3	6/299	6.36224056035047 E-4	CDKN1A;MT2A;SERPINE1;HMOX1;AD M;SERPINB8
EPAS1	6/299	6.36224056035047	CDKN1A;MT2A;SERPINE1;HMOX1;AD

		E-4	M;SERPINB8
ERF	6/299	6.36224056035047 E-4	CDKN1A;MT2A;SERPINE1;HMOX1;AD M;SERPINB8
ETS1	6/299	6.36224056035047 E-4	CDKN1A;SERPINE1;HMOX1;ADM;SER PINB8;CORO1A
ETS2	6/299	6.36224056035047 E-4	CDKN1A;MT2A;SERPINE1;HMOX1;AD M;SERPINB8
ETV3L	6/299	6.36224056035047 E-4	CDKN1A;SPHK1;SERPINE1;SLAMF7; ADM;SERPINB8
ETV5	6/299	6.36224056035047 E-4	CDKN1A;MT2A;SERPINE1;HMOX1;AD M;SERPINB8
ETV7	6/299	6.36224056035047 E-4	CDKN1A;MT2A;SERPINE1;HMOX1;SL AMF7;ADM
FARSA	6/299	6.36224056035047 E-4	NUDC;PSMD2;CCDC86;PRPF19;SRM; BYSL
FOSL2	6/299	6.36224056035047 E-4	CDKN1A;MT2A;SERPINE1;HMOX1;AD M;SERPINB8
FOXN4	6/299	6.36224056035047 E-4	NGFR;CDKN1A;MT2A;ARC;ADM;SERP INB8
FOXO1	6/299	6.36224056035047 E-4	CDKN1A;MT2A;SERPINE1;HMOX1;AD M;SERPINB8
FOXO3	6/299	6.36224056035047 E-4	CDKN1A;MT2A;SERPINE1;HMOX1;AD M;SERPINB8
FOXO4	6/299	6.36224056035047 E-4	CDKN1A;MT2A;SERPINE1;HMOX1;AD M;SERPINB8
FOXQ1	6/299	6.36224056035047 E-4	CDKN1A;MT2A;SERPINE1;HMOX1;AD M;SERPINB8
GATA3	6/299	6.36224056035047 E-4	CDKN1A;MT2A;SERPINE1;HMOX1;AD M;SERPINB8
GBX2	6/299	6.36224056035047 E-4	NGFR;CDKN1A;ARC;SERPINE1;ADM; SERPINB8
GLIS2	6/299	6.36224056035047 E-4	CDKN1A;SPHK1;SERPINE1;HMOX1;A DM;SERPINB8
GRHL1	6/299	6.36224056035047 E-4	CDKN1A;MT2A;SERPINE1;HMOX1;AD M;SERPINB8
HES1	6/299	6.36224056035047 E-4	CDKN1A;MT2A;SERPINE1;HMOX1;AD M;SERPINB8
HEY1	6/299	6.36224056035047 E-4	CDKN1A;MT2A;SERPINE1;HMOX1;AD M;SERPINB8
HEYL	6/299	6.36224056035047 E-4	NGFR;CDKN1A;SERPINE1;ADM;S1PR 3;SERPINB8
HIC1	6/299	6.36224056035047 E-4	CDKN1A;SPHK1;SERPINE1;ADM;S1P R3;SERPINB8
HIF3A	6/299	6.36224056035047 E-4	CDKN1A;MT2A;SERPINE1;PDE2A;AD M;SERPINB8
HIST1H1T	6/299	6.36224056035047 E-4	CDKN1A;MT2A;SPHK1;SERPINE1;AD M;SERPINB8
HLX	6/299	6.36224056035047 E-4	CDKN1A;SPHK1;SERPINE1;HMOX1;A DM;SERPINB8
HMGA2	6/299	6.36224056035047 E-4	CDKN1A;MT2A;SERPINE1;HMOX1;AD M;SERPINB8
HOPX	6/299	6.36224056035047 E-4	CDKN1A;MT2A;SERPINE1;HMOX1;AD M;SERPINB8
HOXB6	6/299	6.36224056035047 E-4	CDKN1A;MT2A;SERPINE1;HMOX1;AD M;SERPINB8
HOXC10	6/299	6.36224056035047 E-4	LRRC15;CDKN1A;SERPINE1;HMOX1; ADM;SERPINB8
HOXC13	6/299	6.36224056035047 E-4	LRRC15;CDKN1A;SERPINE1;HMOX1; ADM;SERPINB8

HOXC6	6/299	6.36224056035047E-4	CDKN1A;MT2A;SERPINE1;HMOX1;ADM;SERPINB8
ID1	6/299	6.36224056035047E-4	CDKN1A;MT2A;SERPINE1;HMOX1;ADM;SERPINB8
ID2	6/299	6.36224056035047E-4	CDKN1A;MT2A;SERPINE1;HMOX1;ADM;SERPINB8
ID3	6/299	6.36224056035047E-4	CDKN1A;MT2A;SERPINE1;HMOX1;ADM;SERPINB8
IGHM	6/299	6.36224056035047E-4	CDKN1A;MT2A;SERPINE1;HMOX1;SLAMF7;CORO1A
IRF1	6/299	6.36224056035047E-4	CDKN1A;MT2A;SERPINE1;HMOX1;ADM;CORO1A
IRF7	6/299	6.36224056035047E-4	CDKN1A;MT2A;SERPINE1;HMOX1;ADM;CORO1A
IRF9	6/299	6.36224056035047E-4	CDKN1A;MT2A;SERPINE1;HMOX1;ADM;SERPINB8
JDP2	6/299	6.36224056035047E-4	CDKN1A;MT2A;SERPINE1;HMOX1;ADM;SERPINB8
JUN	6/299	6.36224056035047E-4	CDKN1A;MT2A;SERPINE1;HMOX1;ADM;SERPINB8
KLF15	6/299	6.36224056035047E-4	CDKN1A;MT2A;SERPINE1;HMOX1;ADM;SERPINB8
KLF4	6/299	6.36224056035047E-4	CDKN1A;MT2A;SERPINE1;HMOX1;ADM;SERPINB8
KLF5	6/299	6.36224056035047E-4	CDKN1A;MT2A;SERPINE1;HMOX1;ADM;SERPINB8
KLF6	6/299	6.36224056035047E-4	CDKN1A;MT2A;SERPINE1;HMOX1;ADM;SERPINB8
KLF9	6/299	6.36224056035047E-4	CDKN1A;MT2A;SERPINE1;HMOX1;ADM;SERPINB8
LENG9	6/299	6.36224056035047E-4	CDKN1A;MT2A;OSGIN1;HMOX1;ADM;SERPINB8
MAFB	6/299	6.36224056035047E-4	CDKN1A;MT2A;SERPINE1;HMOX1;ADM;SERPINB8
MBNL3	6/299	6.36224056035047E-4	CDKN1A;MT2A;SERPINE1;HMOX1;ADM;SERPINB8
MEF2B	6/299	6.36224056035047E-4	CDKN1A;SPHK1;HMOX1;ADM;ATF5;SERPINB8
MET	6/299	6.36224056035047E-4	CDKN1A;MT2A;SERPINE1;HMOX1;ADM;SERPINB8
MGMT	6/299	6.36224056035047E-4	CDKN1A;MT2A;SERPINE1;HMOX1;ADM;SERPINB8
MLXIPL	6/299	6.36224056035047E-4	CDKN1A;MT2A;SERPINE1;HMOX1;ADM;SERPINB8
MSX1	6/299	6.36224056035047E-4	CDKN1A;MT2A;SPHK1;SERPINE1;ADM;SERPINB8
MXD1	6/299	6.36224056035047E-4	CDKN1A;MT2A;SERPINE1;HMOX1;ADM;SERPINB8
MYC	6/299	6.36224056035047E-4	CDKN1A;MT2A;SERPINE1;HMOX1;ADM;SRM
NFE2	6/299	6.36224056035047E-4	CDKN1A;MT2A;SERPINE1;HMOX1;ADM;CORO1A
NFE2L3	6/299	6.36224056035047E-4	CDKN1A;MT2A;SERPINE1;HMOX1;ADM;SERPINB8
NFIL3	6/299	6.36224056035047E-4	CDKN1A;MT2A;SERPINE1;HMOX1;ADM;SERPINB8
NFKB1	6/299	6.36224056035047E-4	CDKN1A;MT2A;SERPINE1;HMOX1;ADM;SERPINB8
NOC4L	6/299	6.36224056035047	NUDC;GAR1;CCDC86;PRPF19;SRM;B

		E-4	YSL
NPAS2	6/299	6.36224056035047 E-4	CDKN1A;MT2A;SERPINE1;PDE2A;ADM;SERPINB8
NPAS4	6/299	6.36224056035047 E-4	CDKN1A;ARC;SERPINE1;PDE2A;ADM;SERPINB8
NR0B1	6/299	6.36224056035047 E-4	CDKN1A;MT2A;SERPINE1;HMOX1;ADM;SERPINB8
NR0B2	6/299	6.36224056035047 E-4	CDKN1A;SERPINE1;OSGIN1;HMOX1;ADM;SERPINB8
NUFIP1	6/299	6.36224056035047 E-4	CDKN1A;SERPINE1;CCDC86;SERPINB8;SRM;BYSL
OLIG1	6/299	6.36224056035047 E-4	CDKN1A;MT2A;ARC;HMOX1;ADM;SERPINB8
PARP12	6/299	6.36224056035047 E-4	CDKN1A;MT2A;SERPINE1;HMOX1;ADM;SERPINB8
PLXNA3	6/299	6.36224056035047 E-4	CDKN1A;MT2A;SERPINE1;HMOX1;ADM;SERPINB8
PLXND1	6/299	6.36224056035047 E-4	CDKN1A;SERPINE1;PDE2A;HMOX1;ADM;SERPINB8
PPARD	6/299	6.36224056035047 E-4	CDKN1A;MT2A;SERPINE1;HMOX1;ADM;SERPINB8
PRDM7	6/299	6.36224056035047 E-4	CDKN1A;SERPINE1;SLAMF7;ADM;PHACTR1;SERPINB8
PRR3	6/299	6.36224056035047 E-4	CDKN1A;MT2A;HMOX1;SERPINB8;CORO1A;SRM
RBPJ	6/299	6.36224056035047 E-4	CDKN1A;MT2A;SERPINE1;HMOX1;ADM;SERPINB8
RGS9	6/299	6.36224056035047 E-4	CDKN1A;MT2A;ARC;PDE2A;PHACTR1;SERPINB8
RIOK2	6/299	6.36224056035047 E-4	CDKN1A;POLR2A;SERPINE1;HMOX1;SERPINB8;BYSL
RNASE2	6/299	6.36224056035047 E-4	CDKN1A;MT2A;SERPINE1;HMOX1;ADM;CORO1A
RNF125	6/299	6.36224056035047 E-4	CDKN1A;MT2A;SERPINE1;HMOX1;ADM;SERPINB8
RUNX1	6/299	6.36224056035047 E-4	CDKN1A;MT2A;SERPINE1;HMOX1;ADM;SERPINB8
SEBOX	6/299	6.36224056035047 E-4	CDKN1A;ARC;OSGIN1;HMOX1;ATF5;SERPINB8
SEMA4A	6/299	6.36224056035047 E-4	CDKN1A;HMOX1;SLAMF7;ADM;SERPINB8;CORO1A
SF3A2	6/299	6.36224056035047 E-4	SF3B4;NUDC;POLR2A;PRPF19;SF1;SRM
SKIL	6/299	6.36224056035047 E-4	CDKN1A;MT2A;SERPINE1;HMOX1;ADM;SERPINB8
SLC26A10	6/299	6.36224056035047 E-4	NGFR;CDKN1A;ARC;PDE2A;ADM;SERPINB8
SMAD6	6/299	6.36224056035047 E-4	CDKN1A;SPHK1;SERPINE1;HMOX1;ADM;SERPINB8
SNAI2	6/299	6.36224056035047 E-4	CDKN1A;MT2A;SERPINE1;HMOX1;ADM;SERPINB8
SOX4	6/299	6.36224056035047 E-4	CDKN1A;MT2A;SERPINE1;HMOX1;ADM;SERPINB8
SOX9	6/299	6.36224056035047 E-4	CDKN1A;MT2A;SERPINE1;HMOX1;ADM;SERPINB8
SP100	6/299	6.36224056035047 E-4	CDKN1A;MT2A;SERPINE1;HMOX1;SLAMF7;SERPINB8
SRF	6/299	6.36224056035047 E-4	CDKN1A;MT2A;POLR2A;SERPINE1;ADM;SERPINB8

STAT1	6/299	6.36224056035047 E-4	CDKN1A;MT2A;SERPINE1;HMOX1;AD M;CORO1A
STAT2	6/299	6.36224056035047 E-4	CDKN1A;MT2A;SERPINE1;HMOX1;AD M;SERPINB8
STAT5A	6/299	6.36224056035047 E-4	CDKN1A;MT2A;SERPINE1;HMOX1;SE RPINB8;CORO1A
TBX19	6/299	6.36224056035047 E-4	CDKN1A;SERPINE1;HMOX1;SLAMF7; ADM;SERPINB8
TBX3	6/299	6.36224056035047 E-4	CDKN1A;MT2A;SERPINE1;HMOX1;AD M;SERPINB8
TBX6	6/299	6.36224056035047 E-4	CDKN1A;MT2A;HMOX1;ADM;SERPINB 8;CORO1A
TCF15	6/299	6.36224056035047 E-4	NGFR;CDKN1A;SERPINE1;HMOX1;AD M;SERPINB8
TCF23	6/299	6.36224056035047 E-4	CDKN1A;MT2A;ARC;SERPINE1;HMOX 1;SERPINB8
TCF24	6/299	6.36224056035047 E-4	NGFR;CDKN1A;MT2A;ARC;ADM;SERP INB8
TEAD3	6/299	6.36224056035047 E-4	CDKN1A;SPHK1;SERPINE1;HMOX1;A DM;SERPINB8
TFAP2C	6/299	6.36224056035047 E-4	CDKN1A;MT2A;SERPINE1;HMOX1;AD M;SERPINB8
TFAP4	6/299	6.36224056035047 E-4	CDKN1A;MT2A;CCDC86;HMOX1;SERP INB8;SRM
TFCP2L1	6/299	6.36224056035047 E-4	CDKN1A;MT2A;SERPINE1;HMOX1;AD M;SERPINB8
TFE3	6/299	6.36224056035047 E-4	CDKN1A;MT2A;SERPINE1;HMOX1;AD M;SERPINB8
TFEC	6/299	6.36224056035047 E-4	CDKN1A;SERPINE1;HMOX1;SLAMF7; SERPINB8;CORO1A
TGIF1	6/299	6.36224056035047 E-4	CDKN1A;MT2A;SERPINE1;HMOX1;AD M;SERPINB8
THAP11	6/299	6.36224056035047 E-4	CDKN1A;NUDC;CCDC86;PRPF19;ATF 5;SRM
THAP8	6/299	6.36224056035047 E-4	CDKN1A;MT2A;OSGIN1;HMOX1;ADM; SERPINB8
TIPARP	6/299	6.36224056035047 E-4	CDKN1A;MT2A;SERPINE1;HMOX1;AD M;SERPINB8
TOX2	6/299	6.36224056035047 E-4	CDKN1A;MT2A;SERPINE1;HMOX1;AD M;SERPINB8
TRMT1	6/299	6.36224056035047 E-4	CDKN1A;MT2A;CCDC86;HMOX1;SRM; BYSL
U2AF1L4	6/299	6.36224056035047 E-4	CDKN1A;MT2A;ADM;SERPINB8;CORO 1A;SRM
UNKL	6/299	6.36224056035047 E-4	CDKN1A;POLR2A;OSGIN1;HMOX1;AD M;SERPINB8
ZBTB16	6/299	6.36224056035047 E-4	CDKN1A;MT2A;SERPINE1;HMOX1;AD M;SERPINB8
ZBTB46	6/299	6.36224056035047 E-4	CDKN1A;SPHK1;SERPINE1;PDE2A;A DM;SERPINB8
ZBTB48	6/299	6.36224056035047 E-4	CDKN1A;NUDC;ADM;SERPINB8;CORO 1A;SRM
ZC3H10	6/299	6.36224056035047 E-4	CDKN1A;MT2A;POLR2A;HMOX1;ADM; SERPINB8
ZDHHC11	6/299	6.36224056035047 E-4	CDKN1A;SERPINE1;HMOX1;SLAMF7; ADM;SERPINB8
ZNF114	6/299	6.36224056035047 E-4	CDKN1A;SPHK1;SERPINE1;HMOX1;A DM;SERPINB8
ZNF142	6/299	6.36224056035047	NXF1;POLR2A;PRCC;CCDC86;SERPI

		E-4	NB8;SRM
ZNF165	6/299	6.36224056035047 E-4	CDKN1A;MT2A;SERPINE1;HMOX1;ADM;SERPINB8
ZNF177	6/299	6.36224056035047 E-4	CDKN1A;SERPINE1;NAP1L3;HMOX1;ADM;SERPINB8
ZNF263	6/299	6.36224056035047 E-4	CDKN1A;POLR2A;CCDC86;HMOX1;ADM;ATF5
ZNF358	6/299	6.36224056035047 E-4	CDKN1A;MT2A;HMOX1;ADM;SERPINB8;SRM
ZNF385C	6/299	6.36224056035047 E-4	NGFR;CDKN1A;ARC;SERPINE1;SERPINB8;PLEKHA7
ZNF395	6/299	6.36224056035047 E-4	CDKN1A;MT2A;SERPINE1;HMOX1;ADM;SERPINB8
ZNF404	6/299	6.36224056035047 E-4	CDKN1A;MT2A;SERPINE1;HMOX1;ADM;SERPINB8
ZNF416	6/299	6.36224056035047 E-4	CDKN1A;ARC;SERPINE1;HMOX1;ADM;SERPINB8
ZNF432	6/299	6.36224056035047 E-4	CDKN1A;MT2A;SERPINE1;HMOX1;ADM;SERPINB8
ZNF488	6/299	6.36224056035047 E-4	NGFR;CDKN1A;SERPINE1;HMOX1;ADM;SERPINB8
ZNF503	6/299	6.36224056035047 E-4	CDKN1A;MT2A;SERPINE1;HMOX1;ADM;SERPINB8
ZNF541	6/299	6.36224056035047 E-4	NGFR;CDKN1A;ARC;KCNC3;SERPINE1;SERPINB8
ZNF569	6/299	6.36224056035047 E-4	CDKN1A;SERPINE1;NAP1L3;HMOX1;ADM;SERPINB8
ZNF580	6/299	6.36224056035047 E-4	CDKN1A;MT2A;HMOX1;ADM;SERPINB8;CORO1A
ZNF581	6/299	6.36224056035047 E-4	CDKN1A;MT2A;HMOX1;ADM;SERPINB8;SRM
ZNF600	6/299	6.36224056035047 E-4	CDKN1A;MT2A;HMOX1;SLAMF7;ADM;SERPINB8
ZNF692	6/299	6.36224056035047 E-4	NXF1;CDKN1A;MT2A;ADM;SERPINB8;SRM
ZNF696	6/299	6.36224056035047 E-4	CDKN1A;MT2A;SPHK1;CCDC86;ADM;BYSL
ZNFX1	6/299	6.36224056035047 E-4	CDKN1A;MT2A;SERPINE1;HMOX1;ADM;SERPINB8
AKAP8	5/299	0.0033964572273667195	NXF1;CDKN1A;POLR2A;CCDC86;SF1
AR	5/299	0.0033964572273667195	CDKN1A;SERPINE1;HMOX1;ADM;SERPINB8
ARID3A	5/299	0.0033964572273667195	CDKN1A;SERPINE1;HMOX1;ADM;SERPINB8
BACH1	5/299	0.0033964572273667195	CDKN1A;SERPINE1;HMOX1;ADM;SERPINB8
BCL11B	5/299	0.0033964572273667195	CDKN1A;ADM;PHACTR1;SERPINB8;CORO1A
BOLA1	5/299	0.0033964572273667195	CDKN1A;MT2A;HMOX1;SERPINB8;SRM
CARHSP1	5/299	0.0033964572273667195	CDKN1A;MT2A;HMOX1;ADM;SERPINB8
CENPB	5/299	0.0033964572273667195	CDKN1A;MT2A;NUDC;ATF5;SRM
CREB3L3	5/299	0.0033964572273667195	CDKN1A;SERPINE1;OSGIN1;HMOX1;SERPINB8
CTCFL	5/299	0.0033964572273667195	CDKN1A;MT2A;SERPINE1;PDE2A;SERPINB8

DLX4	5/299	0.0033964572273667195	NGFR;CDKN1A;SPHK1;ADM;SERPINB8
DMAP1	5/299	0.0033964572273667195	CDKN1A;NUDC;POLR2A;PRPF19;SRM
DMTF1	5/299	0.0033964572273667195	CDKN1A;SERPINE1;HMOX1;ADM;SERPINB8
DNAJC2	5/299	0.0033964572273667195	NUDC;GAR1;CCDC86;SRM;BYSL
DPF2	5/299	0.0033964572273667195	NXF1;CDKN1A;NUDC;POLR2A;SF1
DRAP1	5/299	0.0033964572273667195	CDKN1A;MT2A;NUDC;PSMD2;SRM
DSP	5/299	0.0033964572273667195	CDKN1A;MT2A;SERPINE1;ADM;SERPINB8
E2F6	5/299	0.0033964572273667195	CDKN1A;HMOX1;ADM;SERPINB8;SRM
E4F1	5/299	0.0033964572273667195	NXF1;CDKN1A;MT2A;POLR2A;SERPINB8
EBF4	5/299	0.0033964572273667195	NGFR;CDKN1A;SERPINE1;ADM;SERPINB8
EHF	5/299	0.0033964572273667195	CDKN1A;MT2A;SERPINE1;ADM;SERPINB8
EN2	5/299	0.0033964572273667195	NGFR;CDKN1A;ARC;ADM;SERPINB8
ESR2	5/299	0.0033964572273667195	CDKN1A;SERPINE1;HMOX1;ADM;SERPINB8
ETV2	5/299	0.0033964572273667195	CDKN1A;SERPINE1;ADM;SERPINB8;CORO1A
FMNL2	5/299	0.0033964572273667195	CDKN1A;SERPINE1;HMOX1;ADM;SERPINB8
FOXA3	5/299	0.0033964572273667195	CDKN1A;SERPINE1;HMOX1;ADM;SERPINB8
FOXC1	5/299	0.0033964572273667195	CDKN1A;MT2A;SERPINE1;ADM;SERPINB8
FOXF1	5/299	0.0033964572273667195	CDKN1A;SERPINE1;ADM;S1PR3;SERPINB8
FOXL1	5/299	0.0033964572273667195	CDKN1A;MT2A;SERPINE1;ADM;SERPINB8
GATA6	5/299	0.0033964572273667195	CDKN1A;SERPINE1;HMOX1;ADM;SERPINB8
GATAD2A	5/299	0.0033964572273667195	CDKN1A;POLR2A;CCDC86;SF1;SRM
GCM1	5/299	0.0033964572273667195	CDKN1A;SERPINE1;HMOX1;ADM;SERPINB8
GRHL3	5/299	0.0033964572273667195	CDKN1A;SERPINE1;HMOX1;ADM;SERPINB8
GTF2F1	5/299	0.0033964572273667195	CDKN1A;NUDC;POLR2A;CCDC86;SRM
H1F0	5/299	0.0033964572273667195	CDKN1A;MT2A;HMOX1;ADM;SERPINB8
HES3	5/299	0.0033964572273667195	CDKN1A;ARC;KCNC3;PDE2A;ADM
HES6	5/299	0.0033964572273667195	CDKN1A;MT2A;ADM;SERPINB8;CORO1A
HESX1	5/299	0.0033964572273667195	CDKN1A;MT2A;SERPINE1;HMOX1;ADM
HEY2	5/299	0.0033964572273667195	CDKN1A;SERPINE1;ADM;S1PR3;SERPINB8
HIF1A	5/299	0.00339645722736	CDKN1A;MT2A;SERPINE1;HMOX1;AD

		67195	M
HIST1H1C	5/299	0.0033964572273667195	CDKN1A;MT2A;SERPINE1;HMOX1;ADM
HIVEP2	5/299	0.0033964572273667195	CDKN1A;MT2A;SERPINE1;ADM;SERPINB8
HMGA1	5/299	0.0033964572273667195	CDKN1A;MT2A;SERPINE1;HMOX1;SRM
HOXA1	5/299	0.0033964572273667195	CDKN1A;SERPINE1;HMOX1;ADM;SERPINB8
HOXA10	5/299	0.0033964572273667195	CDKN1A;SERPINE1;HMOX1;ADM;SERPINB8
HOXA5	5/299	0.0033964572273667195	CDKN1A;SERPINE1;HMOX1;ADM;SERPINB8
HOXB2	5/299	0.0033964572273667195	CDKN1A;MT2A;SERPINE1;ADM;SERPINB8
HOXB8	5/299	0.0033964572273667195	NGFR;CDKN1A;SERPINE1;ADM;SERPINB8
HOXC8	5/299	0.0033964572273667195	CDKN1A;SERPINE1;HMOX1;ADM;SERPINB8
ID4	5/299	0.0033964572273667195	CDKN1A;MT2A;SERPINE1;ADM;SERPINB8
INF2	5/299	0.0033964572273667195	CDKN1A;MT2A;SERPINE1;HMOX1;SERPINB8
IRF3	5/299	0.0033964572273667195	CDKN1A;MT2A;HMOX1;CORO1A;SRM
IRF5	5/299	0.0033964572273667195	CDKN1A;SERPINE1;HMOX1;SLAMF7;CORO1A
IRF6	5/299	0.0033964572273667195	CDKN1A;SERPINE1;HMOX1;ADM;SERPINB8
IRX3	5/299	0.0033964572273667195	CDKN1A;MT2A;SERPINE1;ADM;SERPINB8
JUND	5/299	0.0033964572273667195	CDKN1A;MT2A;SERPINE1;HMOX1;ADM
KLF11	5/299	0.0033964572273667195	CDKN1A;SERPINE1;HMOX1;ADM;SERPINB8
KLF16	5/299	0.0033964572273667195	CDKN1A;MT2A;CCDC86;HMOX1;SRM
KLF7	5/299	0.0033964572273667195	CDKN1A;SERPINE1;HMOX1;ADM;SERPINB8
LEF1	5/299	0.0033964572273667195	CDKN1A;MT2A;SERPINE1;ADM;SERPINB8
MAF	5/299	0.0033964572273667195	CDKN1A;SERPINE1;HMOX1;ADM;SERPINB8
MBD1	5/299	0.0033964572273667195	CDKN1A;POLR2A;SERPINE1;HMOX1;SERPINB8
MESP1	5/299	0.0033964572273667195	CDKN1A;MT2A;SPHK1;ADM;SERPINB8
MITF	5/299	0.0033964572273667195	CDKN1A;SERPINE1;HMOX1;ADM;SERPINB8
MSX2	5/299	0.0033964572273667195	NGFR;CDKN1A;SERPINE1;ADM;SERPINB8
MTA2	5/299	0.0033964572273667195	CDKN1A;POLR2A;PSMD2;PRPF19;SF1
MXI1	5/299	0.0033964572273667195	CDKN1A;MT2A;SERPINE1;HMOX1;ADM
NFATC1	5/299	0.0033964572273667195	CDKN1A;SERPINE1;HMOX1;ADM;SERPINB8
NFATC4	5/299	0.0033964572273667195	CDKN1A;SERPINE1;HMOX1;ADM;SERPINB8

NFE2L2	5/299	0.0033964572273667195	CDKN1A;MT2A;SERPINE1;HMOX1;ADM
NOC3L	5/299	0.0033964572273667195	GAR1;CCDC86;SERPINB8;SRM;BYSL
NR1D2	5/299	0.0033964572273667195	CDKN1A;MT2A;HMOX1;ADM;SERPINB8
NR2F1	5/299	0.0033964572273667195	CDKN1A;SERPINE1;ADM;S1PR3;SERPINB8
NR3C1	5/299	0.0033964572273667195	CDKN1A;SERPINE1;HMOX1;ADM;SERPINB8
OLIG2	5/299	0.0033964572273667195	NGFR;CDKN1A;ARC;ADM;SERPINB8
OSR2	5/299	0.0033964572273667195	CDKN1A;MT2A;SERPINE1;ADM;SERPINB8
OVOL1	5/299	0.0033964572273667195	CDKN1A;SERPINE1;HMOX1;ADM;SERPINB8
PAWR	5/299	0.0033964572273667195	CDKN1A;SERPINE1;HMOX1;ADM;SERPINB8
PAX8	5/299	0.0033964572273667195	CDKN1A;SERPINE1;HMOX1;ADM;SERPINB8
PITX1	5/299	0.0033964572273667195	CDKN1A;MT2A;SERPINE1;ADM;SERPINB8
PITX3	5/299	0.0033964572273667195	NGFR;CDKN1A;SPHK1;SERPINE1;SERPINB8
PLAGL1	5/299	0.0033964572273667195	CDKN1A;MT2A;SERPINE1;ADM;SERPINB8
PLEK	5/299	0.0033964572273667195	CDKN1A;SERPINE1;HMOX1;SLAMF7;CORO1A
PPARG	5/299	0.0033964572273667195	CDKN1A;SERPINE1;HMOX1;ADM;SERPINB8
PREB	5/299	0.0033964572273667195	CDKN1A;PSMD2;PRPF19;SERPINB8;SRM
PRMT3	5/299	0.0033964572273667195	GAR1;CCDC86;SERPINB8;SRM;BYSL
PROX2	5/299	0.0033964572273667195	NGFR;CDKN1A;SERPINE1;PHACTR1;SERPINB8
RARA	5/299	0.0033964572273667195	CDKN1A;SERPINE1;HMOX1;ADM;SERPINB8
RARG	5/299	0.0033964572273667195	CDKN1A;SERPINE1;HMOX1;ADM;SERPINB8
RBM10	5/299	0.0033964572273667195	NXF1;NUDC;POLR2A;PRPF19;SF1
RBM22	5/299	0.0033964572273667195	SF3B4;NXF1;POLR2A;PRPF19;SF1
RCOR2	5/299	0.0033964572273667195	NGFR;CDKN1A;MT2A;ADM;SERPINB8
REL	5/299	0.0033964572273667195	CDKN1A;SERPINE1;HMOX1;ADM;SERPINB8
RNF166	5/299	0.0033964572273667195	CDKN1A;MT2A;HMOX1;CORO1A;SRM
RORC	5/299	0.0033964572273667195	CDKN1A;SERPINE1;HMOX1;ADM;SERPINB8
RUNX2	5/299	0.0033964572273667195	CDKN1A;MT2A;SERPINE1;HMOX1;SERPINB8
SF3A3	5/299	0.0033964572273667195	SF3B4;NUDC;PSMD2;PRPF19;SRM
SIX1	5/299	0.0033964572273667195	CDKN1A;MT2A;SERPINE1;ADM;SERPINB8
SIX2	5/299	0.00339645722736	NGFR;CDKN1A;SERPINE1;ADM;SERP

		67195	INB8
SIX4	5/299	0.0033964572273667195	CDKN1A;MT2A;SERPINE1;ADM;SERPINB8
SLC2A4RG	5/299	0.0033964572273667195	CDKN1A;MT2A;CCDC86;SERPINB8;SRM
SMAD1	5/299	0.0033964572273667195	CDKN1A;SERPINE1;HMOX1;ADM;SERPINB8
SMAD9	5/299	0.0033964572273667195	CDKN1A;SERPINE1;HMOX1;ADM;SERPINB8
SOX10	5/299	0.0033964572273667195	NGFR;CDKN1A;MT2A;ADM;SERPINB8
SOX15	5/299	0.0033964572273667195	CDKN1A;MT2A;SERPINE1;ADM;SERPINB8
SOX17	5/299	0.0033964572273667195	CDKN1A;SERPINE1;PDE2A;ADM;SERPINB8
SOX7	5/299	0.0033964572273667195	CDKN1A;SERPINE1;HMOX1;ADM;SERPINB8
SPIC	5/299	0.0033964572273667195	CDKN1A;SERPINE1;HMOX1;SLAMF7;SERPINB8
SREBF1	5/299	0.0033964572273667195	CDKN1A;MT2A;HMOX1;ADM;SERPINB8
SSH3	5/299	0.0033964572273667195	CDKN1A;MT2A;HMOX1;ADM;SERPINB8
STAT6	5/299	0.0033964572273667195	CDKN1A;SERPINE1;HMOX1;ADM;SERPINB8
TFAP2A	5/299	0.0033964572273667195	CDKN1A;SERPINE1;HMOX1;ADM;SERPINB8
TFAP2E	5/299	0.0033964572273667195	NGFR;CDKN1A;ARC;ADM;SERPINB8
THAP10	5/299	0.0033964572273667195	MT2A;SERPINE1;HMOX1;ADM;SERPINB8
TIGD3	5/299	0.0033964572273667195	CDKN1A;ARC;HMOX1;ADM;PHACTR1
TRAFD1	5/299	0.0033964572273667195	CDKN1A;MT2A;HMOX1;ADM;SERPINB8
TWIST1	5/299	0.0033964572273667195	CDKN1A;MT2A;SERPINE1;ADM;SERPINB8
VAX2	5/299	0.0033964572273667195	NGFR;CDKN1A;SERPINE1;ADM;SERPINB8
VPS72	5/299	0.0033964572273667195	SF3B4;CDKN1A;PSMD2;CCDC86;HMOX1
WT1	5/299	0.0033964572273667195	CDKN1A;SERPINE1;HMOX1;ADM;SERPINB8
XBP1	5/299	0.0033964572273667195	CDKN1A;MT2A;SERPINE1;HMOX1;SERPINB8
YOD1	5/299	0.0033964572273667195	CDKN1A;MT2A;HMOX1;ADM;SERPINB8
ZBED1	5/299	0.0033964572273667195	CDKN1A;MT2A;SERPINE1;ADM;SERPINB8
ZBED2	5/299	0.0033964572273667195	CDKN1A;MT2A;SERPINE1;HMOX1;ADM
ZBTB2	5/299	0.0033964572273667195	CDKN1A;CCDC86;HMOX1;ADM;SERPINB8
ZBTB22	5/299	0.0033964572273667195	CDKN1A;MT2A;HMOX1;ATF5;SERPINB8
ZBTB32	5/299	0.0033964572273667195	CDKN1A;MT2A;SERPINE1;HMOX1;SLAMF7
ZBTB7B	5/299	0.0033964572273667195	CDKN1A;MT2A;HMOX1;ADM;SERPINB8

ZC3HAV1	5/299	0.0033964572273667195	CDKN1A;MT2A;HMOX1;ADM;SERPINB8
ZFHX2	5/299	0.0033964572273667195	CDKN1A;ARC;KCNC3;PDE2A;SERPINB8
ZFP36	5/299	0.0033964572273667195	CDKN1A;MT2A;SERPINE1;HMOX1;ADM
ZFP36L1	5/299	0.0033964572273667195	CDKN1A;MT2A;SERPINE1;HMOX1;ADM
ZFP57	5/299	0.0033964572273667195	CDKN1A;MT2A;SERPINE1;HMOX1;SERPINB8
ZFR2	5/299	0.0033964572273667195	CDKN1A;ARC;PDE2A;PHACTR1;SERPINB8
ZFY	5/299	0.0033964572273667195	CDKN1A;MT2A;SERPINE1;ADM;SERPINB8
ZIC2	5/299	0.0033964572273667195	CDKN1A;MT2A;SERPINE1;ADM;SERPINB8
ZKSCAN4	5/299	0.0033964572273667195	CDKN1A;MT2A;HMOX1;ADM;SERPINB8
ZMAT3	5/299	0.0033964572273667195	CDKN1A;MT2A;SERPINE1;HMOX1;SERPINB8
ZNF101	5/299	0.0033964572273667195	MT2A;HMOX1;ADM;SERPINB8;CORO1A
ZNF14	5/299	0.0033964572273667195	CDKN1A;MT2A;HMOX1;ADM;SERPINB8
ZNF175	5/299	0.0033964572273667195	CDKN1A;SERPINE1;HMOX1;ADM;SERPINB8
ZNF18	5/299	0.0033964572273667195	CDKN1A;SERPINE1;HMOX1;ADM;SERPINB8
ZNF212	5/299	0.0033964572273667195	CDKN1A;HMOX1;ADM;SERPINB8;CORO1A
ZNF217	5/299	0.0033964572273667195	CDKN1A;MT2A;SERPINE1;ADM;SERPINB8
ZNF226	5/299	0.0033964572273667195	CDKN1A;PDE2A;HMOX1;ADM;SERPINB8
ZNF256	5/299	0.0033964572273667195	CDKN1A;MT2A;HMOX1;ADM;SERPINB8
ZNF267	5/299	0.0033964572273667195	CDKN1A;MT2A;HMOX1;ADM;SERPINB8
ZNF275	5/299	0.0033964572273667195	CDKN1A;SERPINE1;HMOX1;ADM;SERPINB8
ZNF281	5/299	0.0033964572273667195	CDKN1A;SERPINE1;HMOX1;ADM;SERPINB8
ZNF316	5/299	0.0033964572273667195	CDKN1A;POLR2A;OSGIN1;HMOX1;SERPINB8
ZNF317	5/299	0.0033964572273667195	CDKN1A;POLR2A;HMOX1;ADM;SERPINB8
ZNF329	5/299	0.0033964572273667195	CDKN1A;MT2A;HMOX1;ADM;SERPINB8
ZNF335	5/299	0.0033964572273667195	NXF1;CDKN1A;POLR2A;CCDC86;SERPINB8
ZNF35	5/299	0.0033964572273667195	CDKN1A;MT2A;HMOX1;ADM;SERPINB8
ZNF354A	5/299	0.0033964572273667195	CDKN1A;SERPINE1;HMOX1;ADM;SERPINB8
ZNF385A	5/299	0.0033964572273667195	CDKN1A;SERPINE1;HMOX1;ADM;SERPINB8
ZNF394	5/299	0.0033964572273667195	CDKN1A;MT2A;HMOX1;ADM;SERPINB8
ZNF425	5/299	0.00339645722736	CDKN1A;SERPINE1;HMOX1;ADM;SER

		67195	PINB8
ZNF428	5/299	0.0033964572273667195	CDKN1A;MT2A;NUDC;SERPINB8;SRM
ZNF438	5/299	0.0033964572273667195	CDKN1A;MT2A;HMOX1;ADM;SERPINB8
ZNF439	5/299	0.0033964572273667195	CDKN1A;MT2A;SERPINE1;ADM;SERPINB8
ZNF467	5/299	0.0033964572273667195	CDKN1A;MT2A;HMOX1;ADM;SERPINB8
ZNF468	5/299	0.0033964572273667195	CDKN1A;SERPINE1;HMOX1;ADM;SERPINB8
ZNF474	5/299	0.0033964572273667195	CDKN1A;SERPINE1;HMOX1;ADM;SERPINB8
ZNF485	5/299	0.0033964572273667195	CDKN1A;CCDC86;HMOX1;ADM;SERPINB8
ZNF547	5/299	0.0033964572273667195	CDKN1A;MT2A;SERPINE1;HMOX1;SERPINB8
ZNF558	5/299	0.0033964572273667195	CDKN1A;SERPINE1;HMOX1;ADM;SERPINB8
ZNF57	5/299	0.0033964572273667195	CDKN1A;MT2A;HMOX1;ADM;SERPINB8
ZNF584	5/299	0.0033964572273667195	CDKN1A;SPHK1;CCDC86;ADM;SERPINB8
ZNF597	5/299	0.0033964572273667195	CDKN1A;SERPINE1;HMOX1;ADM;SERPINB8
ZNF616	5/299	0.0033964572273667195	CDKN1A;MT2A;NAP1L3;ADM;SERPINB8
ZNF641	5/299	0.0033964572273667195	CDKN1A;SERPINE1;HMOX1;ADM;SERPINB8
ZNF668	5/299	0.0033964572273667195	CDKN1A;POLR2A;CCDC86;ADM;CORO1A
ZNF672	5/299	0.0033964572273667195	CDKN1A;MT2A;ADM;ATF5;SERPINB8
ZNF7	5/299	0.0033964572273667195	CDKN1A;ARC;HMOX1;ADM;SERPINB8
ZNF703	5/299	0.0033964572273667195	CDKN1A;SERPINE1;HMOX1;ADM;SERPINB8
ZNF75D	5/299	0.0033964572273667195	CDKN1A;MT2A;HMOX1;ADM;SERPINB8
ZNF761	5/299	0.0033964572273667195	CDKN1A;SERPINE1;HMOX1;ADM;SERPINB8
ZNF77	5/299	0.0033964572273667195	CDKN1A;MT2A;HMOX1;ADM;SERPINB8
ZNF79	5/299	0.0033964572273667195	CDKN1A;SERPINE1;HMOX1;ADM;SERPINB8
ZNF799	5/299	0.0033964572273667195	CDKN1A;SERPINE1;HMOX1;ADM;SERPINB8
ZNF821	5/299	0.0033964572273667195	CDKN1A;HMOX1;ADM;SERPINB8;CORO1A
ZNF823	5/299	0.0033964572273667195	CDKN1A;MT2A;HMOX1;ADM;SERPINB8
ZNF841	5/299	0.0033964572273667195	CDKN1A;SERPINE1;HMOX1;ADM;SERPINB8
ZRSR2	5/299	0.0033964572273667195	CDKN1A;MT2A;NUDC;HMOX1;SERPINB8
ZSCAN18	5/299	0.0033964572273667195	CDKN1A;MT2A;NAP1L3;HMOX1;SERPINB8
AKAP8L	4/299	0.015457367105658653	NXF1;CDKN1A;HMOX1;CORO1A

AKNA	4/299	0.015457367105658653	CDKN1A;HMOX1;SERPINB8;CORO1A
ANKZF1	4/299	0.015457367105658653	CDKN1A;HMOX1;ADM;SERPINB8
ARNT2	4/299	0.015457367105658653	CDKN1A;SERPINE1;ADM;SERPINB8
ATF4	4/299	0.015457367105658653	CDKN1A;MT2A;HMOX1;ADM
ATF6	4/299	0.015457367105658653	CDKN1A;MT2A;HMOX1;SERPINB8
ATMIN	4/299	0.015457367105658653	CDKN1A;SERPINE1;HMOX1;SERPINB8
BCL11A	4/299	0.015457367105658653	CDKN1A;ADM;PHACTR1;SERPINB8
BNC1	4/299	0.015457367105658653	CDKN1A;SERPINE1;ADM;SERPINB8
BRPF1	4/299	0.015457367105658653	NXF1;CDKN1A;POLR2A;SF1
CAMTA2	4/299	0.015457367105658653	CDKN1A;POLR2A;PDE2A;SERPINB8
CBLL1	4/299	0.015457367105658653	CDKN1A;CCDC86;SERPINB8;SF1
CPSF4L	4/299	0.015457367105658653	NGFR;CDKN1A;OSGIN1;SERPINB8
CREB3L4	4/299	0.015457367105658653	CDKN1A;MT2A;ADM;SERPINB8
CREBL2	4/299	0.015457367105658653	CDKN1A;MT2A;HMOX1;SERPINB8
CUX2	4/299	0.015457367105658653	CDKN1A;PDE2A;PHACTR1;SERPINB8
DEAF1	4/299	0.015457367105658653	CDKN1A;ADM;SERPINB8;SRM
DLX5	4/299	0.015457367105658653	NGFR;CDKN1A;ADM;SERPINB8
DMRTA1	4/299	0.015457367105658653	CDKN1A;SERPINE1;ADM;SERPINB8
DNAJC1	4/299	0.015457367105658653	CDKN1A;MT2A;HMOX1;SERPINB8
DVL2	4/299	0.015457367105658653	CDKN1A;POLR2A;HMOX1;SERPINB8
DVL3	4/299	0.015457367105658653	CDKN1A;POLR2A;PSMD2;SF1
E2F2	4/299	0.015457367105658653	CDKN1A;MT2A;HMOX1;CORO1A
E2F7	4/299	0.015457367105658653	CDKN1A;SERPINE1;ADM;SERPINB8
EBF1	4/299	0.015457367105658653	PDE2A;ADM;S1PR3;SERPINB8
ERG	4/299	0.015457367105658653	CDKN1A;SERPINE1;PDE2A;SERPINB8
ESRRA	4/299	0.015457367105658653	CDKN1A;MT2A;CCDC86;PRPF19
ETV1	4/299	0.015457367105658653	CDKN1A;SERPINE1;HMOX1;SERPINB8
ETV3	4/299	0.015457367105658653	CDKN1A;SERPINE1;HMOX1;ADM
FIZ1	4/299	0.015457367105658653	CDKN1A;HMOX1;ATF5;SRM
FLI1	4/299	0.01545736710565	CDKN1A;SERPINE1;SERPINB8;CORO

		8653	1A
FOXA1	4/299	0.015457367105658653	CDKN1A;SERPINE1;ADM;SERPINB8
FOXA2	4/299	0.015457367105658653	CDKN1A;SERPINE1;ADM;SERPINB8
FOXD2	4/299	0.015457367105658653	NGFR;CDKN1A;ADM;SERPINB8
FOXD4	4/299	0.015457367105658653	CDKN1A;SERPINE1;ADM;SERPINB8
FOXF2	4/299	0.015457367105658653	CDKN1A;SERPINE1;ADM;SERPINB8
FOXH1	4/299	0.015457367105658653	CDKN1A;ARC;KCNC3;ADM
FOXN1	4/299	0.015457367105658653	CDKN1A;ARC;SERPINE1;SERPINB8
FOXP3	4/299	0.015457367105658653	CDKN1A;SERPINE1;HMOX1;SLAMF7
FOXR2	4/299	0.015457367105658653	CDKN1A;MT2A;ARC;SERPINB8
GABPB1	4/299	0.015457367105658653	CDKN1A;SERPINE1;HMOX1;SERPINB8
GATA2	4/299	0.015457367105658653	CDKN1A;SERPINE1;HMOX1;ADM
GFI1	4/299	0.015457367105658653	CDKN1A;SLAMF7;SERPINB8;CORO1A
GLIS1	4/299	0.015457367105658653	CDKN1A;SERPINE1;ADM;SERPINB8
GLIS3	4/299	0.015457367105658653	CDKN1A;SERPINE1;ADM;SERPINB8
GTF2E2	4/299	0.015457367105658653	CDKN1A;CCDC86;HMOX1;ADM
GTF2F2	4/299	0.015457367105658653	CDKN1A;NUDC;CCDC86;SRM
GTF2IRD2	4/299	0.015457367105658653	CDKN1A;MT2A;HMOX1;SERPINB8
GTF2IRD2B	4/299	0.015457367105658653	CDKN1A;SERPINE1;HMOX1;SERPINB8
GZF1	4/299	0.015457367105658653	CDKN1A;HMOX1;ADM;SERPINB8
H1FX	4/299	0.015457367105658653	CDKN1A;MT2A;CORO1A;SRM
HAND1	4/299	0.015457367105658653	NGFR;CDKN1A;ADM;SERPINB8
HBP1	4/299	0.015457367105658653	CDKN1A;MT2A;HMOX1;SERPINB8
HES5	4/299	0.015457367105658653	CDKN1A;ARC;ADM;SERPINB8
HIC2	4/299	0.015457367105658653	CDKN1A;POLR2A;HMOX1;SERPINB8
HIST1H1A	4/299	0.015457367105658653	CDKN1A;MT2A;HMOX1;SERPINB8
HMG20B	4/299	0.015457367105658653	CDKN1A;MT2A;HMOX1;CORO1A
HOMEZ	4/299	0.015457367105658653	CDKN1A;HMOX1;ADM;SERPINB8
HOXA11	4/299	0.015457367105658653	CDKN1A;SERPINE1;ADM;SERPINB8
HOXA4	4/299	0.015457367105658653	NGFR;CDKN1A;ADM;SERPINB8

HOXA9	4/299	0.015457367105658653	CDKN1A;SERPINE1;ADM;SERPINB8
HOXB4	4/299	0.015457367105658653	NGFR;CDKN1A;SERPINE1;SERPINB8
HOXB5	4/299	0.015457367105658653	CDKN1A;SERPINE1;ADM;SERPINB8
HOXC9	4/299	0.015457367105658653	CDKN1A;SERPINE1;ADM;SERPINB8
HOXD1	4/299	0.015457367105658653	CDKN1A;SERPINE1;ADM;SERPINB8
HOXD10	4/299	0.015457367105658653	CDKN1A;SERPINE1;ADM;SERPINB8
HOXD11	4/299	0.015457367105658653	CDKN1A;SERPINE1;ADM;SERPINB8
HOXD4	4/299	0.015457367105658653	CDKN1A;SERPINE1;ADM;SERPINB8
HOXD8	4/299	0.015457367105658653	CDKN1A;SERPINE1;ADM;SERPINB8
HSF4	4/299	0.015457367105658653	CDKN1A;HMOX1;ADM;SERPINB8
IRF8	4/299	0.015457367105658653	CDKN1A;HMOX1;SLAMF7;CORO1A
IRX2	4/299	0.015457367105658653	CDKN1A;MT2A;ADM;SERPINB8
IRX5	4/299	0.015457367105658653	CDKN1A;MT2A;ADM;SERPINB8
ISL2	4/299	0.015457367105658653	NGFR;CDKN1A;ADM;SERPINB8
JAZF1	4/299	0.015457367105658653	CDKN1A;HMOX1;PHACTR1;SERPINB8
KAT5	4/299	0.015457367105658653	CDKN1A;POLR2A;PRPF19;SF1
KLF1	4/299	0.015457367105658653	CDKN1A;MT2A;HMOX1;CORO1A
KLF8	4/299	0.015457367105658653	CDKN1A;MT2A;SERPINE1;SERPINB8
LHX1	4/299	0.015457367105658653	NGFR;CDKN1A;ADM;SERPINB8
LHX2	4/299	0.015457367105658653	CDKN1A;MT2A;ADM;SERPINB8
LYL1	4/299	0.015457367105658653	CDKN1A;MT2A;HMOX1;CORO1A
MAZ	4/299	0.015457367105658653	CDKN1A;POLR2A;SF1;SRM
MBD3	4/299	0.015457367105658653	CDKN1A;NUDC;PRPF19;SRM
MEIS3	4/299	0.015457367105658653	CDKN1A;SERPINE1;PDE2A;SERPINB8
MEOX1	4/299	0.015457367105658653	CDKN1A;SERPINE1;ADM;SERPINB8
MIXL1	4/299	0.015457367105658653	CDKN1A;SERPINE1;ADM;SERPINB8
MKX	4/299	0.015457367105658653	CDKN1A;SERPINE1;ADM;SERPINB8
MLX	4/299	0.015457367105658653	CDKN1A;CCDC86;HMOX1;SERPINB8
MST1R	4/299	0.015457367105658653	CDKN1A;SERPINE1;ADM;SERPINB8
MTA1	4/299	0.01545736710565	CDKN1A;POLR2A;CCDC86;SF1

		8653	
MXD4	4/299	0.015457367105658653	CDKN1A;MT2A;ADM;SERPINB8
MYBL1	4/299	0.015457367105658653	CDKN1A;SERPINE1;ADM;SERPINB8
MYBL2	4/299	0.015457367105658653	CDKN1A;SERPINE1;HMOX1;SRM
MYOD1	4/299	0.015457367105658653	NGFR;CDKN1A;SERPINE1;SERPINB8
NCOA3	4/299	0.015457367105658653	CDKN1A;MT2A;HMOX1;SERPINB8
NEUROD4	4/299	0.015457367105658653	NGFR;CDKN1A;SERPINE1;SERPINB8
NFATC2	4/299	0.015457367105658653	CDKN1A;SERPINE1;HMOX1;SERPINB8
NFE2L1	4/299	0.015457367105658653	CDKN1A;MT2A;SERPINE1;SERPINB8
NFXL1	4/299	0.015457367105658653	CDKN1A;HMOX1;ADM;SERPINB8
NHLH1	4/299	0.015457367105658653	NGFR;CDKN1A;ARC;SERPINB8
NR1H2	4/299	0.015457367105658653	CDKN1A;MT2A;HMOX1;ATF5
NR1H4	4/299	0.015457367105658653	CDKN1A;SERPINE1;HMOX1;SERPINB8
NR1I3	4/299	0.015457367105658653	CDKN1A;HMOX1;ATF5;SERPINB8
NR5A1	4/299	0.015457367105658653	CDKN1A;POLR2A;HMOX1;SRM
OVOL2	4/299	0.015457367105658653	CDKN1A;MT2A;ADM;SERPINB8
PBX3	4/299	0.015457367105658653	CDKN1A;MT2A;ADM;SERPINB8
PHOX2A	4/299	0.015457367105658653	NGFR;CDKN1A;ARC;ADM
PITX2	4/299	0.015457367105658653	CDKN1A;SERPINE1;ADM;SERPINB8
PKNOX2	4/299	0.015457367105658653	NGFR;PDE2A;PHACTR1;SERPINB8
PLAG1	4/299	0.015457367105658653	CDKN1A;SERPINE1;ADM;SERPINB8
PLAGL2	4/299	0.015457367105658653	CDKN1A;CCDC86;HMOX1;SERPINB8
PLXNA1	4/299	0.015457367105658653	CDKN1A;SERPINE1;HMOX1;SERPINB8
PLXNA2	4/299	0.015457367105658653	CDKN1A;SERPINE1;PDE2A;SERPINB8
PLXNA4	4/299	0.015457367105658653	CDKN1A;PDE2A;PHACTR1;SERPINB8
PLXNC1	4/299	0.015457367105658653	CDKN1A;ADM;SERPINB8;CORO1A
POLE4	4/299	0.015457367105658653	CDKN1A;MT2A;SERPINB8;SRM
POU2F3	4/299	0.015457367105658653	CDKN1A;SERPINE1;ADM;SERPINB8
POU4F1	4/299	0.015457367105658653	NGFR;CDKN1A;ADM;SERPINB8
PPARA	4/299	0.015457367105658653	CDKN1A;SERPINE1;HMOX1;SERPINB8

PRDM8	4/299	0.015457367105658653	CDKN1A;SERPINE1;ADM;SERPINB8
PROP1	4/299	0.015457367105658653	CDKN1A;SERPINE1;ADM;SERPINB8
RAG1	4/299	0.015457367105658653	CDKN1A;SERPINE1;HMOX1;SERPINB8
RAPGEF4	4/299	0.015457367105658653	CDKN1A;PDE2A;PHACTR1;SERPINB8
RBM5	4/299	0.015457367105658653	NXF1;CDKN1A;POLR2A;SF1
RELA	4/299	0.015457367105658653	CDKN1A;POLR2A;SERPINE1;HMOX1
REPIN1	4/299	0.015457367105658653	CDKN1A;MT2A;SERPINB8;SRM
RFX5	4/299	0.015457367105658653	CDKN1A;MT2A;HMOX1;SERPINB8
RHOXF2	4/299	0.015457367105658653	CDKN1A;SERPINE1;ATF5;SERPINB8
RLF	4/299	0.015457367105658653	CDKN1A;HMOX1;ADM;SERPINB8
RNF113A	4/299	0.015457367105658653	CDKN1A;NUDC;CCDC86;PRPF19
RPA4	4/299	0.015457367105658653	CDKN1A;MT2A;ADM;SERPINB8
RXRA	4/299	0.015457367105658653	CDKN1A;MT2A;HMOX1;SERPINB8
RXRG	4/299	0.015457367105658653	NGFR;CDKN1A;SERPINE1;SERPINB8
SALL4	4/299	0.015457367105658653	CDKN1A;SERPINE1;ADM;SERPINB8
SATB1	4/299	0.015457367105658653	CDKN1A;HMOX1;SERPINB8;CORO1A
SCRT2	4/299	0.015457367105658653	NGFR;CDKN1A;ARC;SERPINB8
SHOX2	4/299	0.015457367105658653	CDKN1A;SERPINE1;ADM;SERPINB8
SIM2	4/299	0.015457367105658653	CDKN1A;SERPINE1;ADM;SERPINB8
SMAD3	4/299	0.015457367105658653	CDKN1A;SERPINE1;HMOX1;SERPINB8
SOHLH1	4/299	0.015457367105658653	CDKN1A;ARC;PDE2A;SERPINB8
SOX18	4/299	0.015457367105658653	CDKN1A;PDE2A;ADM;SERPINB8
SOX3	4/299	0.015457367105658653	CDKN1A;MT2A;ADM;SERPINB8
SOX30	4/299	0.015457367105658653	CDKN1A;ARC;ADM;SERPINB8
SP140	4/299	0.015457367105658653	MT2A;HMOX1;SLAMF7;CORO1A
SP5	4/299	0.015457367105658653	NGFR;CDKN1A;ADM;SERPINB8
SP7	4/299	0.015457367105658653	LRRC15;NGFR;CDKN1A;SERPINE1
SPIB	4/299	0.015457367105658653	CDKN1A;SLAMF7;ADM;CORO1A
SSH1	4/299	0.015457367105658653	CDKN1A;MT2A;SERPINE1;SERPINB8
SSRP1	4/299	0.01545736710565	NUDC;PSMD2;PRPF19;SRM

		8653	
STAT3	4/299	0.015457367105658653	CDKN1A;MT2A;SERPINE1;HMOX1
TAL2	4/299	0.015457367105658653	CDKN1A;MT2A;ADM;SERPINB8
TBX10	4/299	0.015457367105658653	NGFR;CDKN1A;SERPINE1;SERPINB8
TBX15	4/299	0.015457367105658653	CDKN1A;SERPINE1;ADM;SERPINB8
TBX20	4/299	0.015457367105658653	CDKN1A;SERPINE1;ADM;SERPINB8
TBX21	4/299	0.015457367105658653	CDKN1A;SLAMF7;ADM;CORO1A
TCF7L2	4/299	0.015457367105658653	CDKN1A;HMOX1;ADM;SERPINB8
TEAD2	4/299	0.015457367105658653	CDKN1A;SERPINE1;ADM;SERPINB8
TEF	4/299	0.015457367105658653	CDKN1A;MT2A;HMOX1;SERPINB8
TERF2	4/299	0.015457367105658653	CDKN1A;POLR2A;PRPF19;SF1
TFEB	4/299	0.015457367105658653	CDKN1A;MT2A;HMOX1;SERPINB8
TGIF2	4/299	0.015457367105658653	CDKN1A;SERPINE1;ADM;SERPINB8
TIGD2	4/299	0.015457367105658653	CDKN1A;HMOX1;ADM;SERPINB8
TIGD5	4/299	0.015457367105658653	CDKN1A;MT2A;ADM;SERPINB8
TP53	4/299	0.015457367105658653	CDKN1A;POLR2A;SERPINE1;HMOX1
TP63	4/299	0.015457367105658653	CDKN1A;SERPINE1;ADM;SERPINB8
TRIM3	4/299	0.015457367105658653	CDKN1A;PDE2A;HMOX1;SERPINB8
TRIM32	4/299	0.015457367105658653	CDKN1A;SERPINE1;ADM;SERPINB8
TSC22D2	4/299	0.015457367105658653	CDKN1A;SERPINE1;ADM;SERPINB8
UBTF	4/299	0.015457367105658653	CDKN1A;POLR2A;PRPF19;SF1
USP39	4/299	0.015457367105658653	NUDC;PSMD2;PRPF19;SRM
WNT8B	4/299	0.015457367105658653	NGFR;CDKN1A;PDE2A;SERPINB8
YY2	4/299	0.015457367105658653	CDKN1A;MT2A;ADM;SERPINB8
ZBED3	4/299	0.015457367105658653	CDKN1A;HMOX1;ADM;SERPINB8
ZBTB3	4/299	0.015457367105658653	CDKN1A;HMOX1;ADM;SERPINB8
ZBTB39	4/299	0.015457367105658653	CDKN1A;POLR2A;BRPF3;SERPINB8
ZBTB4	4/299	0.015457367105658653	CDKN1A;MT2A;POLR2A;SERPINB8
ZBTB43	4/299	0.015457367105658653	CDKN1A;HMOX1;ADM;SERPINB8
ZBTB45	4/299	0.015457367105658653	CDKN1A;MT2A;ADM;SRM

ZBTB5	4/299	0.01545736710565<br>8653	CDKN1A;HMOX1;ADM;SERPINB8
ZBTB9	4/299	0.01545736710565<br>8653	CDKN1A;CCDC86;SERPINB8;BYSL
ZC3H18	4/299	0.01545736710565<br>8653	NUDC;POLR2A;CCDC86;SF1
ZC3H6	4/299	0.01545736710565<br>8653	CDKN1A;HMOX1;ADM;SERPINB8
ZC3H7B	4/299	0.01545736710565<br>8653	CDKN1A;POLR2A;SERPINB8;SF1
ZDHHC19	4/299	0.01545736710565<br>8653	CDKN1A;SERPINE1;ADM;SERPINB8
ZFP2	4/299	0.01545736710565<br>8653	CDKN1A;HMOX1;PHACTR1;SERPINB8
ZFP3	4/299	0.01545736710565<br>8653	CDKN1A;HMOX1;ADM;SERPINB8
ZFP36L2	4/299	0.01545736710565<br>8653	CDKN1A;MT2A;HMOX1;ADM
ZFP37	4/299	0.01545736710565<br>8653	CDKN1A;HMOX1;ADM;SERPINB8
ZFP41	4/299	0.01545736710565<br>8653	NGFR;CDKN1A;ARC;SERPINB8
ZFP82	4/299	0.01545736710565<br>8653	CDKN1A;SERPINE1;ADM;SERPINB8
ZFYVE26	4/299	0.01545736710565<br>8653	CDKN1A;MT2A;HMOX1;SERPINB8
ZIK1	4/299	0.01545736710565<br>8653	CDKN1A;NAP1L3;ADM;SERPINB8
ZMAT5	4/299	0.01545736710565<br>8653	CDKN1A;CCDC86;PRPF19;SERPINB8
ZNF10	4/299	0.01545736710565<br>8653	CDKN1A;NAP1L3;ADM;SERPINB8
ZNF117	4/299	0.01545736710565<br>8653	CDKN1A;HMOX1;ADM;SERPINB8
ZNF133	4/299	0.01545736710565<br>8653	CDKN1A;MT2A;ADM;SERPINB8
ZNF134	4/299	0.01545736710565<br>8653	CDKN1A;HMOX1;ADM;SERPINB8
ZNF143	4/299	0.01545736710565<br>8653	CDKN1A;POLR2A;ADM;SERPINB8
ZNF169	4/299	0.01545736710565<br>8653	CDKN1A;HMOX1;PHACTR1;SERPINB8
ZNF174	4/299	0.01545736710565<br>8653	CDKN1A;POLR2A;SERPINB8;CORO1A
ZNF189	4/299	0.01545736710565<br>8653	CDKN1A;HMOX1;ADM;SERPINB8
ZNF195	4/299	0.01545736710565<br>8653	CDKN1A;ADM;SERPINB8;SF1
ZNF20	4/299	0.01545736710565<br>8653	CDKN1A;SERPINE1;ADM;SERPINB8
ZNF205	4/299	0.01545736710565<br>8653	CDKN1A;MT2A;HMOX1;SERPINB8
ZNF211	4/299	0.01545736710565<br>8653	CDKN1A;HMOX1;ADM;SERPINB8
ZNF213	4/299	0.01545736710565<br>8653	CDKN1A;OSGIN1;HMOX1;SERPINB8
ZNF219	4/299	0.01545736710565<br>8653	CDKN1A;MT2A;ADM;SERPINB8
ZNF22	4/299	0.01545736710565	CDKN1A;MT2A;ADM;SERPINB8

		8653	
ZNF222	4/299	0.015457367105658653	CDKN1A;HMOX1;ADM;SERPINB8
ZNF223	4/299	0.015457367105658653	CDKN1A;MT2A;ADM;SERPINB8
ZNF23	4/299	0.015457367105658653	CDKN1A;HMOX1;ADM;SERPINB8
ZNF25	4/299	0.015457367105658653	CDKN1A;SERPINE1;ADM;SERPINB8
ZNF266	4/299	0.015457367105658653	CDKN1A;ADM;SERPINB8;CORO1A
ZNF280B	4/299	0.015457367105658653	CDKN1A;SERPINE1;ADM;SERPINB8
ZNF311	4/299	0.015457367105658653	CDKN1A;SERPINE1;ADM;SERPINB8
ZNF32	4/299	0.015457367105658653	CDKN1A;HMOX1;ADM;SERPINB8
ZNF333	4/299	0.015457367105658653	CDKN1A;POLR2A;HMOX1;SERPINB8
ZNF341	4/299	0.015457367105658653	CDKN1A;POLR2A;ADM;SERPINB8
ZNF343	4/299	0.015457367105658653	CDKN1A;SPHK1;PHACTR1;SERPINB8
ZNF346	4/299	0.015457367105658653	CDKN1A;POLR2A;SERPINB8;SF1
ZNF350	4/299	0.015457367105658653	CDKN1A;MT2A;HMOX1;SERPINB8
ZNF354B	4/299	0.015457367105658653	CDKN1A;HMOX1;ADM;SERPINB8
ZNF362	4/299	0.015457367105658653	CDKN1A;HMOX1;SERPINB8;SRM
ZNF365	4/299	0.015457367105658653	CDKN1A;SERPINE1;ADM;SERPINB8
ZNF366	4/299	0.015457367105658653	CDKN1A;SERPINE1;PDE2A;SERPINB8
ZNF382	4/299	0.015457367105658653	CDKN1A;HMOX1;ADM;SERPINB8
ZNF383	4/299	0.015457367105658653	CDKN1A;HMOX1;ADM;SERPINB8
ZNF384	4/299	0.015457367105658653	NXF1;CDKN1A;POLR2A;SF1
ZNF415	4/299	0.015457367105658653	NAP1L3;HMOX1;ADM;SERPINB8
ZNF419	4/299	0.015457367105658653	CDKN1A;HMOX1;ADM;SERPINB8
ZNF426	4/299	0.015457367105658653	CDKN1A;HMOX1;ADM;SERPINB8
ZNF430	4/299	0.015457367105658653	CDKN1A;MT2A;ADM;SERPINB8
ZNF436	4/299	0.015457367105658653	CDKN1A;SERPINE1;ADM;SERPINB8
ZNF440	4/299	0.015457367105658653	CDKN1A;SERPINE1;ADM;SERPINB8
ZNF444	4/299	0.015457367105658653	CDKN1A;MT2A;SERPINB8;SRM
ZNF460	4/299	0.015457367105658653	CDKN1A;POLR2A;SERPINE1;ADM
ZNF486	4/299	0.015457367105658653	CDKN1A;MT2A;ADM;SERPINB8

ZNF502	4/299	0.015457367105658653	CDKN1A;HMOX1;ADM;SERPINB8
ZNF511	4/299	0.015457367105658653	MT2A;NUDC;CCDC86;SRM
ZNF512B	4/299	0.015457367105658653	CDKN1A;MT2A;PRPF19;SERPINB8
ZNF526	4/299	0.015457367105658653	SF3B4;CDKN1A;POLR2A;CCDC97
ZNF549	4/299	0.015457367105658653	CDKN1A;MT2A;ADM;SERPINB8
ZNF550	4/299	0.015457367105658653	CDKN1A;HMOX1;ADM;SERPINB8
ZNF554	4/299	0.015457367105658653	CDKN1A;HMOX1;ADM;SERPINB8
ZNF557	4/299	0.015457367105658653	CDKN1A;HMOX1;ADM;SERPINB8
ZNF561	4/299	0.015457367105658653	CDKN1A;SERPINE1;ADM;SERPINB8
ZNF563	4/299	0.015457367105658653	CDKN1A;SERPINE1;ADM;SERPINB8
ZNF575	4/299	0.015457367105658653	CDKN1A;SERPINE1;ADM;SERPINB8
ZNF576	4/299	0.015457367105658653	CCDC86;ADM;SERPINB8;SRM
ZNF583	4/299	0.015457367105658653	CDKN1A;SERPINE1;ADM;SERPINB8
ZNF585A	4/299	0.015457367105658653	CDKN1A;SERPINE1;ADM;SERPINB8
ZNF586	4/299	0.015457367105658653	CDKN1A;CCDC86;HMOX1;CORO1A
ZNF589	4/299	0.015457367105658653	CCDC86;HMOX1;ADM;SERPINB8
ZNF594	4/299	0.015457367105658653	CDKN1A;SERPINE1;ADM;SERPINB8
ZNF598	4/299	0.015457367105658653	CDKN1A;POLR2A;CCDC86;SRM
ZNF623	4/299	0.015457367105658653	CDKN1A;HMOX1;ADM;SERPINB8
ZNF627	4/299	0.015457367105658653	CDKN1A;HMOX1;ADM;SERPINB8
ZNF629	4/299	0.015457367105658653	CDKN1A;SERPINE1;ADM;SERPINB8
ZNF646	4/299	0.015457367105658653	CDKN1A;POLR2A;ADM;CORO1A
ZNF653	4/299	0.015457367105658653	CDKN1A;ADM;ATF5;SERPINB8
ZNF671	4/299	0.015457367105658653	CDKN1A;HMOX1;ADM;SERPINB8
ZNF674	4/299	0.015457367105658653	CDKN1A;MT2A;ADM;SERPINB8
ZNF684	4/299	0.015457367105658653	CDKN1A;MT2A;ADM;SERPINB8
ZNF689	4/299	0.015457367105658653	CCDC86;ADM;PRPF19;SERPINB8
ZNF691	4/299	0.015457367105658653	CDKN1A;HMOX1;ADM;SERPINB8
ZNF699	4/299	0.015457367105658653	CDKN1A;SERPINE1;ADM;SERPINB8
ZNF705A	4/299	0.01545736710565	CDKN1A;HMOX1;SERPINB8;CORO1A

		8653	
ZNF738	4/299	0.015457367105658653	CDKN1A;MT2A;ADM;SERPINB8
ZNF763	4/299	0.015457367105658653	CDKN1A;HMOX1;ADM;SERPINB8
ZNF773	4/299	0.015457367105658653	CDKN1A;HMOX1;ADM;SERPINB8
ZNF786	4/299	0.015457367105658653	CDKN1A;HMOX1;ADM;SERPINB8
ZNF787	4/299	0.015457367105658653	CDKN1A;SPHK1;CCDC86;SRM
ZNF792	4/299	0.015457367105658653	CDKN1A;HMOX1;ADM;SERPINB8
ZNF843	4/299	0.015457367105658653	NGFR;CDKN1A;PDE2A;SERPINB8
ZNF846	4/299	0.015457367105658653	CDKN1A;SERPINE1;HMOX1;SERPINB8
ZNF91	4/299	0.015457367105658653	CDKN1A;MT2A;ADM;SERPINB8
ZRSR1	4/299	0.015457367105658653	CDKN1A;MT2A;HMOX1;SERPINB8
ZSCAN10	4/299	0.015457367105658653	CDKN1A;MT2A;ADM;SERPINB8
ZSCAN16	4/299	0.015457367105658653	MT2A;HMOX1;ADM;SERPINB8
ZXDB	4/299	0.015457367105658653	CDKN1A;SERPINE1;HMOX1;SERPINB8
